# Design, Synthesis and Evaluation of Praziquantel Analogues and New Molecular Hybrids as Potential Antimalarial and Anti-Schistosomal Agents

**DOI:** 10.3390/molecules28135184

**Published:** 2023-07-03

**Authors:** Freddy Mugisho Kasago, Cécile Häberli, Jennifer Keiser, Wayiza Masamba

**Affiliations:** 1 Department of Chemical and Physical Sciences, Faculty of Natural Sciences, Walter Sisulu University, Nelson Mandela Drive, Mthatha 5117, South Africa; freddymkasago@gmail.com; 2 Department of Medical Parasitology and Infection Biology, Swiss Tropical and Public Health Institute, Kreuzstr. 2, CH-4123 Allschwil, Switzerland; cecile.haeberli@swisstph.ch (C.H.); jennifer.keiser@swisstph.ch (J.K.)

**Keywords:** antimalarial, cinnamic acids, molecular hybrids, praziquantel, *S. mansoni*, schistosomiasis

## Abstract

Malaria and schistosomiasis are two of the neglected tropical diseases that persistently wreak havoc worldwide. Although many antimalarial drugs such as chloroquine are readily available, the emergence of drug resistance necessitates the development of new therapies to combat this disease. Conversely, Praziquantel (PZQ) remains the sole effective drug against schistosomiasis, but its extensive use raises concerns about the potential for drug resistance to develop. In this project, the concept of molecular hybridization was used as a strategy to design the synthesis of new molecular hybrids with potential antimalarial and antischistosomal activity. A total of seventeen molecular hybrids and two PZQ analogues were prepared by coupling 6-alkylpraziquanamines with cinnamic acids and cyclohexane carboxylic acid, respectively. The synthesised compounds were evaluated for their antimalarial and antischistosomal activity; while all of the above compounds were inactive against *Plasmodium falciparum* (IC_50_ > 6 µM), many were active against schistosomiasis with four particular compounds exhibiting up to 100% activity against newly transformed schistosomula and adult worms at 50 µM. Compared to PZQ, the reference drug, the activity of which is 91.7% at 1 µM, one particular molecular hybrid, compound **32**, which bears a para-isopropyl group on the cinnamic acid moiety, exhibited a notable activity at 10 µM (78.2% activity). This compound has emerged as the front runner candidate that might, after further optimization, hold promise as a potential lead compound in the fight against schistosomiasis.

## 1. Introduction

In 2021, the World Health Organisation (WHO) reported 240 million schistosomiasis cases worldwide with 700 million people living in regions classified as endemic [1,2], a real health threat in developing countries. Humans are infected by contact with contaminated freshwater, either by swimming or working in infected water bodies. Once inside the human body, the adult worms settle in the blood vessels and can live there for years, undetected by the immune system while excreting hundreds to thousands of eggs daily, which either leave the body in excreta or become trapped in nearby tissues [3,4,5]. The disease can lead to a range of complications, including liver and spleen damage, kidney failure, bladder cancer, and increased susceptibility to other infections, as well as death if left untreated [6].

Efforts to control and eliminate the disease in Africa and the world have been ongoing for many years. These include prevention measures by improving sanitation and hygiene, and using medication to treat infected individuals [7]. In the absence of any vaccine against schistosomiasis, chemotherapy has remained the only option for its control. Praziquantel (PZQ) [8] emerged as the only drug amongst many compounds screened for their anthelmintic activity. The drug is typically administered as a racemate; however, the active enantiomer, (R)-PZQ, exhibits reduced side effects and can be obtained through either classical resolution [9] or asymmetric synthesis [9,10]. Due to its efficacy, safety, operational convenience, and low cost, this drug is extensively and exclusively used today with no viable alternative. Unfortunately, this is a recipe for the development of the phenomenon of resistance [11].

Despite its limitations, such as its efficacy on only 1–2-week-old larvae and 5-week and older adult worms, and its inability to kill immature worms 3–4 weeks post infection, PZQ can still serve as a valuable scaffold for molecular hybridization in the development of new lead compounds to combat schistosomiasis.

**Figure 1:**
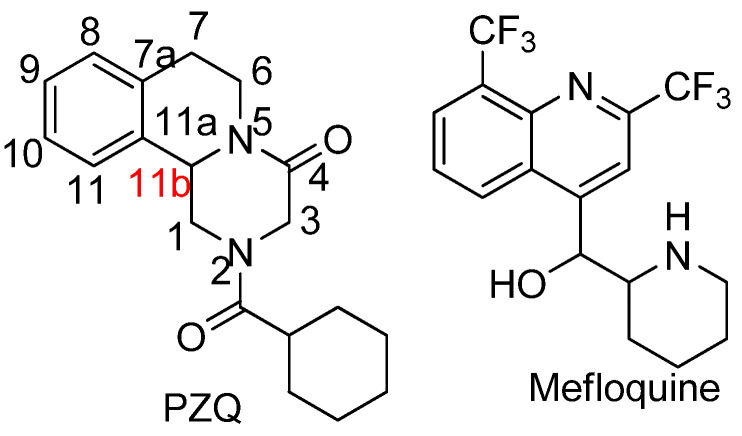
Chemical structure of PZQ including atom numbering (in red, the chiral center of the molecule [25] and the structure of mefloquine.

On the other hand, malaria, a life-threatening disease caused by *Plasmodium* parasites transmitted through the bites of infected mosquitoes, continues to be a significant global health concern. Current treatments for malaria primarily rely on artemisinin-based combination therapies (ACTs) [12], which combine artemisinin derivatives with other antimalarial drugs. While ACTs have proven effective in reducing mortality rates, the emergence and spread of drug-resistant strains of *Plasmodium* pose a major challenge [13]. The development of resistance to artemisinin in Southeast Asia and the potential for its spread to other regions underscores the urgent need for novel antimalarial drugs. Additionally, ensuring the accessibility and affordability of these treatments in resource-limited settings remains a critical obstacle in combating malaria worldwide. Mefloquine (see Figure 1) is an antimalarial drug structurally related to quinine with high worm burden reduction (93%) in the *S. mansoni* mouse model [14]. Given the similarities between the PZQ basic structure with the antimalarial quinoline and isoquinoline pharmacophores, two well-known malaria drug scaffolds, it was anticipated that the modified PZQ analogues and molecular hybrids would be active against *Plasmodium falciparum* in addition to their expected antischistosomal activity.

Molecular hybridization as a drug discovery strategy involves the rational design of new chemical entities by the fusion (usually via a covalent linker) of two or more drugs, both active compounds and/or pharmacophoric units recognised and derived from known bioactive molecules [15,16,17]. The expected outcome for this chemical modification is to produce a new hybrid compound with improved affinity and efficacy, when compared to the parent drugs. Additionally, this strategy can result in compounds presenting a modified selectivity profile, different and/or dual modes of action, and reduced undesired side effects, ultimately, leading to new therapies.

Molecular hybrids in their general form represent intra-molecular combined therapies where the previously independent biologically active principles that act at different targets are brought together by a covalent link in order to combine the effects of each molecule and address the complexity deriving from a plethora of biochemical and physiological processes involved in a particular pathology [15,18,19]. This concept of hybridization of biologically active molecules has been used by numerous research groups to target a variety of diseases including cancer, malaria, tuberculosis, and HIV/AIDS, as well as schistosomiasis, amongst others [19,20,21]. 

Based on precedents in the literature [22], molecular hybridization is expected to accelerate the discovery of new lead compounds for the treatment of schistosomiasis.

Cinnamic acid and derivatives have demonstrated a wide range of biological activities in different studies and had been reported to have significant activity against malaria parasites [23]. Their incorporation in the PZQ tetrahydroisoquinoline nucleus, a well-known malaria scaffold, is anticipated to confer antimalarial activity to these new molecular entities. Indeed, several chemical entities with isoquinoline structure isolated from medicinal plants have been reported to demonstrate good antiplasmodial activity comparable to chloroquine taken as a reference [24]. 

The work of Liu et al. has shown that substitution on position C3 (see Figure 1) of PZQ maintains the two aromatic rings in a non-coplanar conformation, which leads to a complete loss of biological activity. Hence, the substitution at C6, which so far has remained unexplored, might contribute to the overall optimum activity by maintaining the coplanarity of the cinnamic acid moiety with the other aromatic ring.

**Scheme 1:**
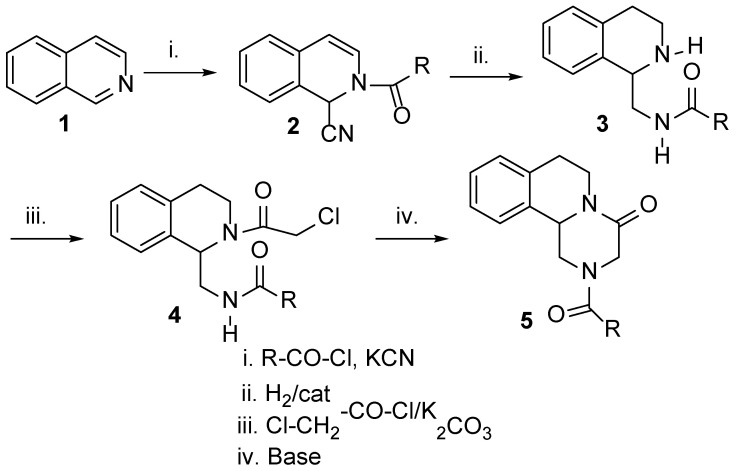
Merck synthesis of PZQ.

The initial synthesis of praziquantel was achieved by Merck, starting with the Reissert reaction of the readily available isoquinoline core **1**, followed by a high-pressure catalytic hydrogenation step to generate an amine intermediate **3**. The subsequent acylation of the latter with chloroacetyl chloride followed by a base-catalyzed cyclisation resulted in the formation of the target compound **5** [26]. The synthetic pathway is depicted in Scheme 1 below:

This approach was found to be limited in two aspects: firstly, the Reissert reaction, the initial step of the process, could only be carried out with a restricted number of acid chlorides, and secondly, the catalytic hydrogenation of **3** requires high pressure (70 atm) [27] in order to generate a 1-aminomethyltetrahydroisoquinoline intermediate [26].

The merit of the Merck approach is the use of inexpensive and readily available raw materials. For this reason, it is still widely used for the large-scale production of PZQ [28]. In addition, the use of toxic cyanide is environmentally unfriendly and the overall yield of the reaction has not been reported. 

**Scheme 2:**
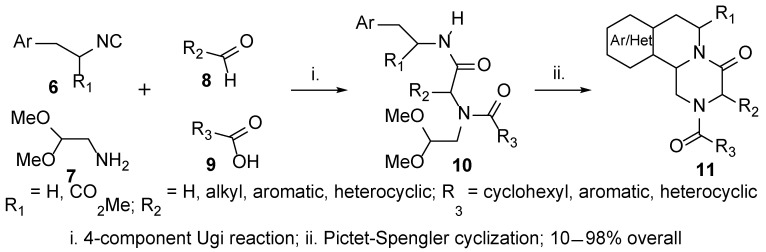
Four-component Ugi synthesis of PZQ derivatives.

A highly efficient method for the synthesis of PZQ derivatives using a two-step, one-pot procedure that involves a four-component Ugi reaction followed by the Pictet–Spengler reaction was described by Liu et al. [29,30] (Scheme 2). This approach offers significant advantages in achieving a wide range of structural diversity, thanks to the extensive availability of commercially accessible carboxylic acids and aldehydes. However, while elegant and convergent, the broader applicability of this approach is hindered by the limited availability of the required isocyanides **6**.

**Scheme 3:**
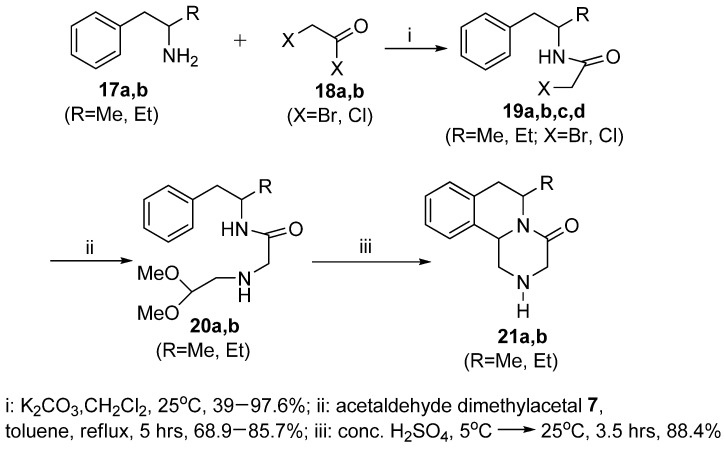
Synthesis of praziquanamines **1**.

A third widely used strategy for the preparation of PZQ is outlined in Scheme 3 [31]. This approach involves a three-step synthesis of piperazinone **21** (R = H), where the formation of both the piperazine and isoquinoline rings simultaneously occurs in a one-pot procedure. By acylating with cyclohexanecarbonyl chloride, the desired final product is obtained. One notable advantage of this synthetic pathway is its ability to introduce molecular diversity through the acylating agent. As a result, numerous alternative methods that rely on the formation of the tetrahydroisoquinoline core have emerged in the literature [30,32].

The primary objective of this project is to create new molecular hybrids by combining the two aforementioned scaffolds, with the aim of discovering potential treatments for schistosomiasis.

## 2. Results

### 2.1. Chemistry

An initial attempt was undertaken to utilize the four-component Ugi reaction, starting from amphetamine-derived isonitrile **6a** (Ar = Ph, R_1_ = Me), formaldehyde **8a** (R2 = H), trans-cinnamic acid **9a** (R_3_ = PhCH = CH-), and aminoacetaldehyde dimethyl acetal **7**, proved unsuccessful as the anticipated product was not detected after the reaction. This outcome can be attributed to the limited solubility of paraformaldehyde within the given reaction conditions (methanol, 0 °C). As a result, our focus was redirected toward the approach by Kim et al. [31].

**Scheme 4:**
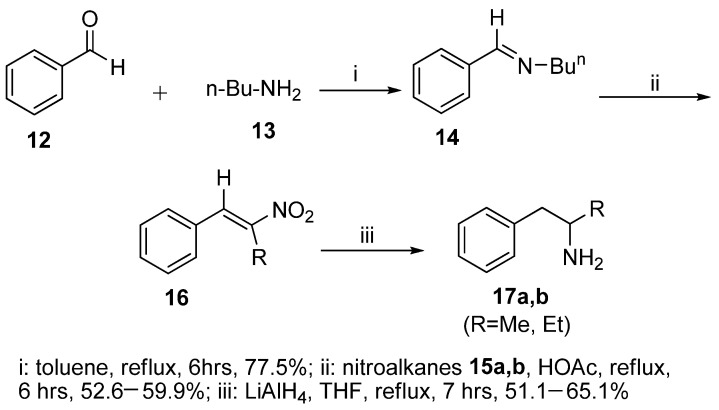
Synthesis amphetamine and 1-phenylbutan-2-amine.

The key intermediates in this strategy, 6-Alkylpraziquanamines **21a,b** (R = Me and Et), were prepared using a two-step procedure involving the condensation of benzaldehyde **12** with n-butylamine **13** to generate the corresponding Schiff base **14** (benzaldehyde imine), followed by reaction with the appropriate nitroalkane (nitroethane **15a** and 1-nitropropane **15b**, respectively) to obtain the expected nitroamines in an aza-Henry reaction (Scheme 4). The resulting nitroamines were not isolated and underwent intramolecular deamination under acidic conditions to yield 2-nitro-1-phenylalkenes intermediates **16a,b** [33]. The two anticipated nitroalkenes **16** were obtained as trans-isomers, known for their greater thermodynamic stability than their cis-isomers, as evidenced by the chemical shifts of their olefinic protons (8.09 ppm for [(1E)-2-nitroprop-1-en-1-yl]benzene **16a** and 8.00 ppm for [(1E)-2-nitrobut-1-en-1-yl]benzene **16b**) (see Scheme 4). Although the Schiff base formation is a straightforward, high-yielding reaction step, the overall moderate yield of 52.6–59.9% in this two-step process can be attributed to the extensive polymerization of the highly reactive nitroalkenes under the reaction conditions. Indeed, a considerable amount of resinous materials and decomposition products were formed during this reaction. In order to synthesize 1-phenylpropan-2-amine (amphetamine) **17a** and 1-phenylbutan-2-amine **17b**, which are the methyl and ethyl analogues of phenylethylamine, respectively, the aforementioned nitroalkenes **16** underwent a reduction implementing the procedure described by Collins and colleagues [26]. The use of LiAlH_4_ as a reducing agent resulted in moderate yields of the two corresponding amines **17** (51.1% and 65.1%). The moderate yield observed in these two reactions may be attributed to the potential formation of complexes between the amine functionality and the aluminum cation, generating a dense emulsion during the work-up process. Consequently, the extraction and recovery of the product became considerably challenging. Attempts to reduce these nitroalkenes **16** by hydrogenation over nickel (Ra-Ni) and Pt catalysts under moderate or high pressures, as well as reduction with NaBH_4_/Ni(OAc)_2_ [34], were unsuccessful. Out of all the reduction methods evaluated, only reduction using LiAlH_4_ in dry THF under reflux in a nitrogen atmosphere for 7 h yielded the expected products.

Access to the alkylated praziquanamine derivatives **21a,b** was achieved by a three-step procedure. First, the abovementioned amines were condensed with either bromoacetyl bromide **18a** (X = Br) or chloroacetyl chloride **18b** (X = Cl) following the procedure of Kim et al. outlined above [31]. Then, the intermediates **19** obtained in the above reaction were reacted with aminoacetaldehyde dimethylacetal **7** to afford intermediates **20a,b**. Finally, intermediates **20a,b** were subjected to Pictet-Spiengler cyclisation (Scheme 3), resulting in the formation of the praziquanamine derivatives **21a,b**.

The first condensation between bromoacetyl bromide **18a** (X = Br) with amphetamine **17a** (R = Me) led to the formation of the expected intermediate 2-bromo-N-(1-phenylpropan-2-yl)acetamide **19ac** (R = Me, X = Br) in 97.6% yield. In the subsequent step, intermediate **19ac** underwent an aminoalkylation reaction with aminoacetaldehyde dimethylacetal **7** to afford intermediate 2-[(2,2-dimethoxyethyl)amino]-N-(1-phenylpropan-2-yl)acetamide **20a** (R = Me) in 85.7% yield. Finally, treatment of **20a** with sulphuric acid produced the desired product, 6-methylpraziquanamine **21a** (R = Me) in 88.4% yield. 

Condensing phenylbutyl amine **17b** (R = Et) with chloroacetyl chloride **18b** (X = Cl) resulted in the formation of 2-chloro-N-(1-phenylbutan-2-yl)acetamide **19bd** (R = Et, X = Cl) in 39.0% yield; this was followed by the aminoalkylation of **19bd** with amino acetaldehyde dimethylacetal **7** to afford 2-[(2,2-dimethoxyethyl)amino]-N-(1-phenylbutan-2-yl)acetamide **20b** in 68.9% yield. In the condensation of **19** with acetaldehyde dimethyl acetal **7**, a minimum of two equivalents of the latter were required, one of which served as a base to neutralize the in-situ-generated acid (HBr or HCl).

**Scheme 5:**
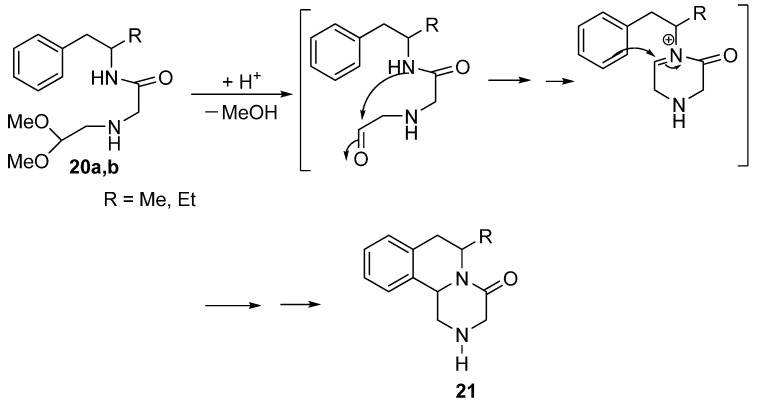
Pictet-Spengler cyclisation of the iminium intermediate.

Upon cleavage of the dimethoxy-protecting groups with concentrated H_2_SO_4_, the aldehyde group was released and underwent concomitant Pictet-Spengler cyclisation, ultimately yielding 6-methyl-1,2,3,6,7,11b-hexahydro-4H-pyrazino[2,1-a]isoquinolin-4-one **21a** (R = Me) and the 6-ethyl-1,2,3,6,7,11b-hexahydro-4H-pyrazino[2,1-a]isoquinolin-4-one **21b** (R = Et) in 88.4 and 35.9% yields, respectively. This intramolecular cyclisation involves the intermediary formation of a reactive iminium cation, which is attacked by the neighboring aromatic ring nucleophile (see Scheme 5).

The yield of 6-Ethyl-1,2,3,6,7,11b-hexahydro-4H-pyrazino[2,1-a]isoquinolin-4-one **21b** (R = Et) was lower than its methyl analogue, possibly due to the steric hindrance caused by the bulkier ethyl group compared to the methyl group.

**Scheme 6:**
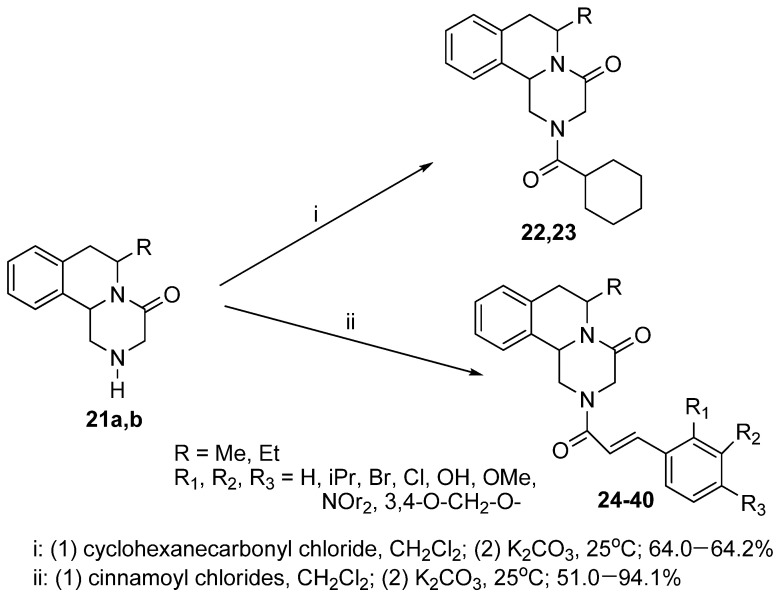
Synthesis of praziquantel analogues and molecular hybrids.

The coupling reaction between the above 6-alkylpraziquanamine derivatives **21a,b** with cyclohexanecarbonyl chloride and the relevant cinnamoyl chlorides proceeded under anhydrous conditions and afforded good yields of the new PZQ analogues (**22,23**) and molecular hybrids (**24–40**), respectively, according to Scheme 6 below:

The results obtained are disclosed in Table 1 below.

### 2.2. Biological Activity

The antiplasmodium activity of the above 19 compounds was assessed at the Drug Discovery and Development Centre (H3D), the University of Cape Town, while the antischistosomal activity was evaluated at the Swiss Tropical and Public Health Institute (Basel, Switzerland). The results of antiplasmodium activity are reported in the Supplementary Materials [35,36], while Table 2 below summarizes the results of the antischistosomal evaluation. At 50 µM, six compounds showed high activity (85–100%) against NTS. At a lower concentration (10 µM), these compounds revealed only low to moderate activity. The six compounds progressed into tests against adult *S. mansoni*. At 50 µM, four compounds revealed high activity (77–100% activity) and compound **32** also showed high activity at 10 µM. An IC_50_ value of 1.9 µM was determined for this compound.

## 3. Discussion

Condensation between 6-alkylpraziquanamines **21a,b** with the relevant acyl chlorides to afford PZQ analogues **22** and **23** and molecular hybrids **24–40** was carried out under standard conditions and smoothly led to the expected compounds in good to excellent yields (see Table 1), save for molecular hybrid **30**, obtained in a moderate yield (51.0%). This yield can be improved after optimization.

Contrary to our expectation, none of the nineteen molecular hybrids and PZQ analogues showed any significant activity against *P. falciparum*. Given the similarity between the praziquanamine core and the quinoline scaffold, based on the structure-activity relationship and since both *Plasmodium* and *Schisotosoma* parasites share the same redox-heme iron mechanism, we expected a plausible in vitro activity against both malaria and schistosomiasis. Their IC_50_ values are all above 6 µM (see Supplementary Materials), compared to 0.005 and 0.009 µM for artesunate and chloroquine, respectively.

These results confirm that the activity exhibited by certain antimalarial drugs, such as mefloquine, involves a mechanism different from that of the praziquanamine core [14].

Equally surprising was the low activity of PZQ analogues **22** and **23** against schistosomes with an activity of 43.3% and 75% against schistosomula for **22** and **23**, respectively. The low and moderate activity of **22** and **23**, moving from a methyl to an ethyl group, can be explained by precedents in the literature that demonstrated that substitution at the C6 position will affect PZQ activity, which may increase with further steric hindrance (in this case brought about by the extra methylene group). Indeed, the activity of **23** is almost double (1.7 times higher) that of **22** at 50 µM and 1.9 times higher than the activity of **22** at 10 µM.

A bromine atom substitution in the ortho position in combination with a fluorine atom in the para position, as in **31**, enhances the activity (100% at 50 µM), while a chlorine atom substitution in the ortho position leads to a loss of activity (see compound **30**, the activity of which stands at 63.3% at 50 µM). The para substitution seems to favour the activity; compared to **25**, which bears a chlorine atom in the para position, **30** has a lower activity, while **35** exhibits a high activity of 100% at 50 µM.

Compound **36**, with a nitro group on the para position of the cinnamic acid moiety, shows a high activity of 100% at 50 µM, while the nitro group on the meta position, as in **29**, leads to a loss of activity.

Comparing **39** and **40**, each of which contains two methoxy groups, shows that activity increases when one methoxy group is located at the meta position and the other is at the para position. Conversely, activity decreases when one methoxy group is positioned at the ortho position and the other is at the para position.

Most of the compounds that exhibit the highest level of activity, namely **32**, **35**, **36**, and **40**, all share a common feature: a para substitution. This substitution appears to be a privileged position that enhances the activity of these compounds. A fluorine atom in the para position seems to exhibit the opposite effect, decreasing the activity, as in **26** and **37**, suggesting that the high activity in **31** might solely originate from the contribution of the bromine atom.

Overall, seven molecular hybrids exhibited significant in vitro activity against schistosomes (both NTS as well as adult *S. mansoni*).

Compound **32** showed the highest activity with an IC_50_ value of 1.9. In comparison, the IC_50_ value for the reference (PZQ) is 0.54 µM [36,37].

This compound appears to be the front runner of all the active compounds, followed by **31**, **35**, and **40**. It can be noted that compounds **32**, **35**, and **40** are all derived from cinnamic acid bearing a substituent on the para position, with an isopropyl group inducing the highest activity. The above four molecular hybrids are potential new leads in the fight against schistosomiasis and will be optimised for better activity.

## 4. Materials and Methods

### 4.1. General

The progress of reactions was monitored by thin-layer chromatography (TLC) using a UV Labex lamp supplied by Tallaboratories to visualise spots on TLC silica gel 60F254 plates under short and medium wavelength light at 254 and 312 nm, respectively. The target compounds were purified by column chromatography using silicagel 60, Merck Kieselgel 60 (70–230). Eluents were prepared by mixing distilled solvents, especially n-hexane, ethyl acetate, dichloromethane (DCM), and diethylether, depending on the properties of the product. The purified products were characterised by ^1^HNMR and ^13^C-NMR spectra recorded on a 400 MHz Varian NMR spectrometer. Chemical shifts (d) are given in ppm, relative to the signal of tetramethylsilane (TMS); and coupling constants (*J*) are given in Hertz. IR spectra were recorded on a PerkinElmer Spectrum One; peak frequencies are given in cm^−1^. Mass spectrometry was determined on an Agilent GC-MS instrument (6890N, Agilent Technologies). Separation was performed on a non-polar ZB-5Ms (30 m, 0.25 mm ID, 0.25 µm film thickness) Zebron 7HG-G010-41 capillary column. Helium was used as the carrier gas at a flow rate of 1 mL/min. The injector temperature was maintained at 240 °C. An amount of 1 µL of the sample was injected in a 20:1 split ratio. The oven temperature was programmed as follows: 100 °C for 5 min, ramped to 330 °C at a rate of 20 °C/min for 10 min. Mass spectra were obtained by electron impact ionisation (EI).

### 4.2. In Vitro Antischistosomal Assays; Screening on Newly Transformed Schistosomula (NTS)

*S. mansoni cercariae* were mechanically changed to newly transformed schistosomula as described elsewhere [38]. Briefly, snails were placed under light to stimulate cercarial shedding, and the cercarial suspension was collected. The tails were separated from the heads by rinsing three times with cold HBSS. NTS were then incubated overnight in a culture medium and used the next day. Test compounds and controls were dissolved in DMSO (Fluka, Buchs, Switzerland) to a concentration of 10 mM. One hundred NTS were then incubated in each well of a 96-well plate with culture medium and the test compound for a final well volume of 250 μL. The culture medium was composed of Medium 199 (Invitrogen, Carlsbad, CA, USA) supplemented with 5% fetal calf serum (Lucerne, Switzerland) and a 1% penicillin/streptomycin mixture (Lucerne, Switzerland). Compounds were tested at 10 and 50 μM in triplicate and repeated once with NTS incubated in no more than 1% DMSO. NTS incubated in 1% DMSO and PZQ served as negative and positive controls. NTS were kept in an incubator at 37 °C and 5% CO_2_ for up to 72 h, after which the condition of the NTS was microscopically evaluated using a scale from 3 (normal activity and morphological alteration) to 0 (dead).

### 4.3. Adult S. mansoni Worms

To obtain adult schistosomes, mice were subcutaneously infected with 80–100 cercariae. The mice were then euthanised after 7–8 weeks with CO_2_, and the worms were collected from the hepatic portal and mesenteric veins. Two pairs of adult worms were incubated in each well of a 24-well plate with 2 mL of a culture medium and the test compound. The culture medium was composed of RPMI 1640 (Invitrogen, Carlsbad, CA, USA) supplemented with 5% fetal calf serum (Lucerne, Switzerland) and a 1% penicillin/streptomycin mixture (Lucerne, Switzerland). Compounds with >70% activity against NTS were evaluated against adult worms at 1, 10, and 50 μM with incubation at <1% DMSO. NTS incubated in 1% DMSO and PZQ served as negative and positive controls. Worms were kept in an incubator at 37 °C and 5% CO_2_ for up to 72 h, after which their condition was microscopically evaluated using a scale from 3 (normal activity and no morphological alterations) to 0 (dead) [39]. All animal experiments were approved by Swiss national and cantonal authorities (permit 2070).

*6-Methylpraziquanamine* **21a**: to 2-[(2,2-dimethoxyethyl)amino]-N-(1-phenylpropan-2-yl)acetamide (1.77 g; 6.3 mmole) was added drop wise to a solution of concentrated sulphuric acid (1.68 mL; 32.8 mmoles, 5.2 eqv) at 5 °C. After stirring at room temperature for 3.5 h, the reaction mixture was poured into ice water and the pH was adjusted to 12 with 25% aqueous NaOH solution with cooling. The mixture was extracted with ethyl acetate and the ethyl acetate solution was washed with brine, dried (Na_2_SO_4_), and concentrated to give a brown viscous oil (1.20 g; 88.4%). The product was purified by column chromatography using methanol as the mobile phase. ^1^H NMR (400 MHz, CDCl_3_) δ 7.25–7.13 (m, 2H), 7.10 (dq, *J* = 6.6, 3.8 Hz, 2H), 5.34–5.22 (m, 1H), 4.70 (dd, *J* = 9.9, 4.4 Hz, 1H), 3.71–3.55 (m, 2H), 3.48 (dd, *J* = 17.3, 0.8 Hz, 1H), 3.39 (t, *J* = 0.7 Hz, 1H), 3.07 (dd, *J* = 16.1, 5.8 Hz, 1H), 2.86 (ddd, *J* = 12.8, 9.8, 0.9 Hz, 1H), 2.57–2.48 (m, 1H), 1.09 (d, *J* = 6.9 Hz, 3H). ^13^C NMR (101 MHz, CDCl_3_) δ 166.87, 132.94, 132.54, 129.91, 127.05, 126.31, 124.34, 52.59, 49.91, 49.48, 41.20, 34.14, 16.44. FT-IR (cm^−1^): 2936.69, 1647.96, 1510.48, 1469.39, 1139.65, 1046.87, 741.74, 711.81.

*6-Ethylpraziquanamine* **21b**: was obtained using a similar procedure as that of 6-methylpraziquanamine, starting from the corresponding N-[(2-phenyl)ethyl[(2,2-dimethoxyethyl)amino] acetamide (1.76 g), and yielded a brown viscous oil (0.49 g; 35,9%). The product was purified by column chromatography (on alumina) with n-hexane and ethyl acetate mixture (2:1) as eluent; ^1^H NMR (400 MHz, CDCl_3_) δ 7.28–7.09 (m, 4H), 5.15–5.05 (m, 1H), 4.67 (dd, *J* = 10.1, 4.3 Hz, 1H), 3.74–3.65 (m, 2H), 3.56 (d, *J* = 17.5 Hz, 1H), 3.08 (dd, *J* = 16.3, 5.8 Hz, 1H), 2.88 (dd, *J* = 12.9, 10.0 Hz, 1H), 2.65 (dd, *J* = 16.3, 1.9 Hz, 1H), 1.97–1.90 (m, 1H), 1.44 (m, 2H), 0.91 (t, *J* = 7.4 Hz, 3H). ^13^C NMR (101 MHz, CDCl_3_) δ 167.37, 133.20, 132.89, 130.03, 127.12, 126.40, 124.58, 53.19, 50.13, 49.97, 47.06, 32.42, 23.25, 10.70. FT-IR (cm^−1^): 2921.67; 1633.86; 1495.46; 1454.35; 1305.48; 1124.61; 1030.84; 739.64; 699.73.

General procedure for the synthesis of praziquantel analogues and molecular hybrids: the coupling process was performed following a general procedure whereby cyclohexanecarbonyl chloride or the corresponding cinnamoyl chlorides were condensed with 6-alkylpraziquanamines. The acyl chlorides were prepared in a separate flask by the dropwise addition of thionyl chloride to a mixture of the corresponding acid in dry DCM. When the TLC showed that the reaction was complete, the solvent was evaporated, including excess thionyl chloride, and the residue was redissolved in 1 millilitre of dry dichloromethane. The resulting solution was added to a mixture of potassium carbonate (1.1 eqv) and the substrate (6-alkylpraziquanamine, 1–5 mmoles) in 2 mL of dry dichloromethane. The solution was stirred at RT until the TLC showed that the reaction was complete. The product was then filtered off, evaporated, and purified by column chromatography.

*2-(Cyclohexylcarbonyl)-6-methyl-1,2,3,6,7,11b-hexahydro-4H-pyrazino[2,1-a]isoquinolin-4-one, 6-methylpraziquantel* **22**; colourless crystals (0.10 g, 68.3%); m.p. 133–135 °C; Rf = 0.365 (n-hexane-ethyl acetate, 1:1); ^1^H NMR (400 MHz, CDCl_3_) δ 7.23–7.25 (m, 4H), 5.22 (s, 1H), 4.96 (d, *J* = 13.7 Hz, 1H), 4.71 (d, *J* = 10.0 Hz, 1H), 4.45–4.36 (m, 1H), 4.10 (d, *J* = 17.3 Hz, 1H), 3.12 (dd, *J* = 16.0, 5.7 Hz, 1H), 3.00–2.89 (m, 1H), 2.60 (d, *J* = 16.1 Hz, 1H), 2.45 (t, *J* = 12.0 Hz, 1H), 1.58–1.44 (m, 2H), 1.25 (d, *J* = 11.0 Hz, 7H), 1.14–1.04 (m, 4H). ^13^C NMR (101 MHz, CDCl_3_) δ 174.84, 164.18, 132.49, 131.80, 129.91, 127.68, 126.83, 125.28, 51.53, 49.00, 45.10, 42.50, 40.78, 34.29, 29.09, 28.97, 25.67, 16.96. FT-IR (cm^−1^): 3748.29, 2925.97, 2007.79, 1735.02, 1623.12, 1435.91, 1327.90, 1225.25, 759.60. GC-MS (EI): tR = 15.747 min, MW = 326.4, *m*/*z*[M^+^] = 326.2.

*2-Cyclohexanecarbonyl-6-ethyl-1,2,3,6,7,11b-hexahydro-pyrazino[2,1-a]isoquinolin-4-one, 6-ethylpraziquantel* **23**; yellow crystals (0.10 g, 64.2%); Rf = 0.483 (n-hexane-ethyl acetate, 1:1); ^1^H NMR (400 MHz, CDCl_3_) δ 7.27–7.24 (m, 4H), 7.14 (d, *J* = 6.8 Hz, 2H), 5.06 (d, *J* = 14.6 Hz, 2H), 4.68 (d, *J* = 10.9 Hz, 1H), 4.47 (d, *J* = 17.4 Hz, 1H), 4.11 (d, *J* = 17.5 Hz, 1H), 3.10 (dd, *J* = 16.3, 5.9 Hz, 1H), 2.87 (dd, *J* = 13.4, 10.4 Hz, 1H), 2.70 (d, *J* = 16.1 Hz, 1H), 2.47 (t, *J* = 11.9 Hz, 1H), 1.96 (s, 2H), 1.58–1.51 (m, 1H), 1.44 (tt, *J* = 14.3, 6.6 Hz, 1H), 1.34–1.21 (m, 6H), 0.89 (dt, *J* = 17.6, 7.4 Hz, 3H). ^13^C NMR (101 MHz, CDCl_3_) δ 174.79, 164.60, 132.62, 131.94, 129.88, 127.59, 126.77, 125.32, 51.78, 49.07, 48.15, 45.31, 40.78, 32.36, 29.26, 28.92, 25.71, 25.64, 10.65. FT-IT (cm^−1^): 2927.16, 2848.30, 1650.76, 1634.25, 1411.13, 1317.89, 1211.14, 1069.25, 759.32, 725.04. GC-MS (EI): tR = 15.989 min, MW = 340.4, *m*/*z*[M^+^] = 340.2.

*6-Methyl-2-[(2E)-3-phenylprop-2-enoyl]-1,2,3,6,7,11b-hexahydro-4H-pyrazino[2,1-a]isoquinolin-4-one* **24**; yellow crystals (0.12 g, 73.4%); m.p. 96–101 °C; Rf = 0.30 (diethyl ether); ^1^H NMR (400 MHz, CDCl_3_) δ 7.76 (d, *J* = 15.4 Hz, 1H), 7.55–7.52 (m, 2H), 7.41–7.37 (m, 3H), 7.35 (s, 1H), 7.33–7.22 (m, 2H), 7.17 (s, 1H), 6.82 (d, *J* = 15.3 Hz, 1H), 5.26 (s, 1H), 5.09 (d, *J* = 13.3 Hz, 1H), 4.82 (s, 1H), 4.58 (d, *J* = 17.6 Hz, 1H), 4.25 (d, *J* = 17.1 Hz, 1H), 3.19–3.03 (m, 2H), 2.62 (d, *J* = 16.0 Hz, 1H), 1.12 (d, *J* = 6.9 Hz, 3H). ^13^C NMR (101 MHz, CDCl_3_) δ 165.18, 163.92, 144.38, 134.77, 132.49, 131.60, 130.12, 129.96, 128.91, 127.98, 127.75, 126.90, 125.49, 115.89, 51.93, 49.14, 45.73, 42.47, 34.40, 16.87. FT-IR (cm^−1^): 3856, 3358, 2937, 1413, 1204, 974, 761. GC-MS (EI): tR = 17.362 min, MW = 346.4, *m*/*z*[M^+^] = 346.2.

*2-[(2E)-3-(4-Chlorophenyl)prop-2-enoyl]-6-methyl-1,2,3,6,7,11b-hexahydro-4H-pyrazino[2,1-a]isoquinolin-4-one* **25**: light yellow solid (0.17 g, 93.6%); m.p. 196–200 °C (dec.); Rf = 0,47 (DCM-ethyl acetate; 1:1.5); ^1^H NMR (400 MHz, CDCl_3_) δ 7.71 (d, *J* = 15.3 Hz, 1H), 7.47 (d, *J* = 8.3 Hz, 2H), 7.41–7.32 (m, 2H), 7.32–7.25 (m, 3H), 7.17 (d, *J* = 7.1 Hz, 1H), 6.81 (d, *J* = 15.3 Hz, 1H), 5.25 (s, 1H), 5.06 (d, *J* = 13.3 Hz, 1H), 4.81 (d, *J* = 9.6 Hz, 1H), 4.57 (d, *J* = 17.2 Hz, 1H), 4.25 (d, *J* = 17.2 Hz, 1H), 3.18–3.01 (m, 2H), 2.67–2.58 (m, 1H), 1.12 (d, *J* = 6.9 Hz, 3H). ^13^C NMR (101 MHz, CDCl_3_) δ 164.86, 163.53, 142.75, 135.82, 133.15, 132.37, 129.91, 129.06, 129.04, 127.69, 126.78, 125.35, 116.42, 51.42, 48.94, 45.56, 42.42, 34.20, 16.86. FT-IR (cm^−1^): 2927.24, 2848.43, 1735.50, 1650.53, 1635.68, 1411.26, 1318.00 1211.36, 1069.26, 759.41, 725.13. GC-MS (EI): tR = 18.536 min, MW = 380.8, *m*/*z*[M^+^] = 380.1.

*2-[(2E)-3-(4-Fluorophenyl)prop-2-enoyl]-6-methyl-1,2,3,6,7,11b-hexahydro-4H-pyrazino[2,1-a]isoquinolin-4-one* **26**; light yellow crystals (0.12 g, 70.5%); m.p. 175–179 °C; Rf = 0,25 (n-hexane-ethyl acetate, 1:1); ^1^H NMR (400 MHz, CDCl_3_) δ 7.71 (d, *J* = 15.4 Hz, 1H), 7.56–7.47 (m, 2H), 7.39–7.25 (m, 2H), 7.25 (dd, *J* = 7.6, 3.8 Hz, 1H), 7.16 (d, *J* = 7.2 Hz, 1H), 7.12–7.00 (m, 2H), 6.74 (d, *J* = 15.4 Hz, 1H), 5.30–5.21 (m, 1H), 5.06 (d, *J* = 13.3 Hz, 1H), 4.80 (d, *J* = 9.9 Hz, 1H), 4.56 (d, *J* = 17.3 Hz, 1H), 4.24 (d, *J* = 17.4 Hz, 1H), 3.13 (dd, *J* = 16.0, 5.7 Hz, 1H), 3.06 (s, 1H), 2.62 (dd, *J* = 16.1, 2.2 Hz, 1H), 1.83 (s, 1H), 1.30–1.17 (m, 2H), 1.11 (d, *J* = 6.9 Hz, 3H). ^13^C NMR (101 MHz, CDCl_3_) δ 163.84, 163.77(*J*_C-F_ = 250 Hz), 143.05, 130.01, 129.81 (*J*_C-F_ = 8.08 Hz), 127.80, 127.80, 126.88, 125.45, 116.13, 115.92, 115.66, 115.16, 51.54, 45.67, 42.51, 34.32, 29.65, 16.95. FT-IR (cm^−1^): 3749.75, 2904.07, 1746.49, 1634.90, 1598.93, 1442.50, 1408.74, 1316.60, 1177.97, 1014.94, 828.27. GC-MS (EI): tR = 17.237 min, MW = 364.4, *m*/*z*[M^+^] = 362.2.

*2-[(2E)-3-(4-Methoxy-phenyl)prop-2-enoyl]-6-methyl-1,2,3,6,7,11b-hexahydro-pyrido[2,1-a]isoquinolin-4-one* **27**; yellow crystals (0.14 g, 75.6%), m.p. 157–162 °C (dec.);Rf = 0.29 (diethyl ether); ^1^H NMR (400 MHz, CDCl_3_) δ 7.71 (d, *J* = 15.3 Hz, 1H), 7.52–7.43 (m, 2H), 7.35–7.20 (m, 3H), 7.14 (d, *J* = 6.9 Hz, 1H), 6.93–6.83 (m, 2H), 6.68 (d, *J* = 15.2 Hz, 1H), 5.24 (t, *J* = 7.5 Hz, 1H), 5.05 (s, 1H), 4.80 (s, 1H), 4.57 (d, *J* = 17.3 Hz, 1H), 4.22 (d, *J* = 17.4 Hz, 1H), 3.81 (s, 3H), 3.11 (dd, *J* = 16.1, 5.6 Hz, 1H), 3.03 (s, 1H), 2.60 (dd, *J* = 16.0, 2.2 Hz, 1H), 1.09 (d, *J* = 7.0 Hz, 3H). ^13^C NMR (101 MHz, CDCl_3_) δ 165.51, 161.18, 143.97, 132.46, 131.60, 29.93, 129.57, 127.69, 127.42, 126.80, 125.43, 114.26, 113.24, 55.29, 51.52, 49.00, 45.55, 42.39, 34.26, 16.88. FT-IR (cm^−1^): 3744.25, 2926.07, 2920.31, 1739.25, 1642.35, 1597.98, 1515.36, 1411.74, 1232.09, 1172.57, 1061.37, 826.26, 710.07, 769.16. GC-MS (EI): tR =19.040 min, MW = 376.4, *m*/*z*[M^+^] = 376.0.

*2-[(2E)-3-(3-Hydroxyphenyl)prop-2-enoyl]-6-methyl-1,2,3,6,7,11b-hexahydro-4H-pyrazino[2,1-a]isoquinolin-4-one* **28**; yellow crystals (0.16 g, 94.1%); m.p. 134–139 °C; Rf = 0.18 (diethyl ether); ^1^H NMR (400 MHz, CDCl_3_) δ 7.82–7.67 (m, 1H), 7.43–7.13 (m, 5H), 7.13–7.00 (m, 2H), 6.99–6.87 (m, 1H), 6.87–6.72 (m, 1H), 5.23 (d, *J* = 8.1 Hz, 1H), 5.03 (d, *J* = 13.2 Hz, 1H), 4.80 (s, 1H), 4.63–4.52 (m, 1H), 4.26 (dd, *J* = 17.5, 9.9 Hz, 1H), 3.17–2.99 (m, 2H), 2.66–2.57 (m, 1H), 1.10 (d, *J* = 6.8 Hz, 3H). ^13^C NMR (101 MHz, CDCl_3_) δ 165.66, 164.26, 157.10, 144.94, 135.85, 131.25, 130.07, 129.99, 127.81, 126.94, 125.51, 120.09, 117.80, 116.76, 115.55, 114.68, 51.57, 48.85, 45.77, 42.79, 34.22, 16.84. FT-IR (cm^−1^): 3200.24, 3189.30, 2932., 29.24, 1591.04, 1435.40, 1290.00, 1227.63, 1065.04, 970.86, 785.88, 755.95. GC-MS (EI): tR = 18.655 min, MW = 362.4, *m*/*z*[M^+^] = 362.2.

*2-[(2E)-3-(3-Nitrophenyl)prop-2-enoyl]- 6-methyl-1,2,3,6,7,11b-hexahydro-4H-pyrazino[2,1-a]isoquinolin-4-one* **29**; pale yellow solid (0.16 g, 87.1%); m.p. 160–164 °C (dec.); Rf = 0.35 (DCM-ethyl acetate, 1:1.5); ^1^H NMR (400 MHz, CDCl_3_) δ 8.40 (s, 1H), 8.23 (dd, *J* = 8.2, 2.3 Hz, 1H), 7.86–7.75 (m, 2H), 7.59 (t, *J* = 8.0 Hz, 1H), 7.39–7.25 (m, 3H), 7.18 (d, *J* = 7.3 Hz, 1H), 7.02–6.93 (m, 1H), 5.27 (dd, *J* = 9.7, 3.8 Hz, 1H), 5.07 (d, *J* = 13.5 Hz, 1H), 4.83 (d, *J* = 10.1 Hz, 1H), 4.59 (d, *J* = 16.9 Hz, 1H), 4.29 (d, *J* = 17.3 Hz, 1H), 3.13 (td, *J* = 19.5, 17.9, 8.7 Hz, 2H), 2.64 (d, *J* = 16.1 Hz, 1H), 1.13 (d, *J* = 7.1 Hz, 3H). ^13^C NMR (101 MHz, CDCl_3_) δ 164.36, 148.69, 141.49, 136.49, 133.65, 131.46, 129.99, 127.85, 126.94, 125.45, 124.36, 122.21, 119.09, 51.49, 49.09, 45.80, 42.61, 34.30, 16.96. FT-IR (cm^−1^): 1661.01, 1653.31, 1605.23, 1527.47, 1413.06, 1350.52, 1223.03, 975.04, 773.67, 737.99. GC-MS (EI): tR = 20.177 min, MW = 391.4, *m*/*z*[M^+^] = 391.1.

*2-[(2E)-3-(2-Chlorophenyl)prop-2-enoyl]-6-methyl-1,2,3,6,7,11b-hexahydro-4H-pyrazino[2,1-a]isoquinolin-4-one* **30**; yellow crystals (0.36 g, 51.0%); Rf = 0.32 (n-hexane-ethyl acetate, 1:1); ^1^H NMR (400 MHz, CDCl_3_) δ 8.11 (d, *J* = 15.2 Hz, 1H), 7.65–7.56 (m, 1H), 7.48–7.38 (m, 1H), 7.38–7.23 (m, 4H), 7.17 (s, 2H), 6.82 (d, *J* = 15.3 Hz, 1H), 5.25 (s, 1H), 5.08 (d, *J* = 7.6 Hz, 1H), 4.82 (s, 1H), 4.55 (d, *J* = 17.0 Hz, 1H), 4.24 (d, *J* = 17.2 Hz, 1H), 3.13 (dd, *J* = 16.1, 5.6 Hz, 1H), 3.06 (s, 1H), 2.66–2.58 (m, 1H), 1.12 (d, *J* = 7.0 Hz, 3H). ^13^C NMR (101 MHz, CDCl_3_) δ 164.90, 163.84, 140.25, 134.88, 133.13, 132.37, 131.48, 130.80, 130.24, 130.03, 127.77, 127.73, 127.05, 126.92, 125.46, 118.92, 51.55, 49.14, 45.72, 42.55, 34.33, 16.94. FT-IR (cm^−1^): 2919.38, 1642.39, 1412.65, 1318.97, 1205.08, 1037.32, 972.58, 756.64. GC-MS (EI): tR = 18.193 min, MW = 380.8, *m*/*z*[M^+^] = 380.1.

*2-[(2E)-3-(2-Bromo-4-fluorophenyl)prop-2-enoyl]-6-methyl-1,2,3,6,7,11b-hexahydro-4H-pyrazino[2,1-a]isoquinolin-4-one* **31**; colorless crystals (0.16 g, 75.0%); m.p. 182–186 °C; Rf = 0.23 (n-hexane-ethyl acetate, 1:1); ^1^H NMR (400 MHz, CDCl_3_) δ 7.74 (d, *J* = 6.4 Hz, 1H), 7.64 (d, *J* = 14.6 Hz, 1H), 7.44 (t, *J* = 6.3 Hz, 1H), 7.36–7.28 (m, 1H), 7.28 (s, 1H), 7.26 (d, *J* = 3.3 Hz, 1H), 7.15 (dt, *J* = 16.5, 10.0 Hz, 2H), 6.77 (d, *J* = 15.2 Hz, 1H), 5.28–5.20 (m, 1H), 5.05 (d, *J* = 13.1 Hz, 1H), 4.80 (d, *J* = 10.9 Hz, 1H), 4.57 (d, *J* = 16.5 Hz, 1H), 4.26 (d, *J* = 16.3 Hz, 1H), 3.11 (dt, *J* = 19.8, 9.7 Hz, 2H), 2.62 (d, *J* = 15.9 Hz, 1H), 1.11 (d, *J* = 6.8 Hz, 3H). ^13^C NMR (101 MHz, CDCl_3_) δ 163.67, 161.63 (*J*_C-F_ = 202Hz), 161.14, 141.58, 132.74, 132.48, 131.49, 130.01, 128.71, 127.81, 126.89, 125.44, 117.02, 116.94 (*J*_C-F_ = 22.22 Hz, 109.97, 109.81 (*J*_C-F_ = 22,22 Hz), 51.50, 49.07, 45.71, 42.53, 34.30, 16.95. FT-IR (cm^−1^): 3748.28, 2972.49, 2927, 1667.50, 1646.87, 1603.11, 1449.33, 1411.68, 1248.82, 1002.85, 819.33, 758.47. GC-MS (EI): tR = 19.047 min, MW = 443.3, *m*/*z*[M-Br81^+^] = 444.1.

*2-[(2E)-3-(4-Isopropyl-phenyl)-acryloyl]-6-methyl-1,2,3,6,7,11b-hexahydro-pyrazino[2,1-a]isoquinolin-4-one* **32**; white powder (0.16 g, 81.2%) m.p. 120–124 °C; Rf = 0.36 (n-hexane-ethyl acetate, 1:1); ^1^H NMR (400 MHz, CDCl_3_) δ 7.73 (d, *J* = 15.4 Hz, 1H), 7.50–7.37 (m, 2H), 7.35–7.24 (m, 1H), 7.23 (dd, *J* = 10.6, 4.2 Hz, 4H), 7.14 (d, *J* = 7.1 Hz, 1H), 6.79 (d, *J* = 15.4 Hz, 1H), 5.24 (q, *J* = 4.6 Hz, 1H), 5.06 (d, *J* = 13.3 Hz, 1H), 4.80 (s, 1H), 4.58 (d, *J* = 17.2 Hz, 1H), 4.23 (d, *J* = 17.3 Hz, 1H), 3.67 (q, *J* = 7.0 Hz, 1H), 3.11 (dd, *J* = 16.0, 5.6 Hz, 1H), 3.03 (s, 1H), 2.90 (p, *J* = 6.9 Hz, 1H), 2.64–2.53 (m, 1H), 1.27–1.14 (m, 7H), 1.09 (dd, *J* = 7.0, 2.0 Hz, 3H). ^13^C NMR (101 MHz, CDCl_3_) δ 165.47, 164.07, 151.36, 144.37, 132.39, 131.63, 129.97, 128.09, 127.74, 126.97, 126.85, 126.70, 126.34, 125.47, 51.56, 49.02, 45.63, 42.48, 34.28, 34.04, 23.70, 16.91. GC-MS (EI): tR = 19.105 min, MW = 388.2, *m*/*z*[M^+^] = 388.2.

*2-[(2E)-3-(3-Benzo[1,3]dioxol-5-yl-acryloyl)-6-methyl-1,2,3,6,7,11b-hexahydro-pyrazino[2,1-a]isoquinolin-4-one* **33**; white powder (0.14 g, 76.0%); m.p. 75–79 °C; Rf = 0.25 (n-hexane-ethyl acetate, 1:1); ^1^H NMR (400 MHz, CDCl_3_) δ 7.56 (d, *J* = 15.3 Hz, 1H), 7.26 (t, *J* = 6.1 Hz, 4H), 7.15 (d, *J* = 7.1 Hz, 1H), 7.05–6.93 (m, 1H), 6.79 (dd, *J* = 9.6, 7.9 Hz, 1H), 6.61 (d, *J* = 15.4 Hz, 1H), 6.01–5.94 (m, 2H), 5.25 (t, *J* = 6.3 Hz, 1H), 4.22 (d, *J* = 17.3 Hz, 2H), 3.89 (d, *J* = 5.2 Hz, 1H), 3.34 (s, 1H), 3.04 (s, 1H), 2.61 (dd, *J* = 16.2, 2.2 Hz, 1H), 2.02 (s, 1H), 1.41 (s, 1H), 1.10 (d, *J* = 6.9 Hz, 3H). ^13^C NMR (101 MHz, CDCl_3_) δ 165.39, 164.10, 149.45, 148.32, 144.09, 136.92, 134.78, 129.17, 128.35, 126.87, 125.48, 124.26, 123.77, 113.75, 108.57, 106.43, 101.38, 51.57, 49.07, 45.63, 42.45, 34.32, 16.94. FT-IR (cm^−1^): 3748.03, 2920.18, 2850.43, 1734.83, 1595.54, 1490.12, 1446.75, 14.30.13, 1415.22, 1125.91, 1033.51, 806.93. GC-MS (EI): tR = 20.043 min, MW = 390.4, *m*/*z*[M^+^] = 390.2.

*6-Ethyl-2-[(2E)-3-phenylprop-2-enoyl]-1,2,3,6,7,11b-hexahydro-4H-pyrazino[2,1-a]isoquinolin-4-one* **34**; yellow crystals (0.14 g, 85.0%); Rf = 0.38 (n-hexane-ethyl acetate, 1:1); ^1^H NMR (400 MHz, CDCl_3_) δ 7.76 (d, *J* = 15.4 Hz, 1H), 7.57–7.49 (m, 2H), 7.43–7.33 (m, 3H), 7.31 (s, 2H), 7.30–7.21 (m, 2H), 7.14 (d, *J* = 6.8 Hz, 1H), 6.83 (d, *J* = 15.4 Hz, 1H), 5.12 (d, *J* = 12.7 Hz, 1H), 5.03 (d, *J* = 7.2 Hz, 1H), 4.76 (s, 1H), 4.62 (d, *J* = 17.2 Hz, 1H), 4.25 (d, *J* = 17.2 Hz, 1H), 4.09 (qd, *J* = 7.2, 0.8 Hz, 1H), 3.13–2.96 (m, 2H), 2.71 (d, *J* = 16.2 Hz, 1H), 1.49–1.30 (m, 1H), 1.23 (td, *J* = 7.1, 0.8 Hz, 1H), 0.94–0.82 (m, 3H). ^13^C NMR (101 MHz, CDCl_3_) δ 171.09, 165.24, 144.36, 134.74, 130.10, 129.92, 128.88, 127.96, 127.69, 126.81, 125.50, 115.85, 60.34, 32.26, 23.73, 20.99, 14.15, 10.71. FT-IT (cm^−1^): 2961., 2925.15, 1643.23, 1412.92, 13.26,84, 1202.74, 1068.80, 976.12, 761.14. GC-MS (EI): tR = 17.559 min, MW = 360.4, *m*/*z*[M^+^] = 360.2.

*2-[(2E)-3-(4-Chloro-phenyl)-acryloyl]-6-ethyl-1,2,3,6,7,11b-hexahydro-pyrazino[2,1-a]isoquinolin-4-one* **35**; yellow crystals (0.13 g, 77.0%); m.p. 116–120 °C; Rf = 0.43 (n-hexane –ethyl acetate, 1:1); ^1^H NMR (400 MHz, CDCl_3_) δ 7.69 (d, *J* = 15.4 Hz, 1H), 7.52–7.43 (m, 2H), 7.38–7.21 (m, 5H), 7.14 (d, *J* = 7.2 Hz, 1H), 6.81 (d, *J* = 15.2 Hz, 1H), 5.11–4.99 (m, 2H), 5.02 (q, *J* = 7.1 Hz, 1H), 4.78–4.71 (m, 1H), 4.61 (d, *J* = 17.1 Hz, 1H), 4.24 (d, *J* = 17.1 Hz, 1H), 3.12–2.93 (m, 2H), 2.70 (d, *J* = 16.2 Hz, 1H), 1.45–1.34 (m, 2H), 0.87 (s, 1H), 0.84 (d, *J* = 7.4 Hz, 2H). ^13^C NMR (101 MHz, CDCl_3_) δ 164.85, 164.13, 142.78, 135.84, 133.15, 132.50, 131.57, 129.84, 129.07, 129.05, 127.61, 126.73, 125.39, 116.39, 51.78, 48.89, 48.15, 45.71, 32.16, 23.63, 10.64. FT-IT (cm^−1^): 3904., 3727, 2915, 1641.02, 1606, 1404.49, 1218, 1013.11, 821.18, 752.02. GC-MS (EI): tR = 18.919 min, MW = 394.8, *m*/*z*[M^+^] = 394.2.

*2-[(2E)-3-(4-Nitro-phenyl)-acryloyl]-6-ethyl-1,2,3,6,7,11b-hexahydro-pyrazino[2,1-a]isoquinolin-4-one*  **36**; colorless crystals (0.14 g, 77.0%); m.p. 195–198 °C; Rf = 0.33 (n-hexane-ethyl acetate, 1:1); ^1^H NMR (400 MHz, CDCl_3_) δ 8.26–8.14 (m, 9H), 7.76 (d, *J* = 15.4 Hz, 4H), 7.71–7.60 (m, 9H), 7.30 (d, *J* = 7.3 Hz, 6H), 7.26–7.11 (m, 14H), 6.97 (d, *J* = 15.4 Hz, 4H), 5.06 (s, 2H), 5.04–4.97 (m, 4H), 4.83 (s, 1H), 4.75 (d, *J* = 9.7 Hz, 3H), 4.61 (d, *J* = 17.0 Hz, 3H), 4.27 (d, *J* = 17.0 Hz, 4H), 3.12–2.95 (m, 8H), 2.71 (d, *J* = 16.1 Hz, 5H), 1.45 (dd, *J* = 14.2, 7.1 Hz, 4H), 1.40 (s, 3H), 1.22 (s, 3H), 0.85 (t, *J* = 7.4 Hz, 15H). ^13^C NMR (101 MHz, CDCl_3_) δ 164.19, 163.93, 148.31, 141.33, 140.86, 132.57, 131.49, 129.93, 128.52, 127.75, 126.82, 125.40, 124.13, 124.02, 120.25, 51.79, 48.96, 48.30, 45.90, 32.20, 23.69, 10.69. FT-IT (cm^−1^): 29,424, 2862, 2170, 2011.81, 1634.73, 1613.56, 1514.06, 1407.55, 1341.60, 1288.73, 1237.90, 1065.93, 840.58, 764.12, 743.80. GC-MS (EI): tR = min, MW = 405.4, *m*/*z*[M^+^] = 405.1.

*2-[(2E)-3-(4-Fluoro-phenyl)-acryloyl]- 6-ethyl-1,2,3,6,7,11b-hexahydro-pyrazino[2,1-a]isoquinolin-4-one*  **37**; colorless crystals (0.11 g, 65.2%), Rf = 0.39 (n-hexane-ethyl acetate, 1:1); ^1^H NMR (400 MHz, CDCl_3_) δ 7.72 (d, *J* = 15.4 Hz, 1H), 7.57–7.48 (m, 2H), 7.35–7.19 (m, 3H), 7.18–6.99 (m, 4H), 6.75 (d, *J* = 15.4 Hz, 1H), 5.07 (dd, *J* = 33.2, 10.3 Hz, 2H), 4.76 (d, *J* = 10.2 Hz, 1H), 4.61 (d, *J* = 17.2 Hz, 1H), 4.25 (d, *J* = 17.3 Hz, 1H), 3.08 (dd, *J* = 16.1, 5.6 Hz, 1H), 3.04–2.94 (m, 1H), 2.72 (d, *J* = 16.2 Hz, 1H), 1.41 (s, 1H), 1.23 (s, 1H), 0.86 (t, *J* = 7.4 Hz, 3H). ^13^C NMR (101 MHz, CDCl_3_) δ 166.25, 164.20, 163.79(*J*_C-F_ = 250Hz), 143.13, 132.65, 131.64, 131.00, 129.84 (*J*_C-F_ = 8.08 Hz), 127.73, 126.84, 125.51, 116.45 (*J*_C-F_ = 22 Hz), 115.56 (*J*_C-F_ = 3.3 Hz), 94.99. FT-IT (cm^−1^): 3748.42, 2927.68, 2861, 1643.24, 1599.62, 1414.70, 1325.46, 1224.84, 1158.78, 827.04, 758.09. GC-MS (EI): tR = 17.447 min, MW = 378.4, *m*/*z*[M^+^] = 378.2.

*2-[(2E)-3-(4-Methoxy-phenyl)-acryloyl]-6-ethyl-1,2,3,6,7,11b-hexahydro-pyrazino[2,1-a]isoquinolin-4-one* **38**; yellow crystals (0.04 g, 75.2%); m.p. 185–189 °C; Rf = 0.21 (n-hexane-ethyl acetate); ^1^H NMR (400 MHz, CDCl_3_) δ 7.72 (d, *J* = 15.3 Hz, 1H), 7.52–7.44 (m, 2H), 7.32 (s, 1H), 7.23 (s, 1H), 7.14 (d, *J* = 7.2 Hz, 1H), 6.93–6.83 (m, 2H), 6.73–6.65 (m, 1H), 5.12 (d, *J* = 13.7 Hz, 1H), 5.03 (q, *J* = 7.0 Hz, 1H), 4.74 (d, *J* = 9.9 Hz, 2H), 4.62 (d, *J* = 17.1 Hz, 1H), 4.23 (d, *J* = 17.4 Hz, 1H), 3.82 (s, 3H), 3.07 (dd, *J* = 16.2, 5.6 Hz, 1H), 2.96 (s, 1H), 2.70 (d, *J* = 16.2 Hz, 1H), 1.48–1.30 (m, 1H), 0.96–0.81 (m, 4H). ^13^C NMR (101 MHz, CDCl_3_) δ 165.56, 164.44, 161.21, 144.11, 132.55, 131.70, 129.89, 129.63, 127.63, 127.40, 126.78, 125.52, 114.28, 113.15, 55.33, 51.90, 48.96, 48.18, 45.69, 32.24, 23.67, 10.71. FT-IT (cm^−1^): 2967., 2929.95, 1637.42, 1597.15, 1413.28, 1413.25, 1253.52, 1237.26, 1173.90, 1029.37, 810.68. GC-MS (EI): tR = 19.373 min, MW = 390.4, *m*/*z*[M^+^] = 390.2.

*2-[(2E)-3-(3,4-Dimethoxy-phenyl)-acryloyl]-6-ethyl-1,2,3,6,7,11b-hexahydro-pyrazino[2,1-a]isoquinolin-4-one*  **39**; yellow solid (0.03 g, 88.8%); Rf = 0.31 (n-hexane-ethyl acetate, 1:1); ^1^H NMR (400 MHz, CDCl_3_) δ 7.72 (d, *J* = 15.2 Hz, 1H), 7.35–7.20 (m, 2H), 7.13 (t, *J* = 9.4 Hz, 2H), 7.05 (d, *J* = 1.7 Hz, 1H), 6.87 (d, *J* = 8.3 Hz, 1H), 6.68 (d, *J* = 15.3 Hz, 1H), 5.15 (d, *J* = 13.1 Hz, 1H), 5.04 (q, *J* = 7.2 Hz, 1H), 4.75 (s, 1H), 4.66 (d, *J* = 17.2 Hz, 1H), 4.26 (d, *J* = 17.4 Hz, 1H), 3.92 (d, *J* = 9.2 Hz, 6H), 3.59–3.42 (m, 1H), 3.09 (dd, *J* = 16.1, 5.5 Hz, 1H), 2.98 (s, 1H), 2.72 (d, *J* = 16.2 Hz, 1H), 2.03 (s, 1H), 1.48–1.29 (m, 1H), 0.88 (q, *J* = 7.6 Hz, 3H). ^13^C NMR (101 MHz, CDCl_3_) δ 165.45, 163.95, 159.19, 152.66, 150.99, 149.19, 144.43, 132.62, 130.01, 129.98, 129.28, 129.12, 127.76, 127.69, 126.83, 125.55, 122.75, 113.27, 111.02, 55.97, 51.97, 49,10, 48.17, 45.71, 32.29, 23.63, 10.75. GC-MS (EI): tR = 20.649 min, MW = 420.5, *m*/*z*[M^+^] = 420.2.

*2-[(2E)-3-(2,4-Dimethoxy-phenyl)-acryloyl]-6-ethyl-1,2,3,6,7,11b-hexahydro-pyrazino[2,1-a]isoquinolin-4-one*  **40**; yellowish solid (0.03 g, 85.0%); Rf = 0.41 (n-hexane-ethyl acetate, 1:1); ^1^H NMR (400 MHz, CDCl_3_) δ 7.92 (d, *J* = 15.4 Hz, 1H), 7.42 (d, *J* = 8.5 Hz, 1H), 7.35–7.23 (m, 1H), 7.27–7.19 (m, 1H), 7.14 (d, *J* = 7.4 Hz, 1H), 6.89 (d, *J* = 15.4 Hz, 1H), 6.54–6.43 (m, 2H), 5.15 (s, 1H), 5.04 (d, *J* = 7.2 Hz, 1H), 4.75 (d, *J* = 9.9 Hz, 1H), 4.63 (d, *J* = 17.7 Hz, 1H), 4.22 (d, *J* = 17.8 Hz, 1H), 3.91–3.68 (m, 6H), 3.09 (dd, *J* = 16.2, 5.6 Hz, 1H), 2.95 (s, 1H), 2.71 (d, *J* = 16.2 Hz, 1H), 1.48–1.31 (m, 1H), 0.95–0.70 (m, 4H). ^13^C NMR (101 MHz, CDCl_3_) δ 166.40, 164.65, 162.45, 159.90, 139.98, 131.32, 129.87, 127.64, 126.78, 125.64, 124.63, 120.08, 116.95, 114.11, 105.08, 98.53, 55.52, 55.46, 52.17, 48.25, 45.98, 32.31, 29.68, 23.71, 10.76. GC-MS (EI): tR = 21.055 min, MW = 420.5, *m*/*z*[M^+^] = 420.2.

## 5. Conclusions

Two PZQ analogues and seventeen molecular hybrids were successfully synthesized in a three-step procedure starting from the corresponding two 2-amino-1-phenylalkanes as precursors. The identity of the synthesized compounds was fully established by NMR spectroscopy, FTIR, and mass spectrometry, and their biological activity against *P. falciparum* and *S. mansoni* was evaluated. While none of the compounds were active against *P. falciparum*, the results identified four molecular hybrids with good activity against *S. manson*. Based on its activity, one particular molecular hybrid can be considered as a front runner, the optimization of which may generate a lead compound in the fight against schistosomiasis. An ortho substitution by a bromine, a chlorine atom substitution on the para position, and a substitution on the para position of cinnamic acid aromatic ring by a nitro group as well as by an isopropyl group, increases the activity of the molecule.

The strategy of molecular hybridization employed in this project, which involves coupling cinnamic acids with the PZQ tetrahydroisoquinoline scaffold, showed promising results in the development of novel compounds with potent activity.

This work also confirmed that substitution at the C6 position of the tetrahydroisoquinoline aromatic ring affects the activity of the molecular hybrid, which increases with the increase in the number of carbon atoms of the substituent, including the substitution on the para position on the cinnamic acid aromatic ring.

## Figures and Tables

**Table 1:** Synthesis of praziquantel analogues and molecular hybrids.

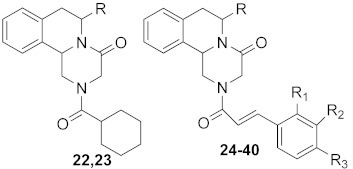					
Compound	R	R_1_	R_2_	R_3_	Yield (%)
**22**	Me	-	-	-	68.3
**23**	Et	-	-	-	64.2
**24**	Me	H	H	H	73.4
**25**	Me	H	H	Cl	93.6
**26**	Me	H	H	F	70.5
**27**	Me	H	H	Ome	75.6
**28**	Me	H	OH	H	94.1
**29**	Me	H	NO_2_	H	87.1
**30**	Me	Cl	H	H	51.0
**31**	Me	Br	H	F	75.0
**32**	Me	H	H	iPr	81.2
**33**	Me	H	-O-CH_2_-O-		76.0
**34**	Et	H	H	H	85.0
**35**	Et	H	H	Cl	77.0
**36**	Et	H	H	NO_2_	77.0
**37**	Et	H	H	F	65.2
**38**	Et	H	H	Ome	75.2
**39**	Et	H	Ome	Ome	88.8
**40**	Et	Ome	H	Ome	85.0

**Table 2:** Antischistosomal worm killing activity of praziquantel analogues and molecular hybrids.

	NTS	NTS	*S. mansoni* Adult	*S. mansoni* Adult	*S. mansoni* Adult	
Compound ID	Effect in % (Dead, 72 h) and SD Test Conc. 50 µM	Effect in % (Dead, 72 h) and SD Test Conc. 10 µM	Effect in % (Dead, 72 h) and SD Test Conc. 50 µM	Effect in % (Dead, 72 h) and SD Test Conc. 10 µM	Effect in % (Dead, 72 h) and SD Test Conc. 1 µM	IC_50_ Schisto Adult (µM) (72 h)
**22**	43.3 (3.3)	20.8 (4.2)	-	-	-	
**23**	75 (5)	37.5 (4.2)	-	-	-	
**24**	46.7 (6.7 < 9)	43.8 (2.1)	-	-	-	
**25**	66.7 (3.3)	62.5 (0)	-	-	-	
**26**	73.3 (6.7)	18.8 (2.1)	-	-	-	
**27**	55 (1.7)	35.4 (2.1)	-	-	-	
**28**	61.7 (1.7)	31.3 (2.1)	-	-	-	
**29**	68.3 (1.7)	29.2 (0)	-	-	-	
**30**	63.3 (3.3)	25 (0)	-	-	-	
**31**	100 (0)	35.4 (2.1)	81.5 (3.7)	34.5 (0)		12.7
**32**	100 (0)	50 (0)	100 (0)	78.2 (0)	38.9 (5.6)	1.9
**33**	66.7 (3.3)	35.4 (2.1)	-	-	-	
**34**	88.3 (1.7)	35.4 (2.1)	33.3 (0)	-	-	
**35**	100 (0)	64.6 (2.1)	96.3 (0)	30.9 (0)		13.8
**36**	95 (1.7)	33.3 (4.2)	48.2 (3.7)	-	-	
**37**	73.3 (3.3)	31.3 (2.1)	-	-	-	
**38**	45 (1.7)	37.5 (0)	-	-	-	
**39**	45 (3.3)	27.1 (2.1)	-	-	-	
**40**	85 (1.7)	29.2 (4.2)	77.8 (0)	34.5 (0)	-	14.7
**PZQ**	100 (0)	-	100 (0)	100 (0)	91.7 (6.5)	

NTS: newly transformed schistosomula.

## Data Availability

Data is shared in the paper and Supplementary Materials.

## Supplementary Materials

The following supporting information can be downloaded at: https://www.mdpi.com/article/10.3390/molecules28135184/s1, In-vitro anti-plasmodium activity, NMR spectra, HPLC-MS and IR spectra of compounds described in this study. Table S1. In vitro antiplasmodial activity evaluation of molecular hybrids and praziquantel analogues.
