# Supplementary material for: Design, Synthesis and Evaluation of Praziquantel Analogues and New Molecular Hybrids as Potential Antimalarial and Anti-Schistosomal Agents
Source: Molecules. 2023 Jul 3;28(13):5184. doi: 10.3390/molecules28135184 (PMC10343899; doi:10.3390/molecules28135184)
Supplement: Supplementary file 1 [file molecules-28-05184-s001.zip › molecules-2423567-supplementary.pdf]

Supplementary Information

Antiplasmodial evaluation: *In-vitro anti-plasmodium assay*. Compounds were tested using parasite lactate dehydrogenase assay as a marker for parasite survival. The respective stock solutions of CQ diphosphate and test compounds were prepared by dissolving 2 mg/mL in distilled water (for CQ) and 100% DMSO for test compounds. The solutions were then stored at −20 °C, with further dilutions prepared on the day of the experiment. The cultures were synchronized in the ring stage using 15 mL of 5% (w/v) d-sorbitol in water. Synchronous cultures of Pf NF54 (CQS) in the late trophozoite stage were prepared to 2% parasitemia and 2% hematocrit. Compounds were tested at starting concentrations of 10 000 ng/mL (1000 ng/mL for CQ), which were then serially diluted two-fold in complete medium to give 10 concentrations with a final volume of 200 µL in each well. Parasites were incubated in the presence of the compounds at 37 °C in a specialized atmosphere of 4% CO_2 and 3% O_2 in nitrogen for 48 h. Following incubation, 100 µL of MalStat reagent and 15 µL of resuspended culture were combined, followed by addition of 25 µL of nitro blue tetrazolium chloride (NBT). The plates were kept in the dark for about 10 min to fully develop, and absorbance was measured at 620 nm on a microplate reader. Raw data were exported to Microsoft Excel for dose-response analysis [1, 2].The as synthesized molecular hybrids and analogues were evaluated for in-vivo antiplasmodium activity against the chloroquine-sensitive strain (NF54) of P. falciparum, the IC_{50} recorded are displayed in the Table S1.

Table S1. In vitro antiplasmodial activity evaluation of molecular hybrids and praziquantel analogues.

Compounds	Antiplasmodial activity IC_{50} µM	SEM
**2**	>6	ND
**3**	>6	ND
**4**	>6	ND

**5**	>6	ND
**6**	>6	ND
**7**	>6	ND
**8**	>6	ND
**9**	>6	ND
**10**	>6	ND
**11**	>6	ND
**12**	>6	ND
**13**	>6	ND
**14**	>6	ND
**15**	>6	ND

**16**	>6	ND
**17**	>6	ND
**18**	>6	ND
**19**	>6	ND
**20**	>6	ND
**Artesunate**	>6	ND
**Chloroquine**	0.009	0.002

The concentrations were presented in µM/L; ND: Not determined, SEM: Standard error on the Mean.

References.

1. Mueller, R., et al., *Lerisetron Analogues with Antimalarial Properties: Synthesis, Structure–Activity Relationship Studies, and Biological Assessment.* ACS Omega, 2020. **5**(12): p. 6967-6982. https://doi.org/10.1021/acsomega.0c00327
2. Makler, M.T., et al., *Parasite Lactate Dehydrogenase as an Assay for Plasmodium falciparum Drug Sensitivity.* The American Journal of Tropical Medicine and Hygiene, 1993. **48**(6): p. 739-741. https://doi.org/10.4269/ajtmh.1993.48.739

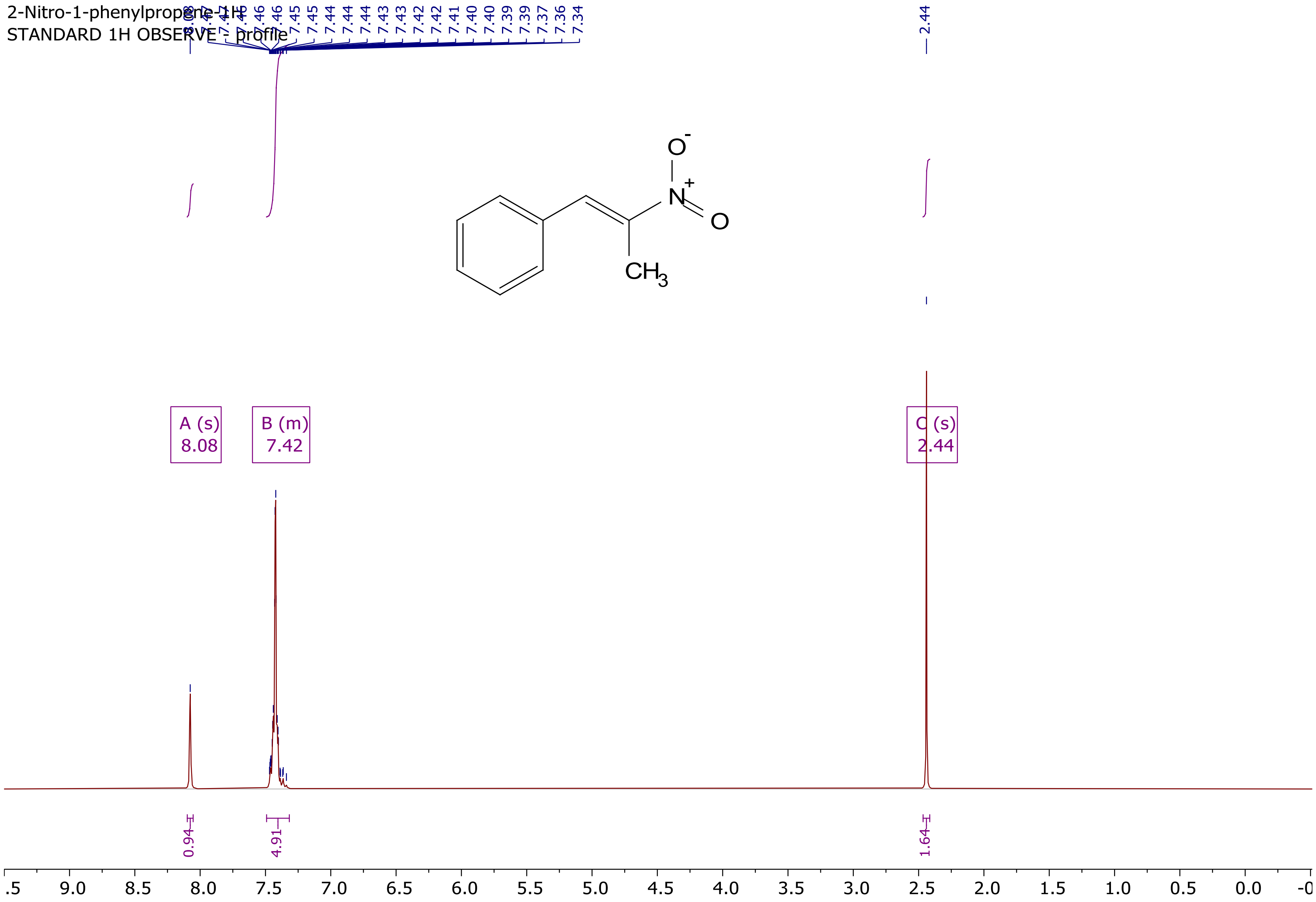

^{1}H-NMR spectrum of trans-2-nitro-1-phenylpropene **165a**

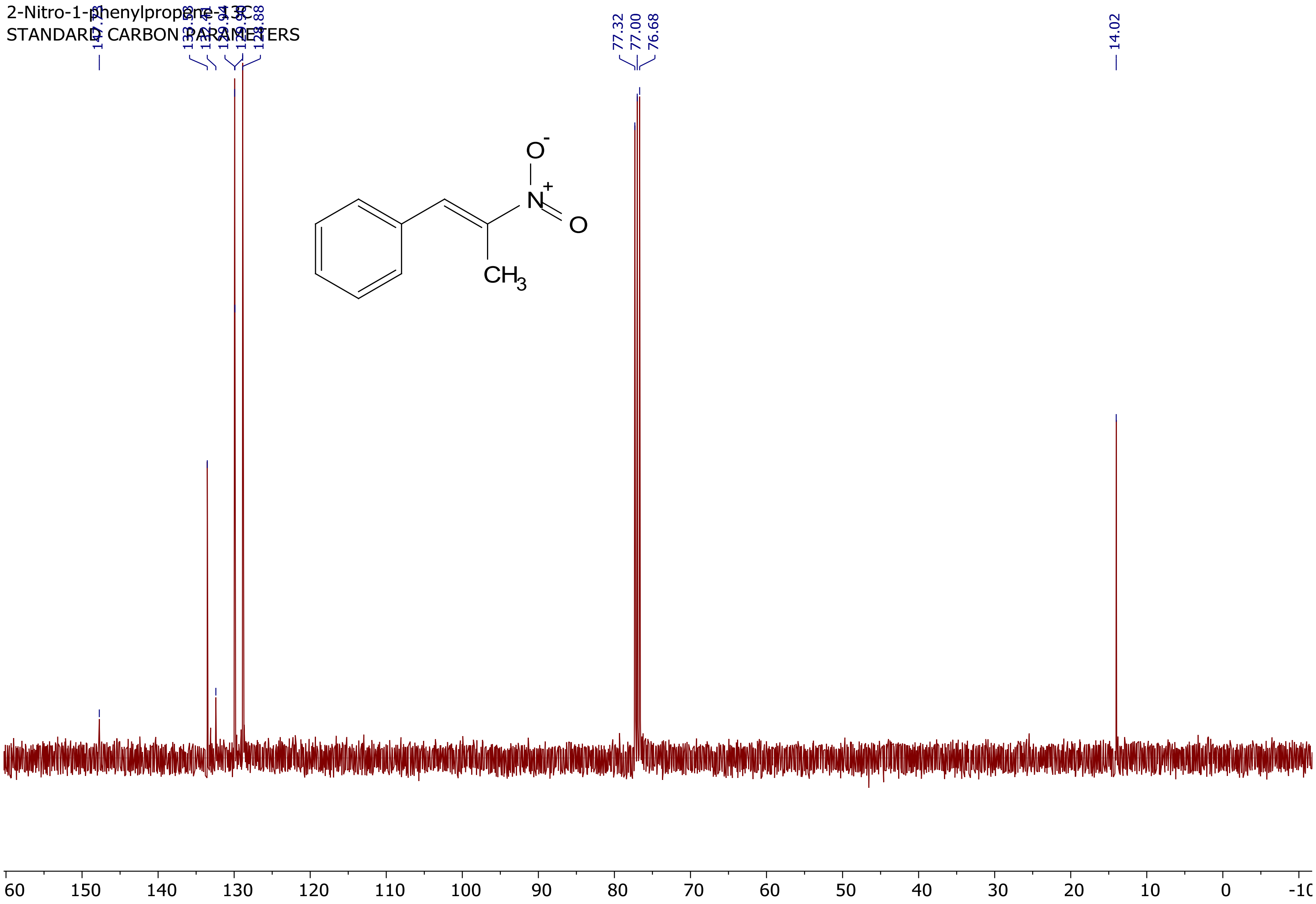

^{13}C-NMR spectrum of trans-2-nitro-1-phenylpropene <u>16</u>~~5~~**a**

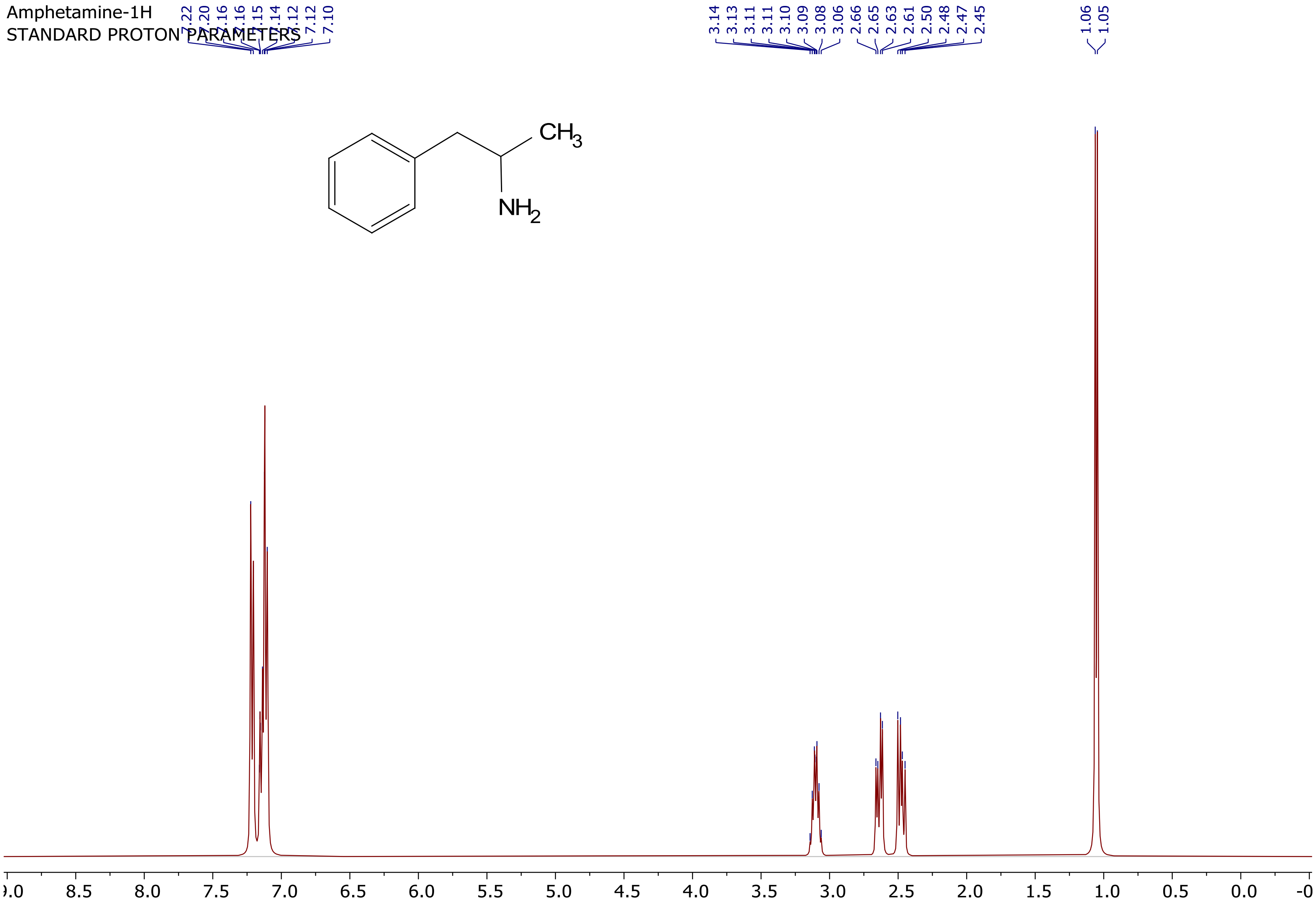

^{1}H-NMR spectrum of amphetamine **17~~6~~a**

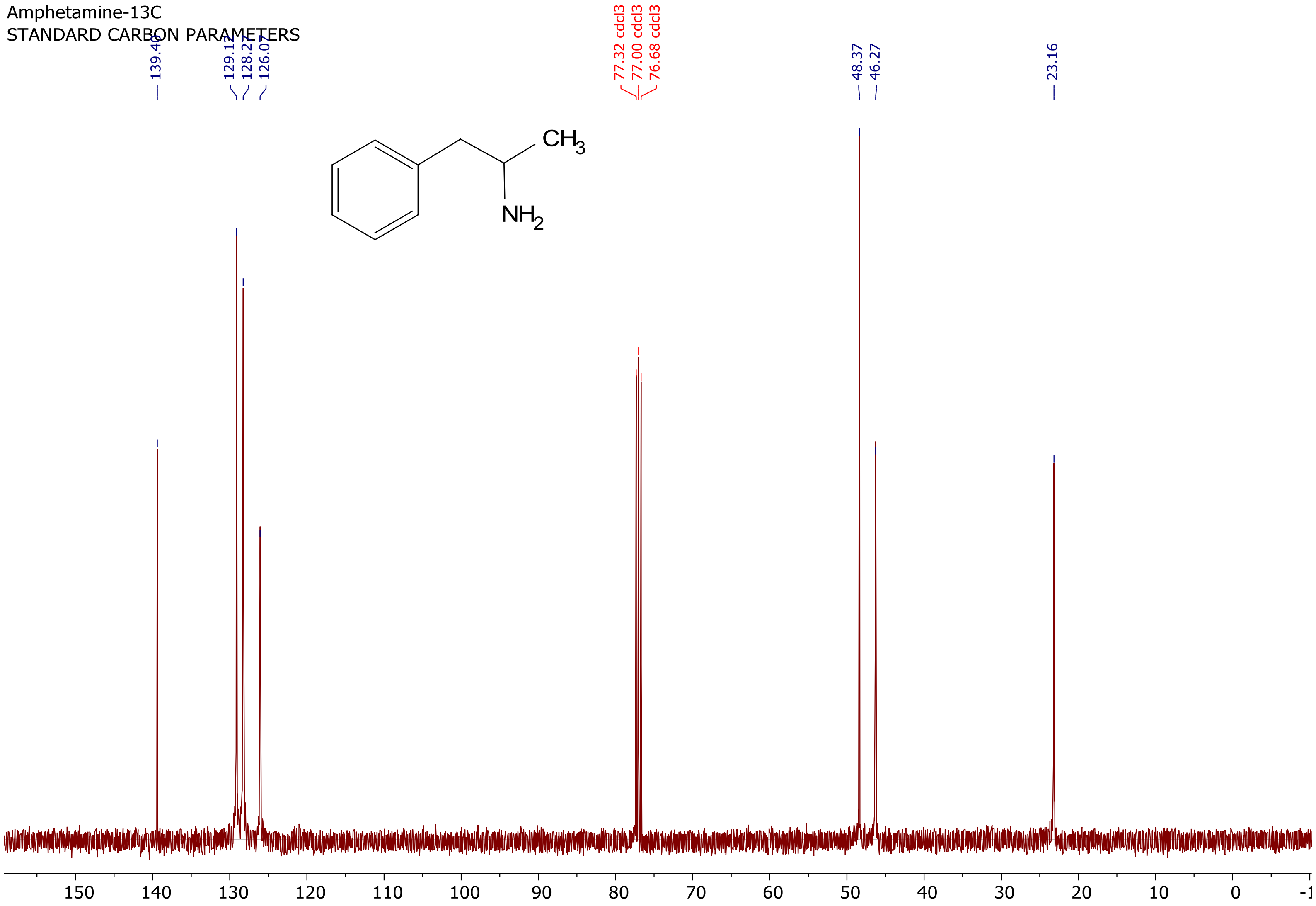

^{13}C-NMR spectrum of amphetamine **17b**

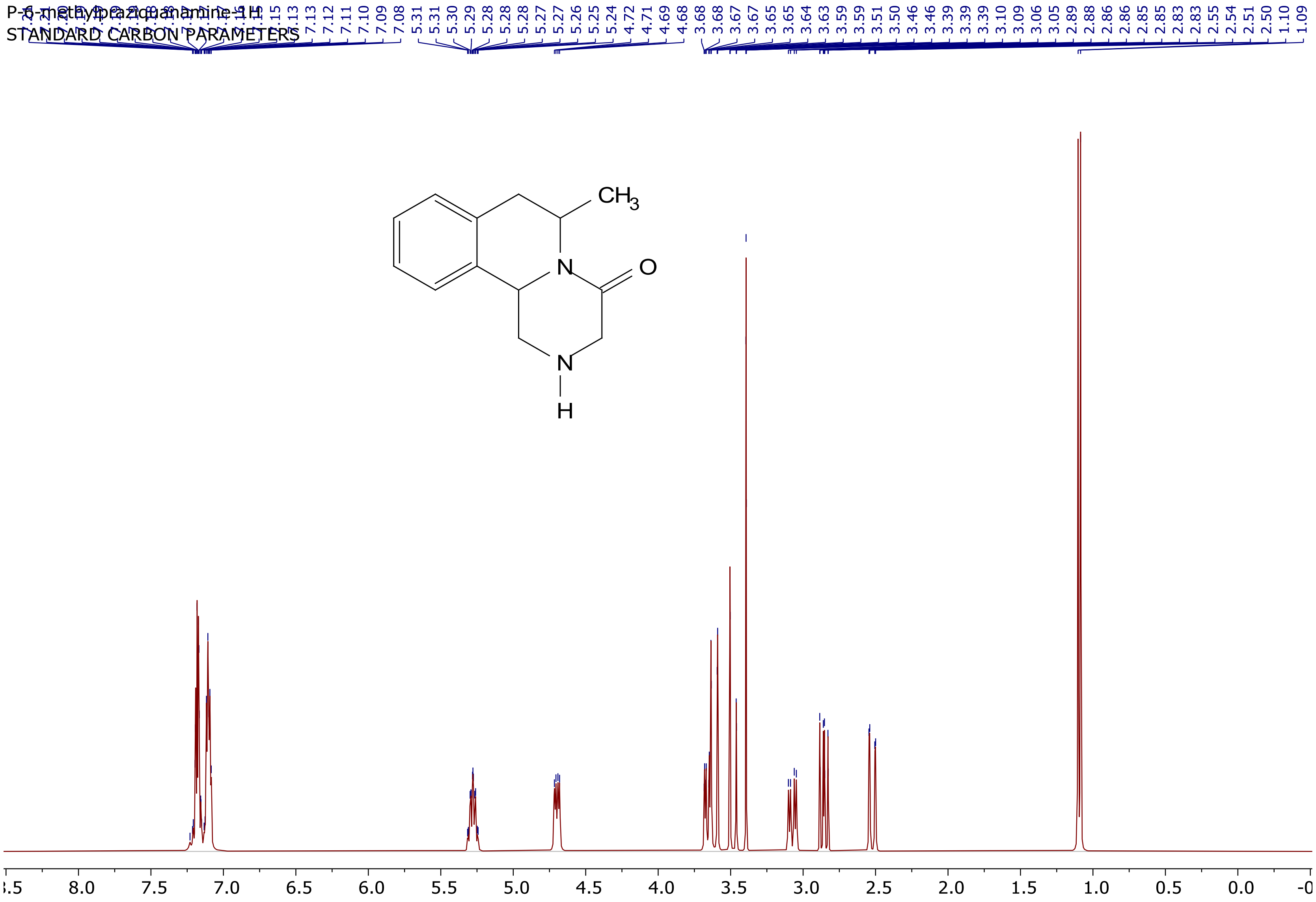

^{1}H-NMR spectrum of 6-methylpraziquananime **21a**

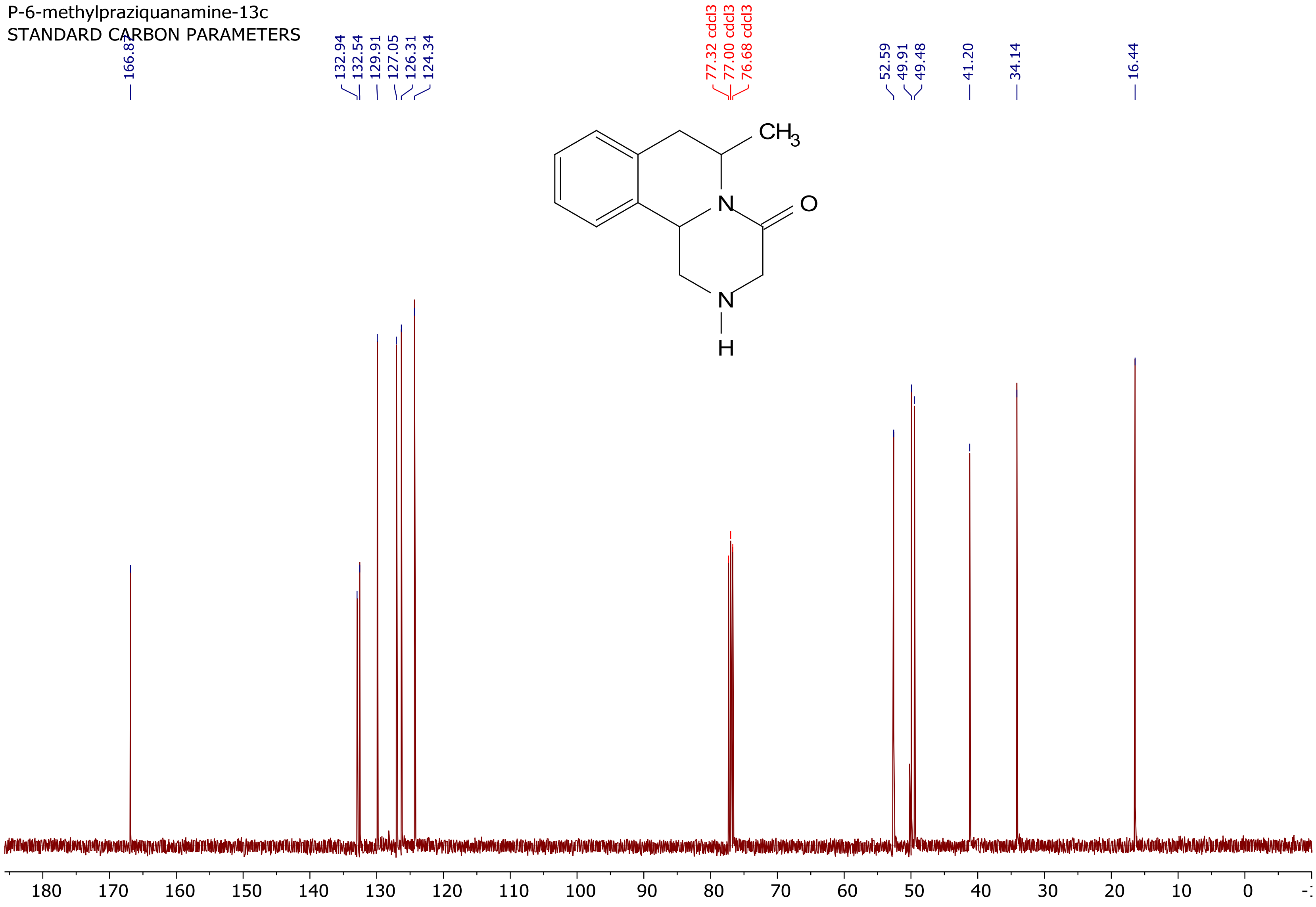

^{13}C-NMR spectrum of 6-methylpraziquananime **21a**

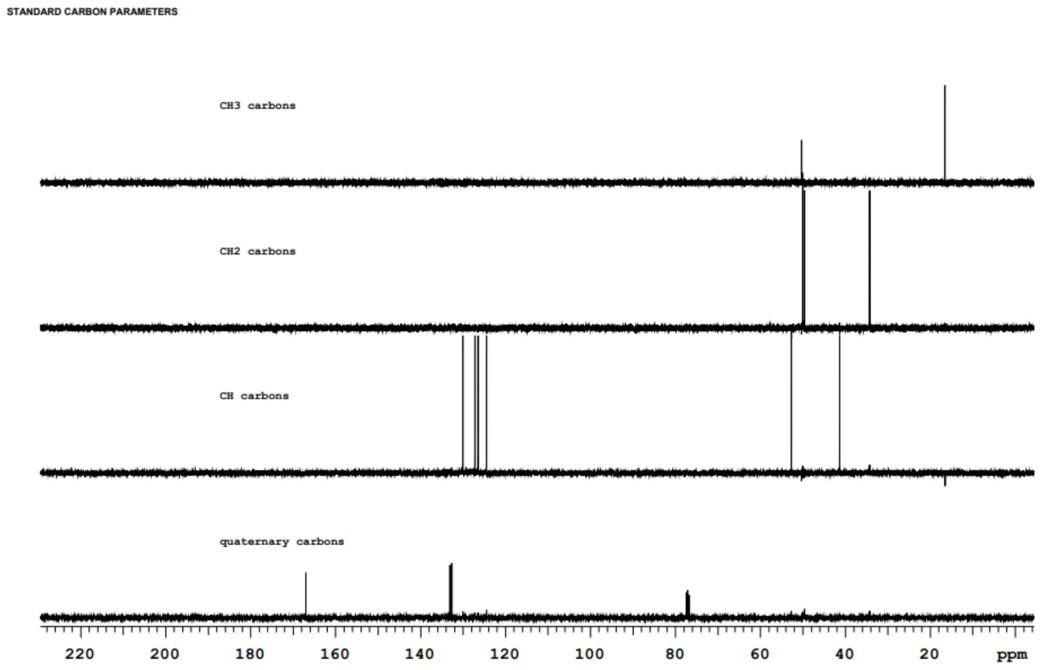

DEPT spectrum of 6-methylpraziquananime **21a**

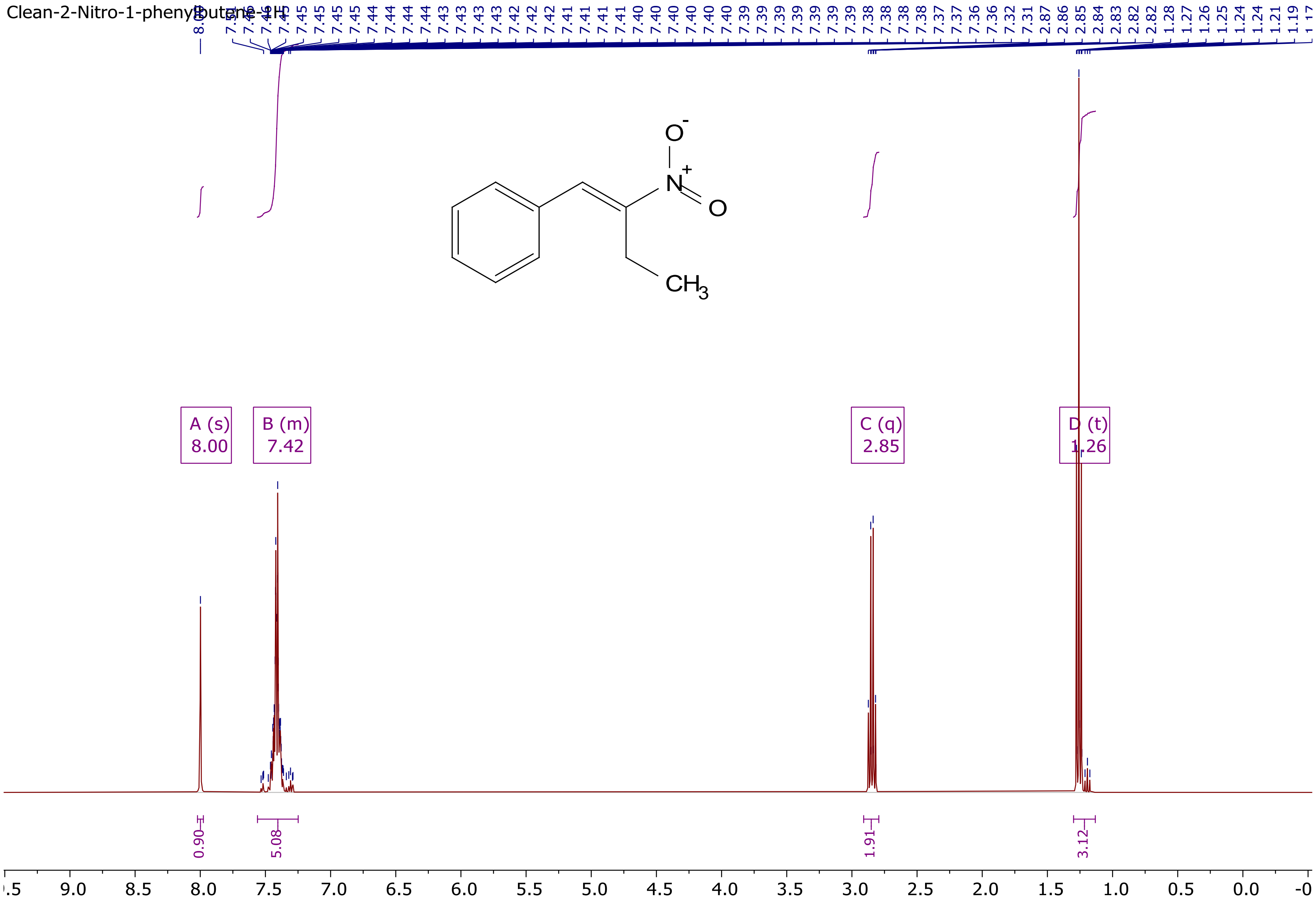

^{1}H-NMR spectrum of trans-2-nitro-1-phenylbutene **16b**

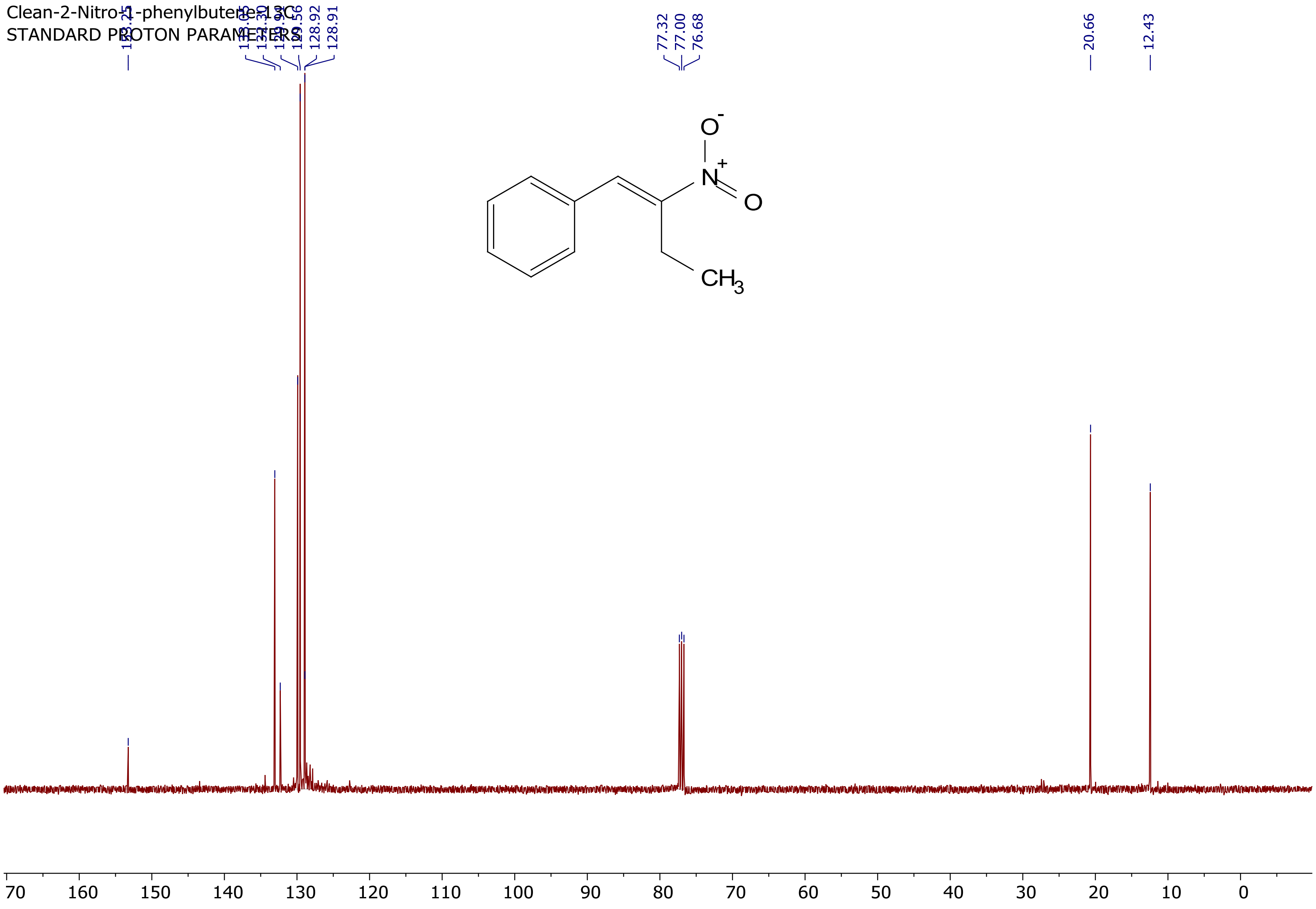

^{13}C-NMR spectrum of trans-2-nitro-1-phenylbutene **16b**

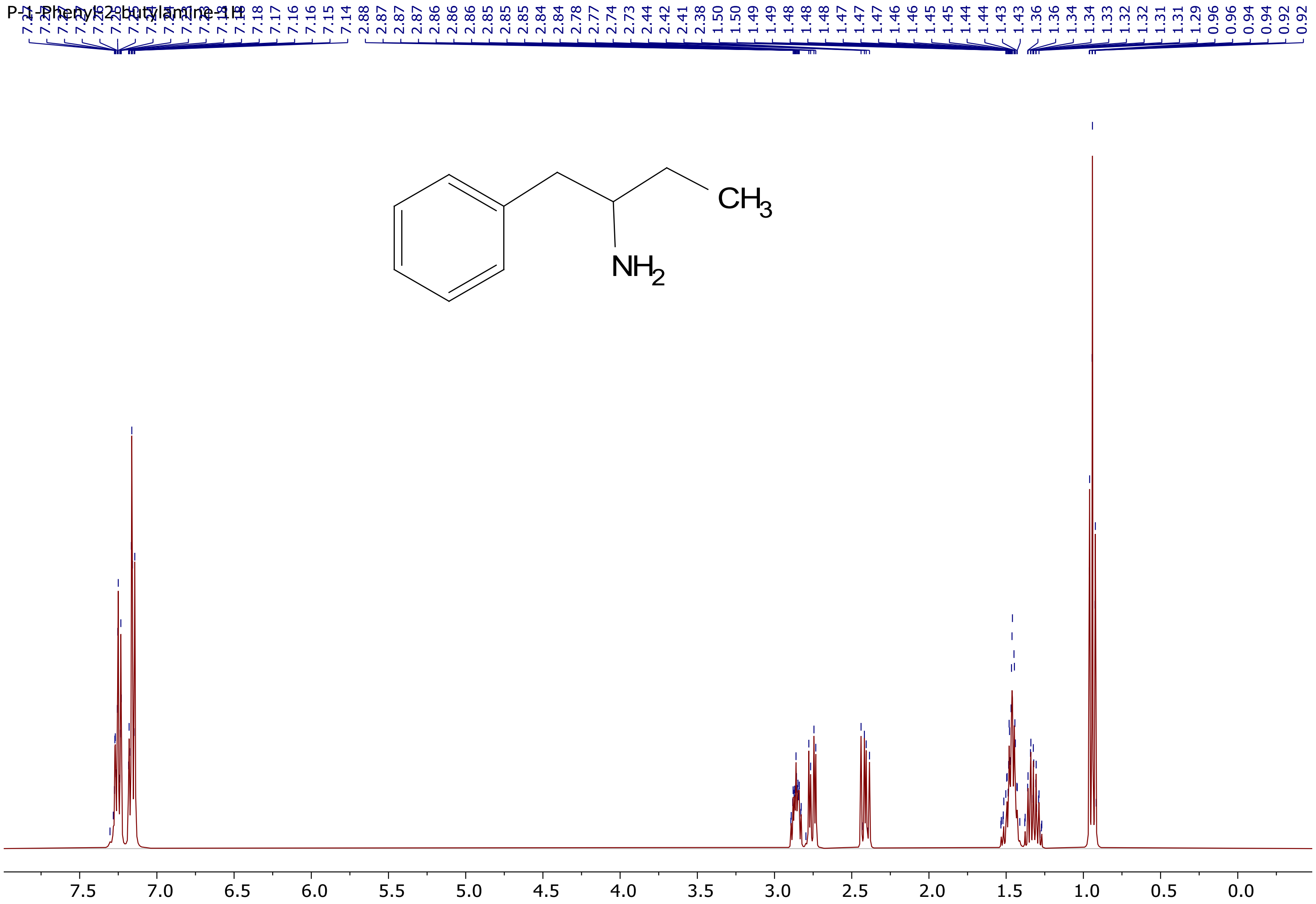

^{1}H-NMR spectrum of 1-phenyl-2-butylamine **17b**

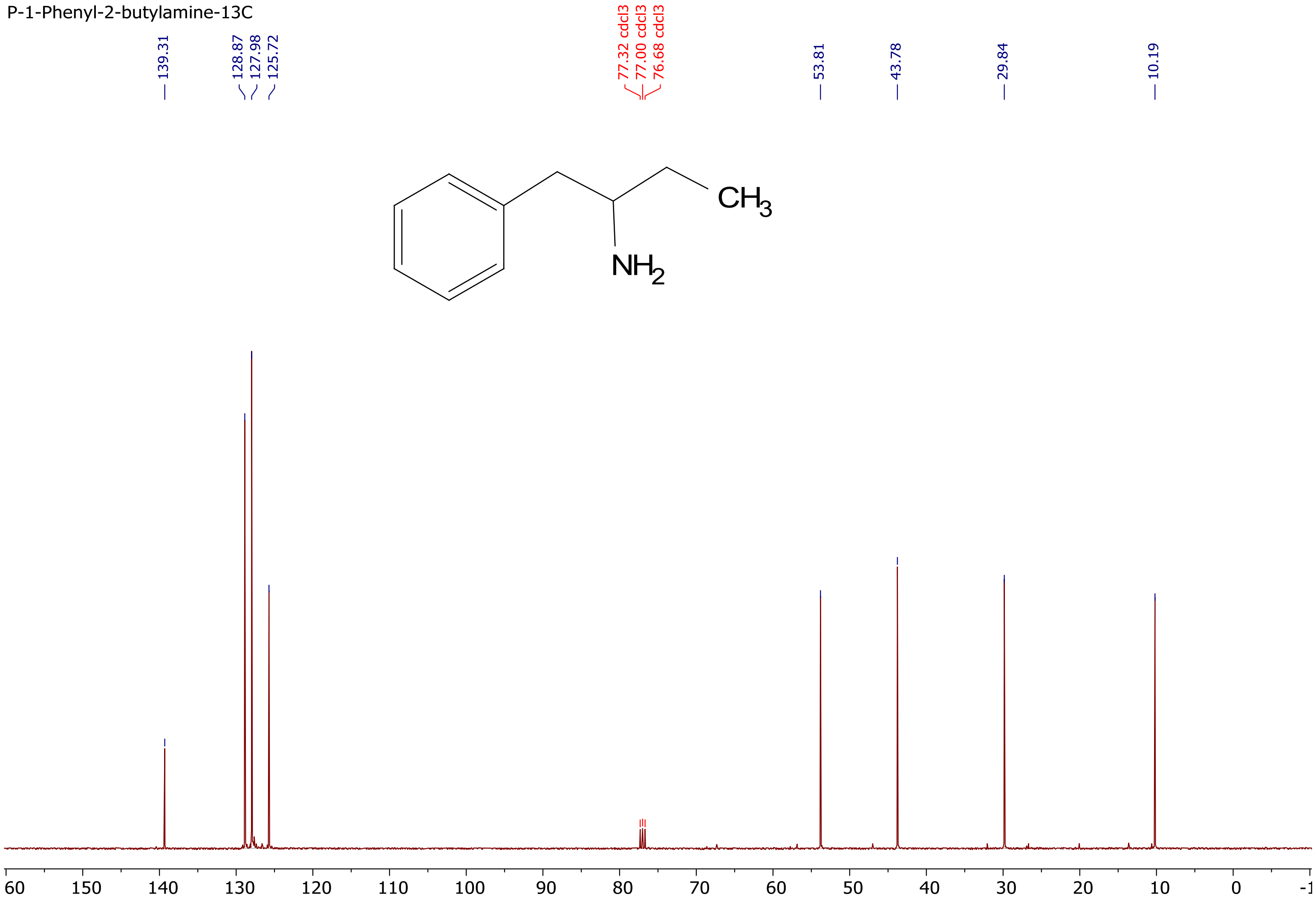

^{13}C-NMR spectrum of 1-phenyl-2-butylamine **17b**

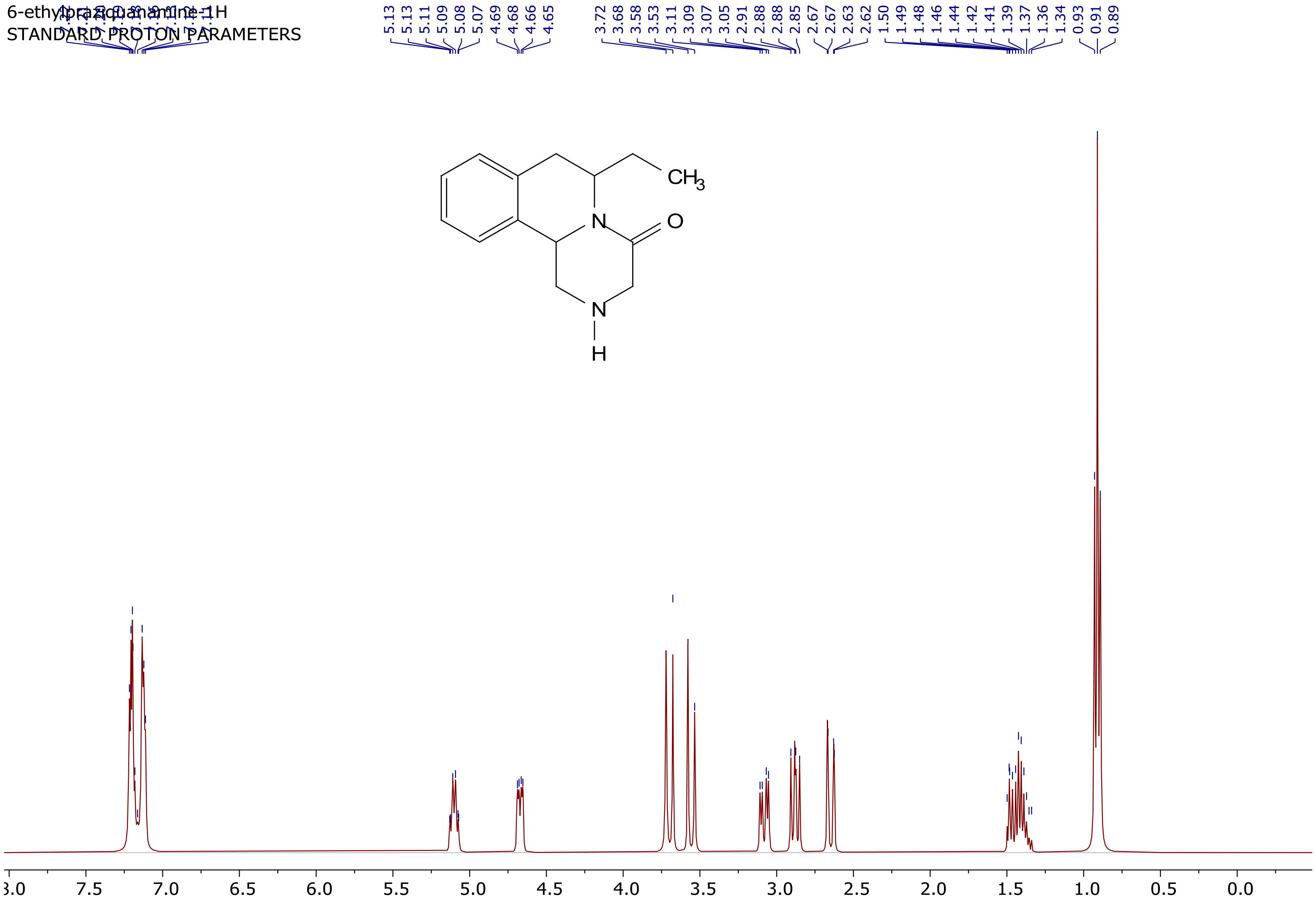

^{1}H-NMR spectrum of 6-ethylpraziquanamine **21b**

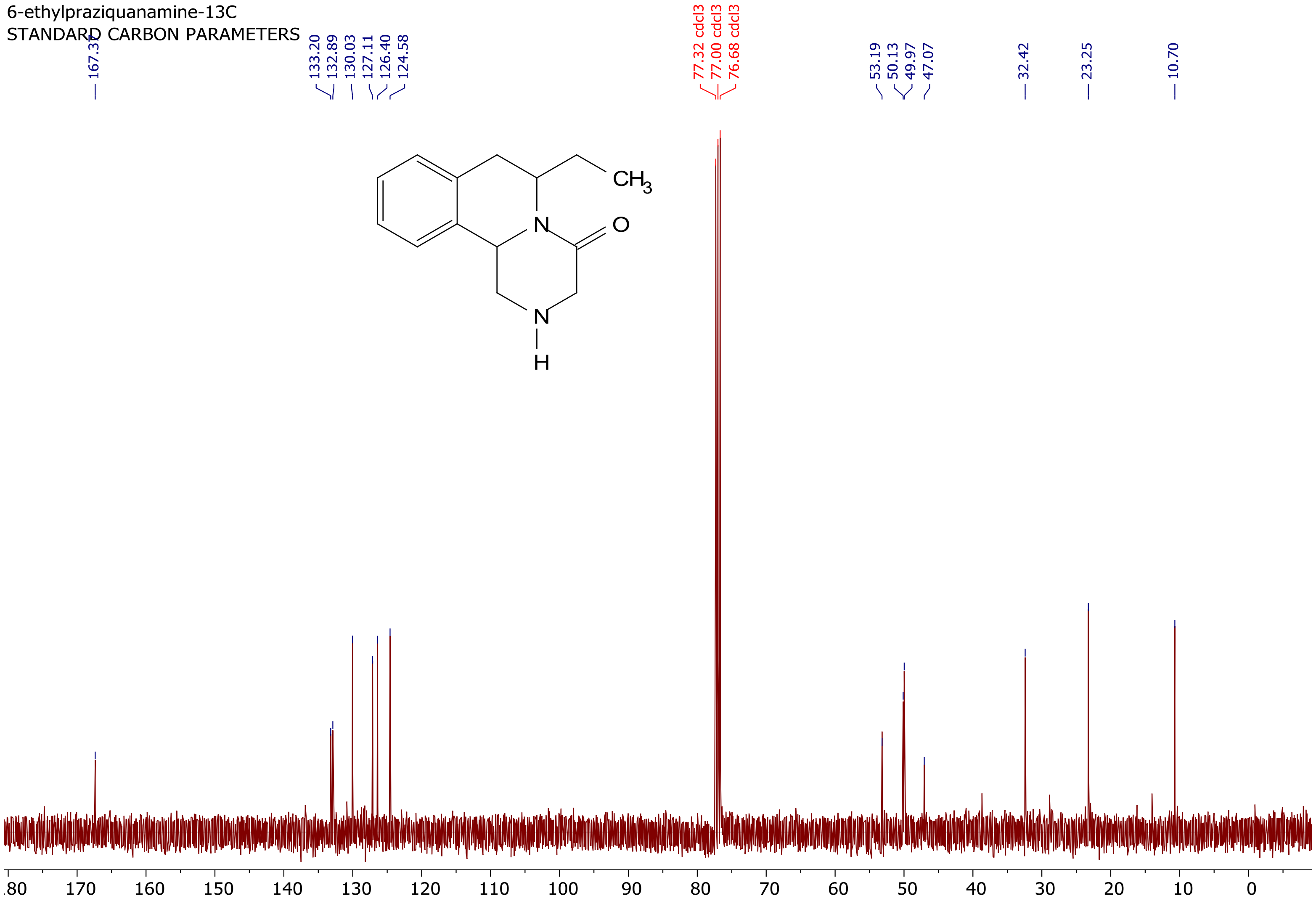

^{13}C-NMR spectrum of 6-ethylpraziquanamine **21b**

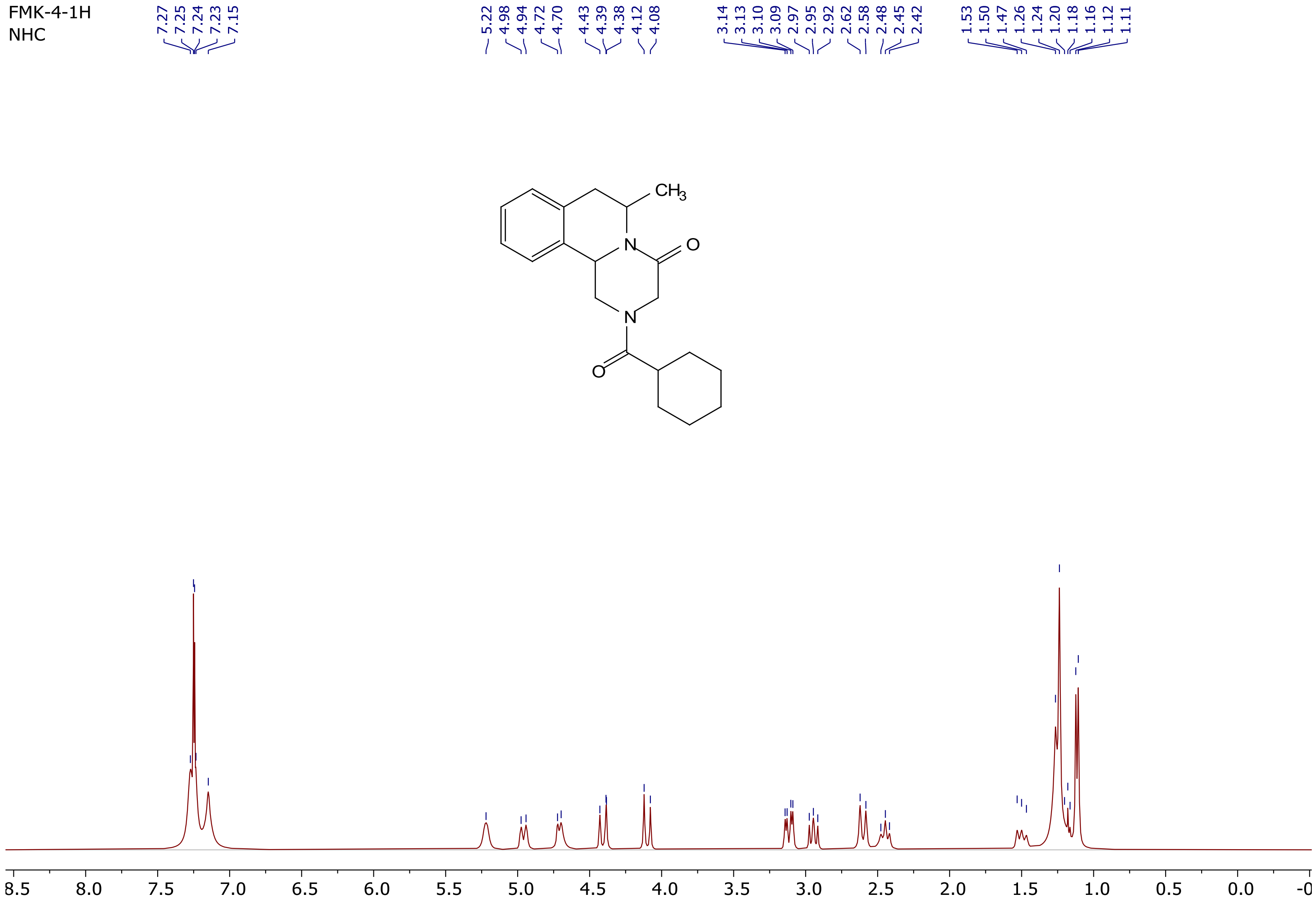

^{1}H-NMR spectrum of 2-(Cyclohexylcarbonyl)-6-methyl-1,2,3,6,7,11b-hexahydro-4H-pyrazino[2,1-a]isoquinolin-4-one,

6-methylpraziquantel **22**

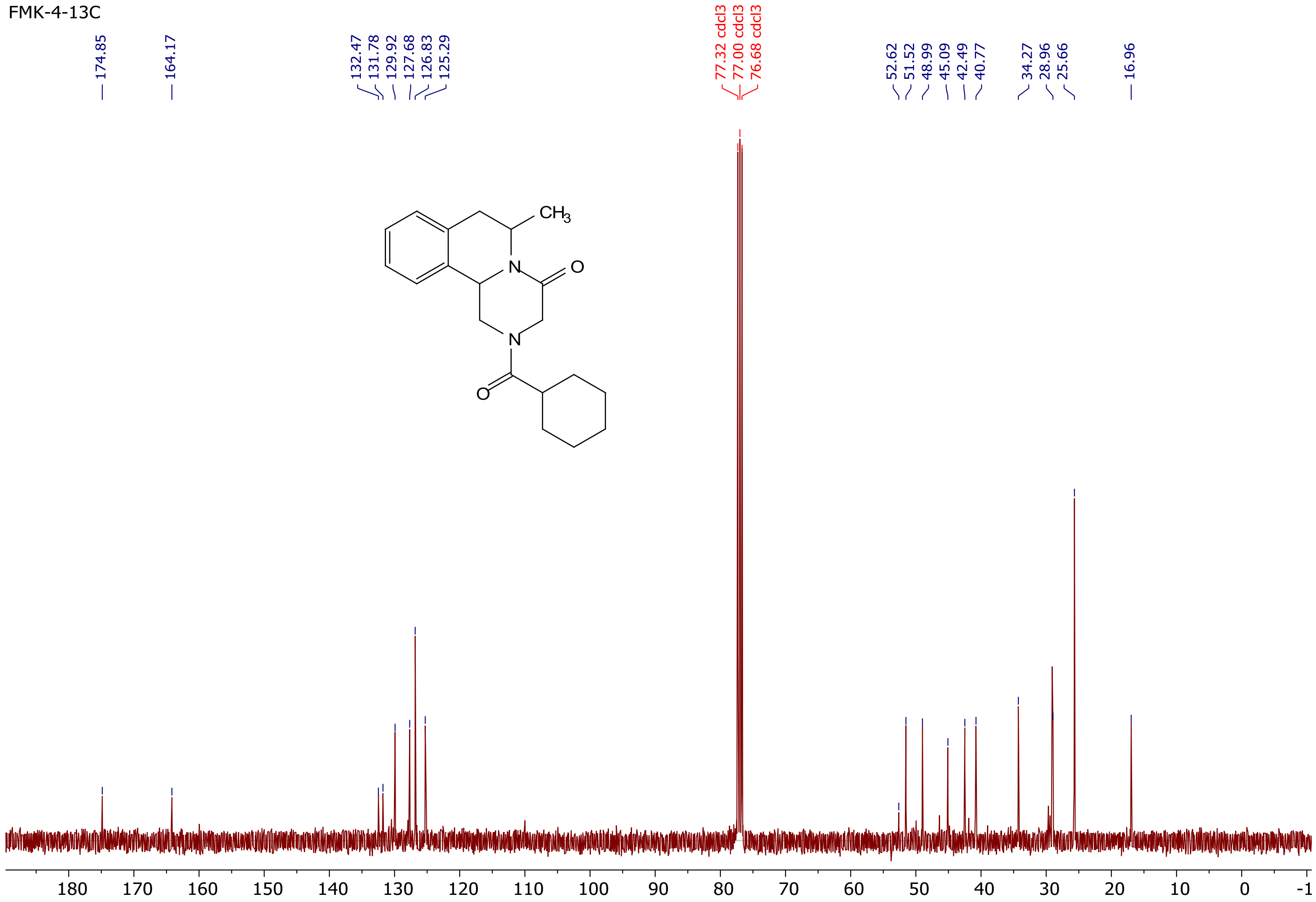

^{13}C-NMR spectrum of 2-(Cyclohexylcarbonyl)-6-methyl-1,2,3,6,7,11b-hexahydro-4H-pyrazino[2,1-a]isoquinolin-4-one,

6-methylpraziquantel **22**

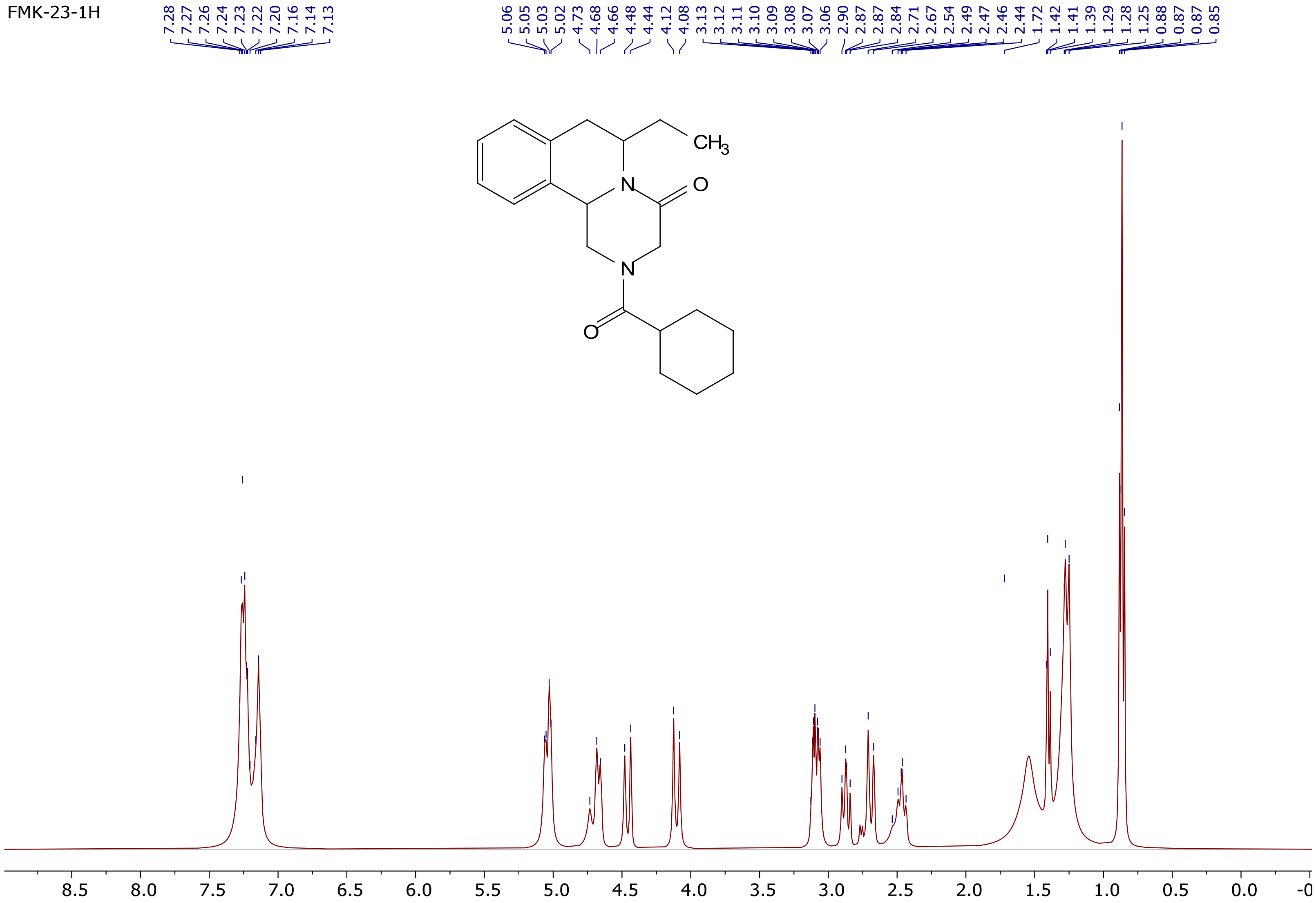

^{1}H-NMR spectrum of 2-Cyclohexanecarbonyl-6-ethyl-1,2,3,6,7,11b-hexahydro-pyrazino[2,1-a]isoquinolin-4-one, 6-ethylpraziquantel **23**

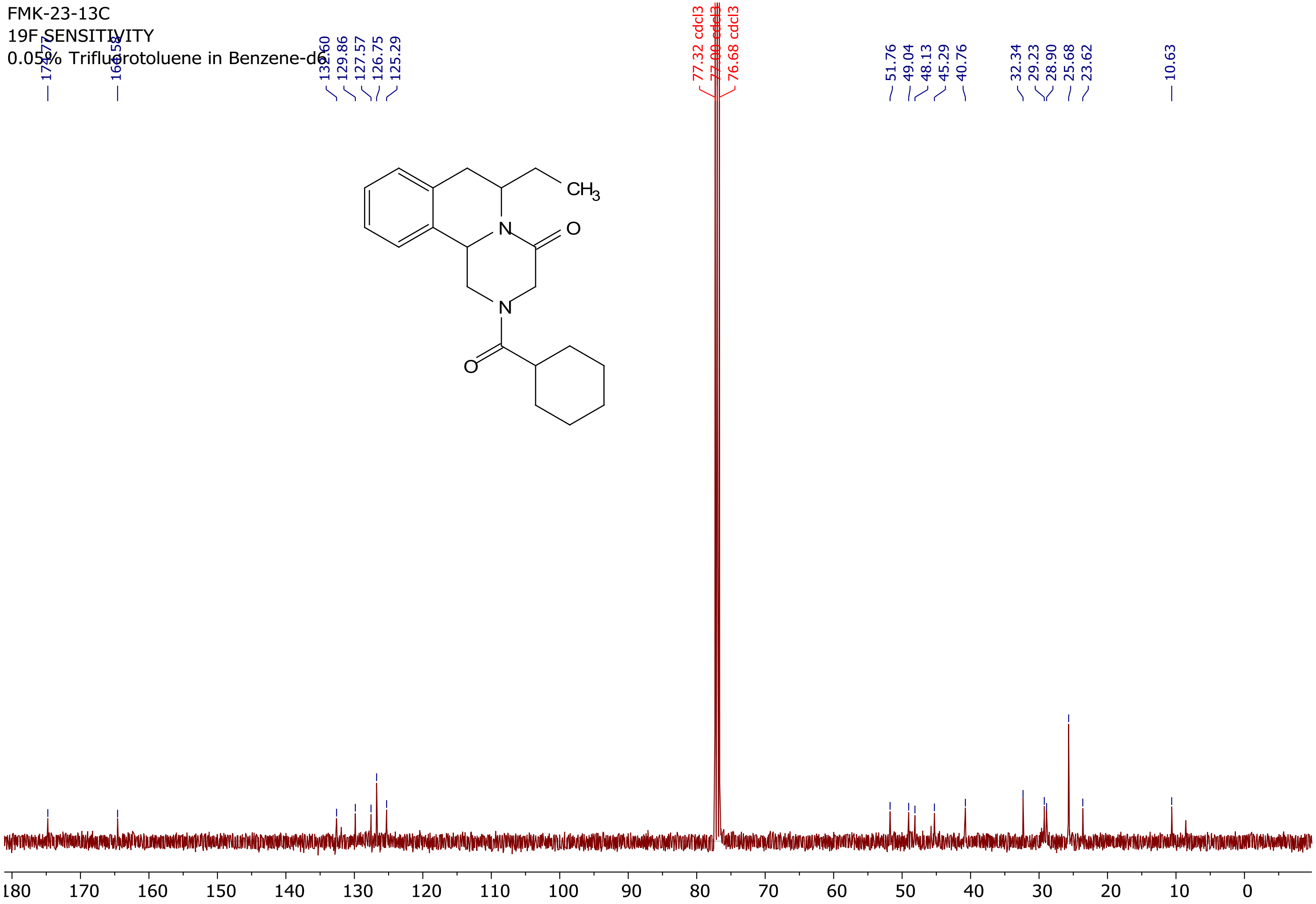

^{13}C-NMR spectrum of 2-Cyclohexanecarbonyl-6-ethyl-1,2,3,6,7,11b-hexahydro-pyrazino[2,1-a]isoquinolin-4-one, 6-ethylpraziquantel **23**

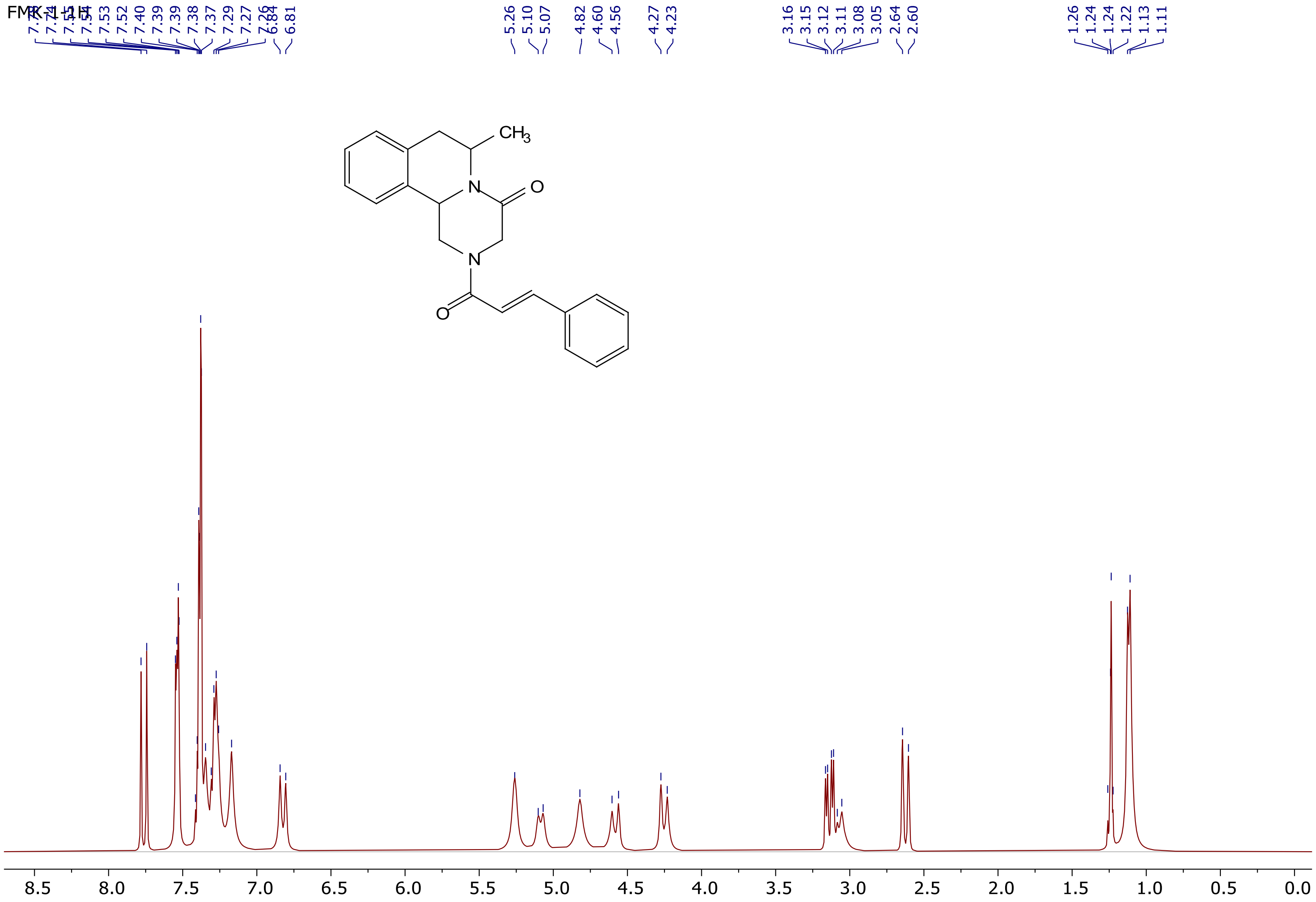

^{1}H-NMR spectrum of 6-Methyl-2-[(*2E*)-3-phenylprop-2-enoyl]-1,2,3,6,7,11b-hexahydro-4H-pyrazino[2,1-a]isoquinolin-4-one **24**

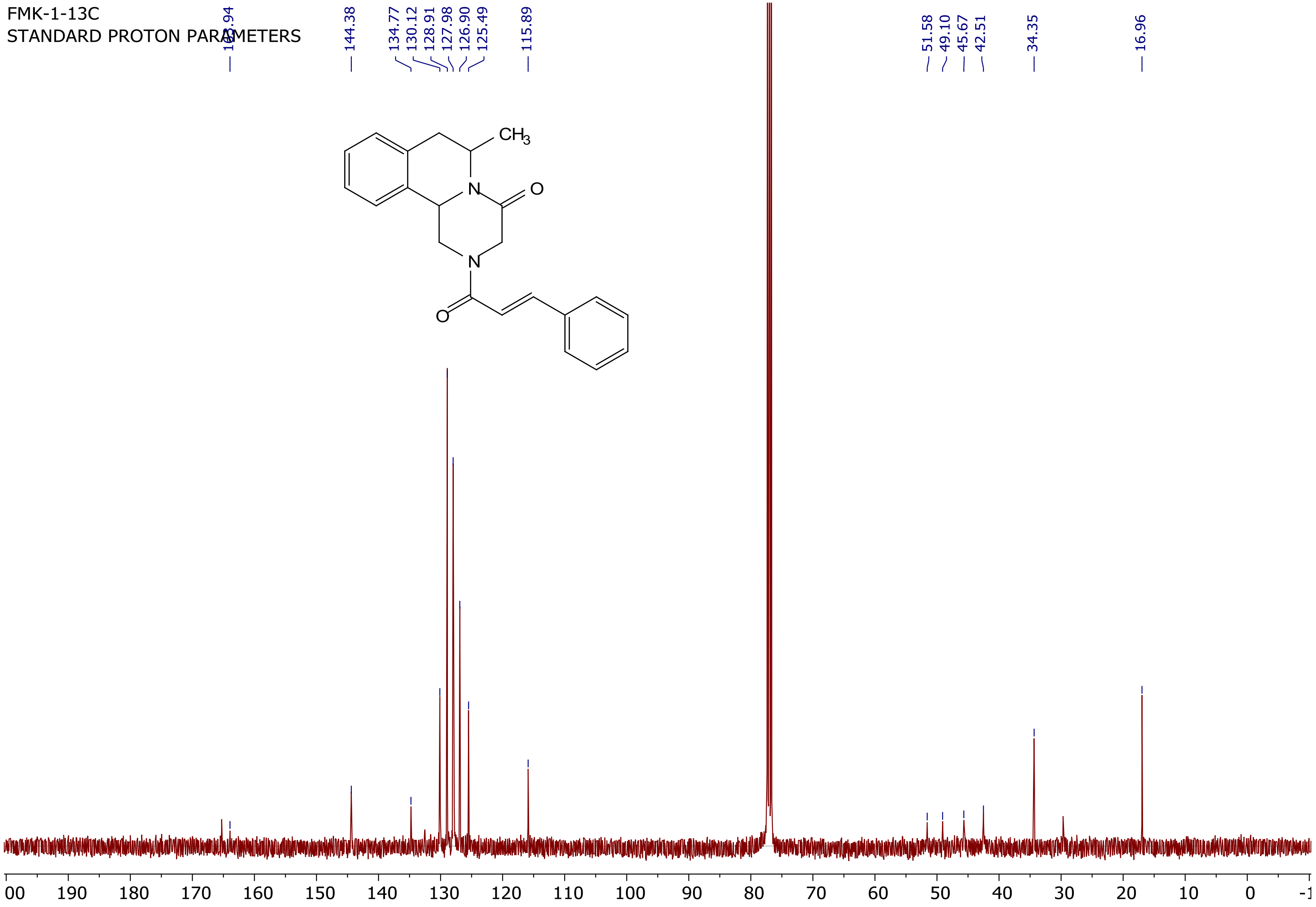

^{13}C-NMR spectrum of 6-Methyl-2-[(*2E*)-3-phenylprop-2-enoyl]-1,2,3,6,7,11b-hexahydro-4H-pyrazino[2,1-a]isoquinolin-4-one **24**

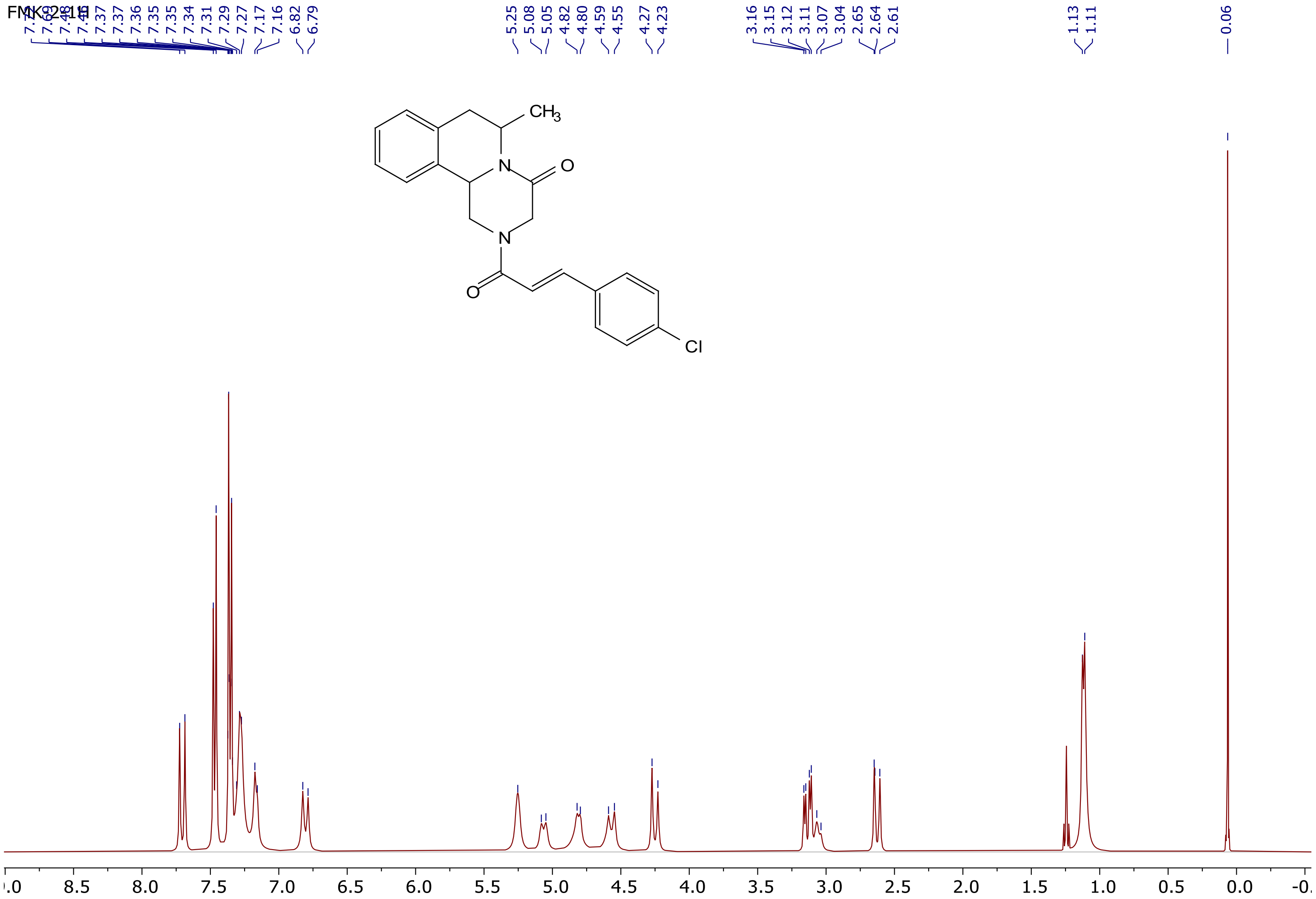

^{1}H-NMR spectrum of 2-[(*2E*)-3-(4-Chlorophenyl)prop-2-enoyl]-6-methyl-1,2,3,6,7,11b-hexahydro-4H-pyrazino[2,1-a]isoquinolin-4-one **25**

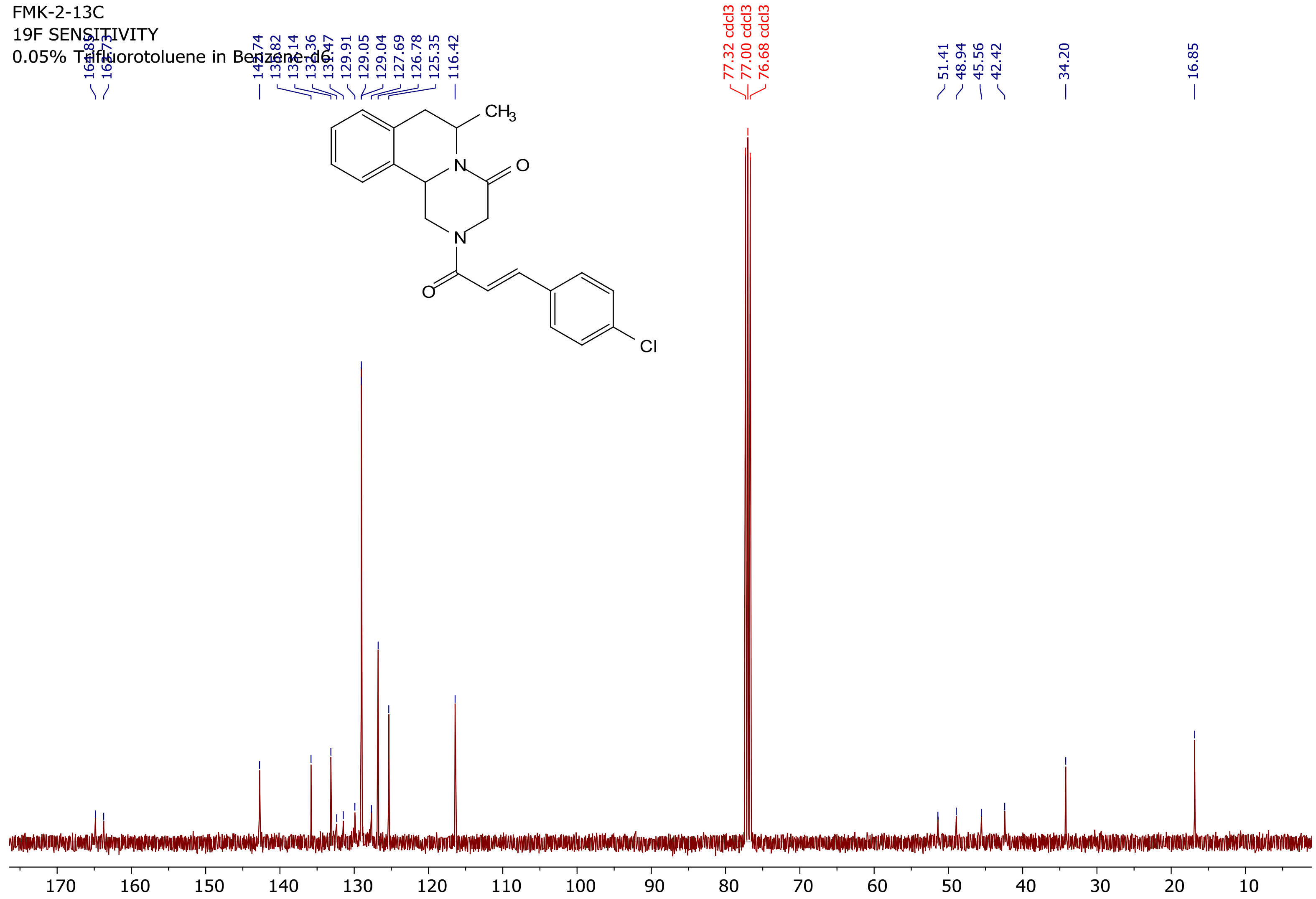

^{13}C-NMR spectrum of 2-[(*2E*)-3-(4-Chlorophenyl)prop-2-enoyl]-6-methyl-1,2,3,6,7,11b-hexahydro-4H-pyrazino[2,1-a]isoquinolin-4-one **25**

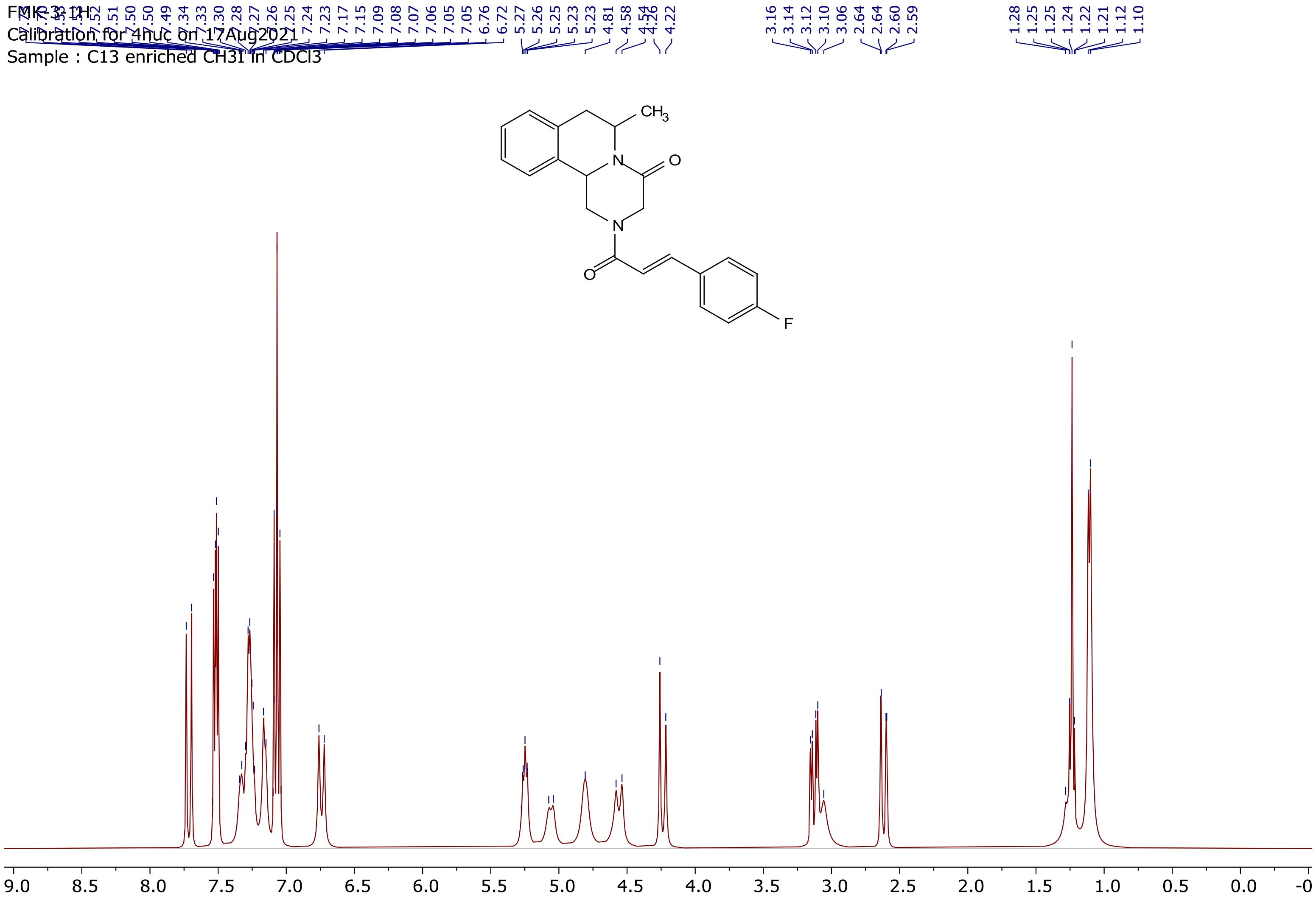

^{1}H-NMR spectrum of 2-[(2*E*)-3-(4-Fluorophenyl)prop-2-enoyl]-6-methyl-1,2,3,6,7,11b-hexahydro-4H-pyrazino[2,1-a]isoquinolin-4-one **26**

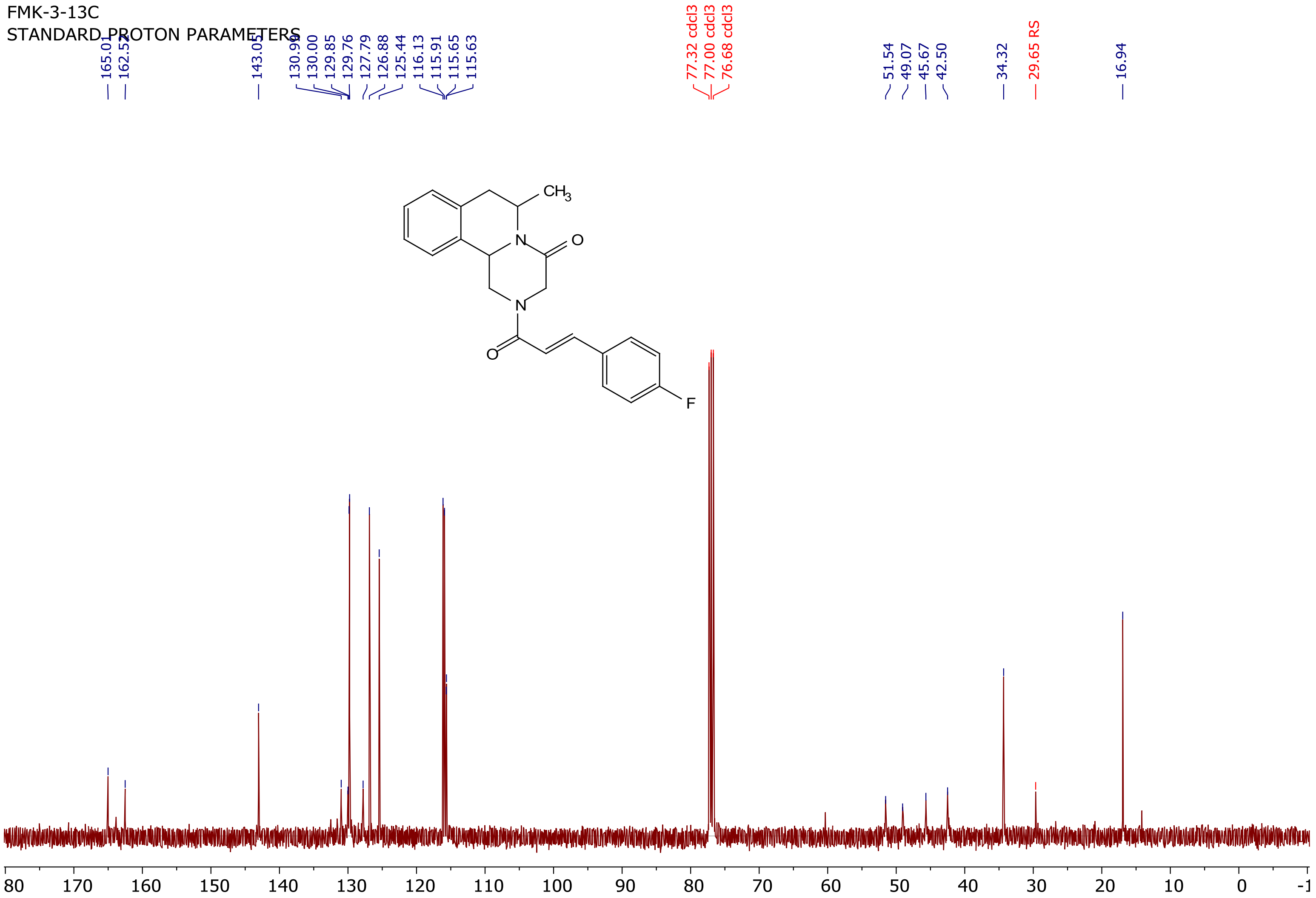

^{13}C-NMR spectrum of 2-[(2*E*)-3-(4-Fluorophenyl)prop-2-enoyl]-6-methyl-1,2,3,6,7,11b-hexahydro-4H-pyrazino[2,1-a]isoquinolin-4-one **26**

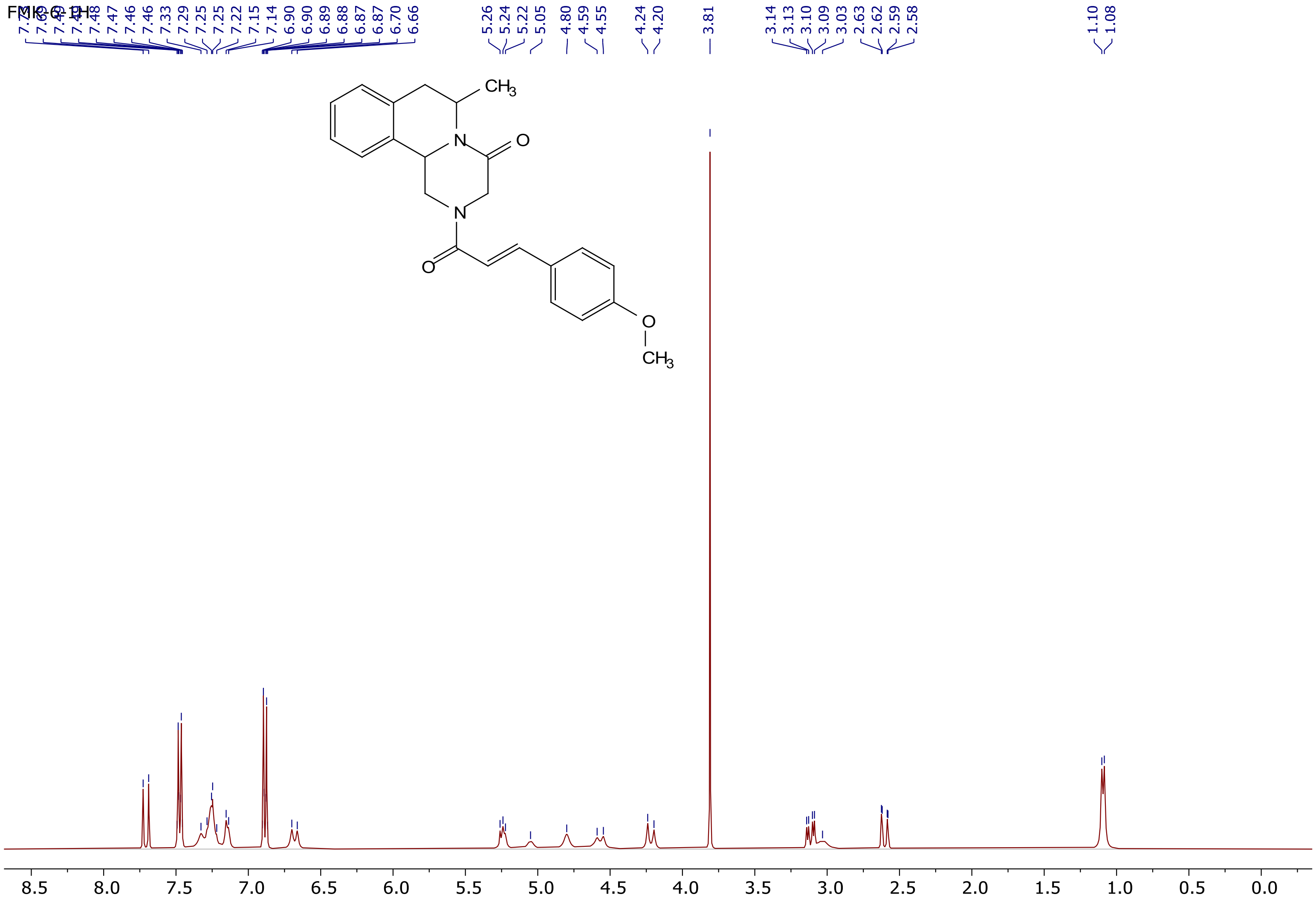

^{1}H-NMR spectrum of 2-[(*2E*)-3-(4-Methoxy-phenyl)prop-2-enoyl]-6-methyl-1,2,3,6,7,11b-hexahydro-pyrido[2,1-a]isoquinolin-4-one **27**

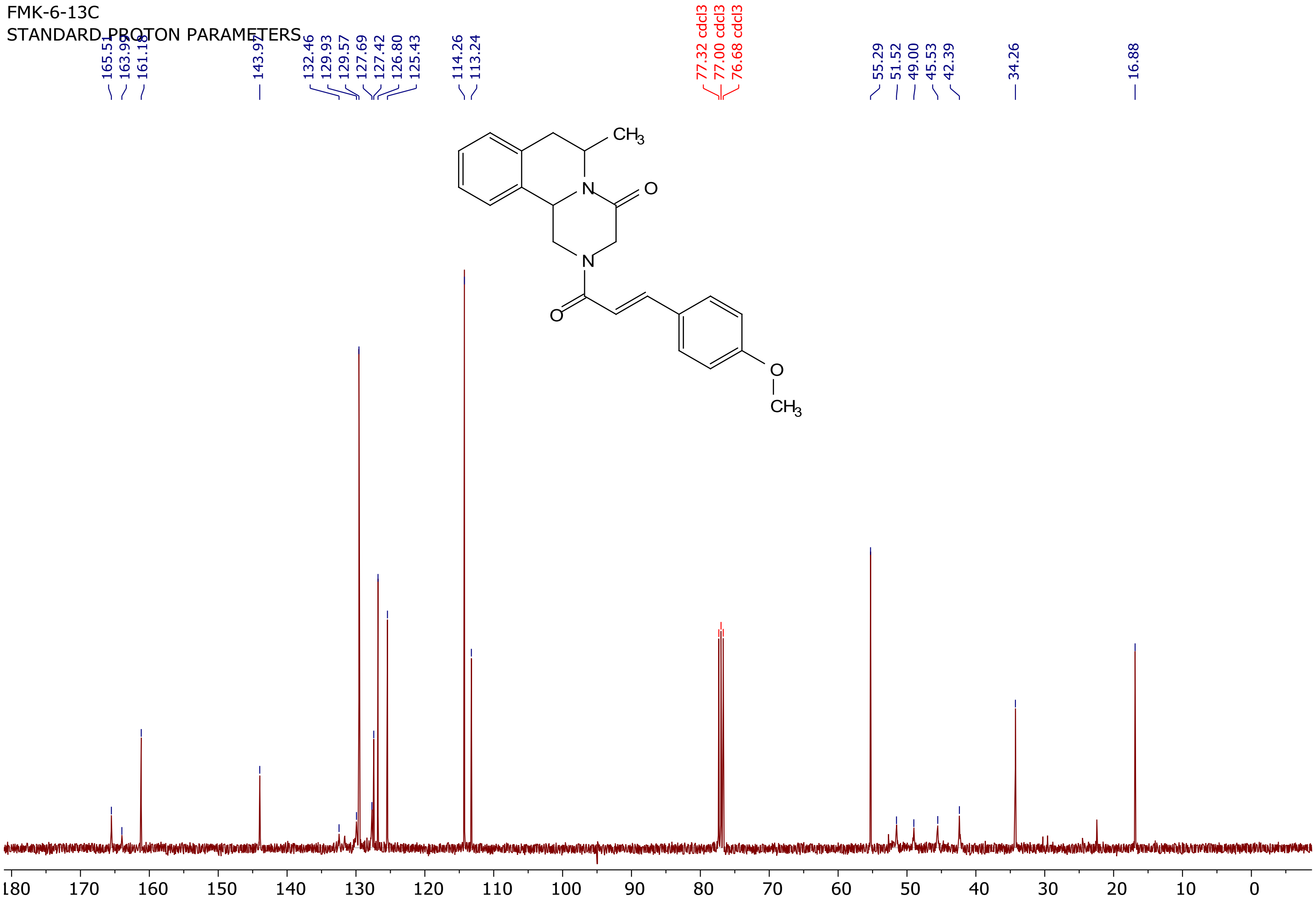

^{13}C-NMR spectrum of 2-[(*2E*)-3-(4-Methoxy-phenyl)prop-2-enoyl]-6-methyl-1,2,3,6,7,11b-hexahydro-pyrido[2,1-a]isoquinolin-4-one **27**

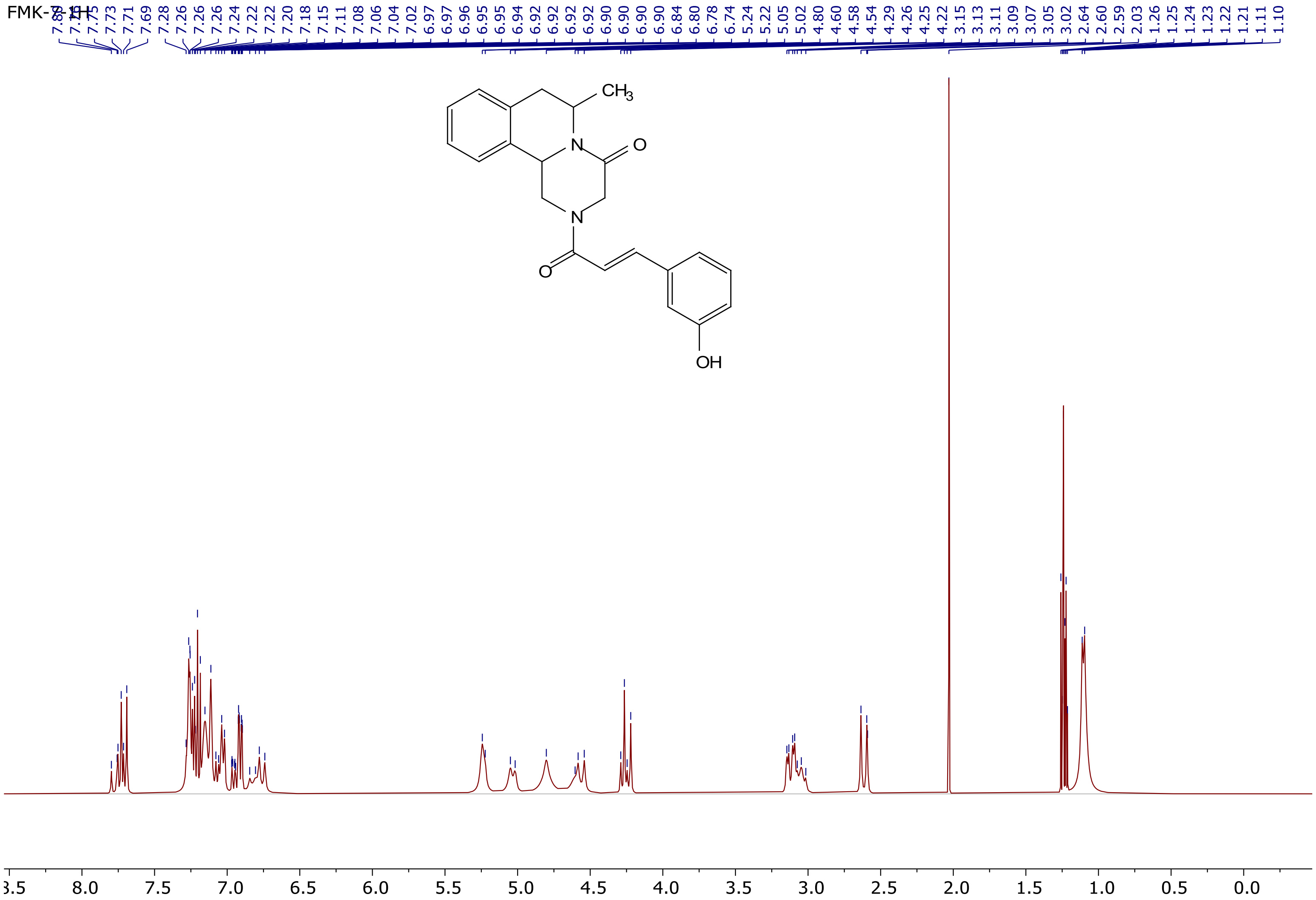

^{1}H-NMR spectrum of 2-[(*2E*)-3-(3-Hydroxyphenyl)prop-2-enoyl]-6-methyl-1,2,3,6,7,11b-hexahydro-4H-pyrazino[2,1-a]isoquinolin-4-one **28**

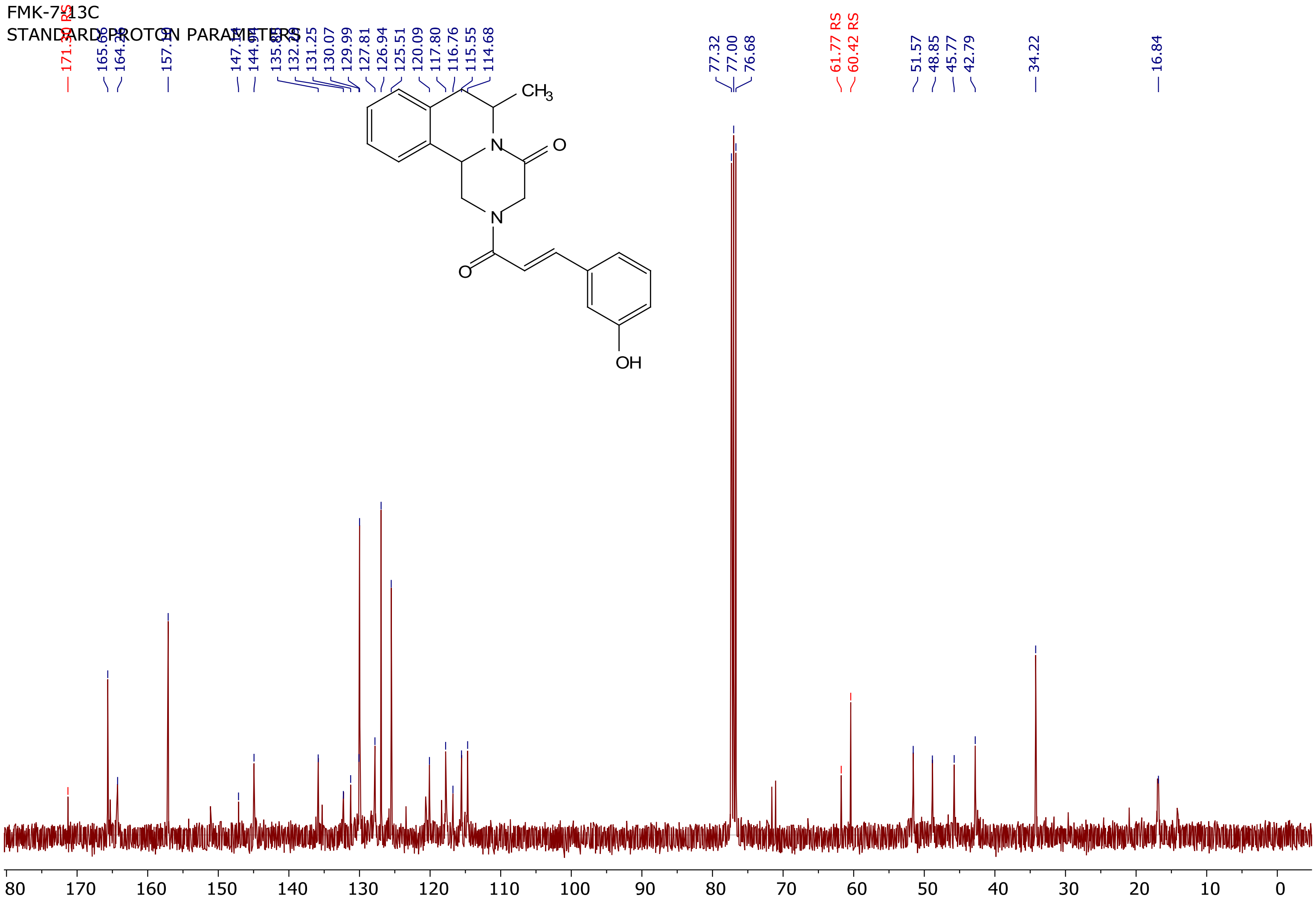

^{13}C-NMR spectrum of 2-[(*2E*)-3-(3-Hydroxyphenyl)prop-2-enoyl]-6-methyl-1,2,3,6,7,11b-hexahydro-4H-pyrazino[2,1-a]isoquinolin-4-one **28**

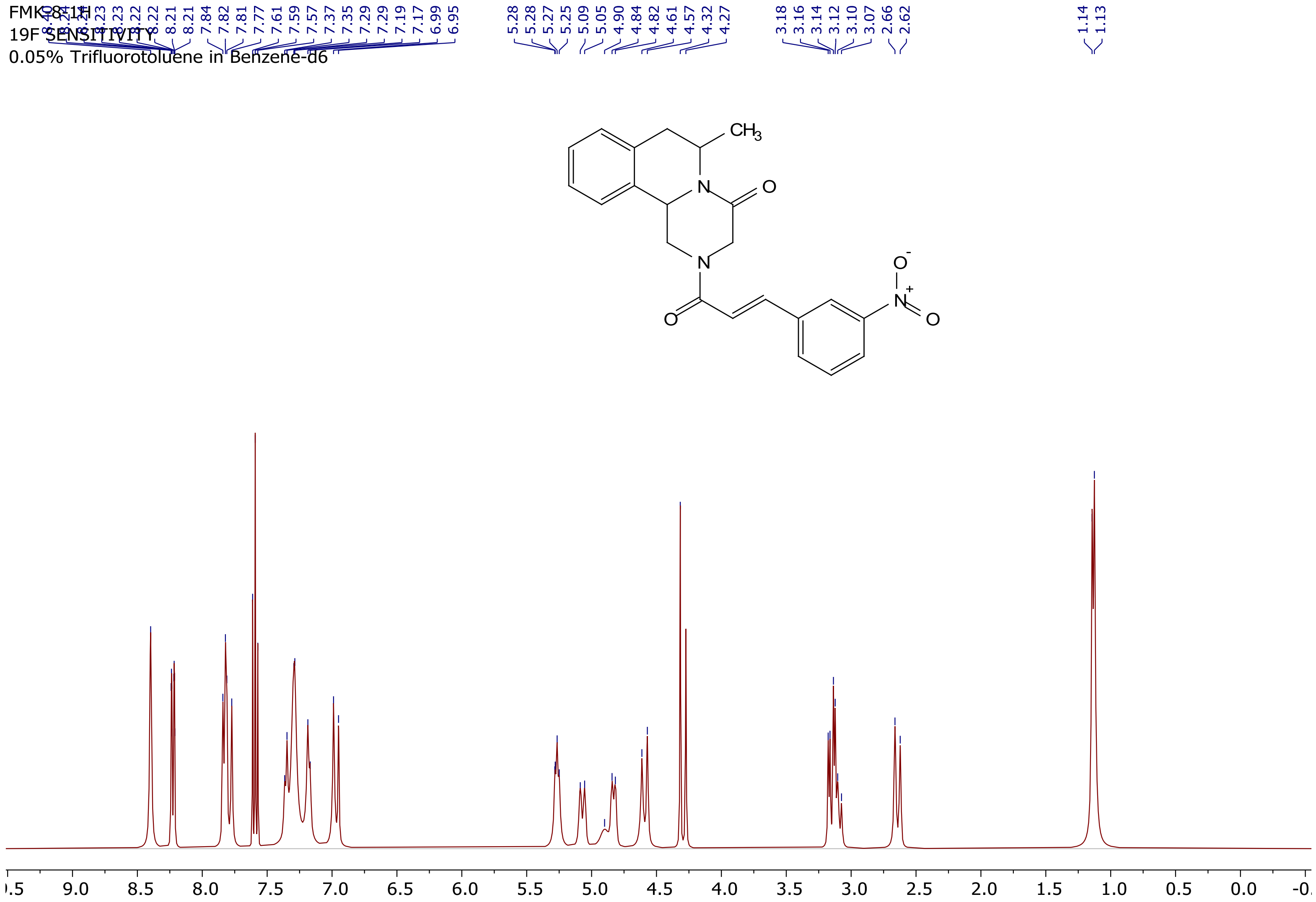

^{1}H-NMR spectrum of 2-[(*2E*)-3-(3-Nitrophenyl)prop-2-enoyl]- 6-methyl-1,2,3,6,7,11b-hexahydro-4H-pyrazino[2,1-a]isoquinolin-4-one **29**

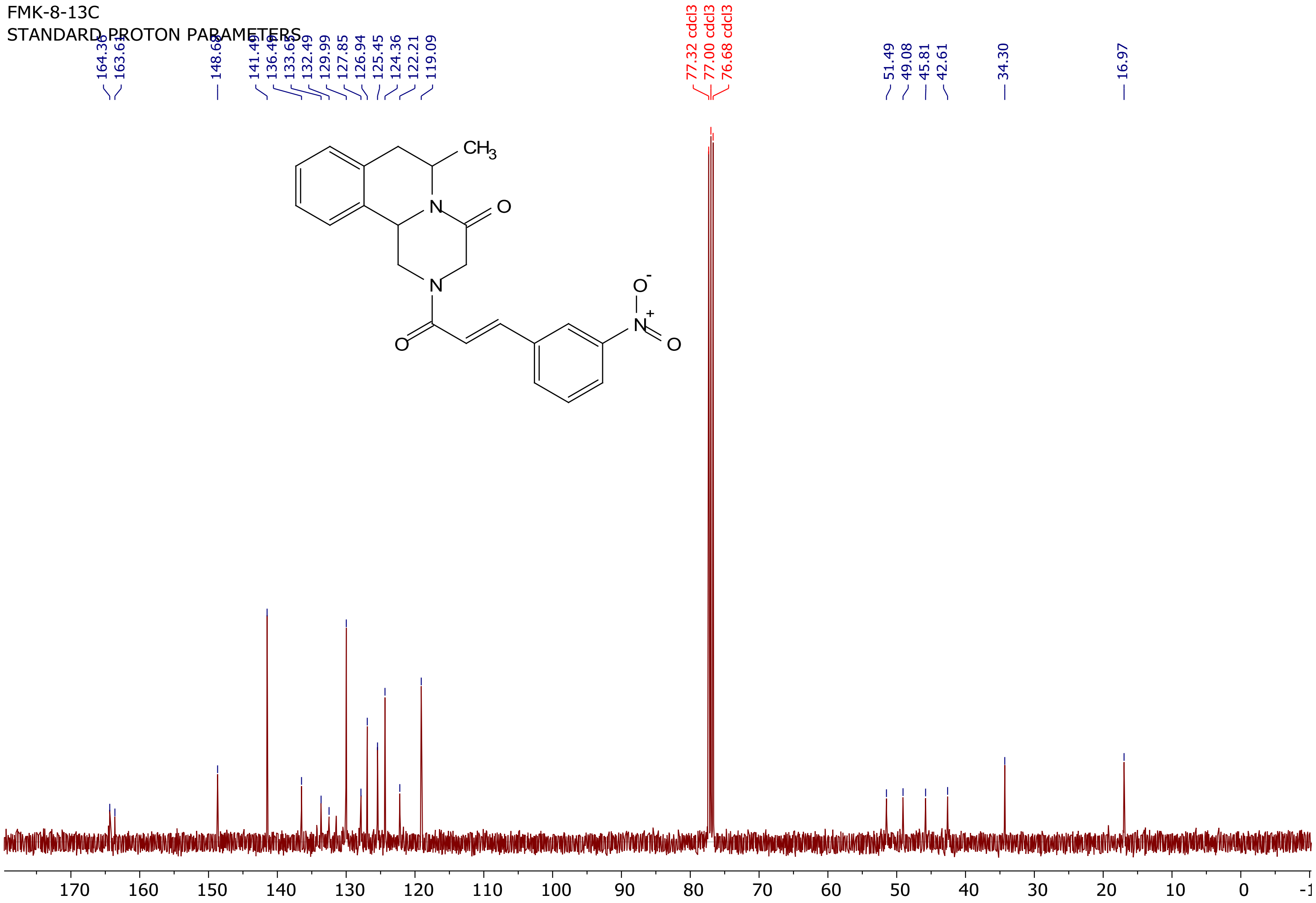

^{13}C-NMR spectrum of 2-[(2*E*)-3-(3-Nitrophenyl)prop-2-enoyl]- 6-methyl-1,2,3,6,7,11b-hexahydro-4H-pyrazino[2,1-a]isoquinolin-4-one **29**

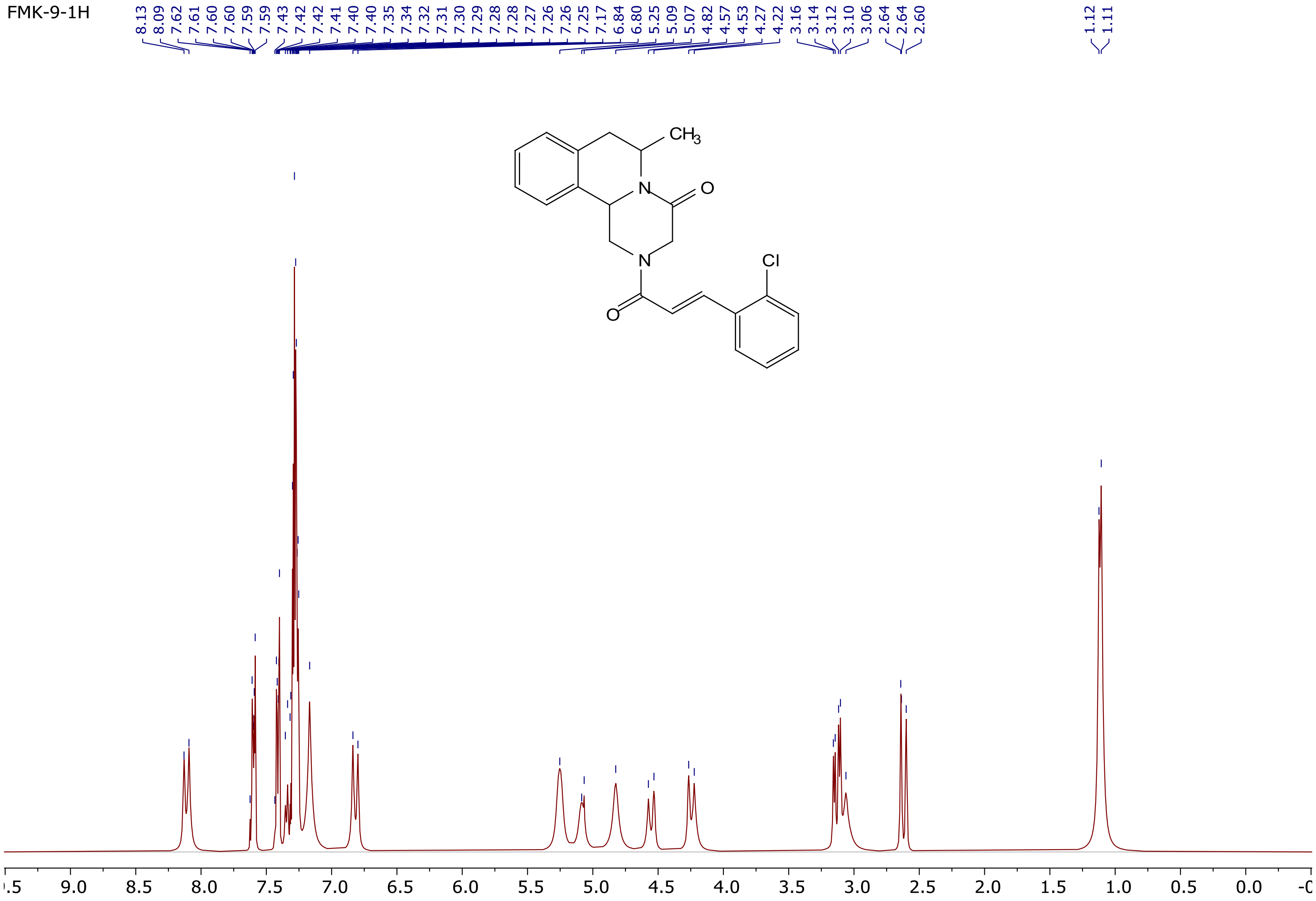

^{1}H-NMR spectrum of 2-[(*2E*)-3-(2-Chlorophenyl)prop-2-enoyl]-6-methyl-1,2,3,6,7,11b-hexahydro-4H-pyrazino[2,1-a]isoquinolin-4-one **30**

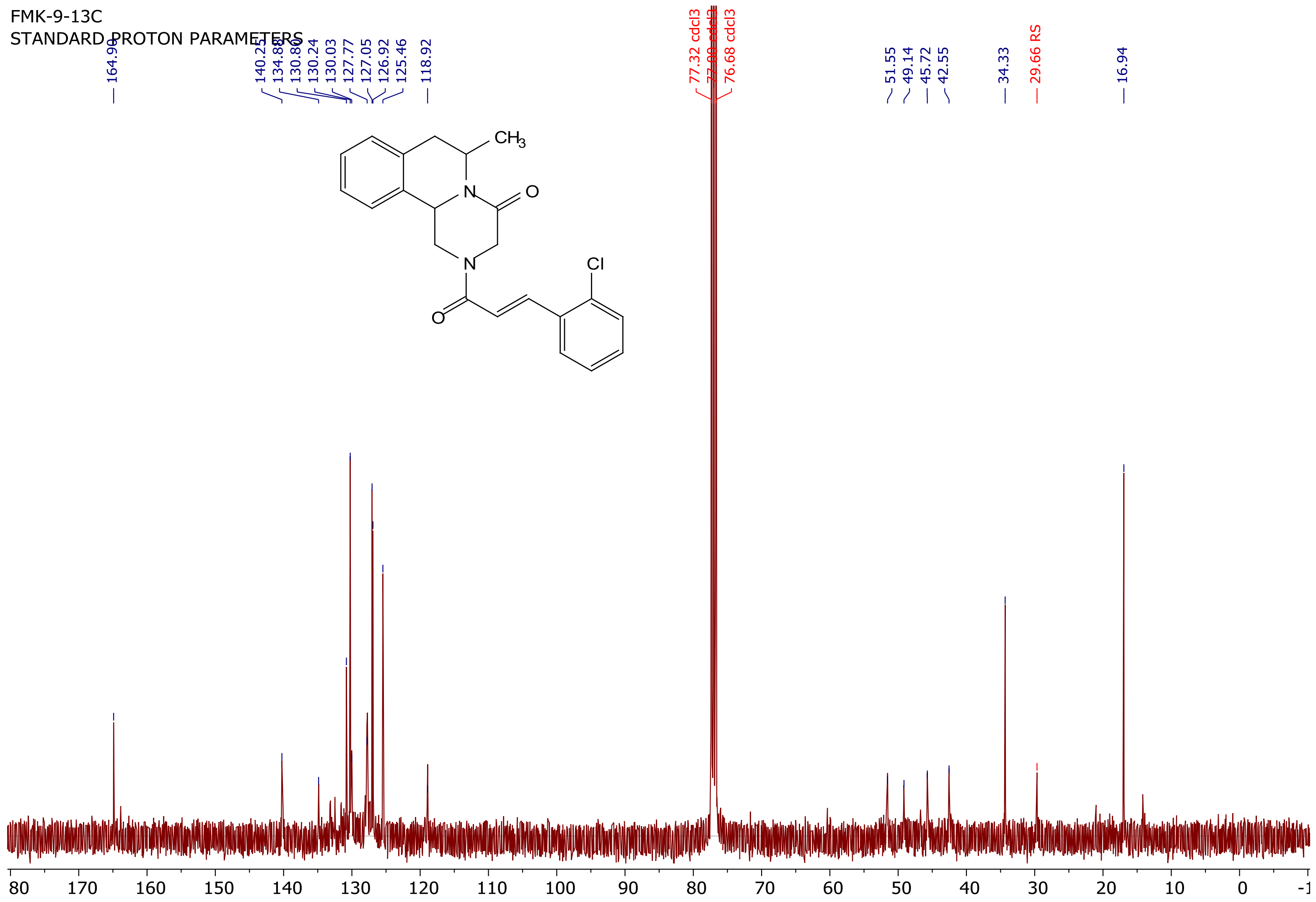

^{13}C-NMR spectrum of 2-[(*2E*)-3-(2-Chlorophenyl)prop-2-enoyl]-6-methyl-1,2,3,6,7,11b-hexahydro-4H-pyrazino[2,1-a]isoquinolin-4-one **30**

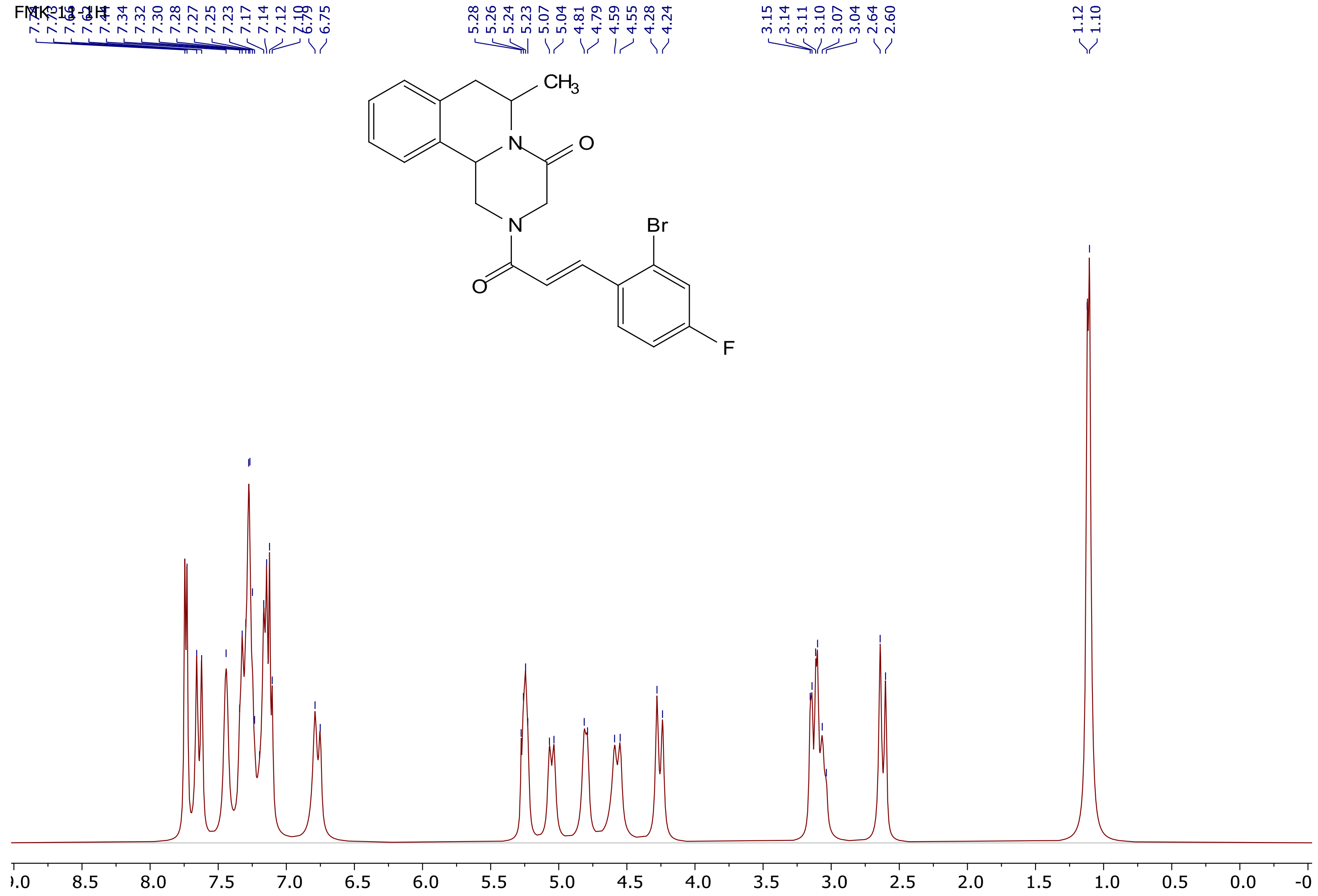

^{1}H-NMR spectrum of 2-[(2*E*)-3-(2-Bromo-4-fluorophenyl)prop-2-enoyl]-6-methyl-1,2,3,6,7,11b-hexahydro-4H-pyrazino[2,1-a]isoquinolin-4-one **31**

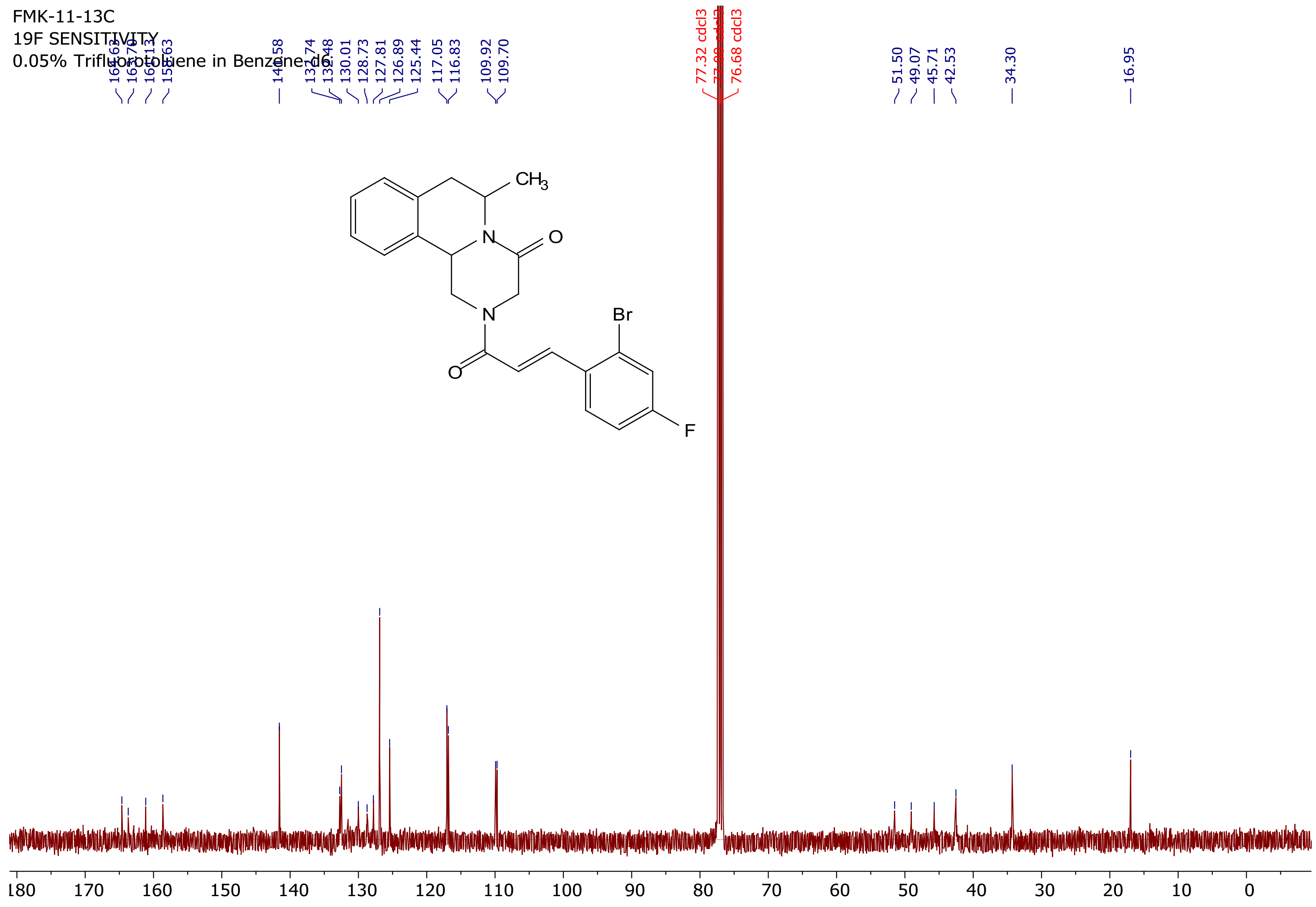

^{13}C-NMR spectrum of 2-[(*2E*)-3-(2-Bromo-4-fluorophenyl)prop-2-enoyl]-6-methyl-1,2,3,6,7,11b-hexahydro-4H-pyrazino[2,1-a]isoquinolin-4-one

31

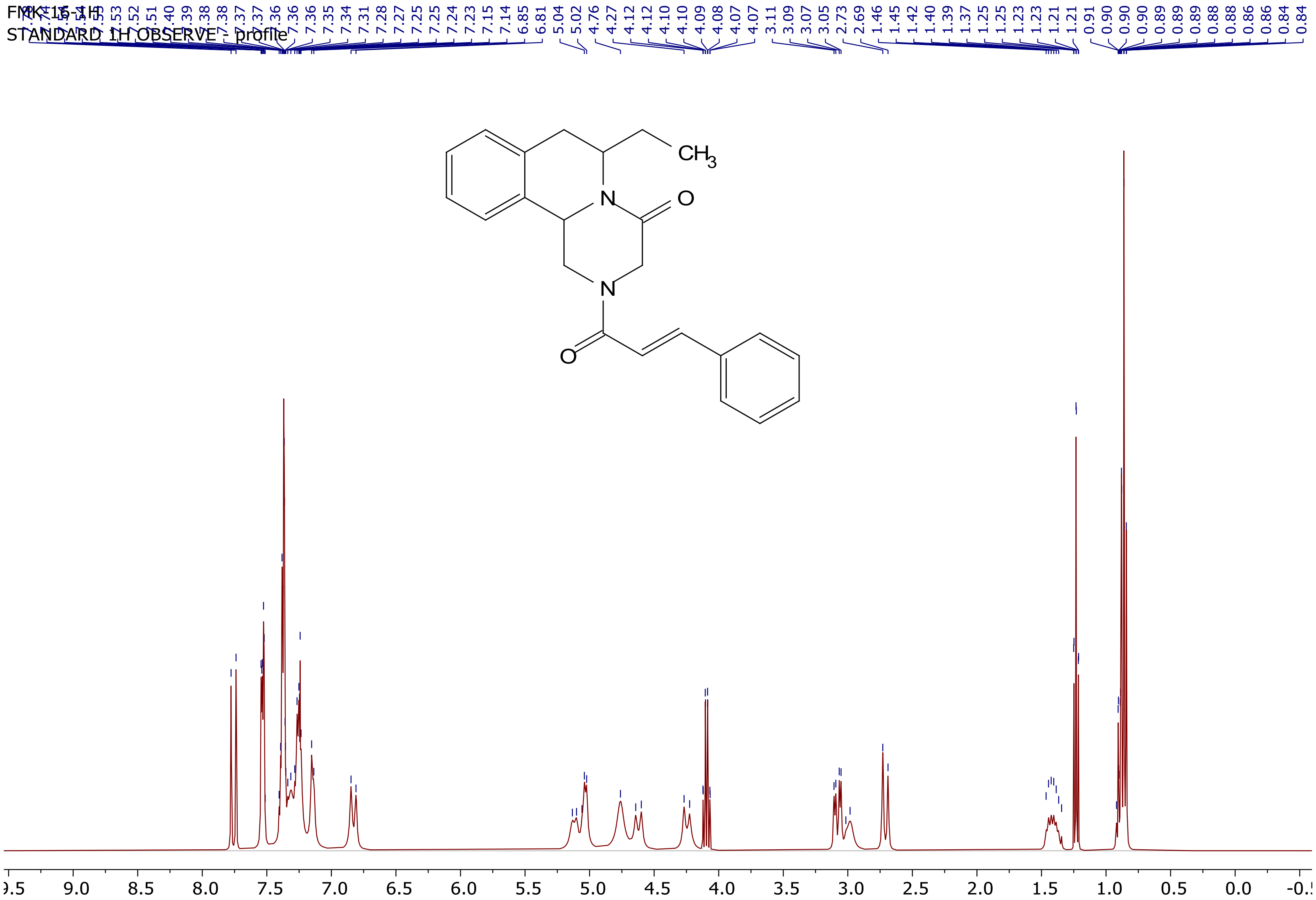

^{1}H-NMR spectrum of 6-Ethyl-2-[(*2E*)-3-phenylprop-2-enoyl]-1,2,3,6,7,11b-hexahydro-4H-pyrazino[2,1-a]isoquinolin-4-one **34**

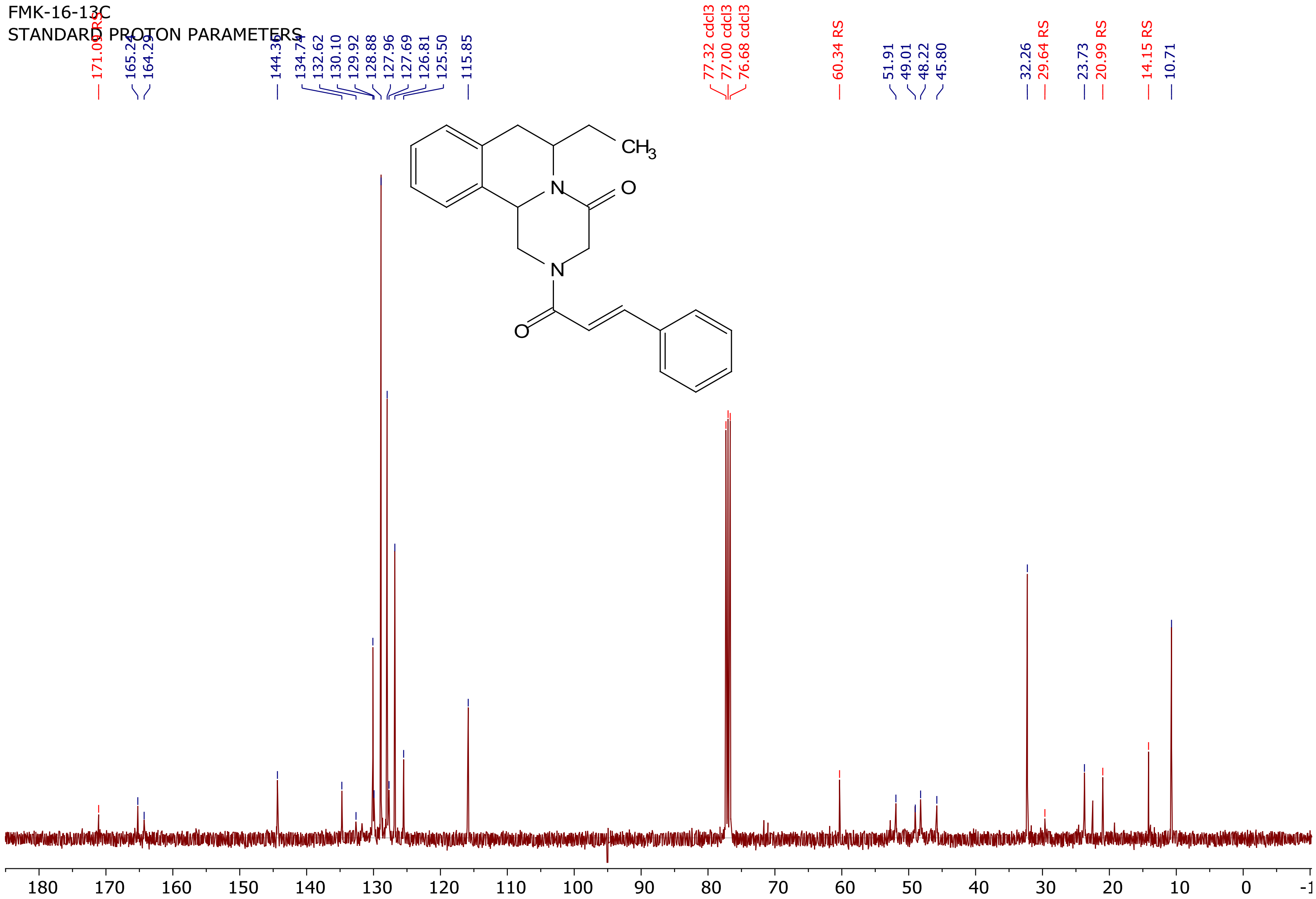

^{13}C-NMR spectrum of 6-Ethyl-2-[(*2E*)-3-phenylprop-2-enoyl]-1,2,3,6,7,11b-hexahydro-4H-pyrazino[2,1-a]isoquinolin-4-one **34**

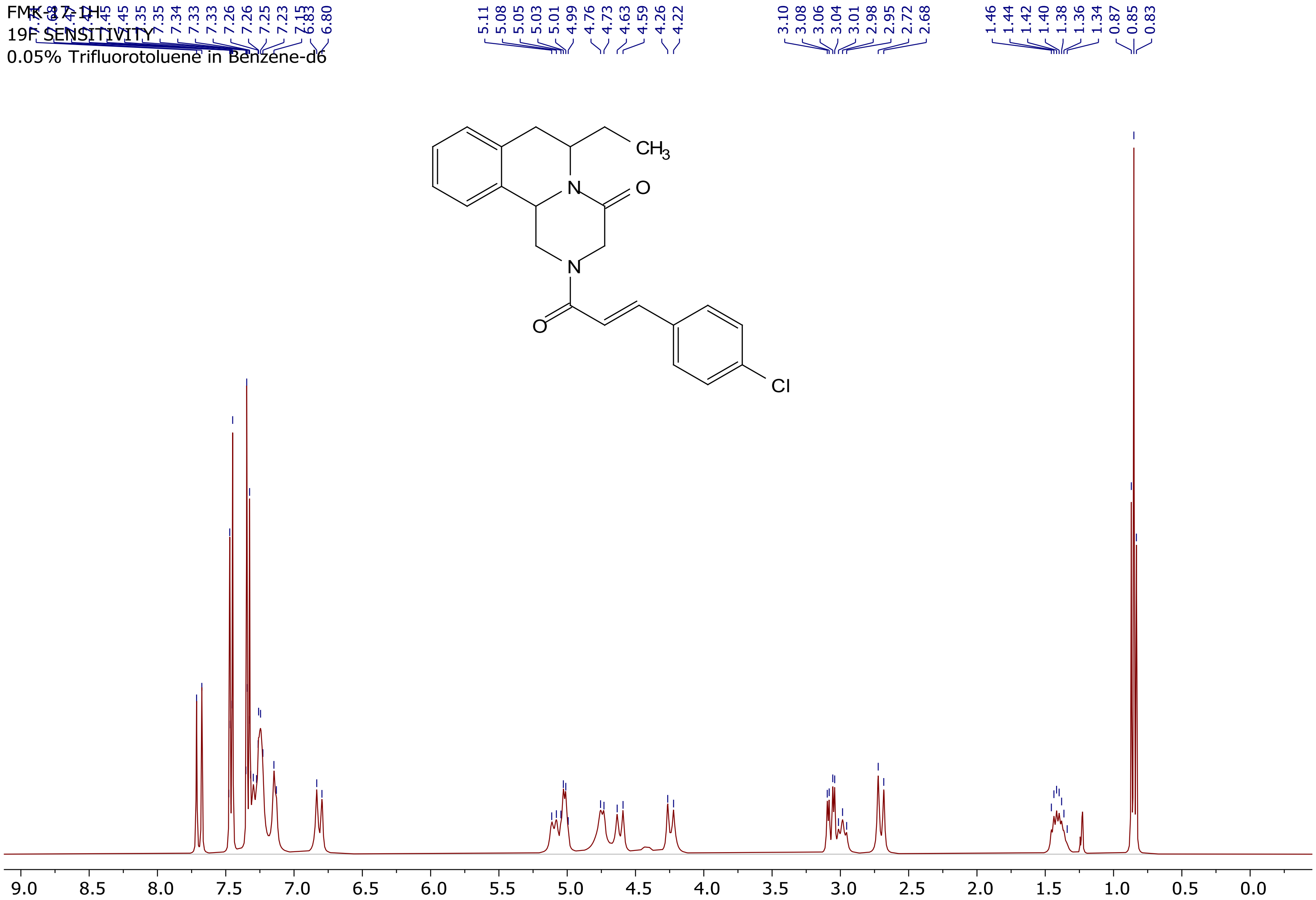

^{1}H-NMR spectrum of 2-[(*2E*)-3-(4-Chloro-phenyl)-acryloyl]-6-ethyl-1,2,3,6,7,11b-hexahydro-pyrazino[2,1-a]isoquinolin-4-one **35**

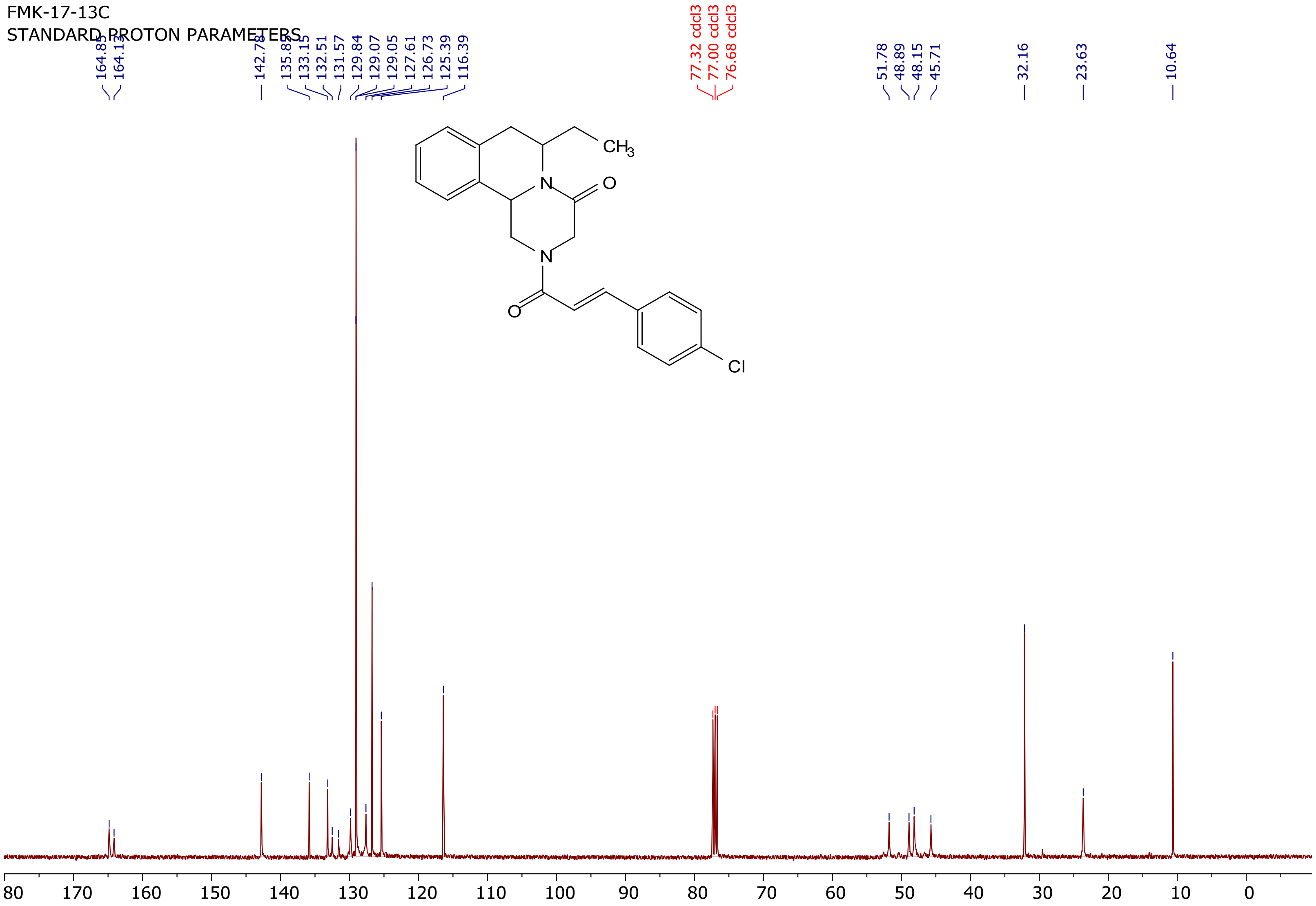

^{13}C-NMR spectrum of 2-[(2*E*)-3-(4-Chloro-phenyl)-acryloyl]-6-ethyl-1,2,3,6,7,11b-hexahydro-pyrazino[2,1-a]isoquinolin-4-one **35**

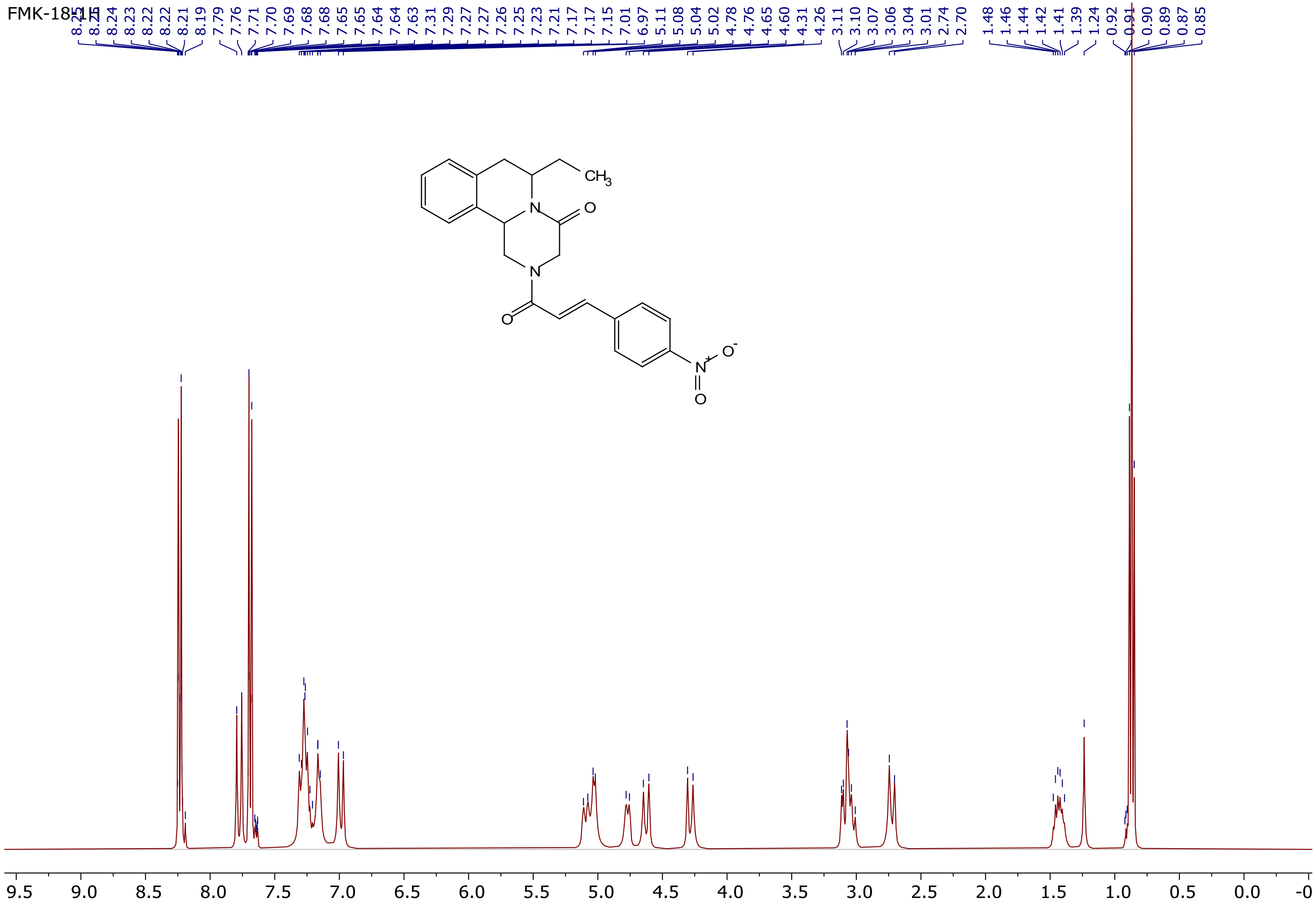

^{1}H-NMR spectrum of 2-[(*2E*)-3-(4-Nitro-phenyl)-acryloyl]-6-ethyl-1,2,3,6,7,11b-hexahydro-pyrazino[2,1-a]isoquinolin-4-one **36**

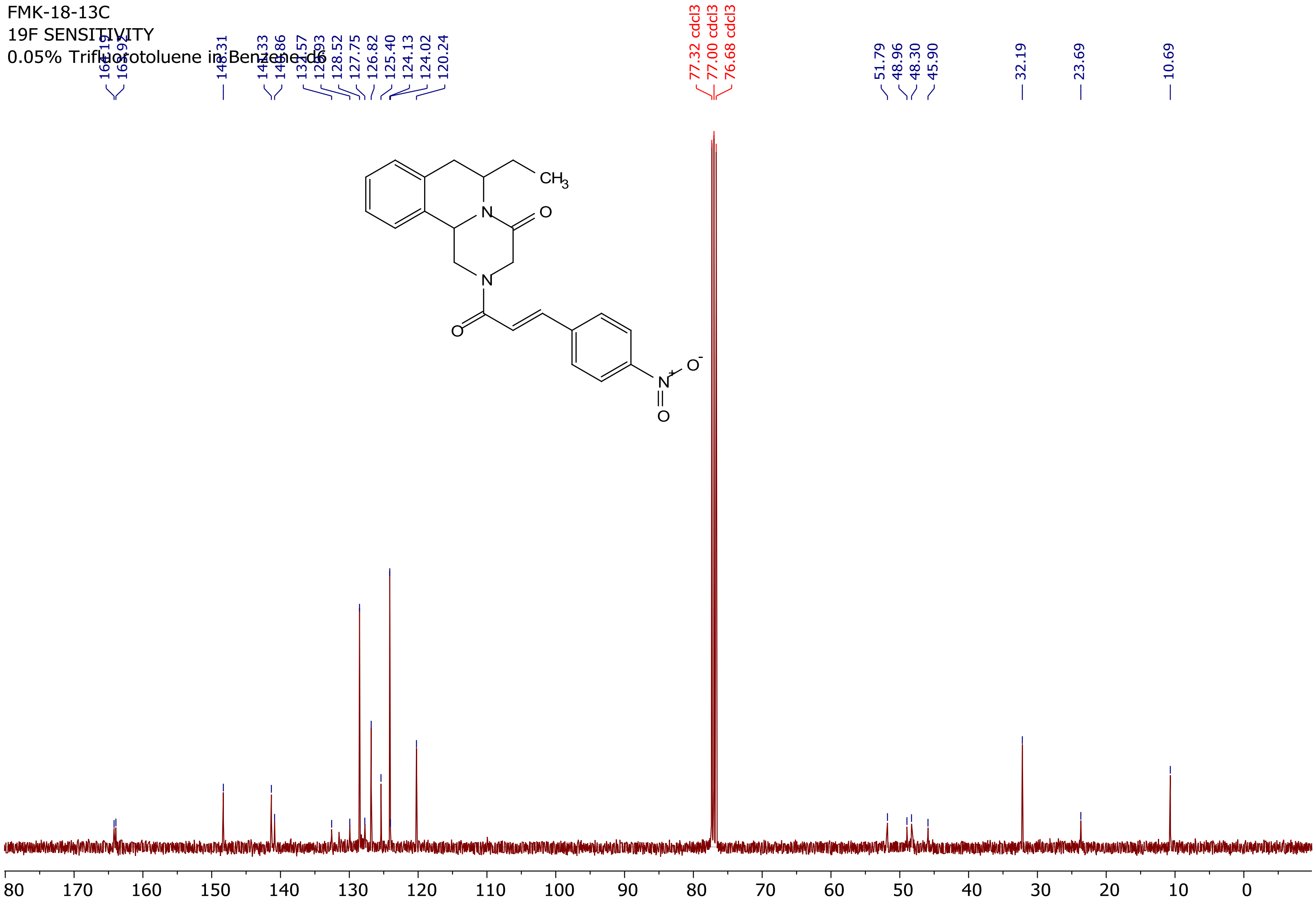

^{13}C-NMR spectrum of 2-[(*2E*)-3-(4-Nitro-phenyl)-acryloyl]-6-ethyl-1,2,3,6,7,11b-hexahydro-pyrazino[2,1-a]isoquinolin-4-one **36**

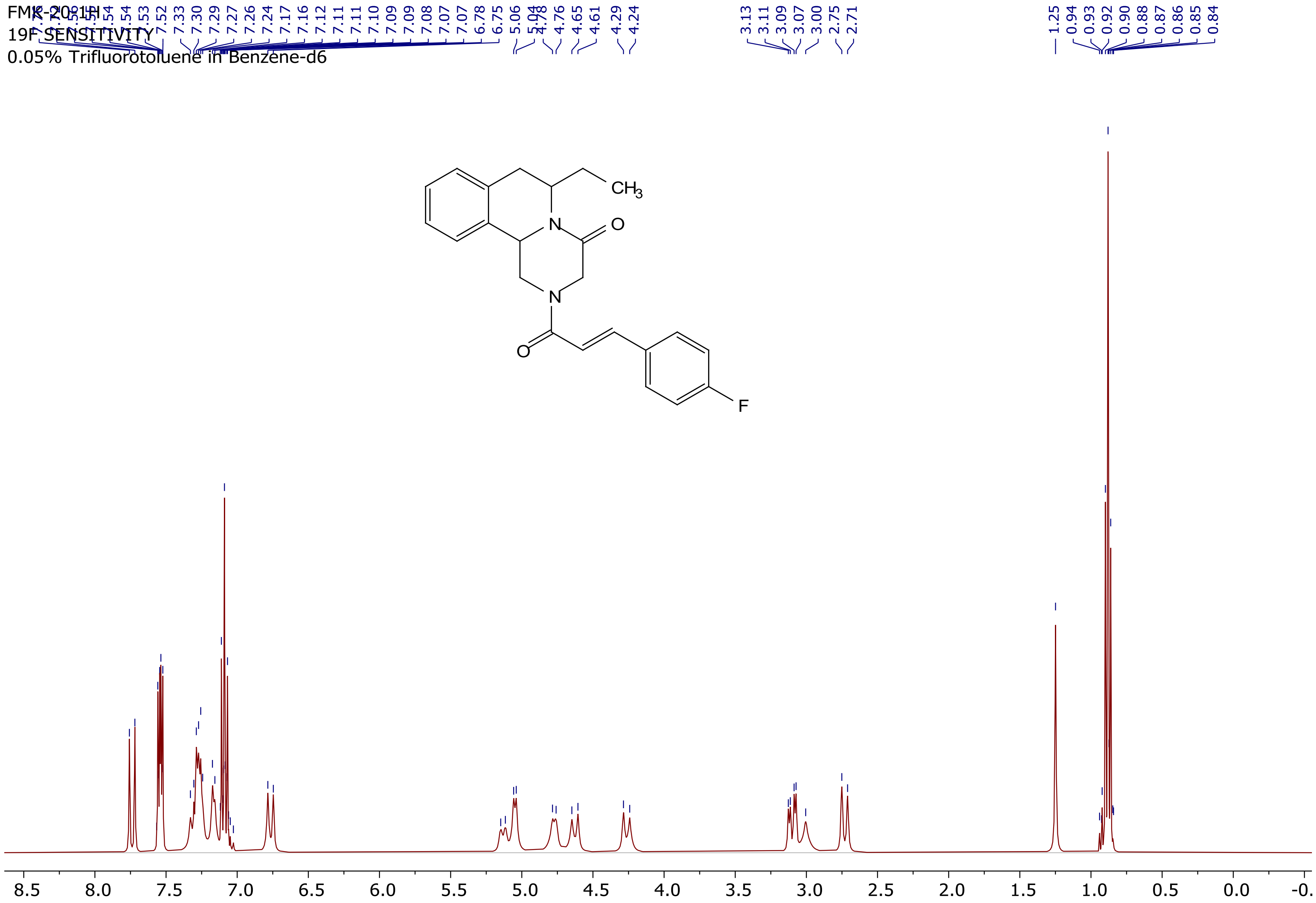

^{1}H-NMR spectrum of 2-[(*2E*)-3-(4-Fluoro-phenyl)-acryloyl]- 6-ethyl-1,2,3,6,7,11b-hexahydro-pyrazino[2,1-a]isoquinolin-4-one **37**

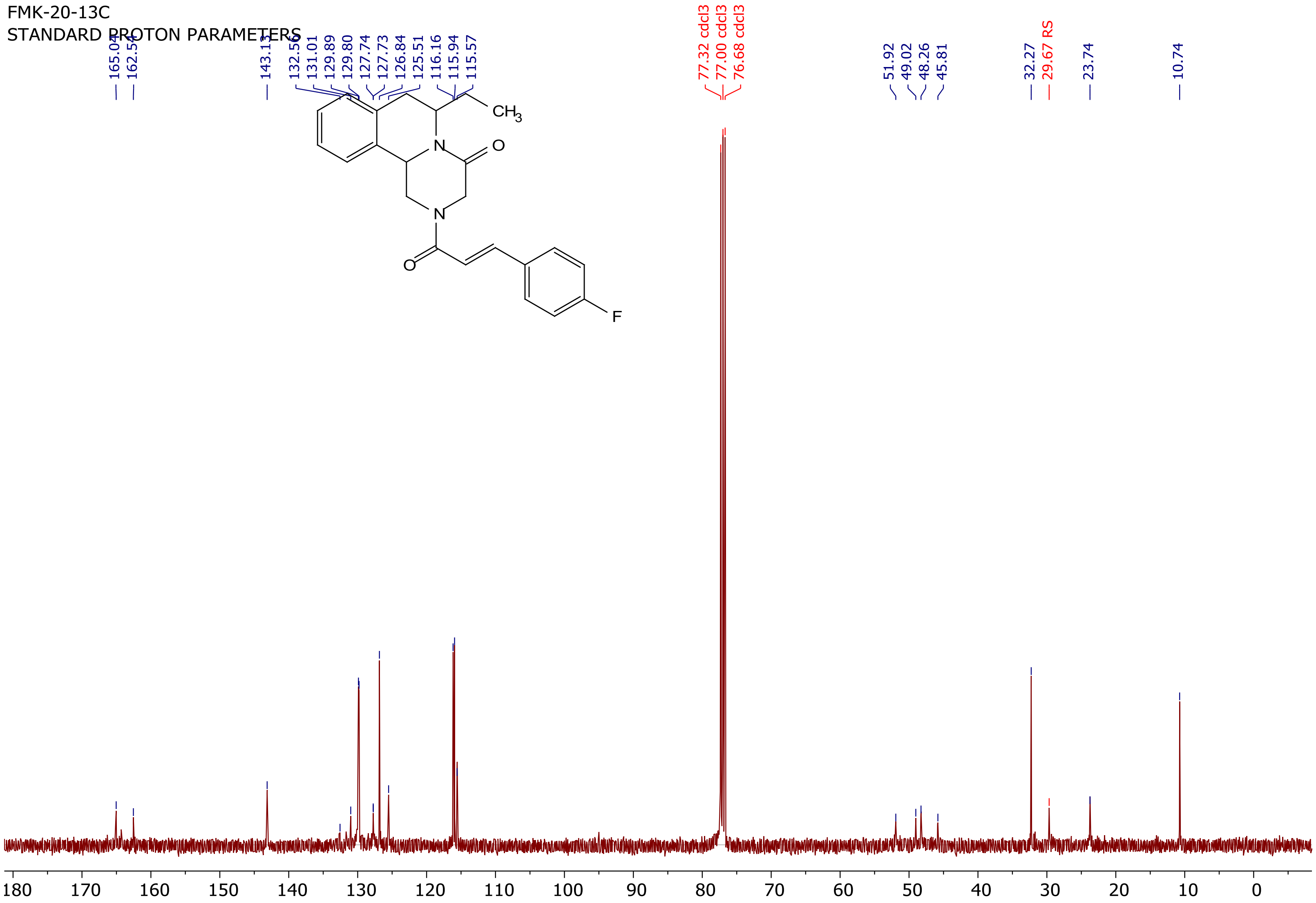

^{13}C-NMR spectrum of 2-[(*2E*)-3-(4-Fluoro-phenyl)-acryloyl]- 6-ethyl-1,2,3,6,7,11b-hexahydro-pyrazino[2,1-a]isoquinolin-4-one **37**

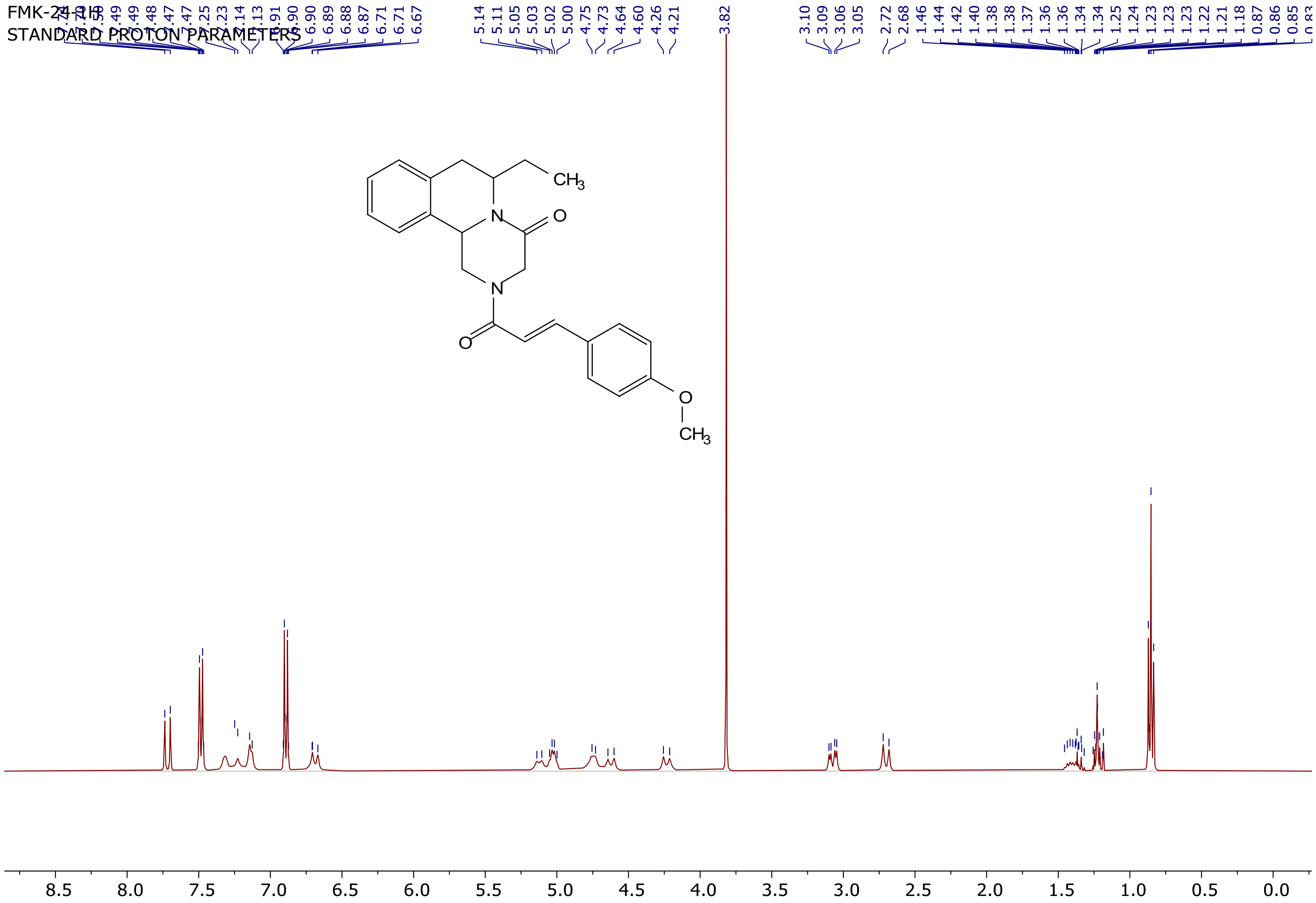

^{1}H-NMR spectrum of 2-[(*2E*)-3-(4-Methoxy-phenyl)-acryloyl]-6-ethyl-1,2,3,6,7,11b-hexahydro-pyrazino[2,1-a]isoquinolin-4-one **38**

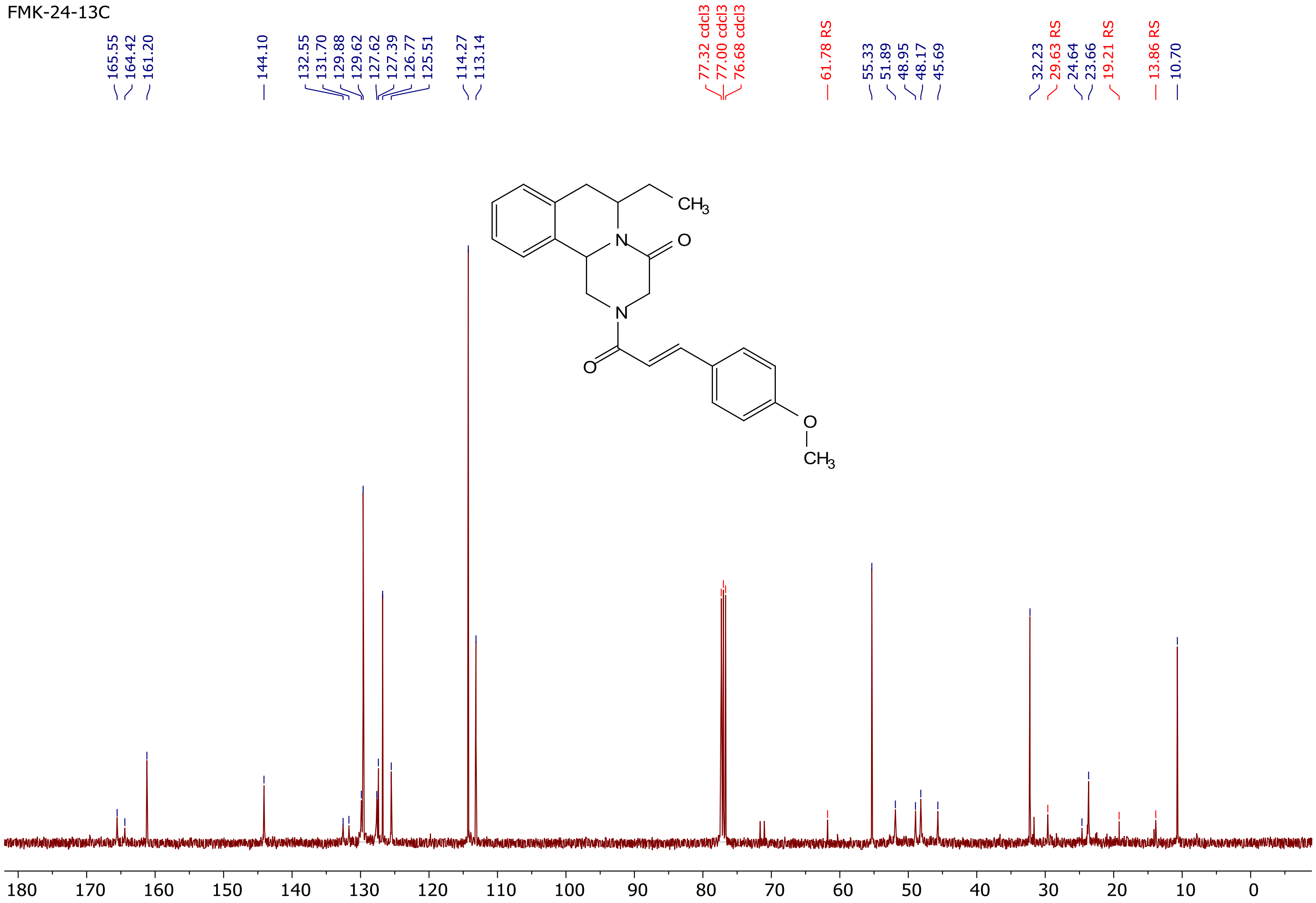

^{13}C-NMR spectrum of 2-[(*2E*)-3-(4-Methoxy-phenyl)-acryloyl]-6-ethyl-1,2,3,6,7,11b-hexahydro-pyrazino[2,1-a]isoquinolin-4-one **38**

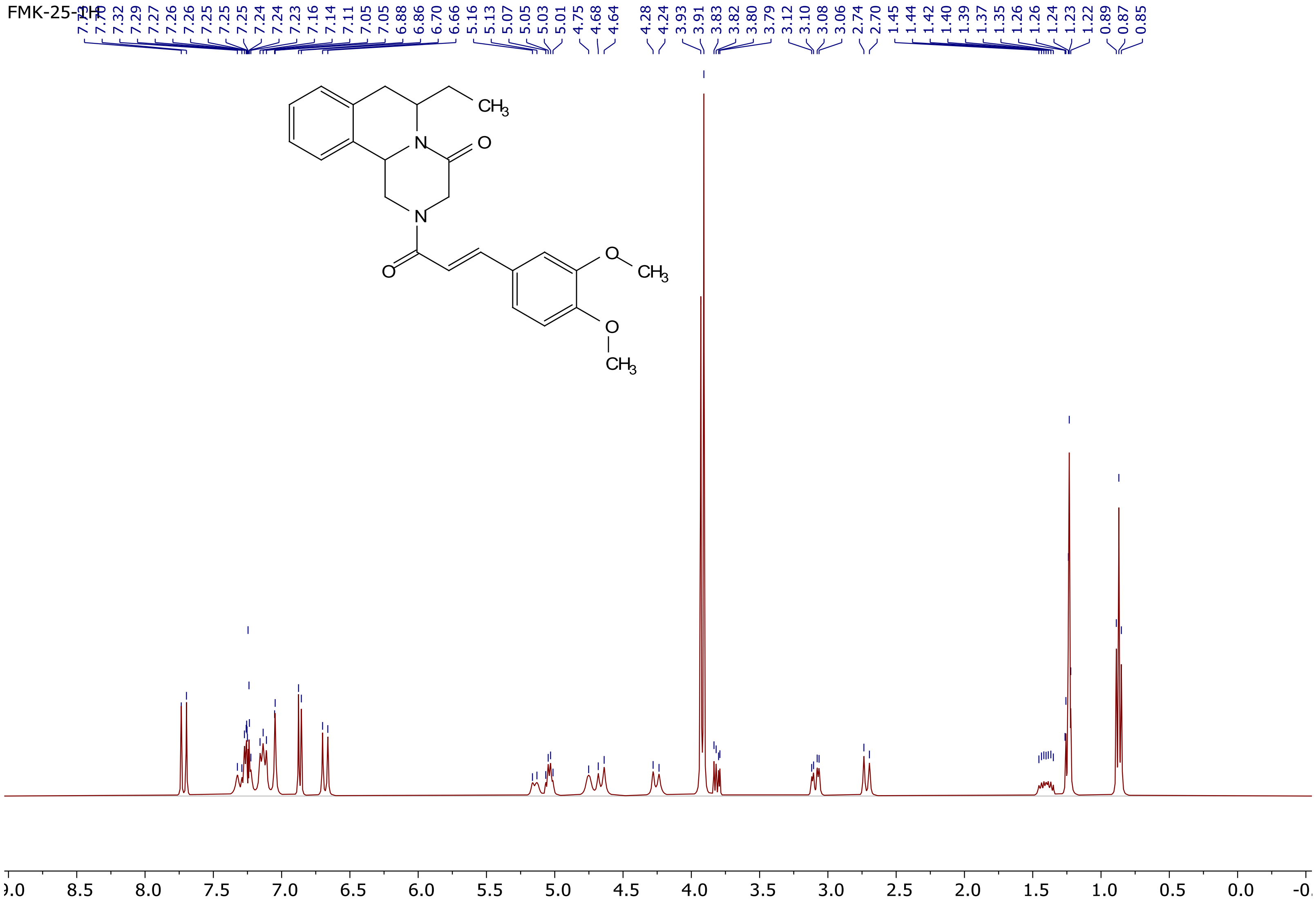

^{1}H-NMR spectrum of 2-[(*2E*)-3-(3,4-Dimethoxy-phenyl)-acryloyl]-6-ethyl-1,2,3,6,7,11b-hexahydro-pyrazino[2,1-a]isoquinolin-4-one **39**

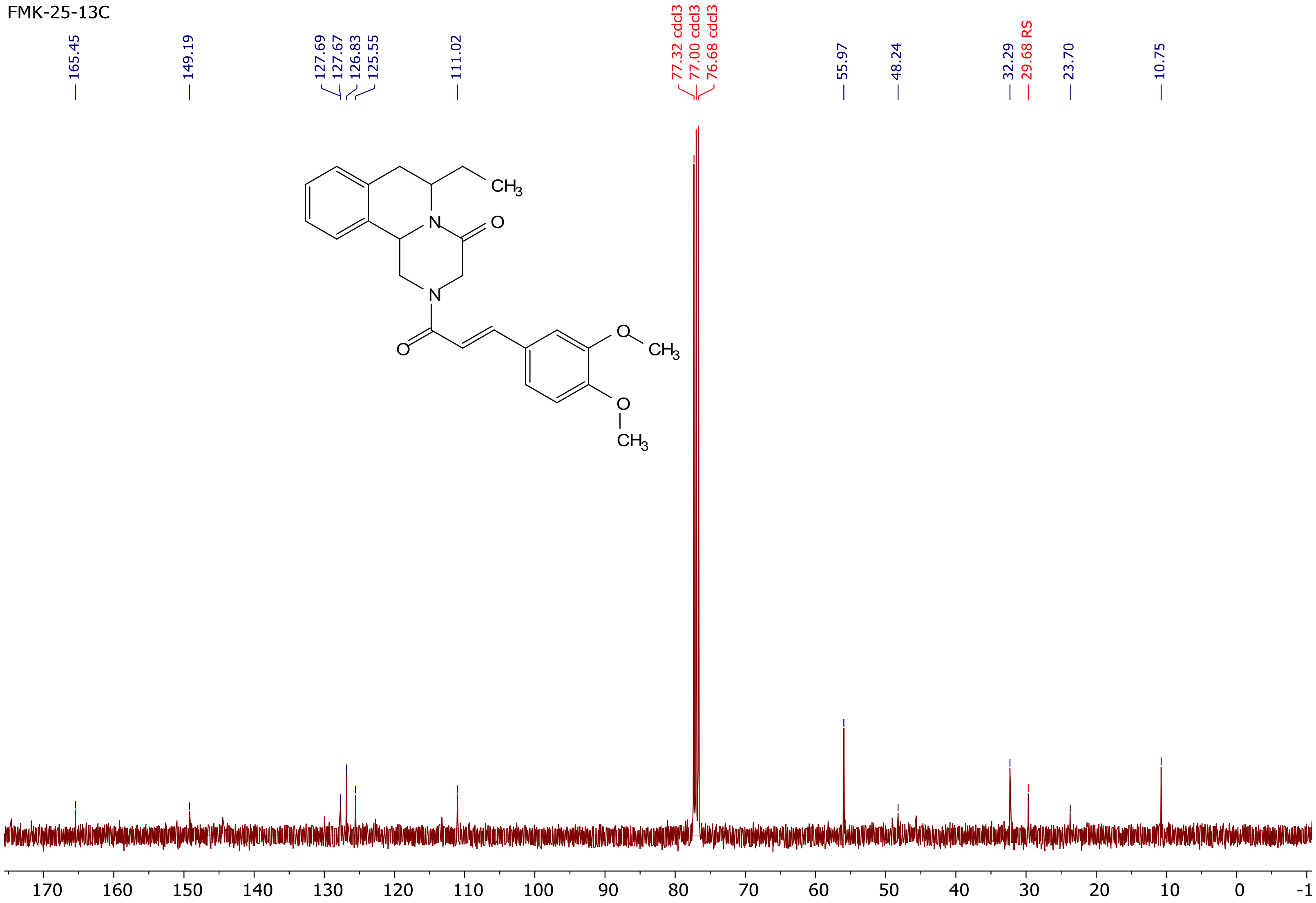

^{13}C-NMR spectrum of 2-[(*2E*)-3-(3,4-Dimethoxy-phenyl)-acryloyl]-6-ethyl-1,2,3,6,7,11b-hexahydro-pyrazino[2,1-a]isoquinolin-4-one **39**

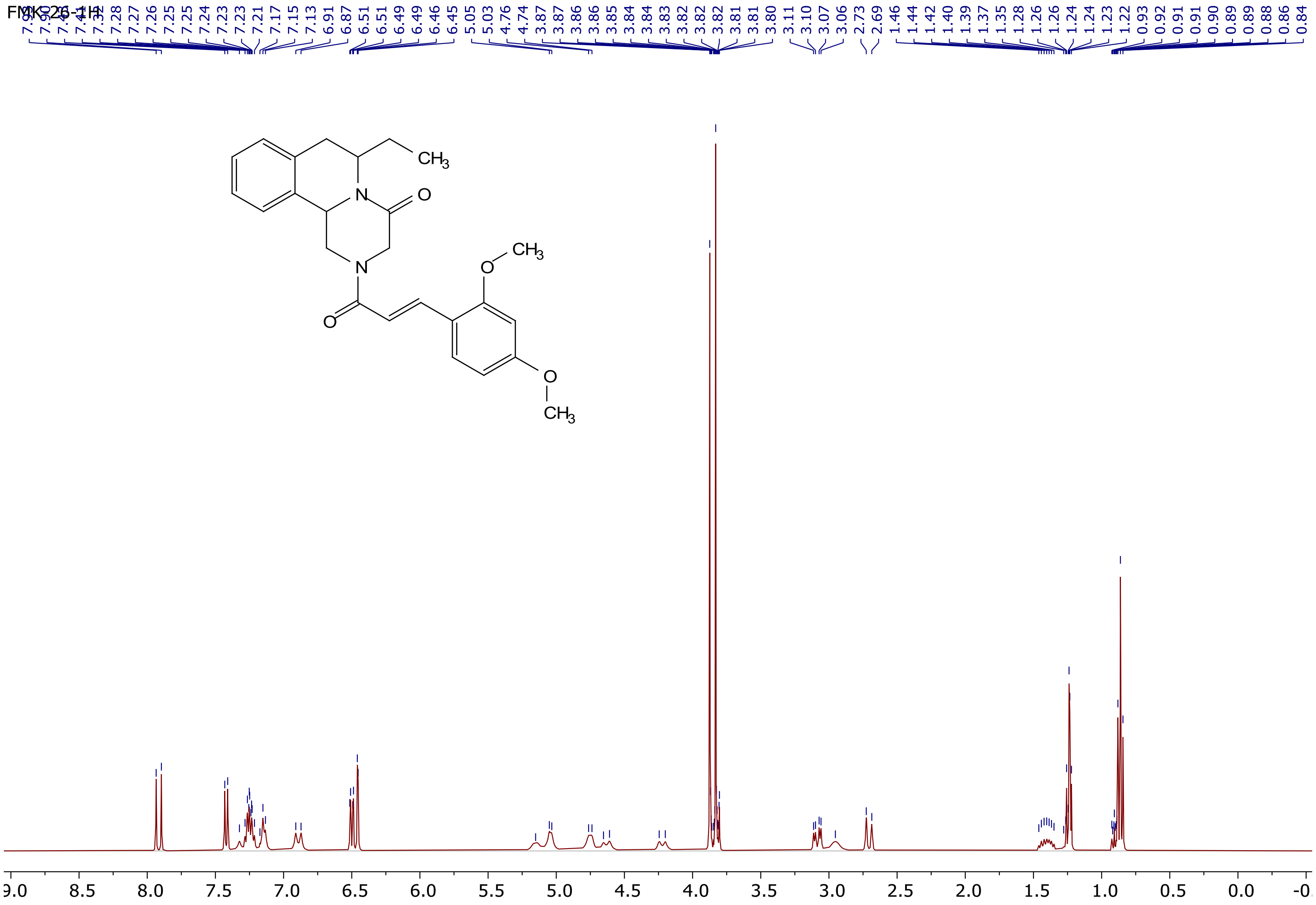

^{1}H-NMR spectrum of 2-[(2*E*)-3-(2,4-Dimethoxy-phenyl)-acryloyl]-6-ethyl-1,2,3,6,7,11b-hexahydro-pyrazino[2,1-a]isoquinolin-4-one **40**

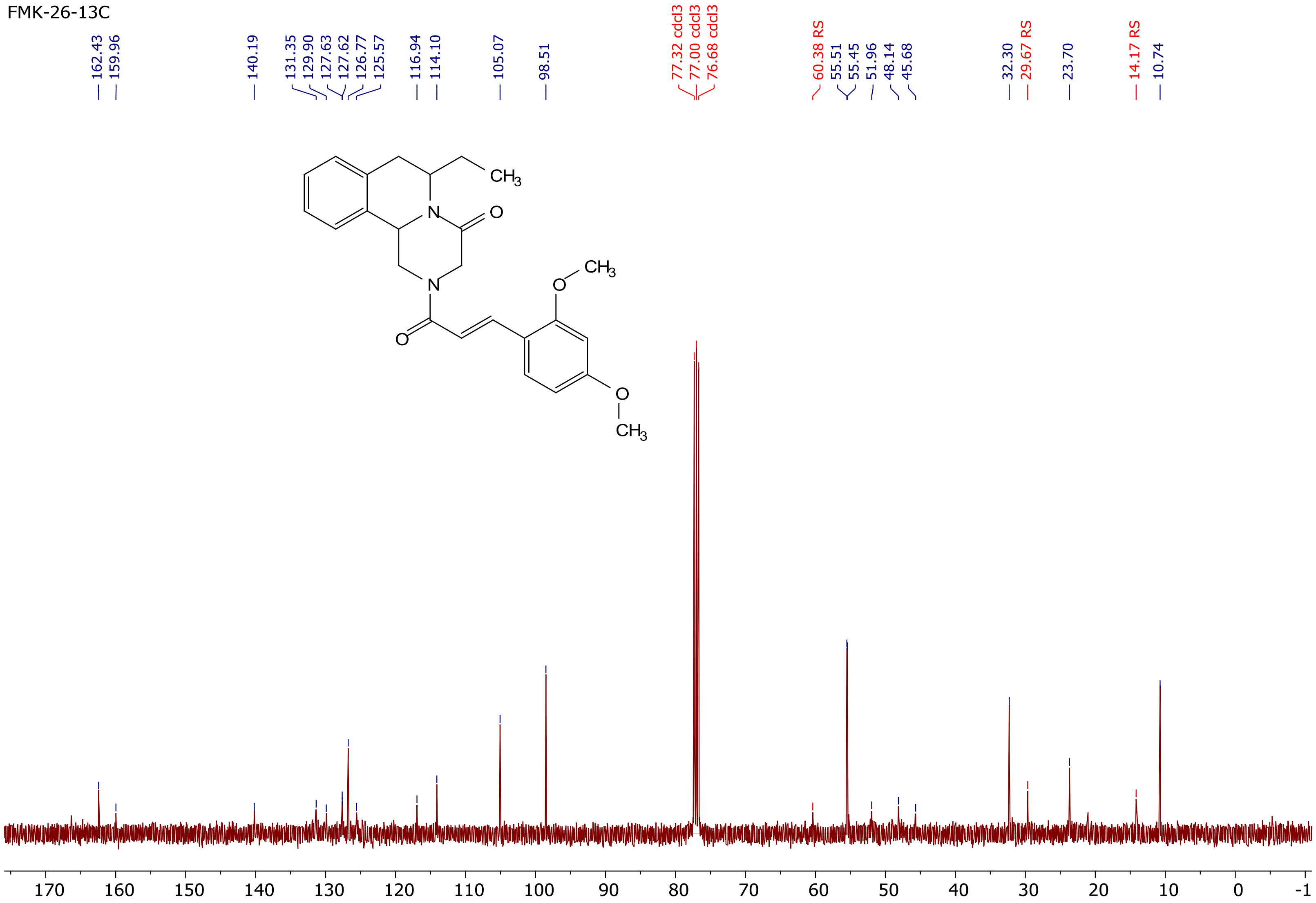

^{13}C-NMR spectrum of 2-[(*2E*)-3-(2,4-Dimethoxy-phenyl)-acryloyl]-6-ethyl-1,2,3,6,7,11b-hexahydro-pyrazino[2,1-a]isoquinolin-4-one **40**

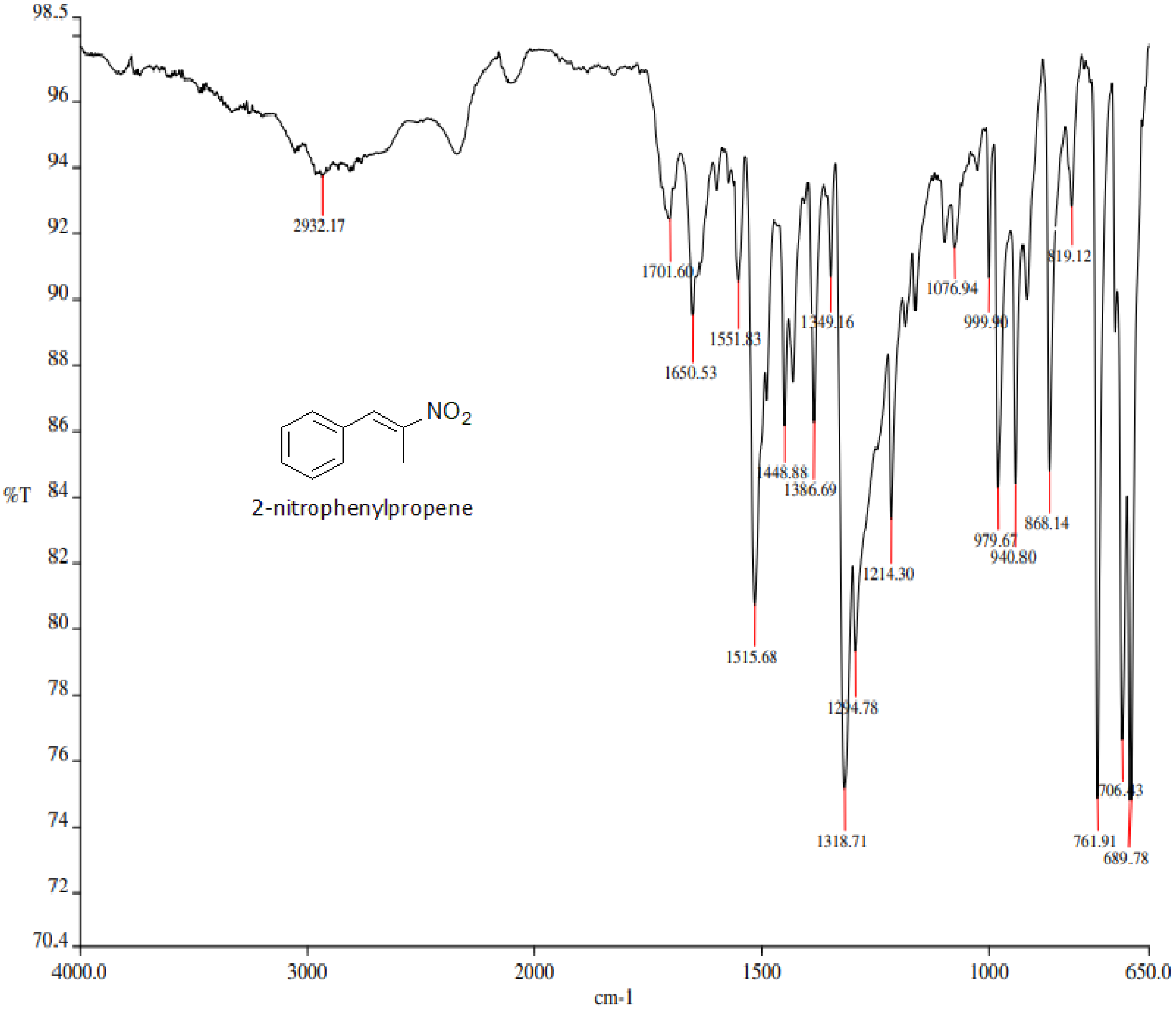

FT-IR Spectrum of trans-2-nitrophenylpropene **16a**

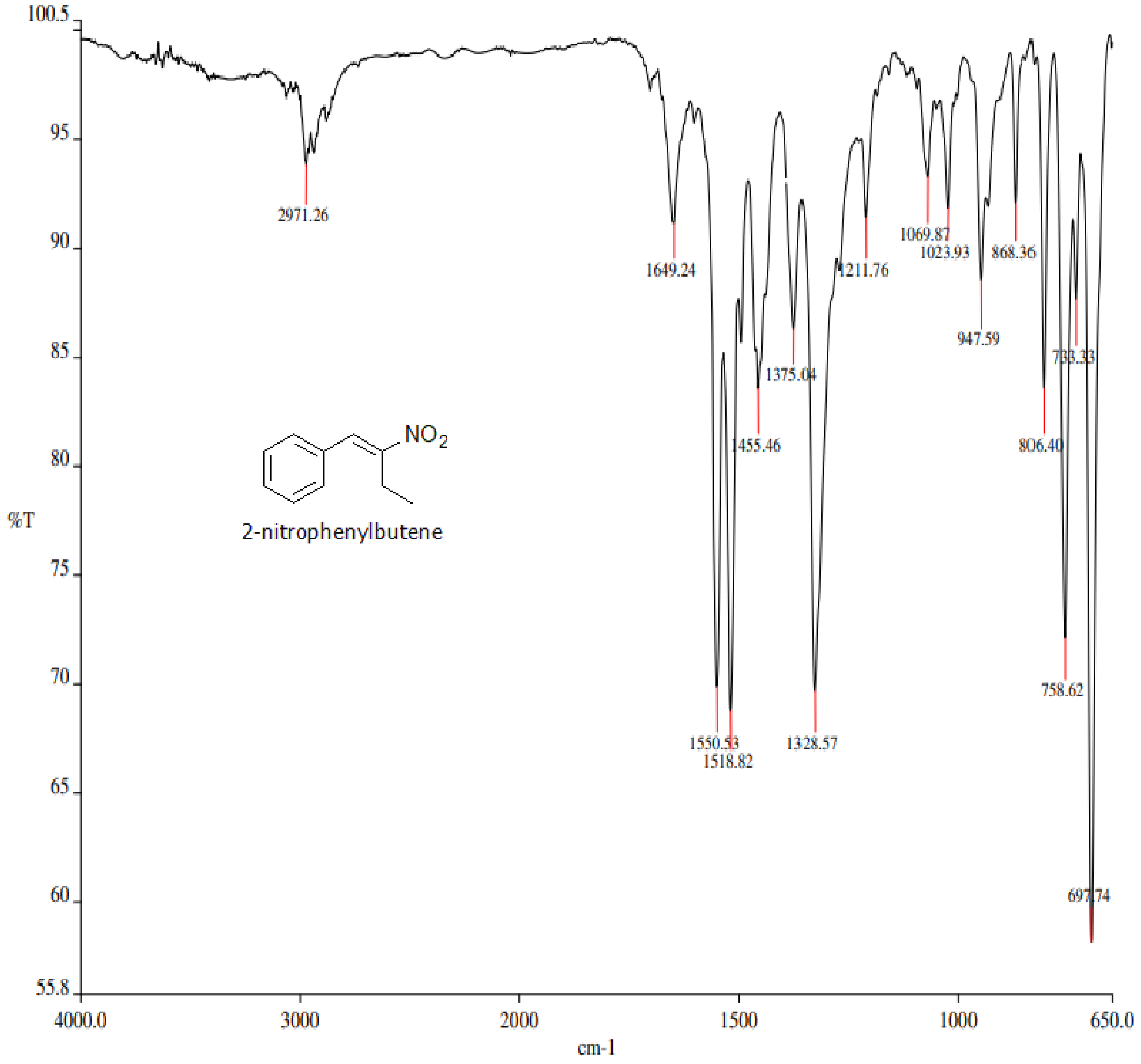

FT-IR Spectrum of trans-2-nitrophenylbutene **16b**

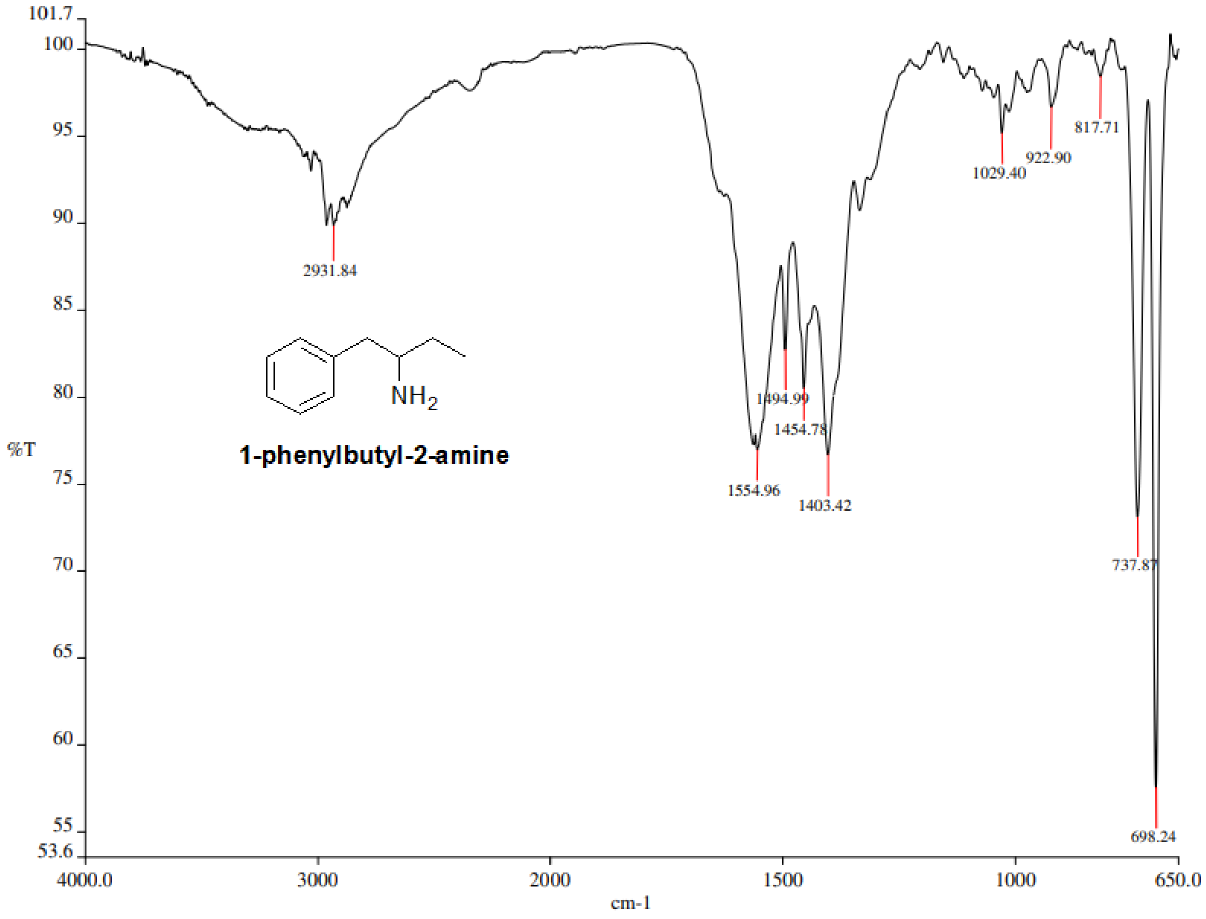

FT-IR Spectrum of 1-phenylbutyl-2-amine **17b**

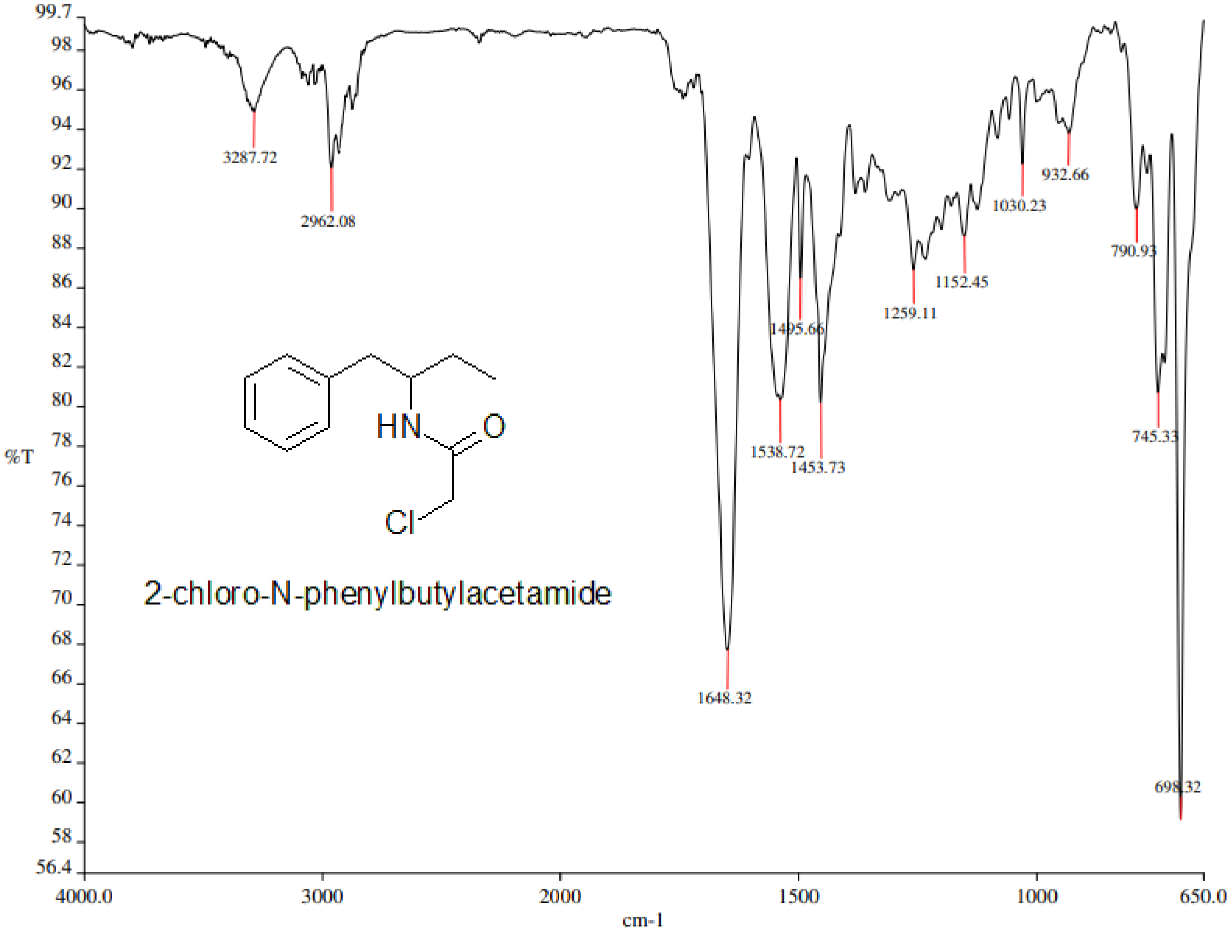

FT-IR Spectrum of 2-chloro-N-(1-phenylbutan-2-yl)acetamide **19bd**

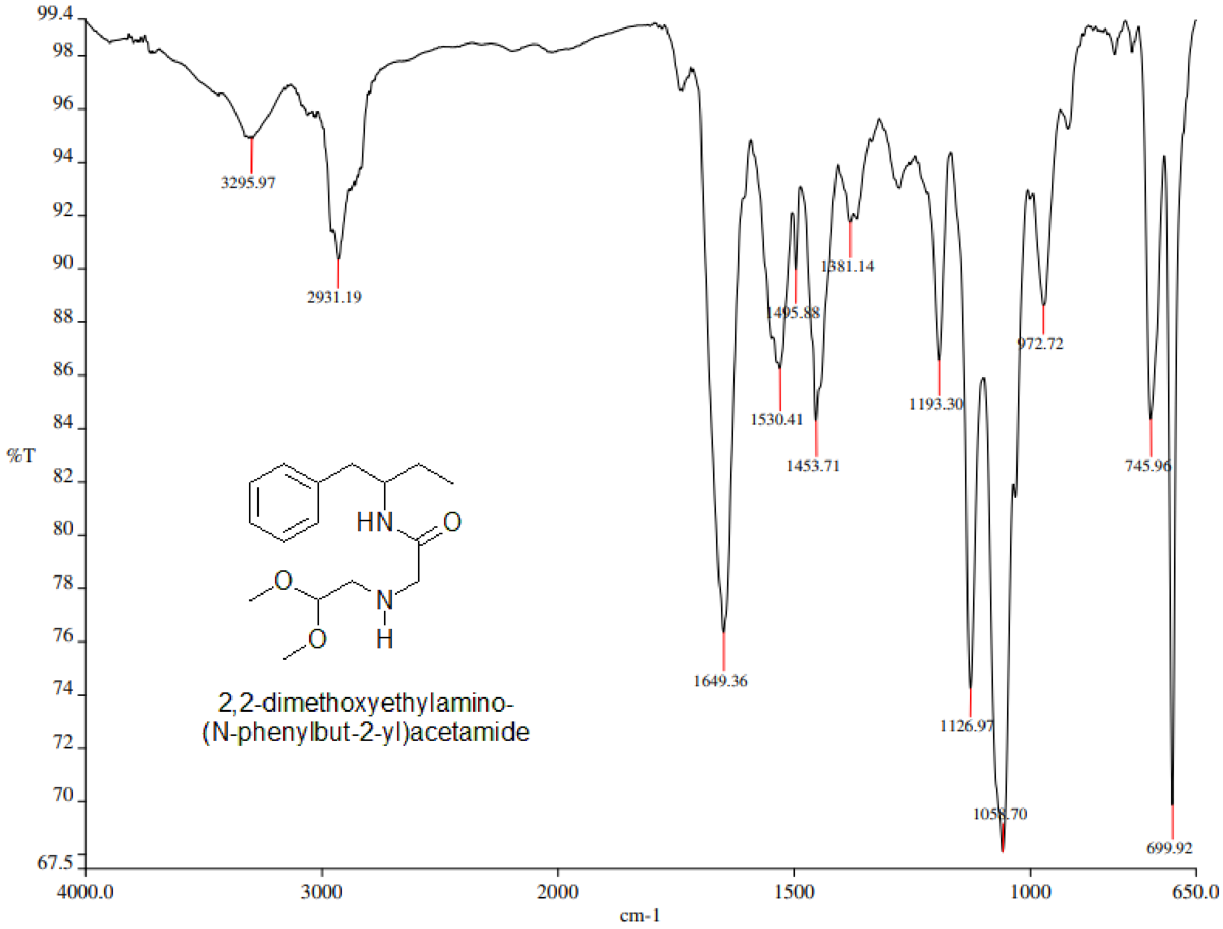

FT-IR Spectrum of 2,2-dimethoxyethylamino-N-(2-phenylbut-2-yl)acetamide **20b**

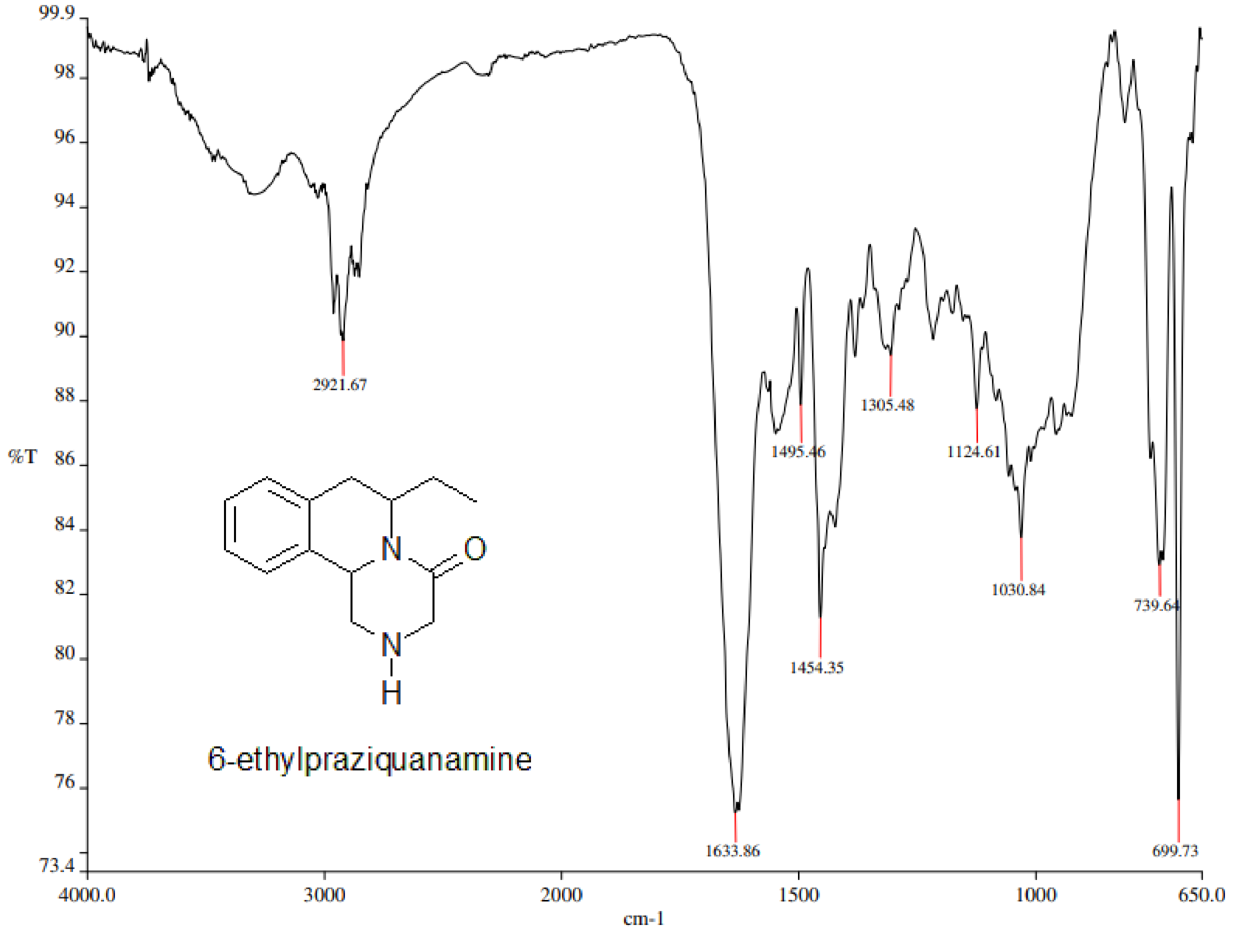

FT-IR Spectrum of 6-Ethylpraziquanamine **21b**

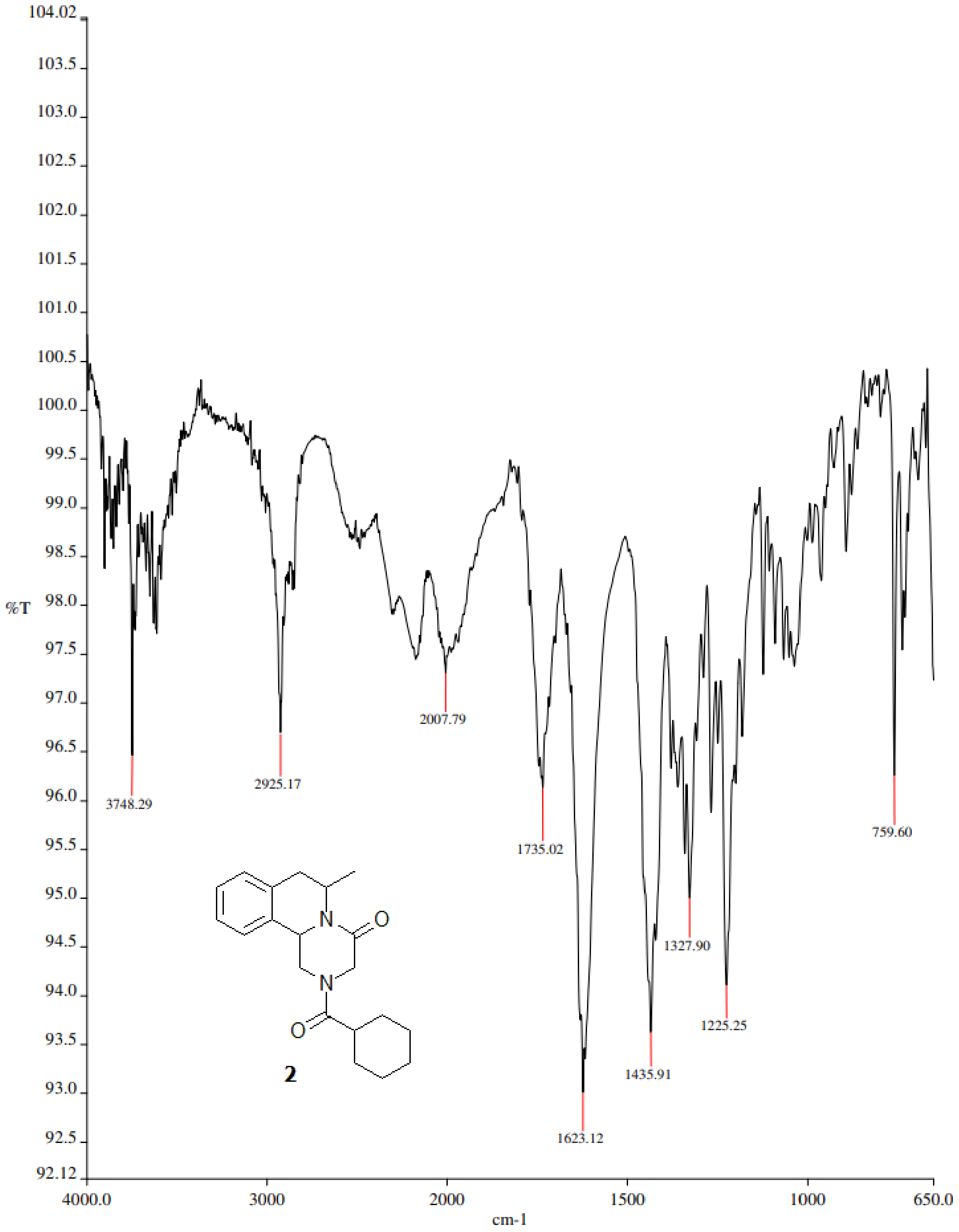

FT-IR spectrum of 2-(Cyclohexylcarbonyl)-6-methyl-1,2,3,6,7,11b-hexahydro-4H-pyrazino[2,1-a]isoquinolin-4-one, 6-methylpraziquantel **22**

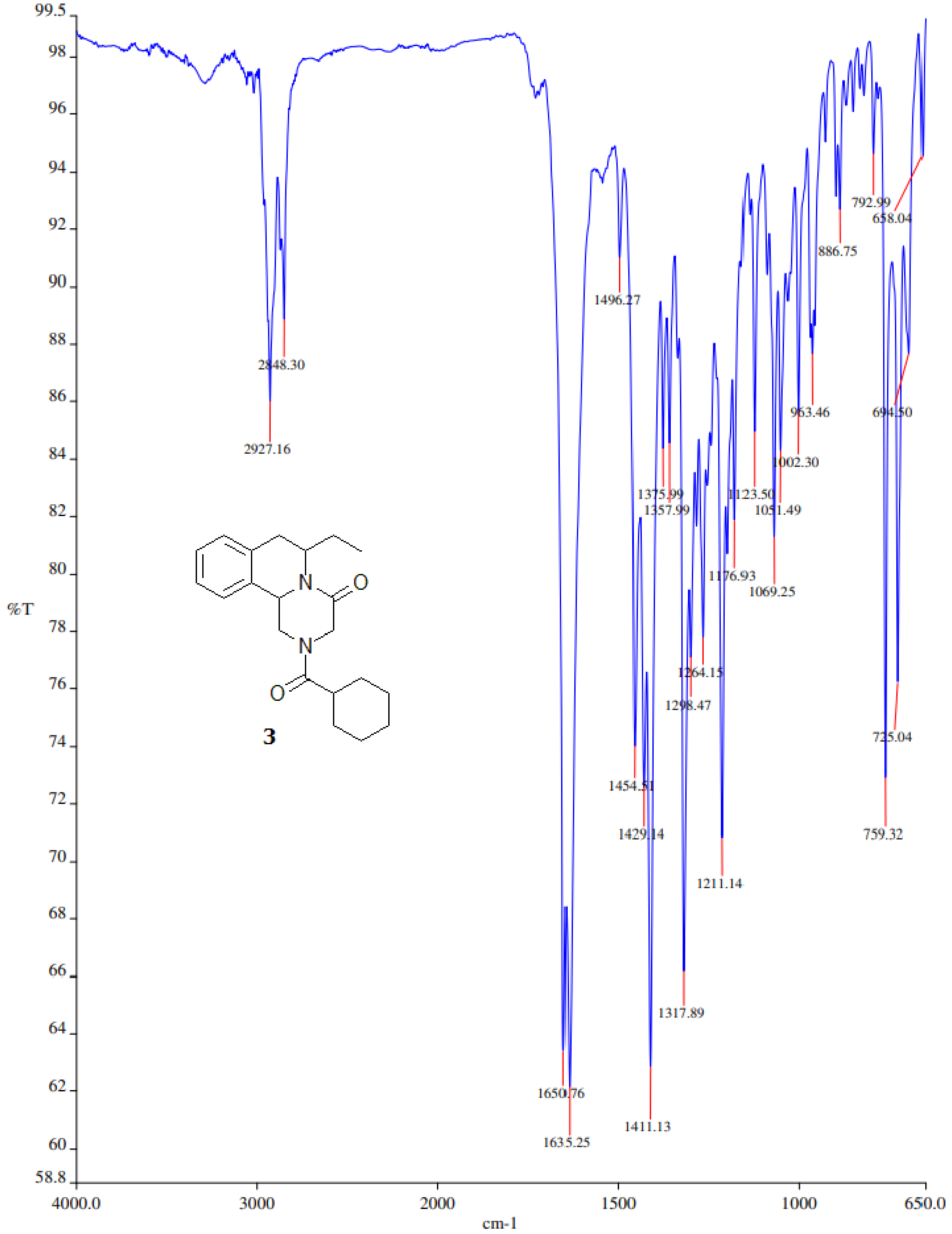

FT-IR spectrum of 2-Cyclohexanecarbonyl-6-ethyl-1,2,3,6,7,11b-hexahydro-pyrazino[2,1-a]isoquinolin-4-one, 6-ethylpraziquantel **23**

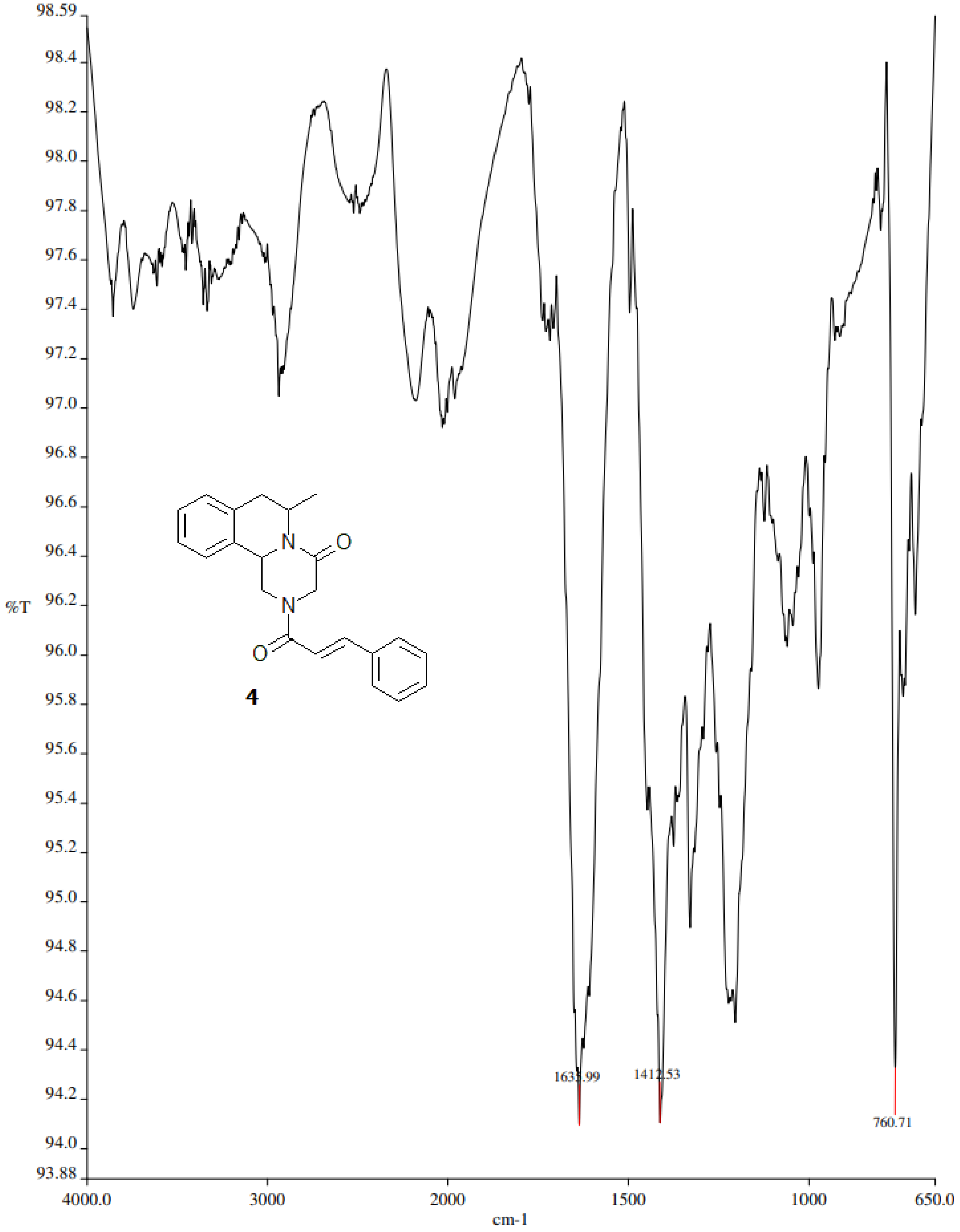

FT-IR spectrum of 6-Methyl-2-[(*2E*)-3-phenylprop-2-enoyl]-1,2,3,6,7,11b-hexahydro-4H-pyrazino[2,1-a]isoquinolin-4-one **24**

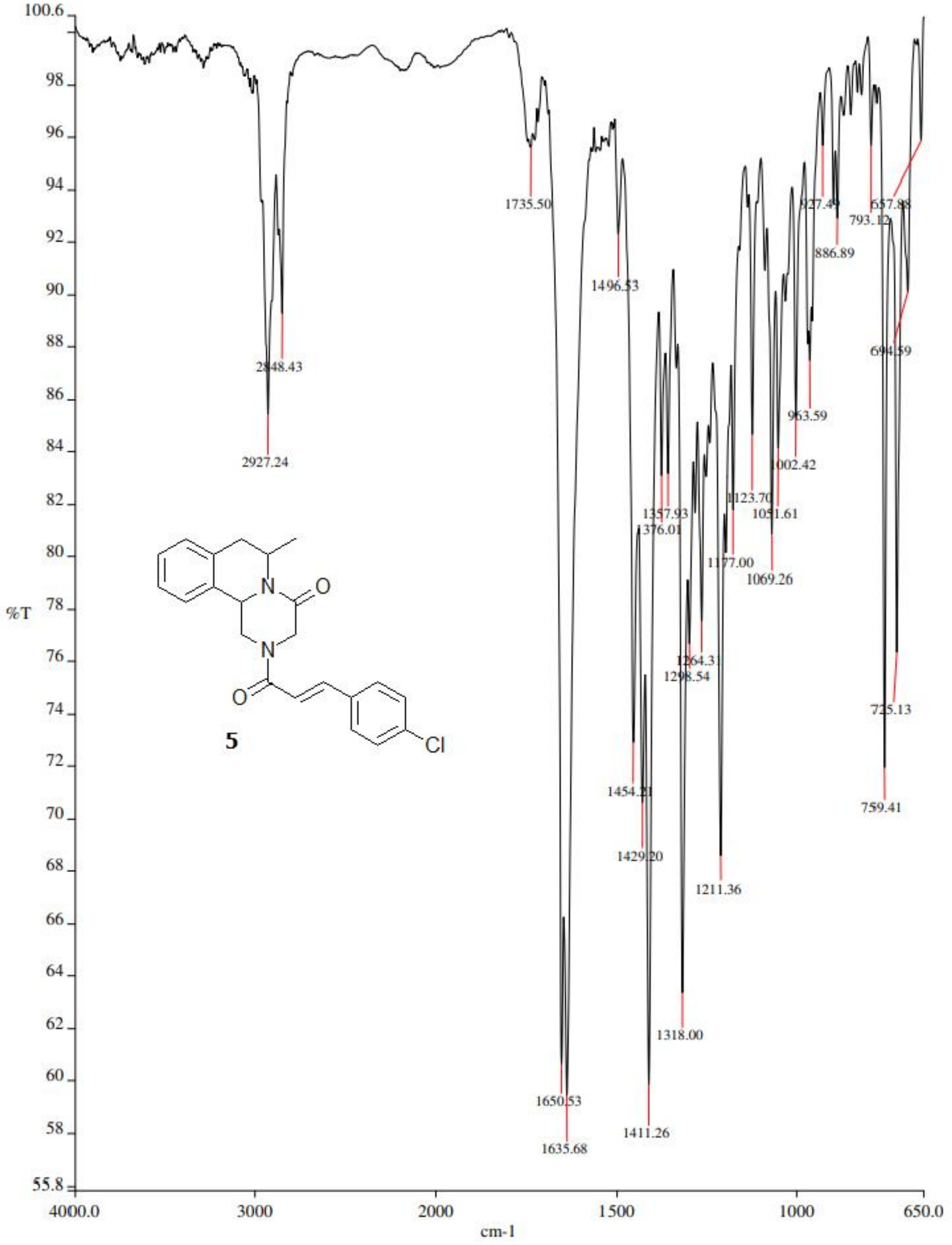

FT-IR spectrum of 2-[(*2E*)-3-(4-Chlorophenyl)prop-2-enoyl]-6-methyl-1,2,3,6,7,11b-hexahydro-4H-pyrazino[2,1-a]isoquinolin-4-one **25**

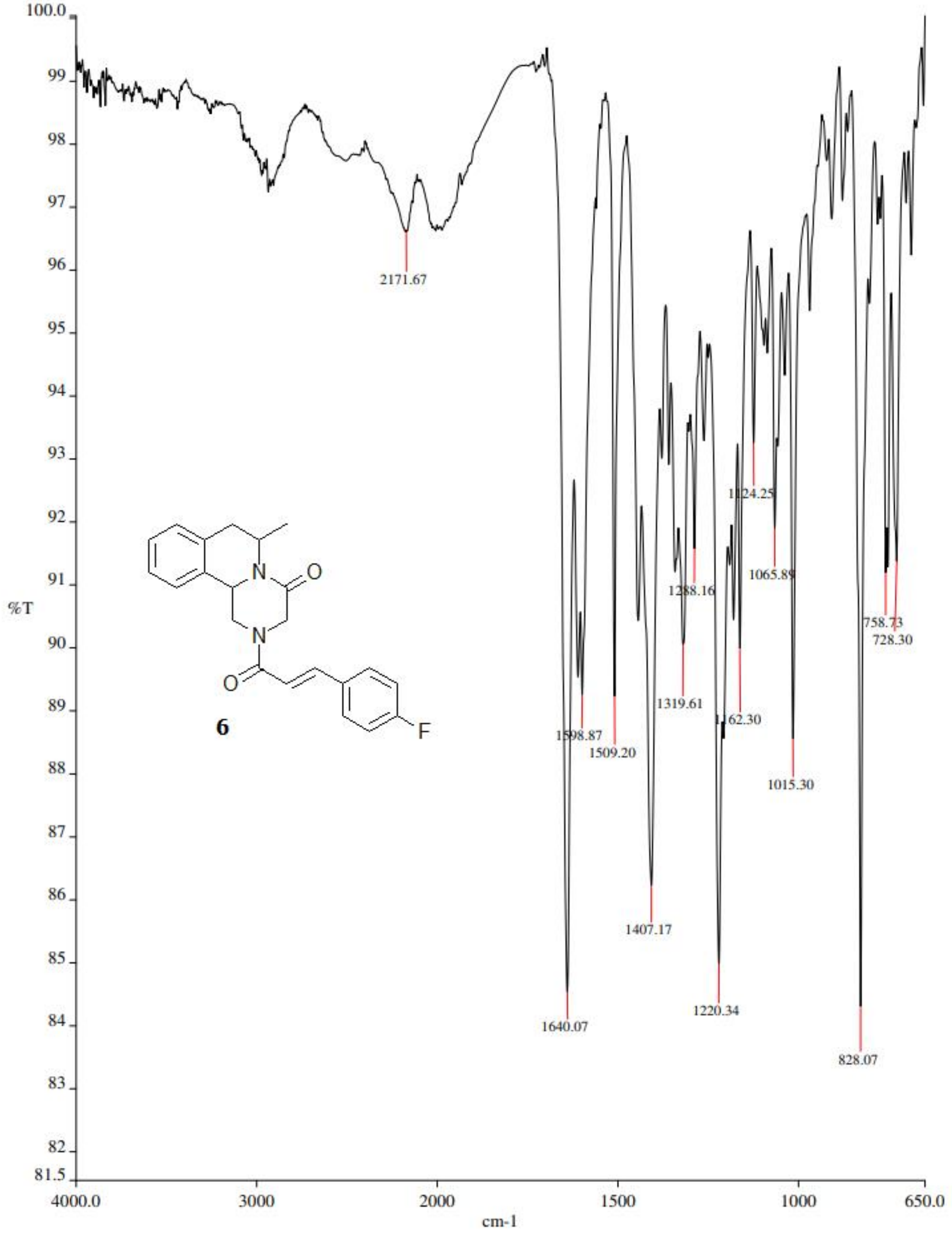

FT-IR spectrum of 2-[(*2E*)-3-(4-Fluorophenyl)prop-2-enoyl]-6-methyl-1,2,3,6,7,11b-hexahydro-4H-pyrazino[2,1-a]isoquinolin-4-one **26**

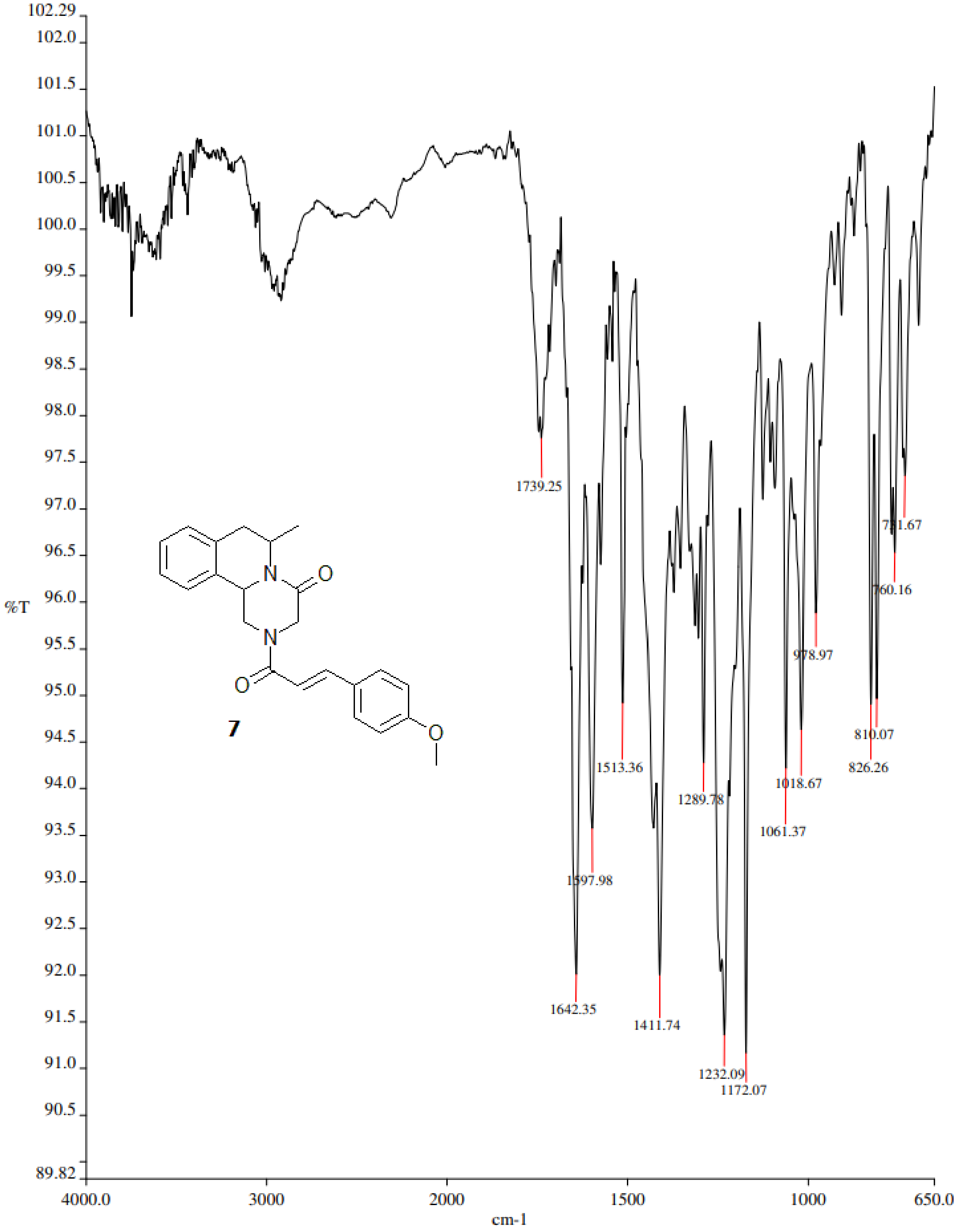

FT-IR spectrum of 2-[(*2E*)-3-(4-Methoxy-phenyl)prop-2-enoyl]-6-methyl-1,2,3,6,7,11b-hexahydro-pyrido[2,1-a]isoquinolin-4-one **27**

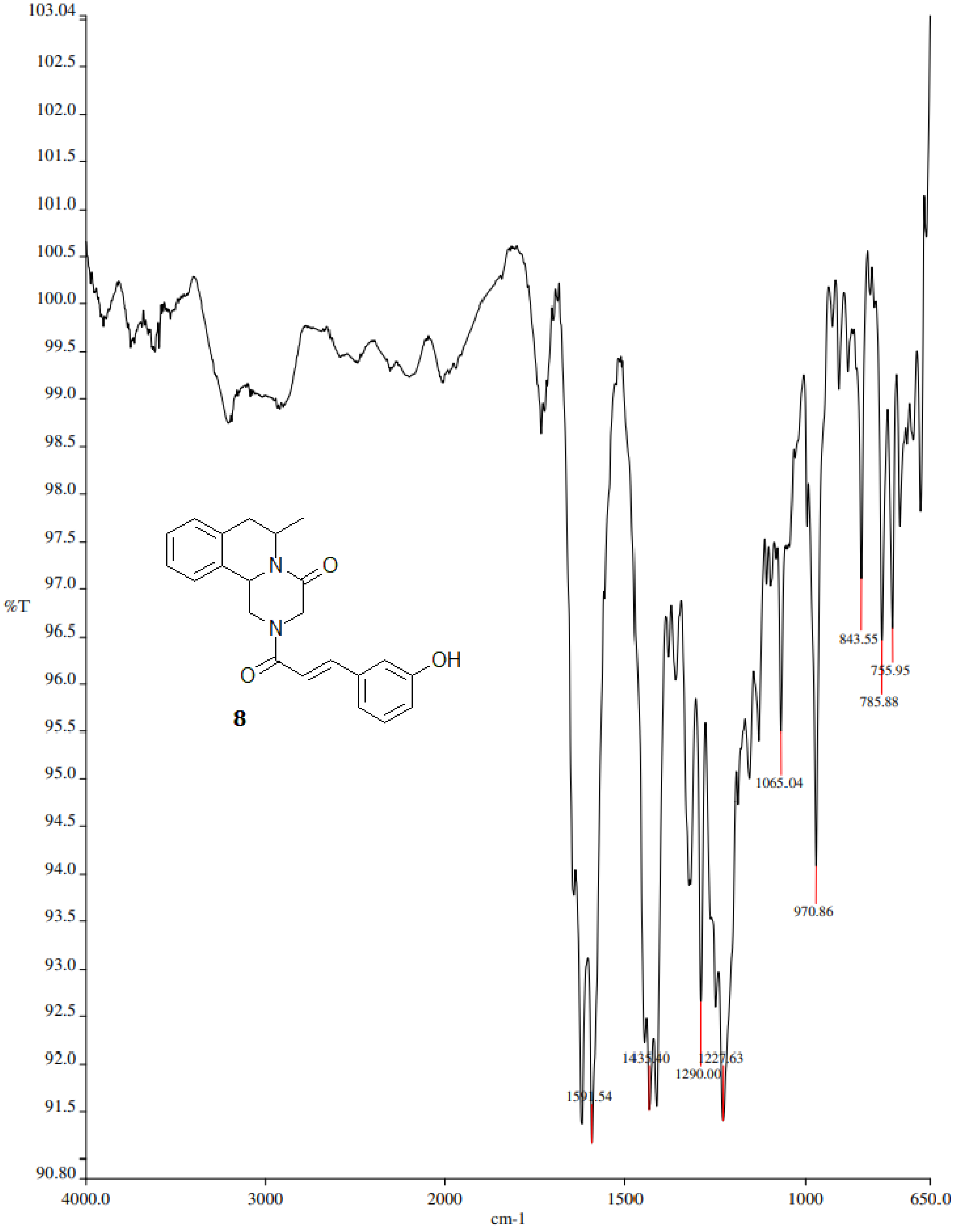

FT-IR spectrum of 2-[(*2E*)-3-(3-Hydroxyphenyl)prop-2-enoyl]-6-methyl-1,2,3,6,7,11b-hexahydro-4H-pyrazino[2,1-a]isoquinolin-4-one **28**

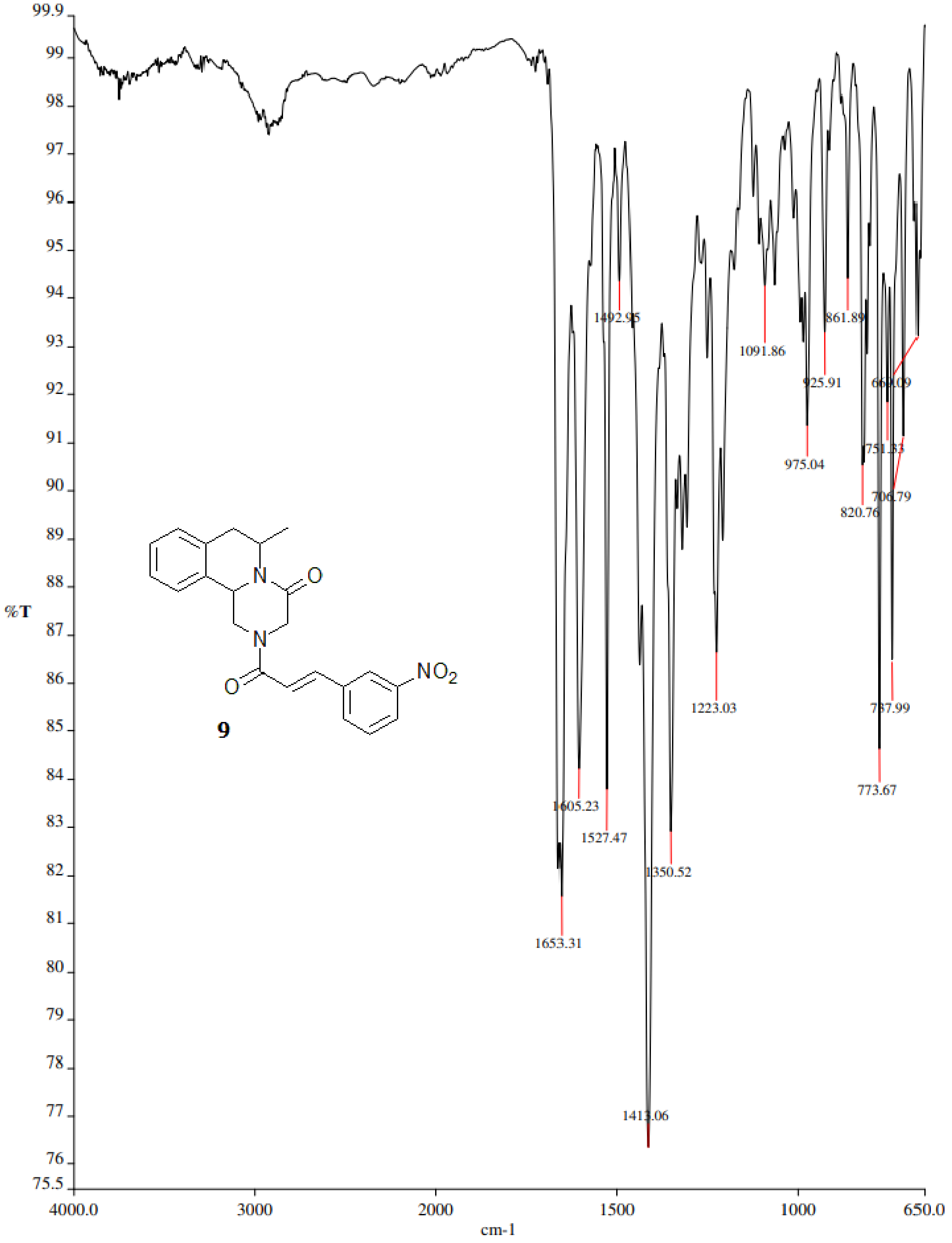

FT-IR spectrum of 2-[(*2E*)-3-(3-Nitrophenyl)prop-2-enoyl]- 6-methyl-1,2,3,6,7,11b-hexahydro-4H-pyrazino[2,1-a]isoquinolin-4-one **29**

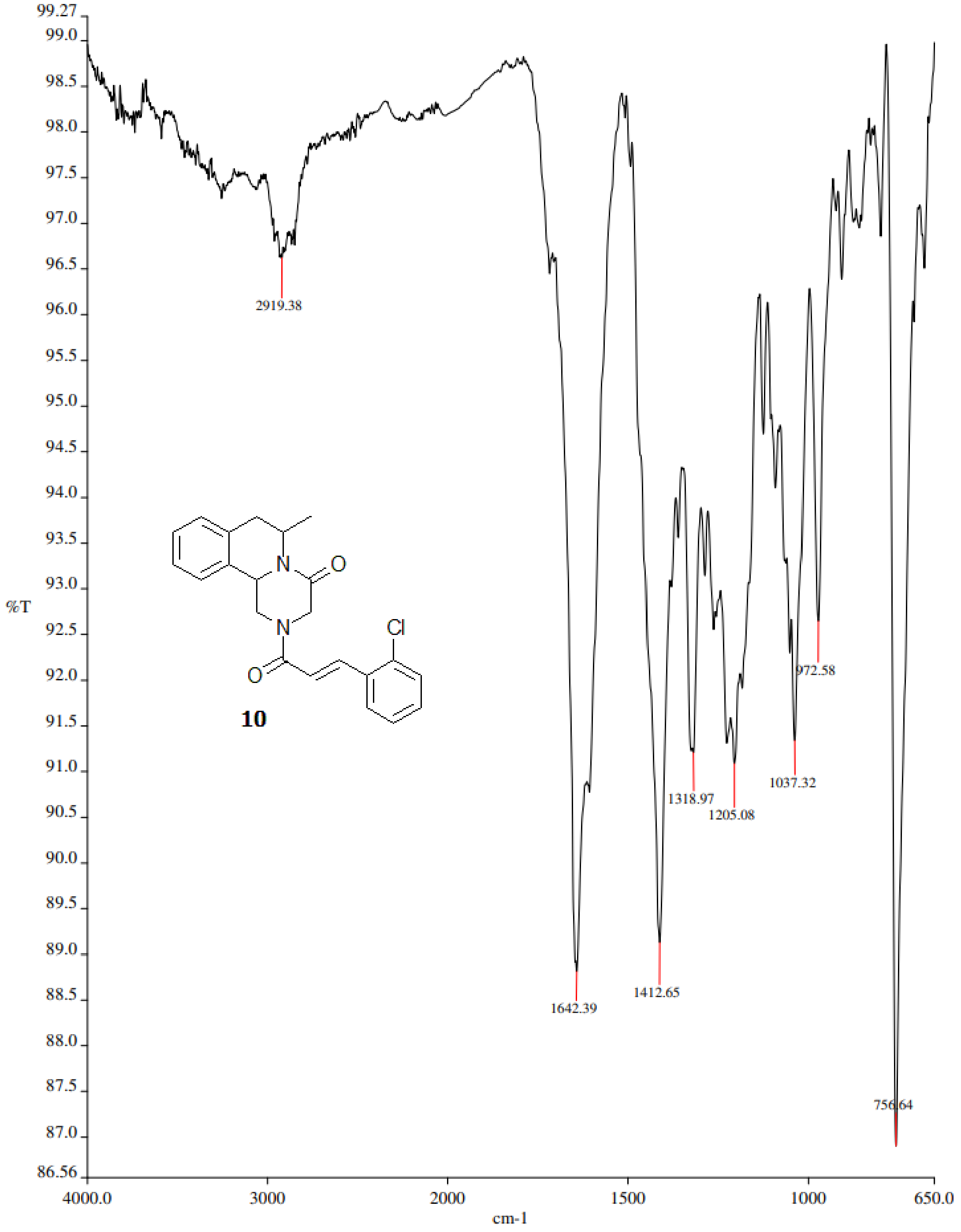

FT-IR spectrum of 2-[(*2E*)-3-(2-Chlorophenyl)prop-2-enoyl]-6-methyl-1,2,3,6,7,11b-hexahydro-4H-pyrazino[2,1-a]isoquinolin-4-one **30**

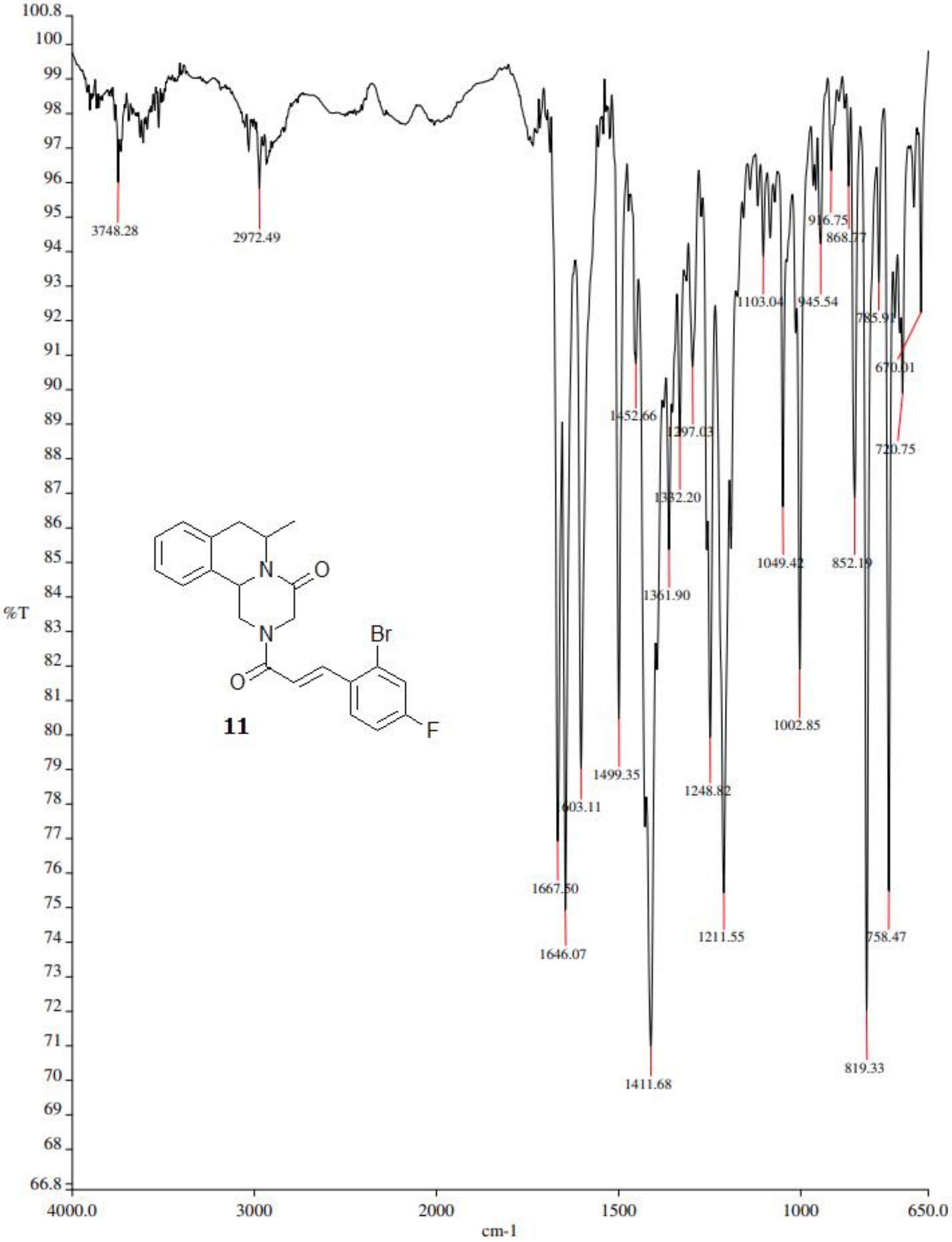

FT-IR spectrum of 2-[(*2E*)-3-(2-Bromo-4-fluorophenyl)prop-2-enoyl]-6-methyl-1,2,3,6,7,11b-hexahydro-4H-pyrazino[2,1-a]isoquinolin-4-one **31**

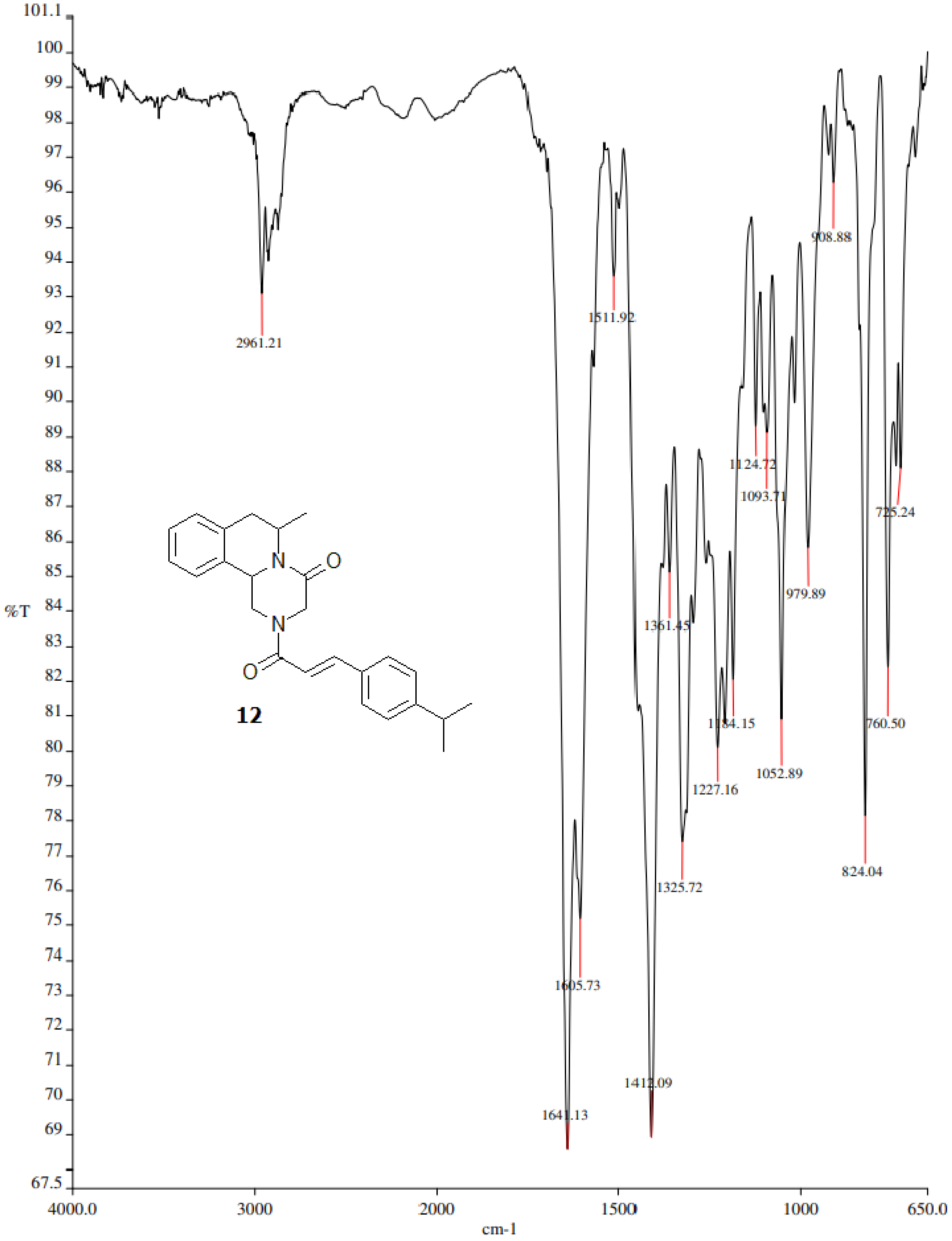

FT-IR spectrum of 2-[(2*E*)-3-(2-Bromo-4-fluorophenyl)prop-2-enoyl]-6-methyl-1,2,3,6,7,11b-hexahydro-4H-pyrazino[2,1-a]isoquinolin-4-one **32**

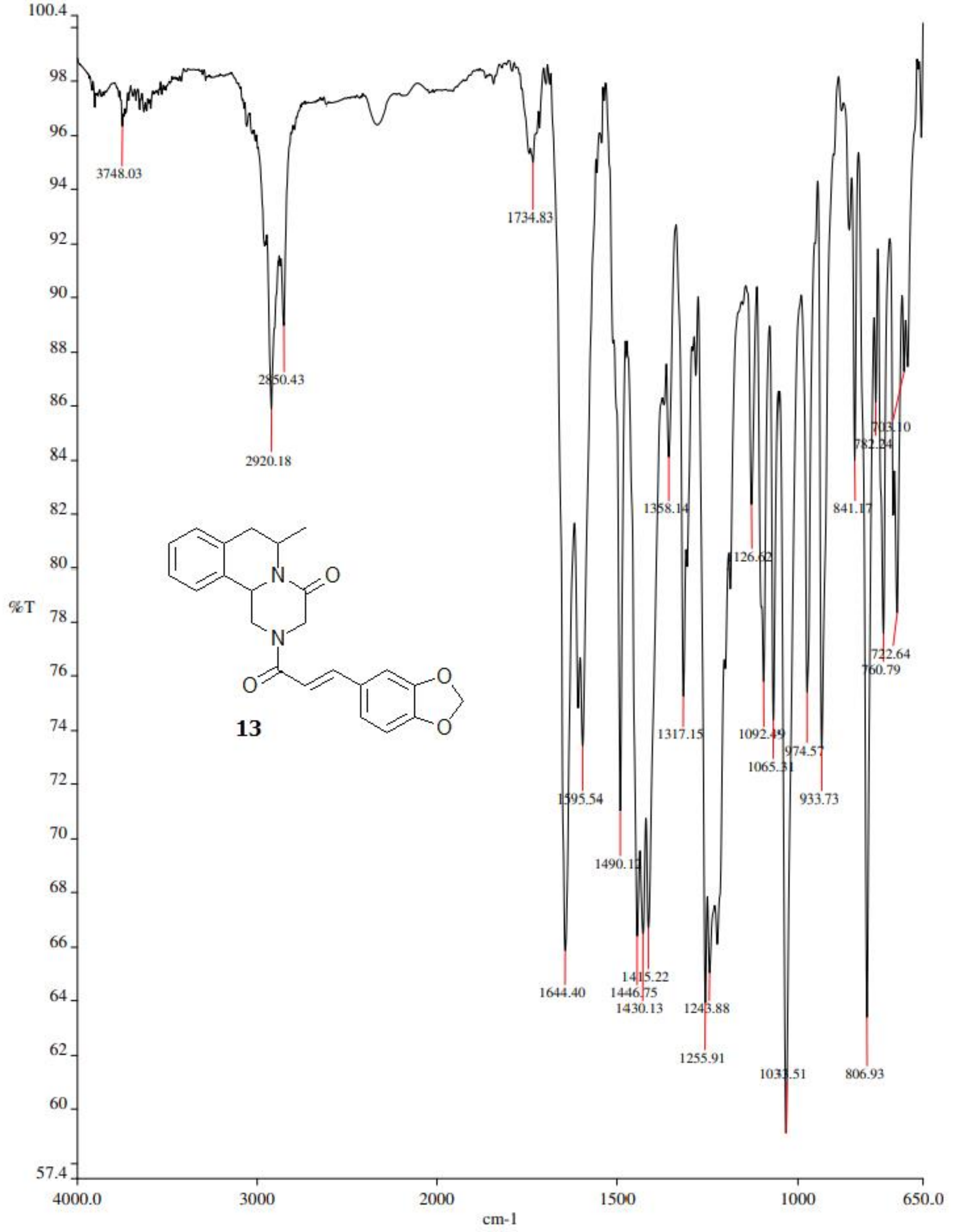

FT-IR spectrum of 6-Ethyl-2-[(*2E*)-3-phenylprop-2-enoyl]-1,2,3,6,7,11b-hexahydro-4H-pyrazino[2,1-a]isoquinolin-4-one **33**

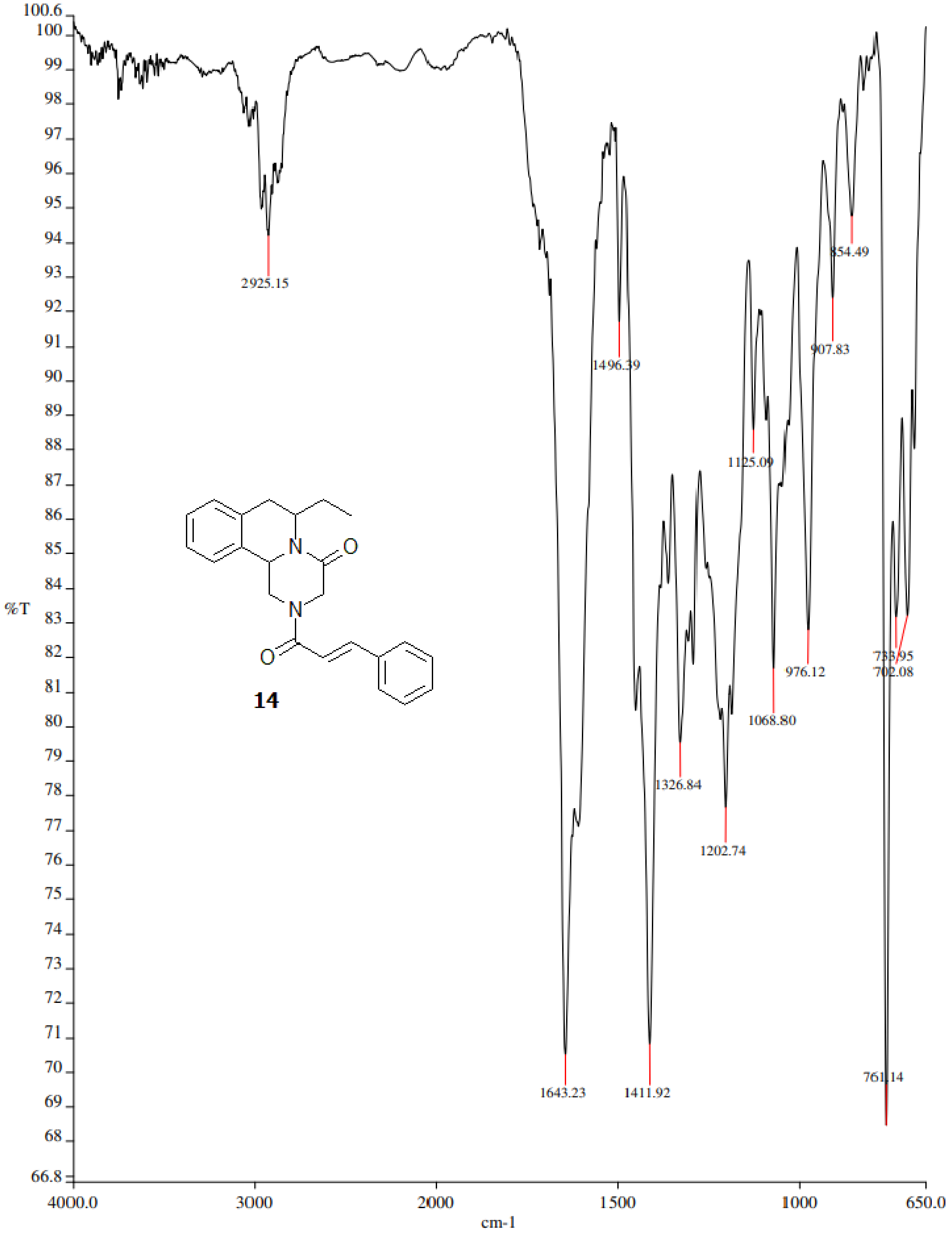

FT-IR spectrum of 6-Ethyl-2-[(*2E*)-3-phenylprop-2-enoyl]-1,2,3,6,7,11b-hexahydro-4H-pyrazino[2,1-a]isoquinolin-4-one **34**

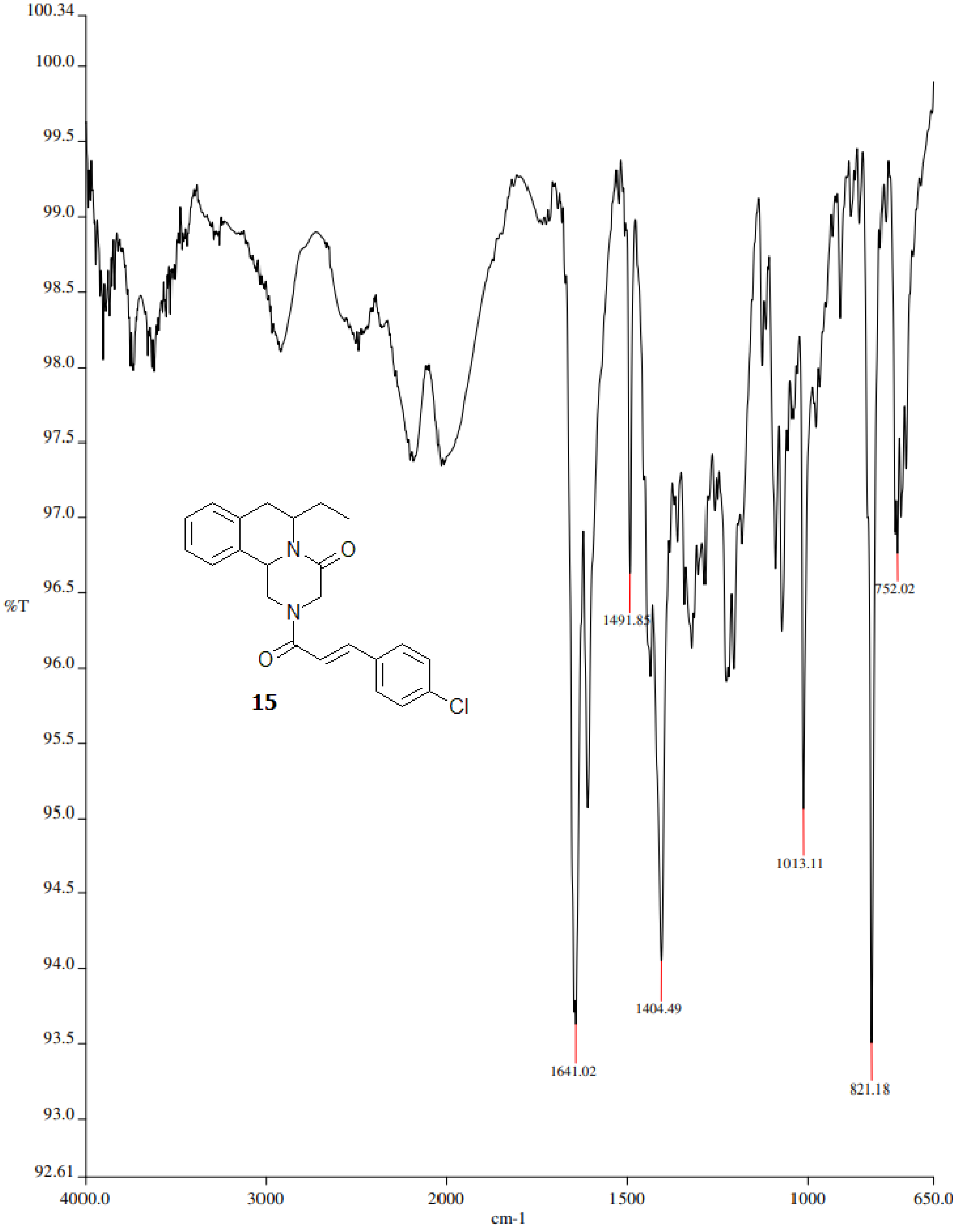

FT-IR spectrum of 6-Ethyl-2-[(2*E*)-3-phenylprop-2-enoyl]-1,2,3,6,7,11b-hexahydro-4H-pyrazino[2,1-a]isoquinolin-4-one **35**

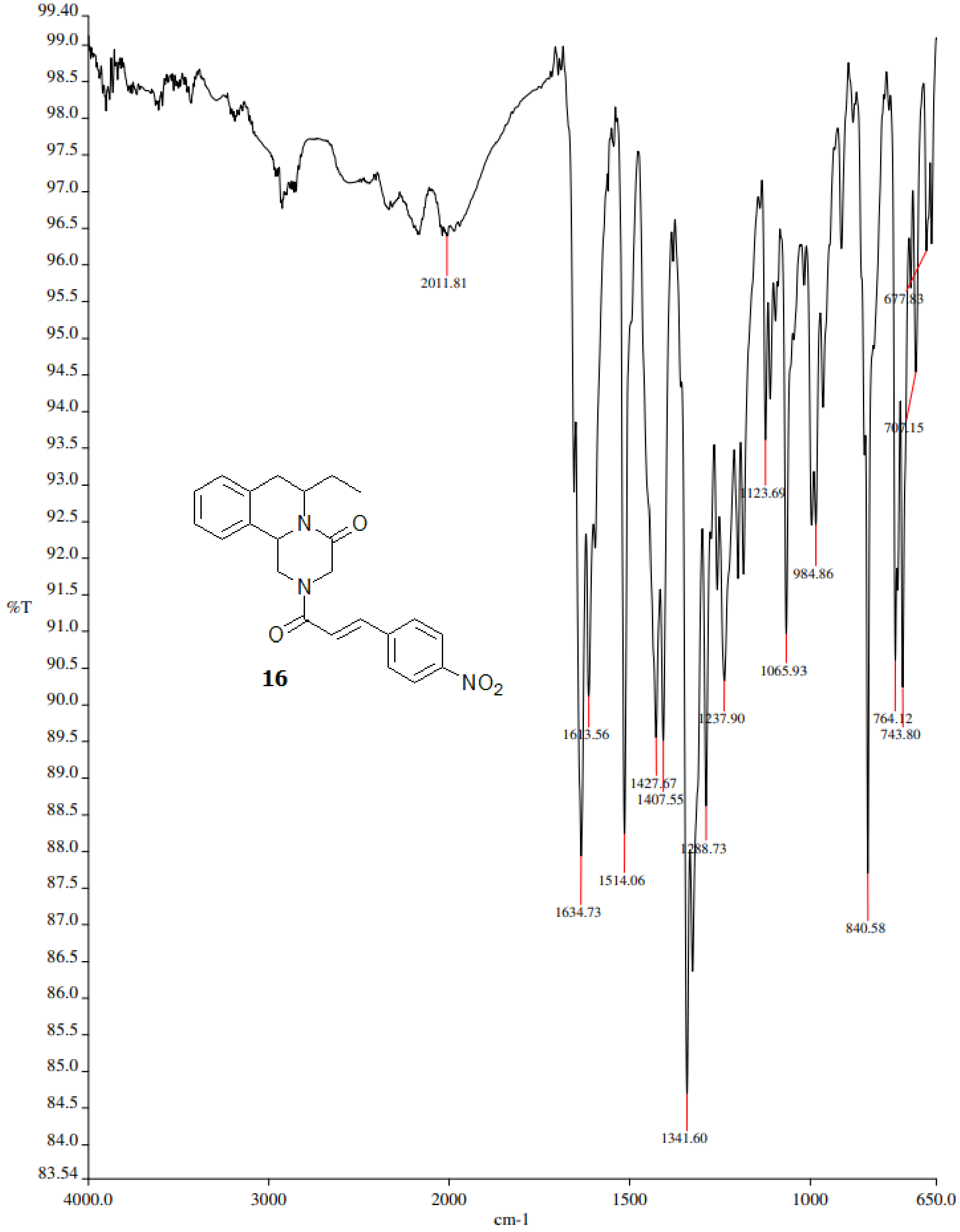

FT-IR spectrum of 6-Ethyl-2-[(*2E*)-3-phenylprop-2-enoyl]-1,2,3,6,7,11b-hexahydro-4H-pyrazino[2,1-a]isoquinolin-4-one **36**

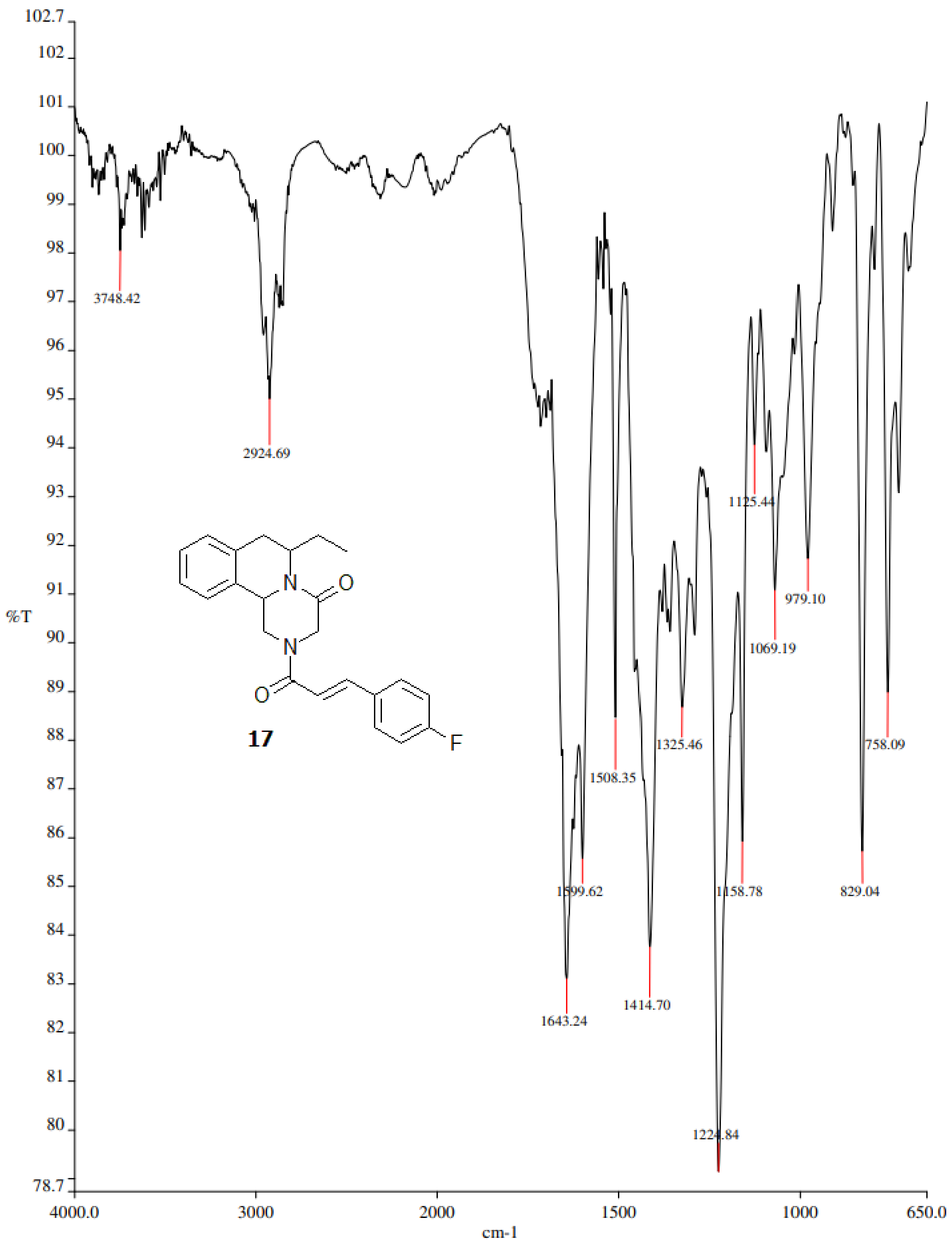

FT-IR spectrum of 6-Ethyl-2-[(2*E*)-3-phenylprop-2-enoyl]-1,2,3,6,7,11b-hexahydro-4H-pyrazino[2,1-a]isoquinolin-4-one **37**

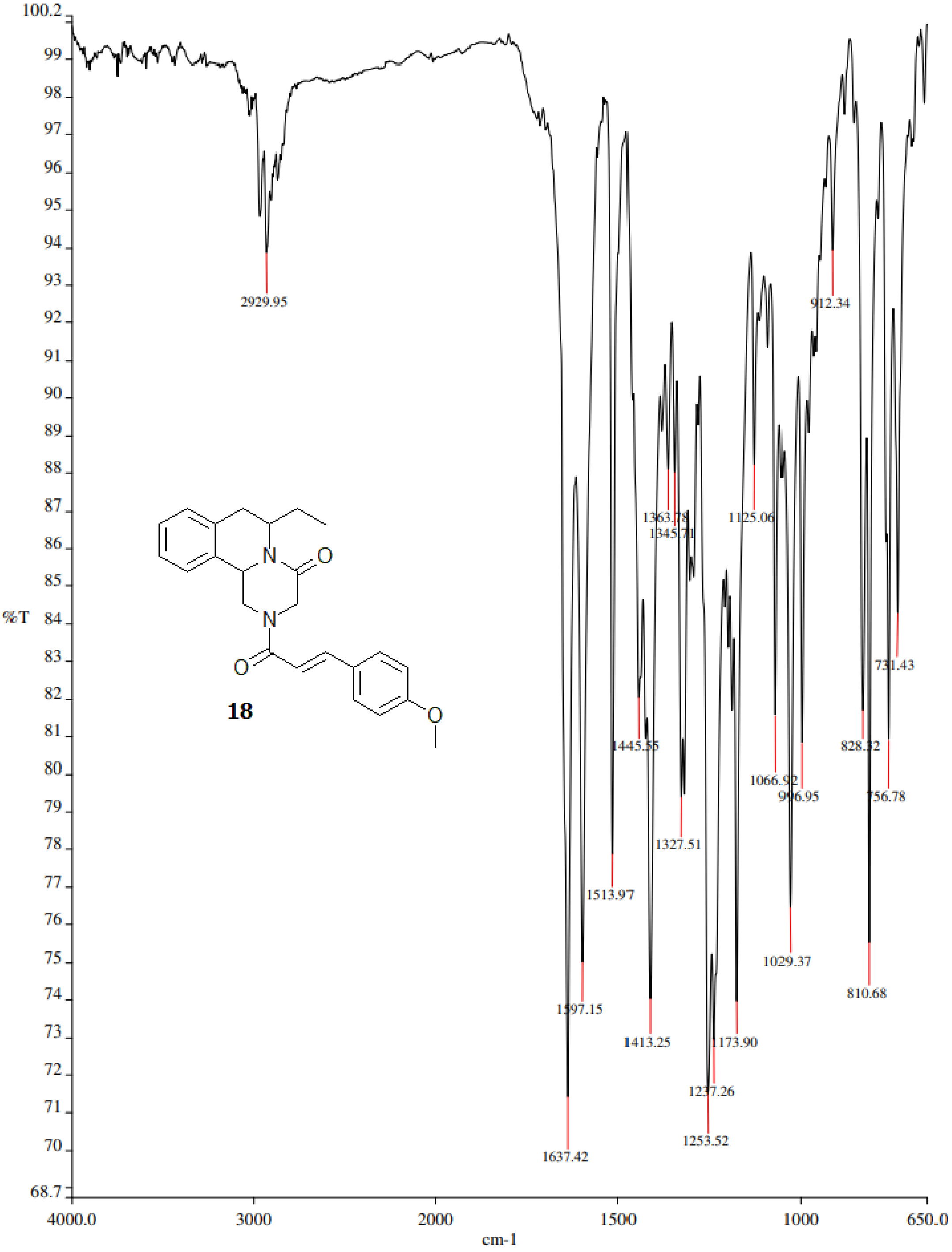

FT-IR spectrum of 6-Ethyl-2-[(2*E*)-3-phenylprop-2-enoyl]-1,2,3,6,7,11b-hexahydro-4H-pyrazino[2,1-a]isoquinolin-4-one **38**

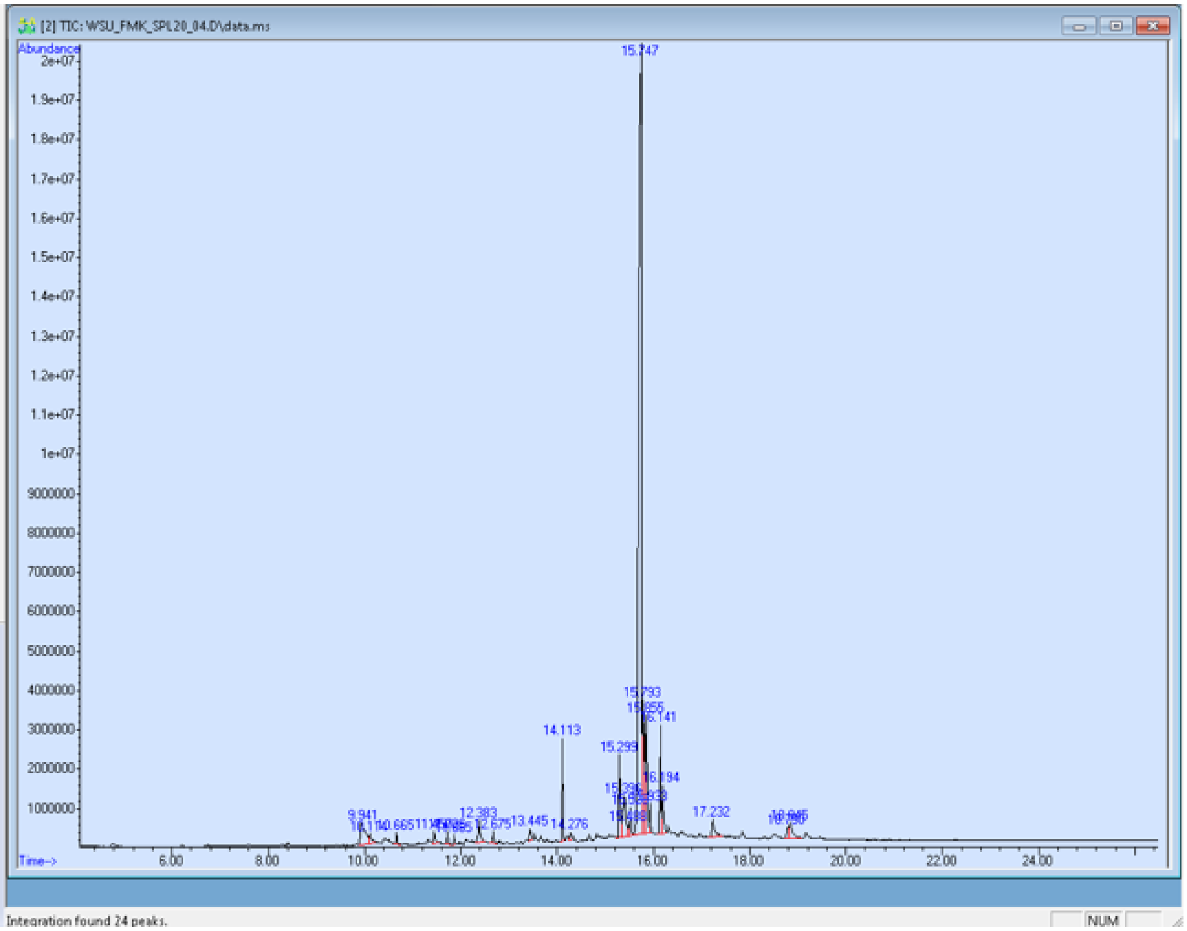

Chromatograph of **22**.

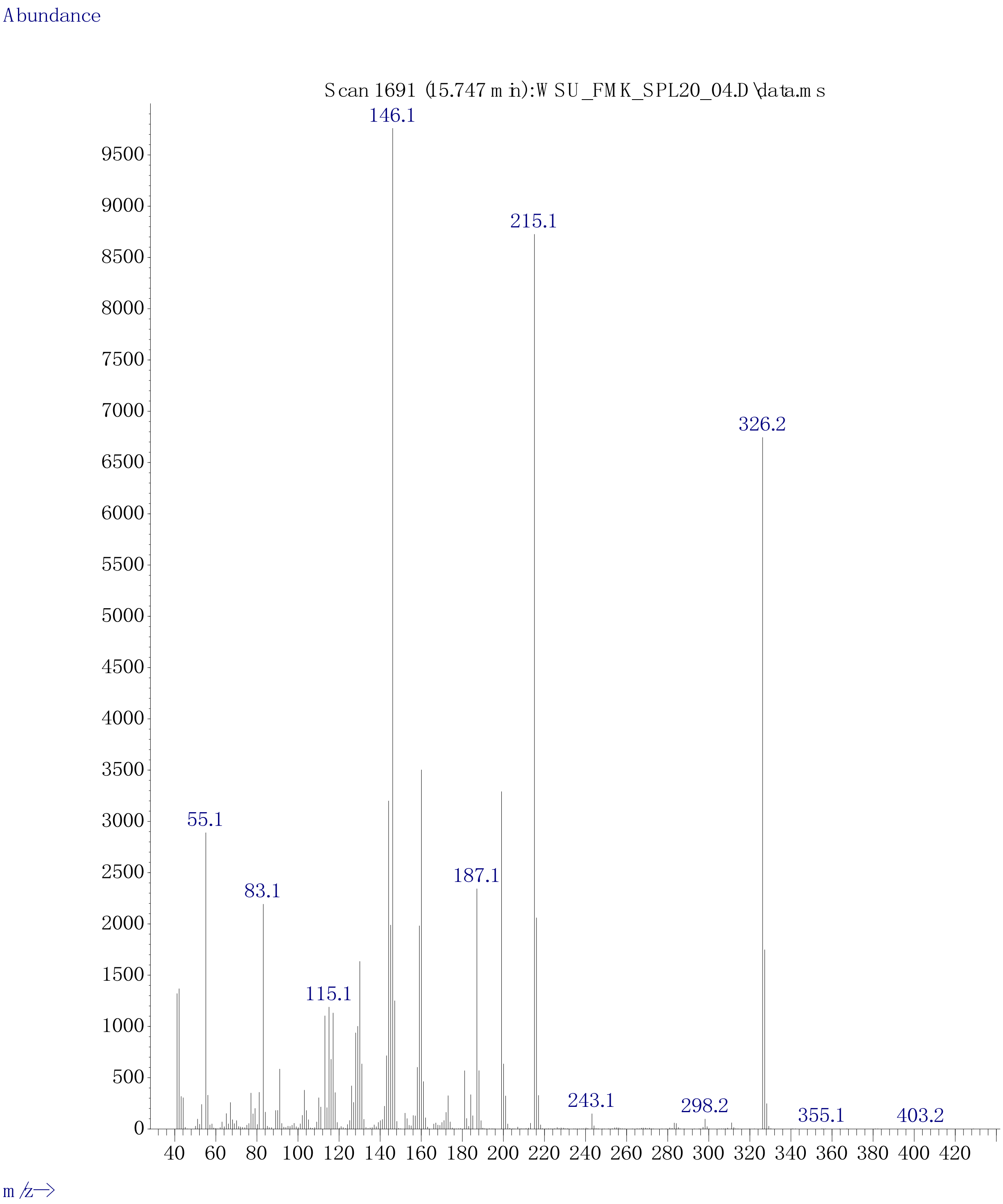

Mass spectrum (m/z) of **22**.

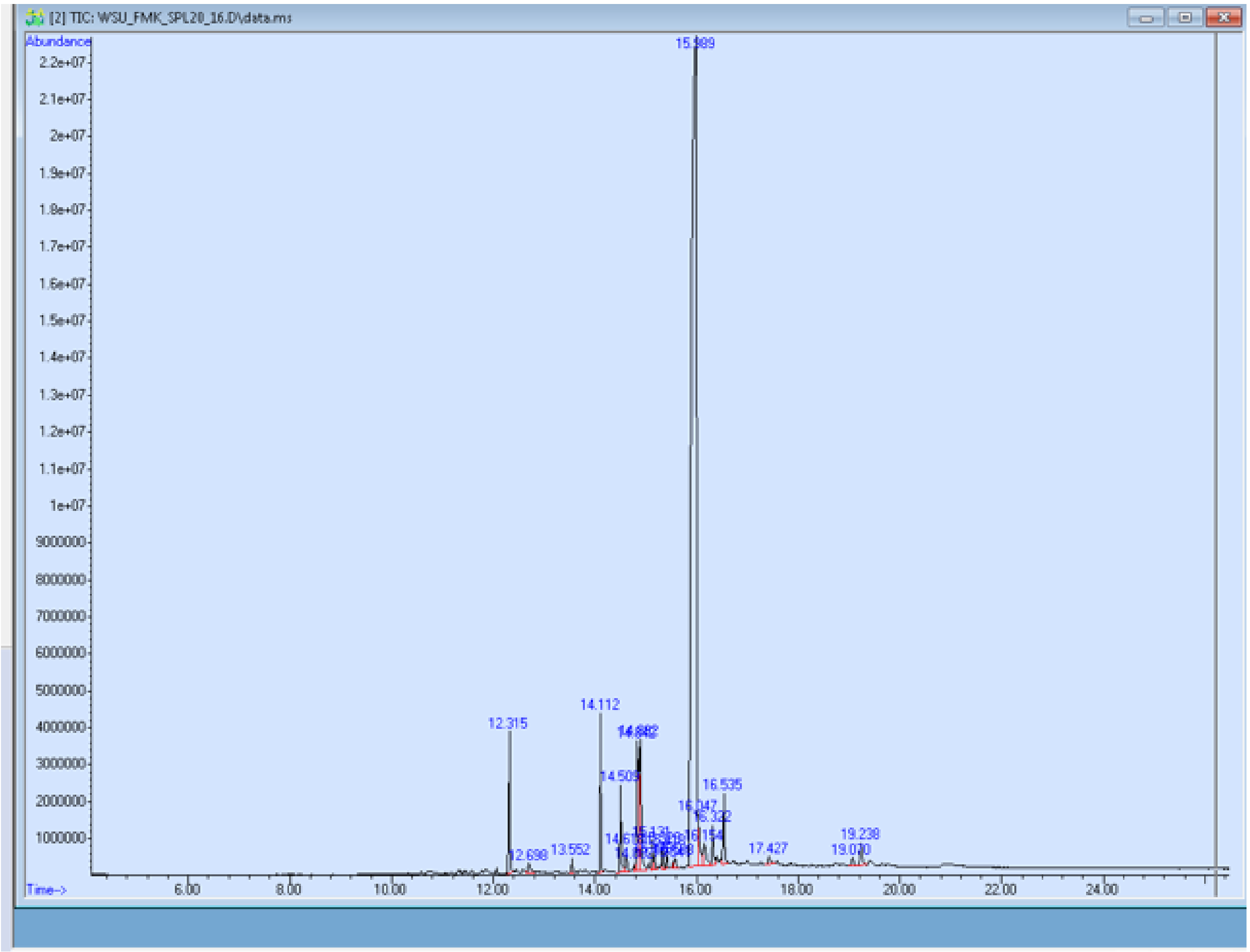

Chromatograph of **23**.

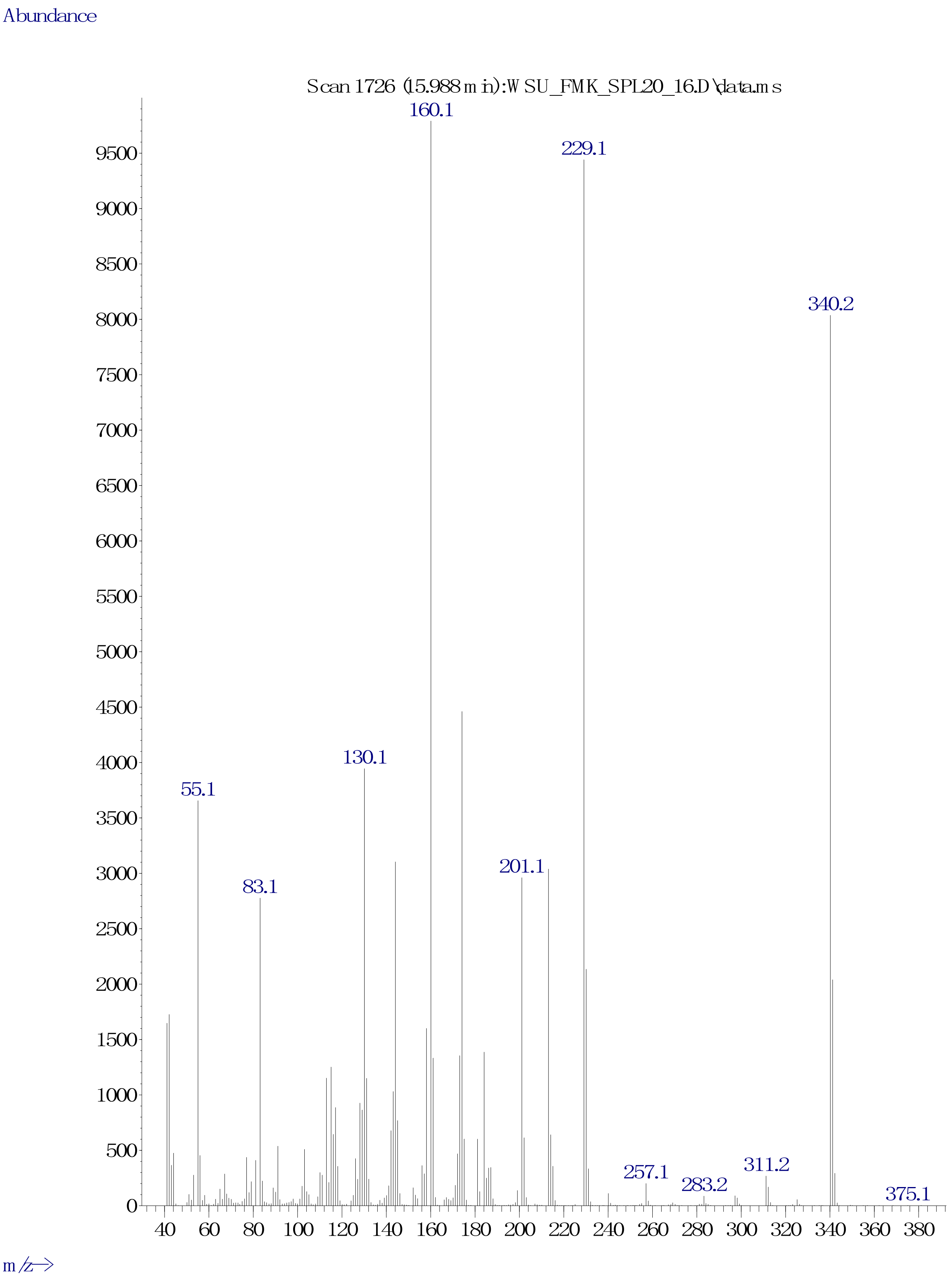

Mass spectrum (m/z) of **23**.

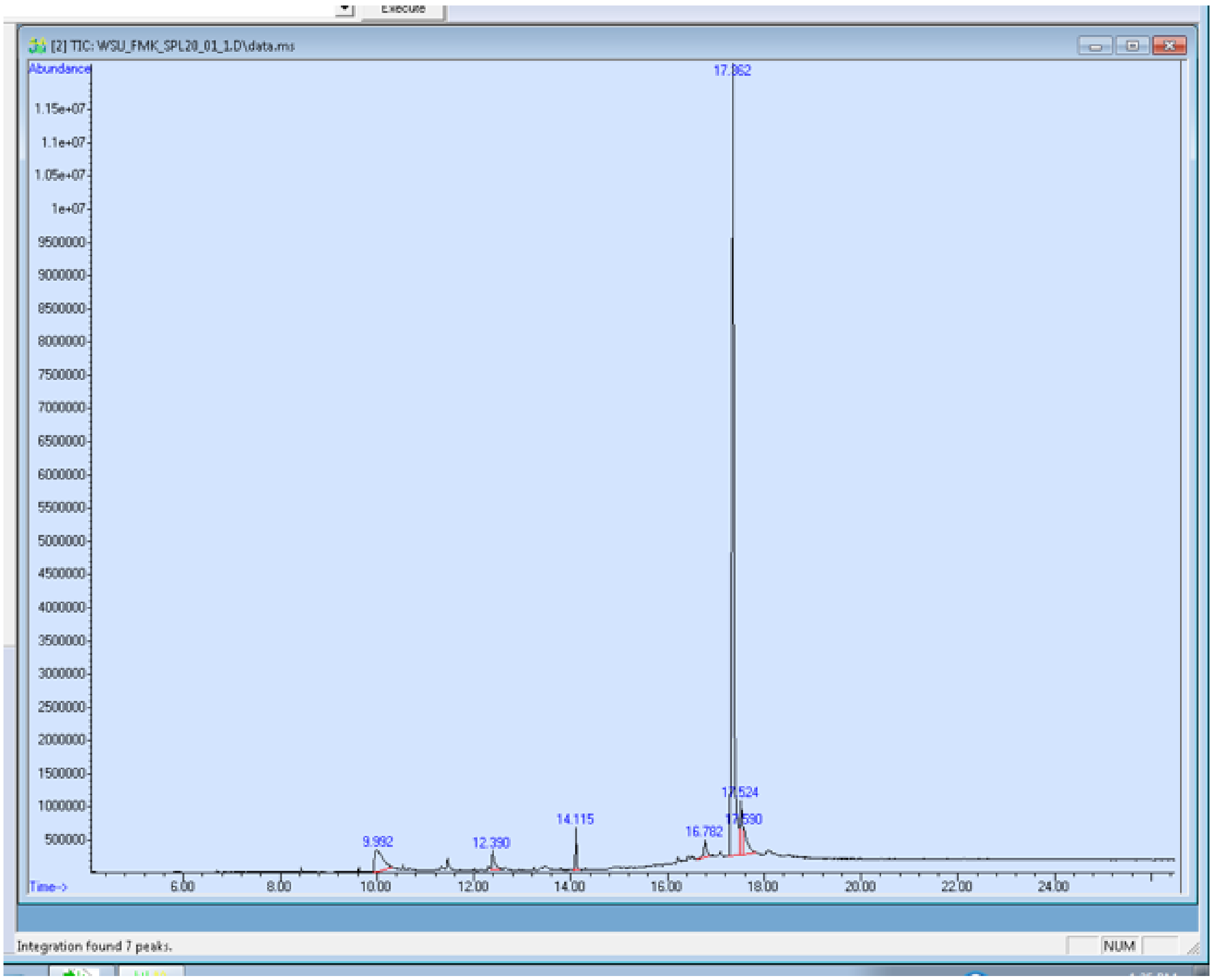

Chromatograph of **24**

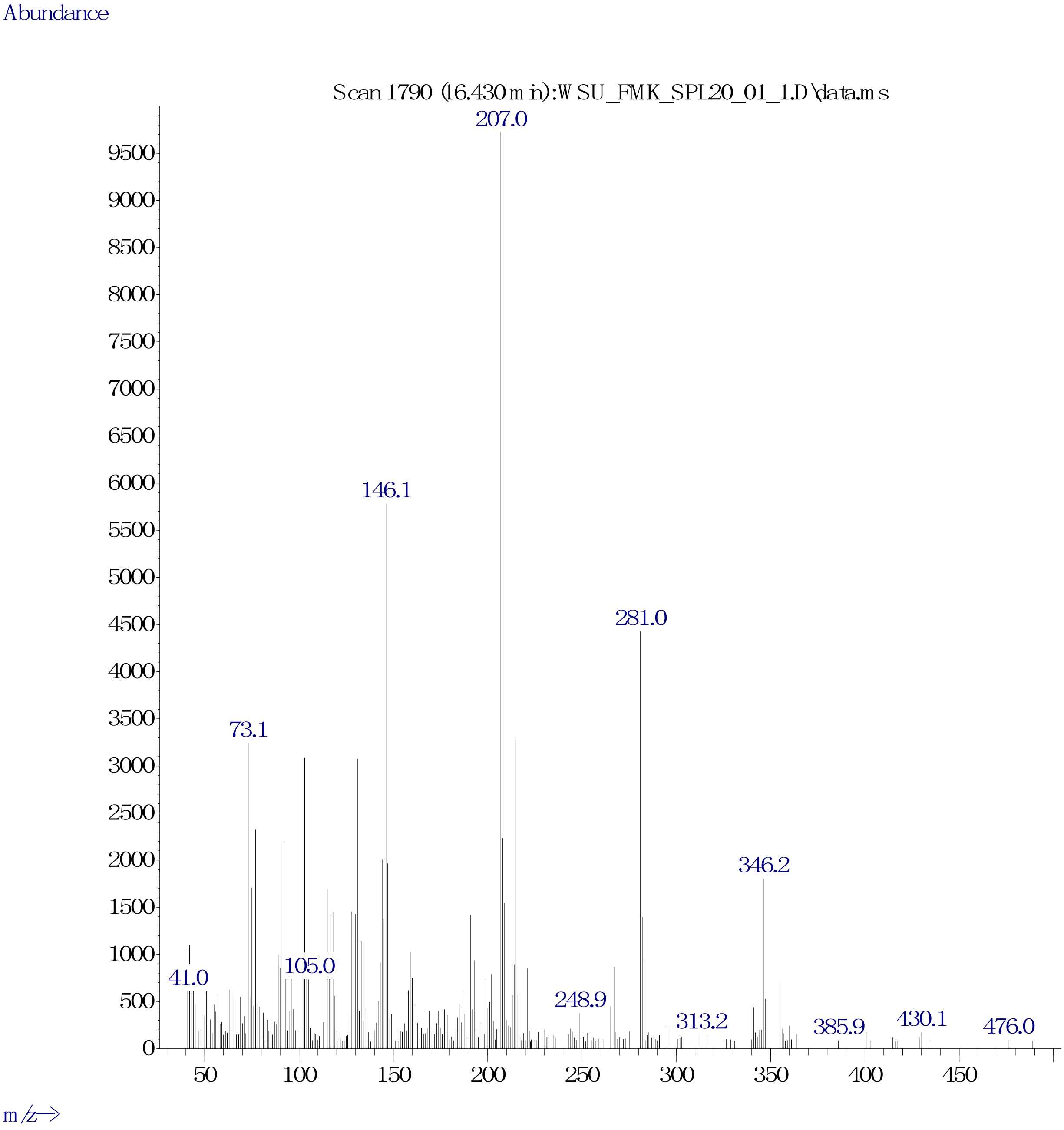

Mass spectrum (m/z) of **24**

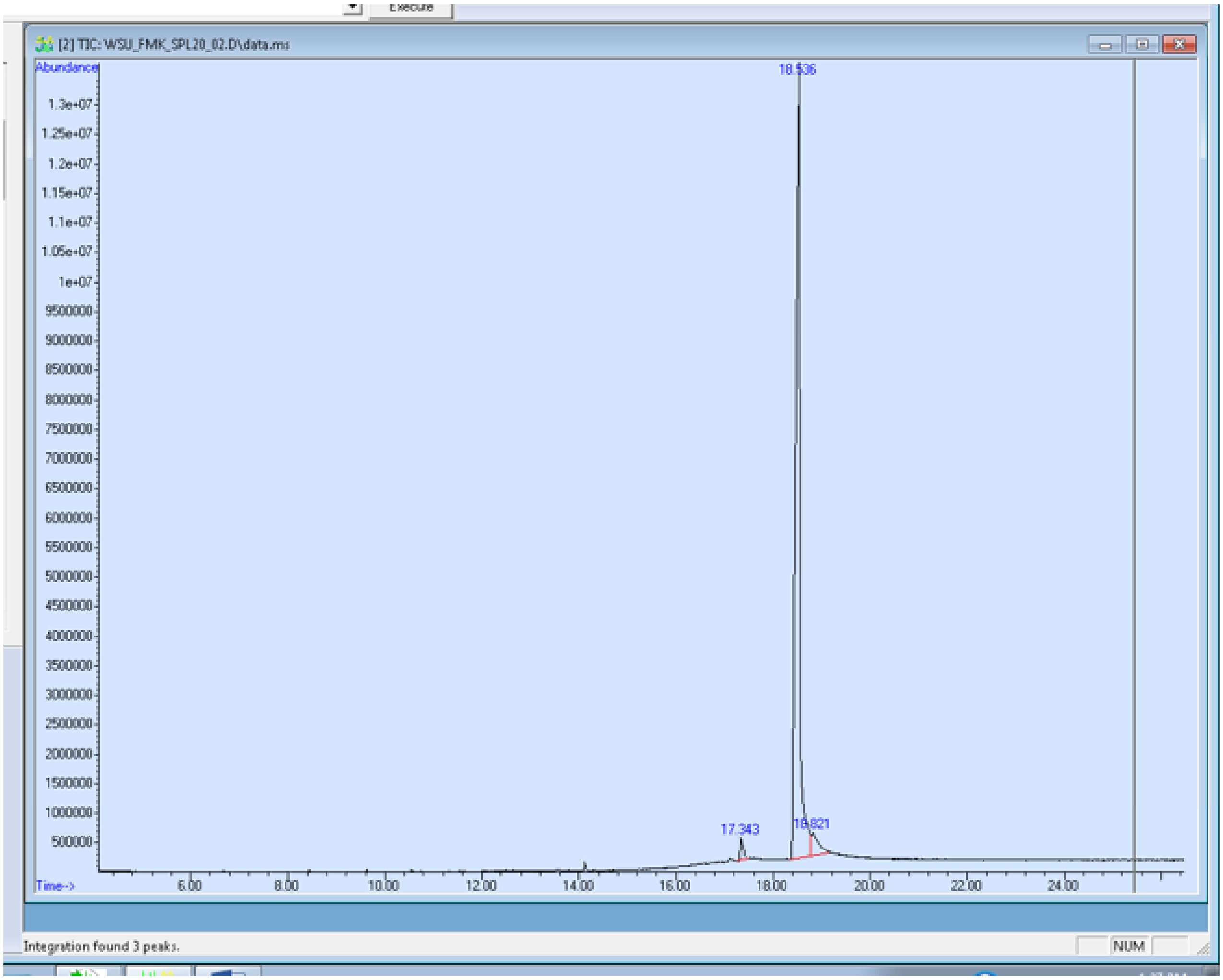

Chromatograph **25**.

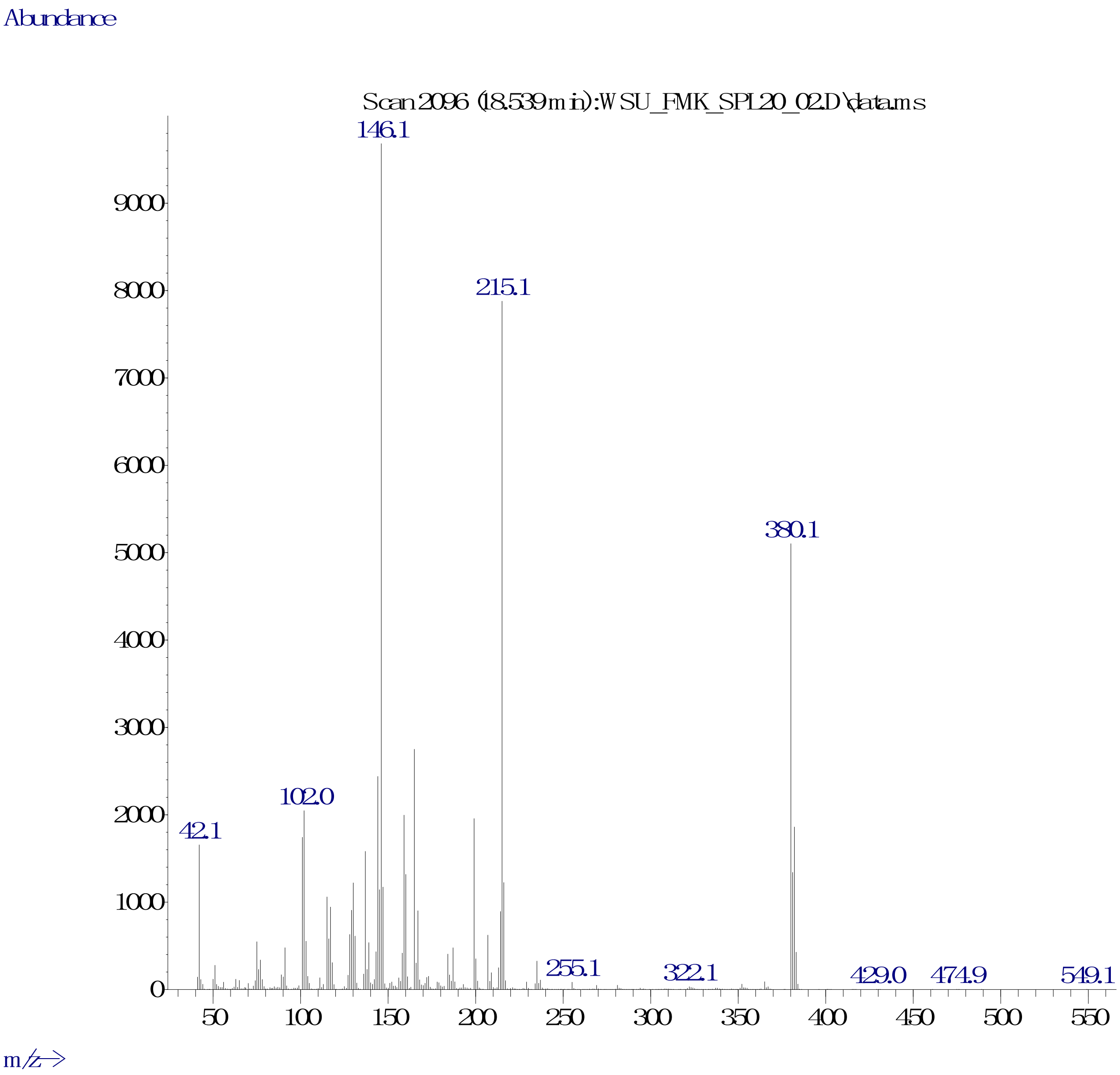

Mass spectrum (m/z) of **25**.

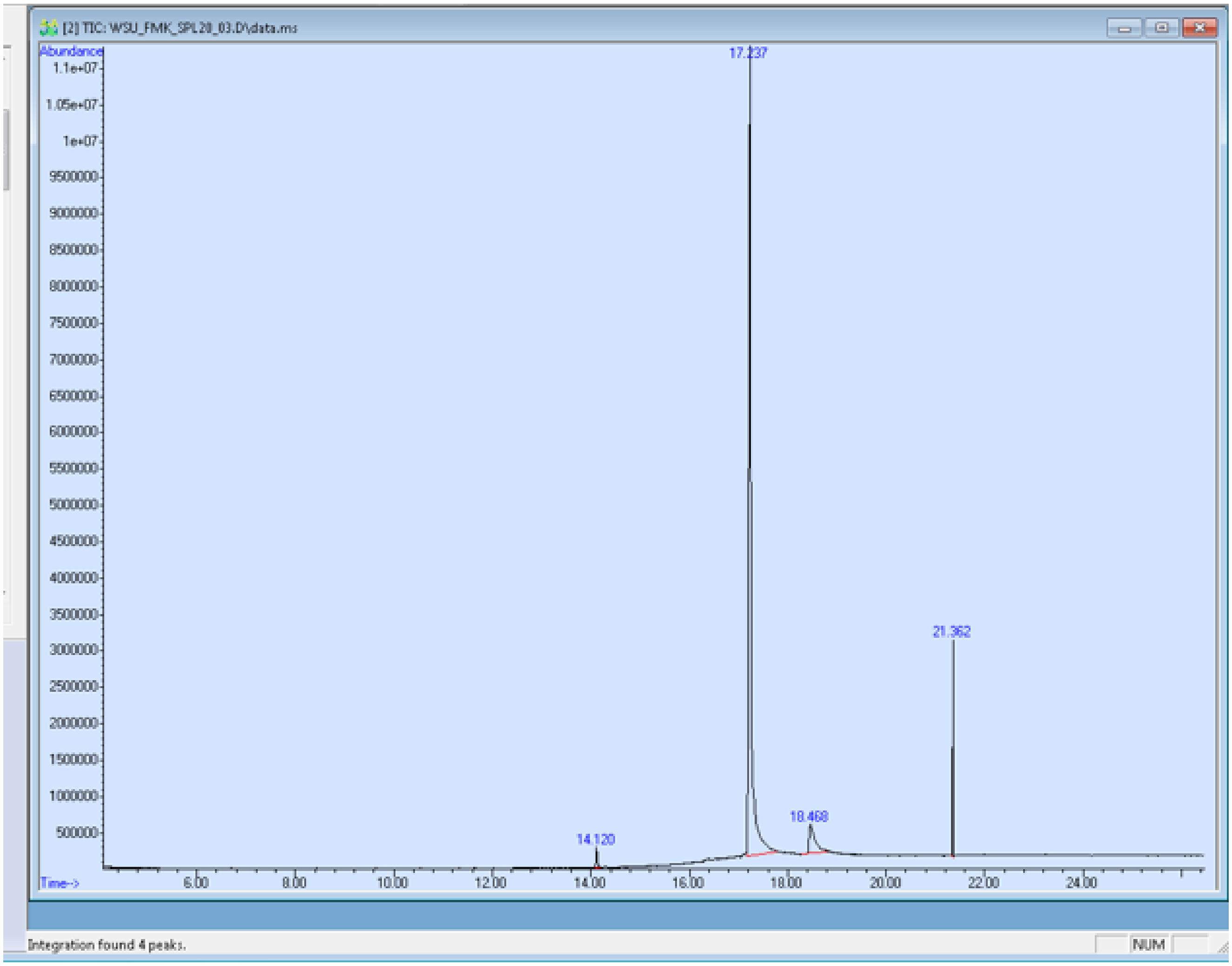

GC Chromatograph of **26**.

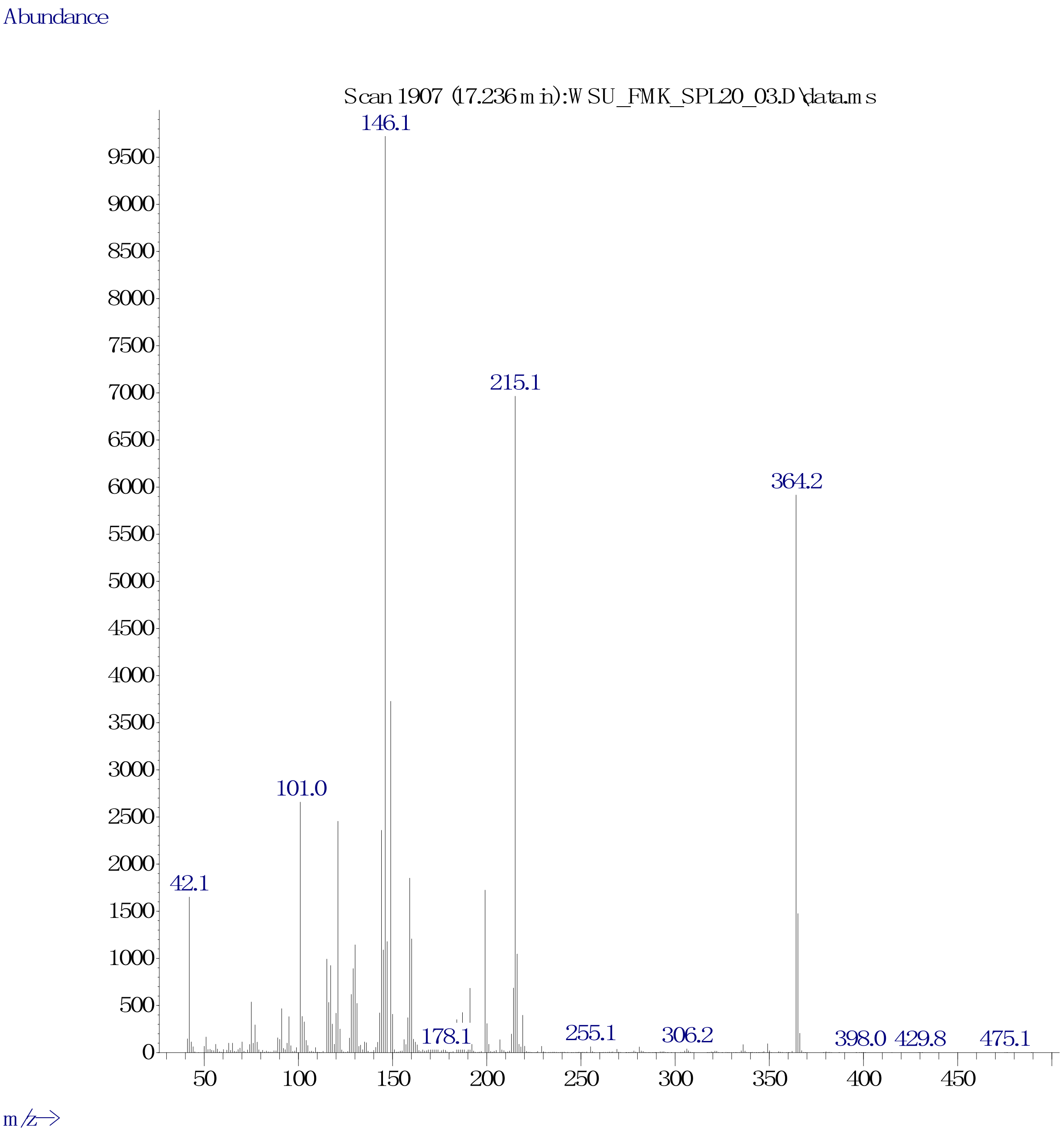

Mass spectrum (m/z) of **26**.

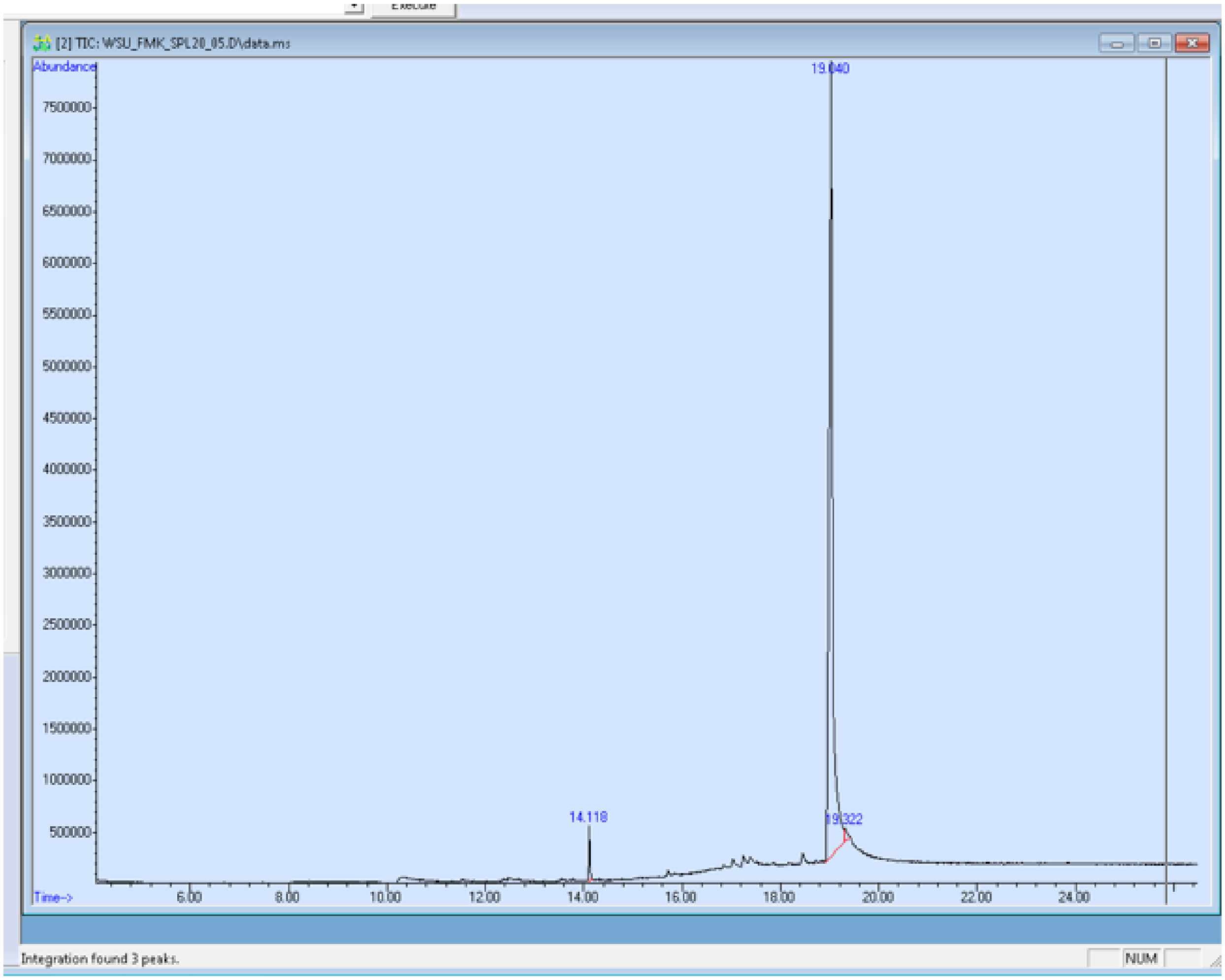

Chromatograph of **27**.

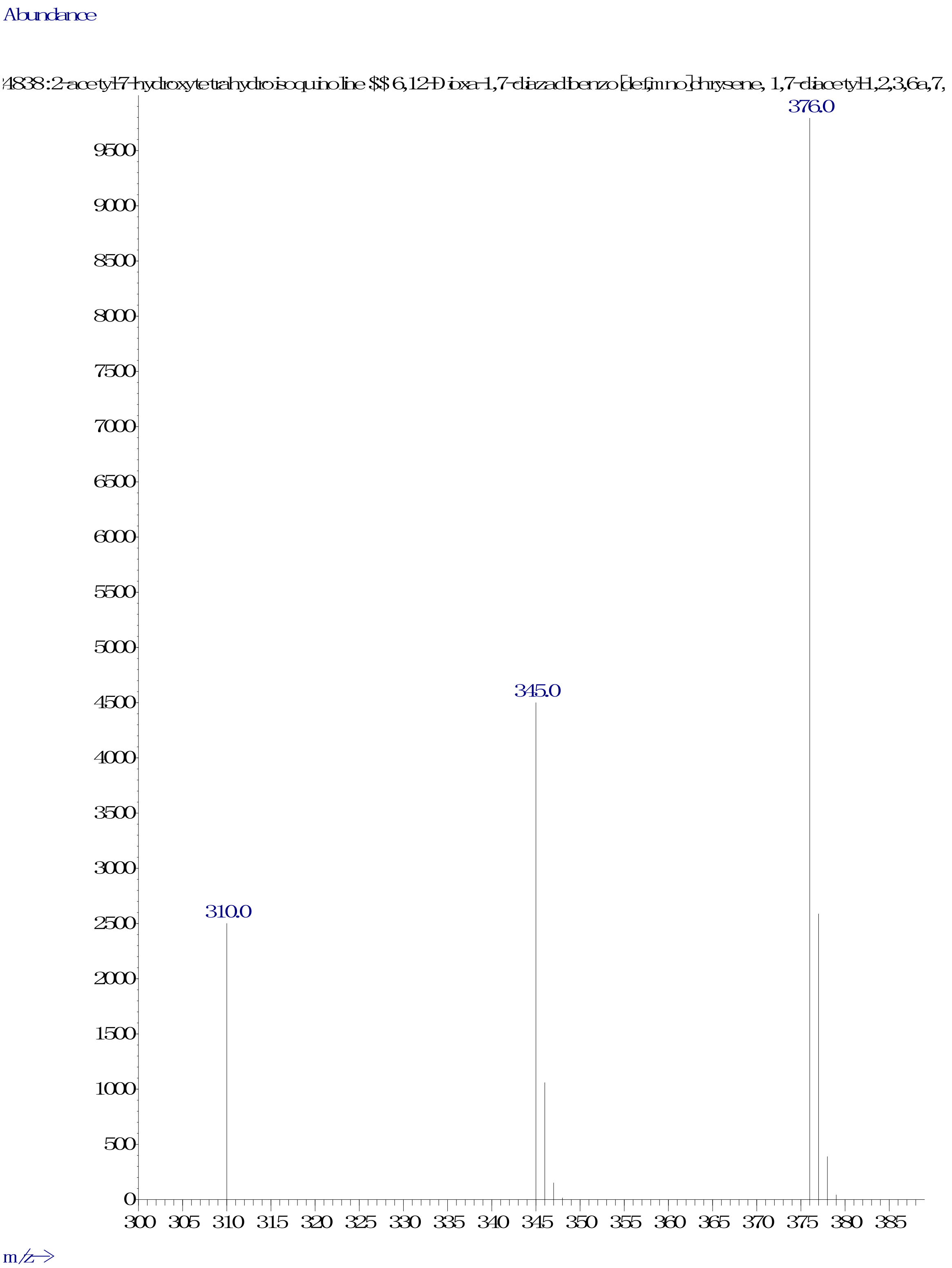

Mass spectrum (m/z) of **27**.

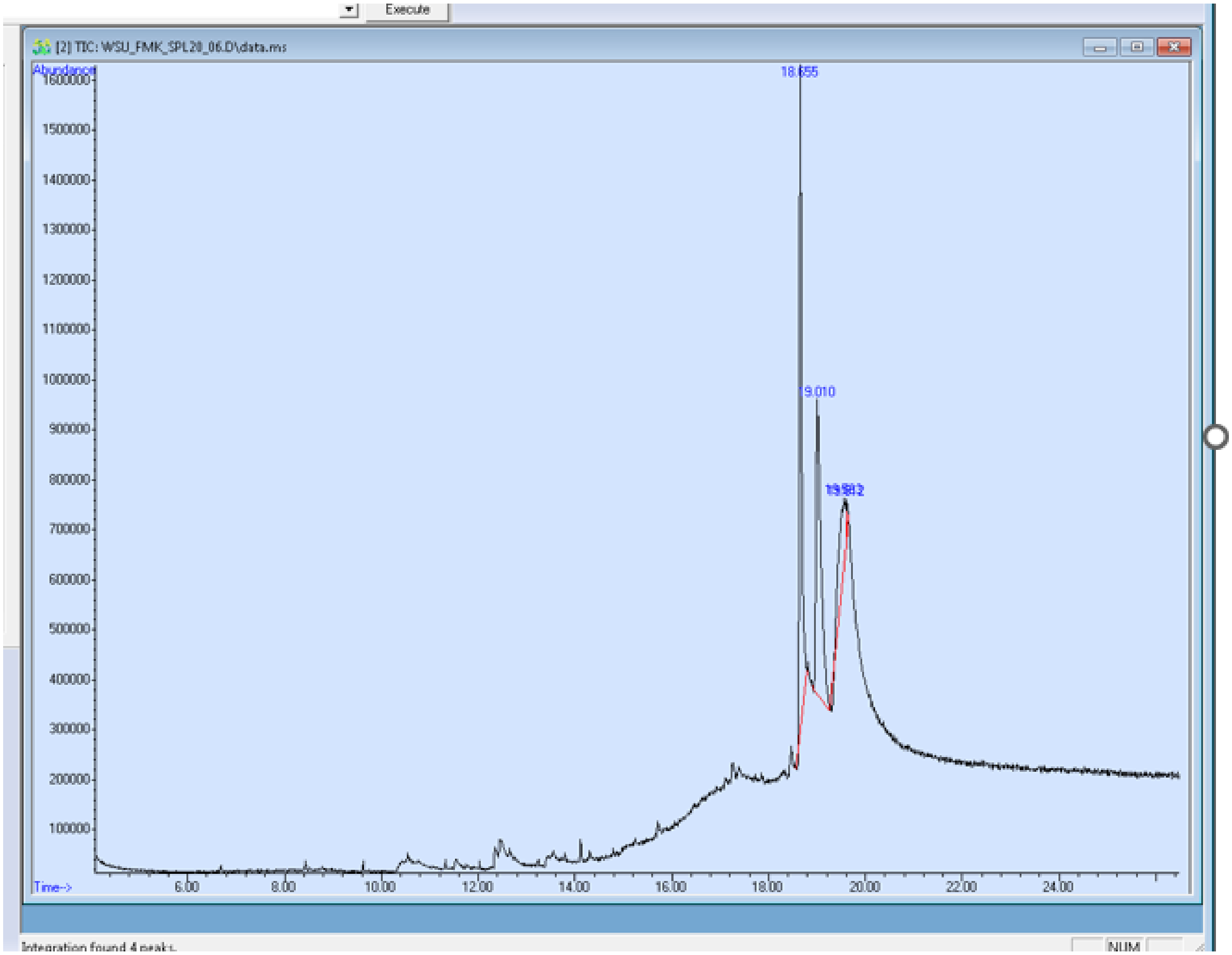

Chromatograph of **28**.

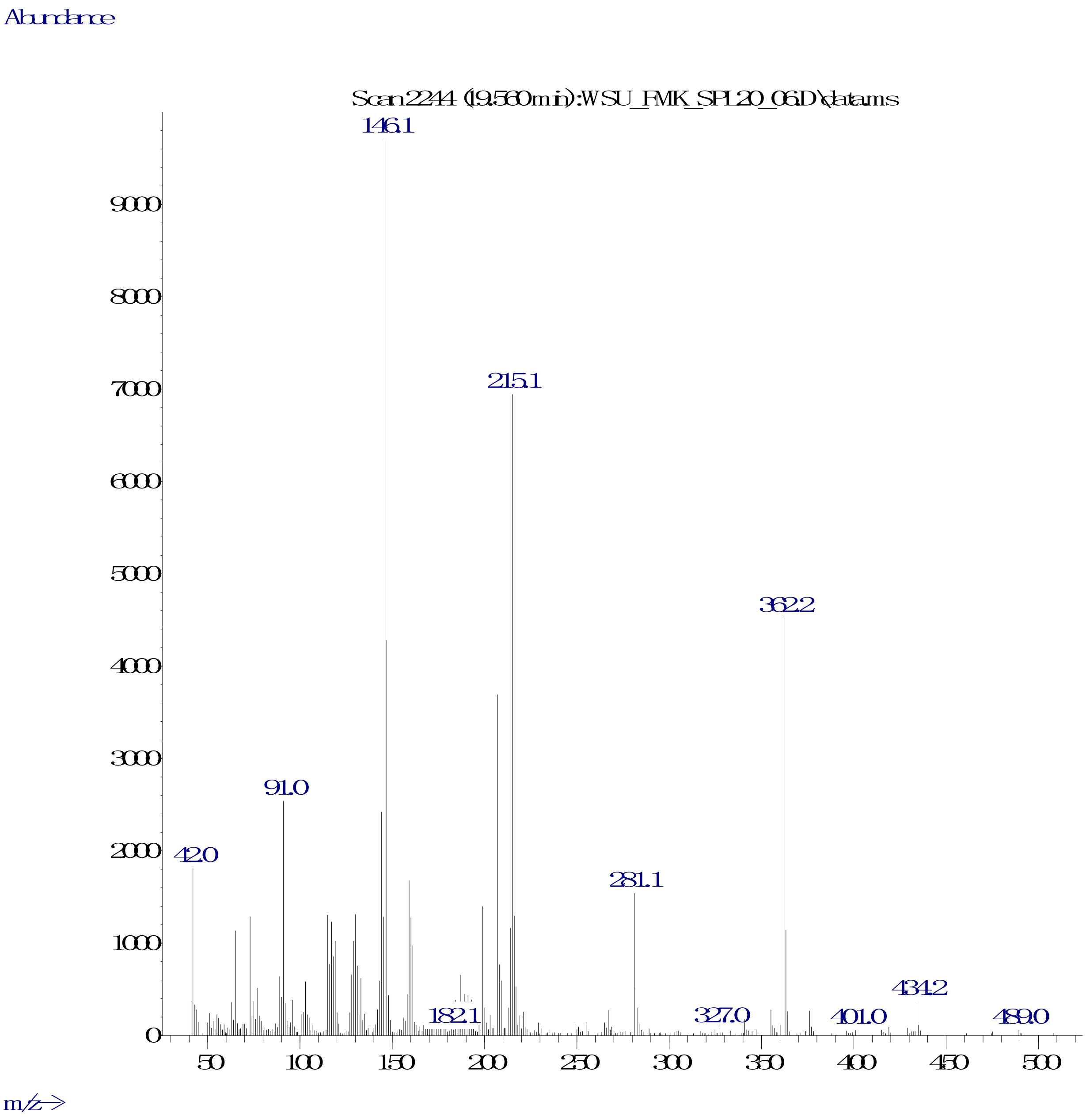

Mass spectrum (m/z) of **28**.

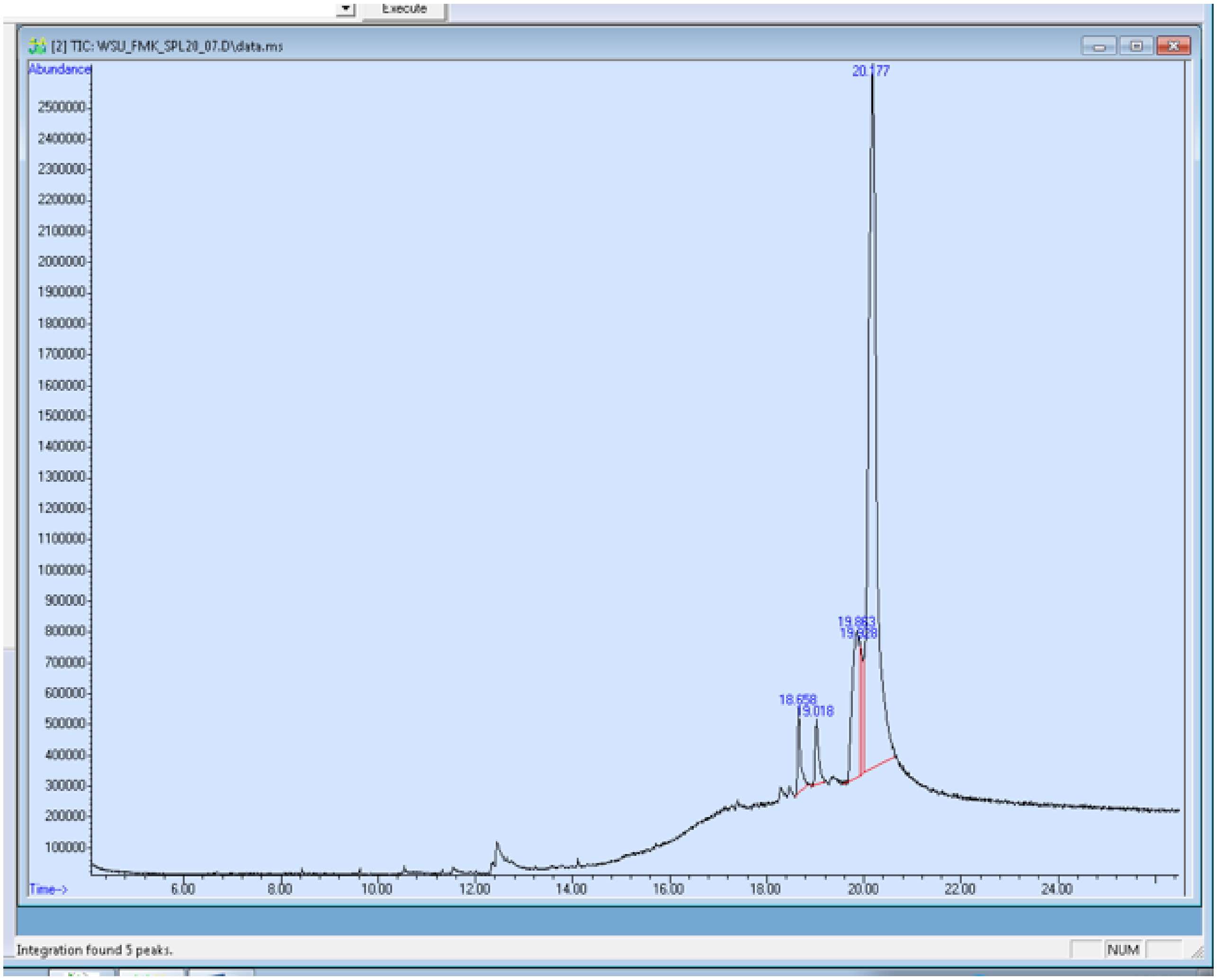

Chromatograph of **29**.

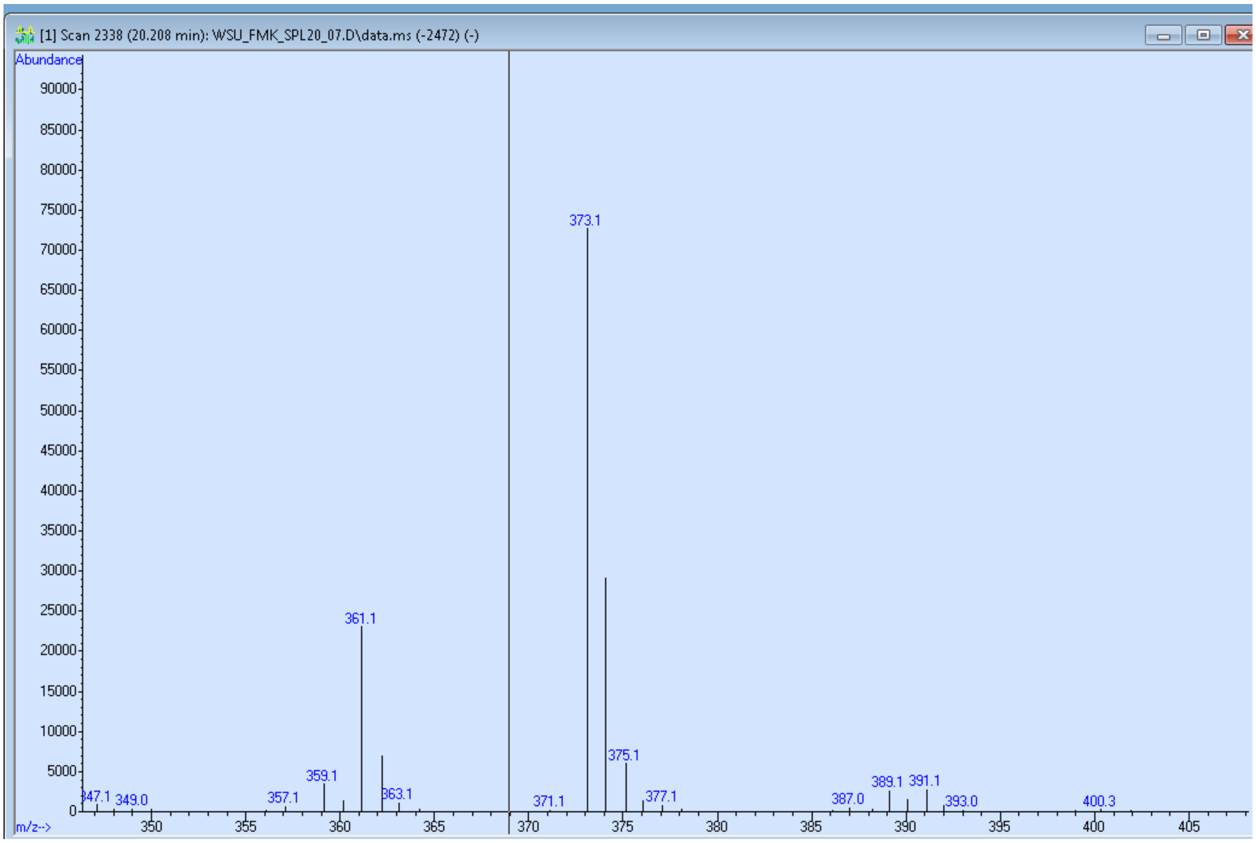

Mass spectrum (m/z) of **29**.

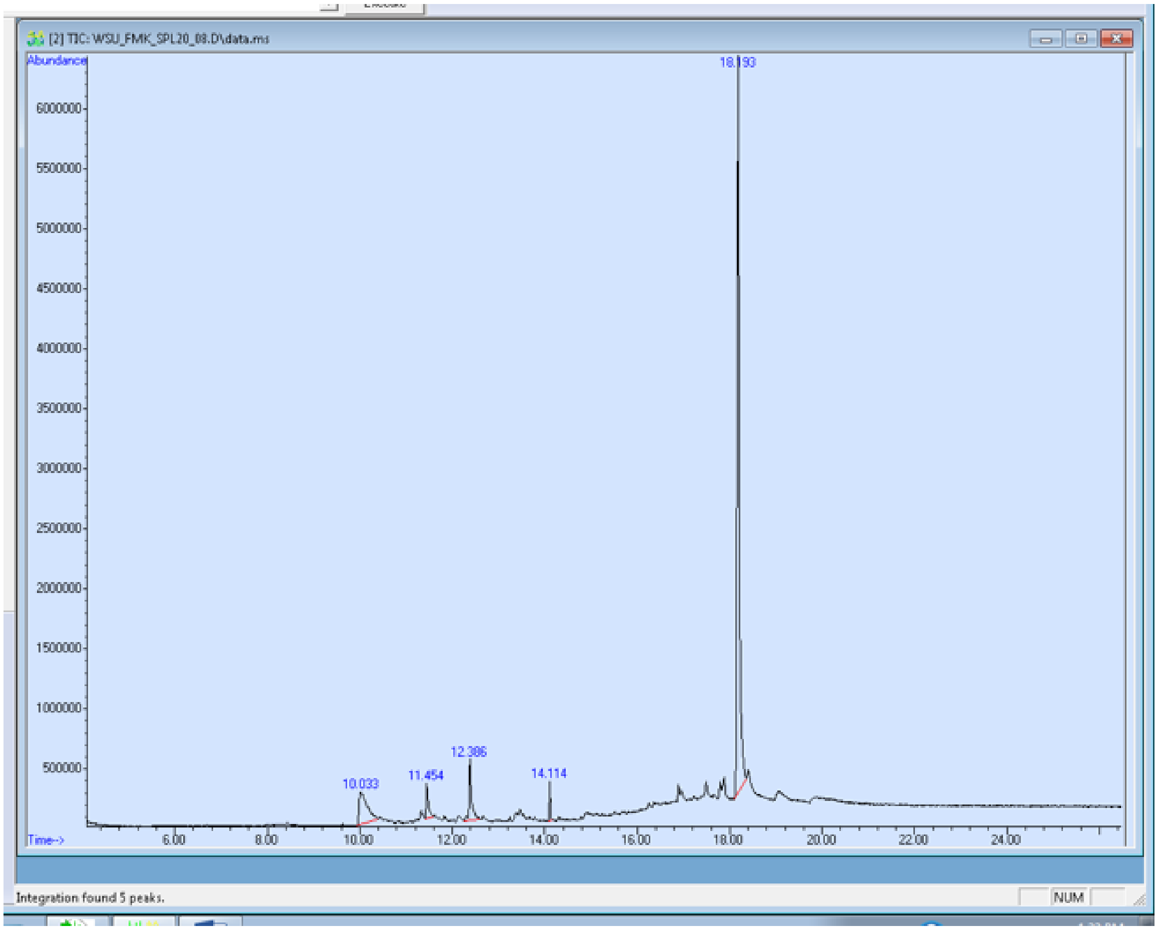

Chromatograph of **30**.

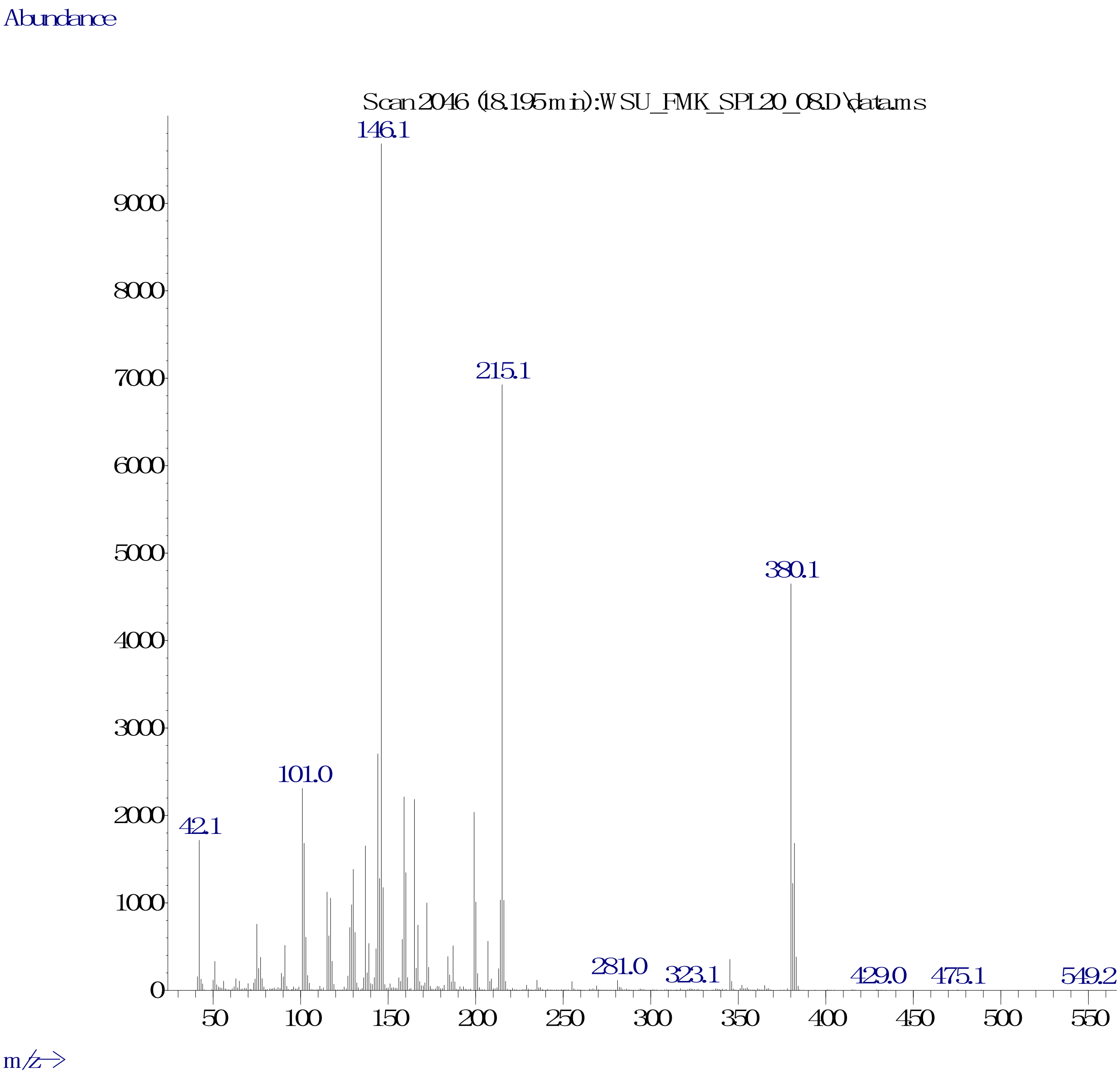

Mass spectrum (m/z) of **30.**

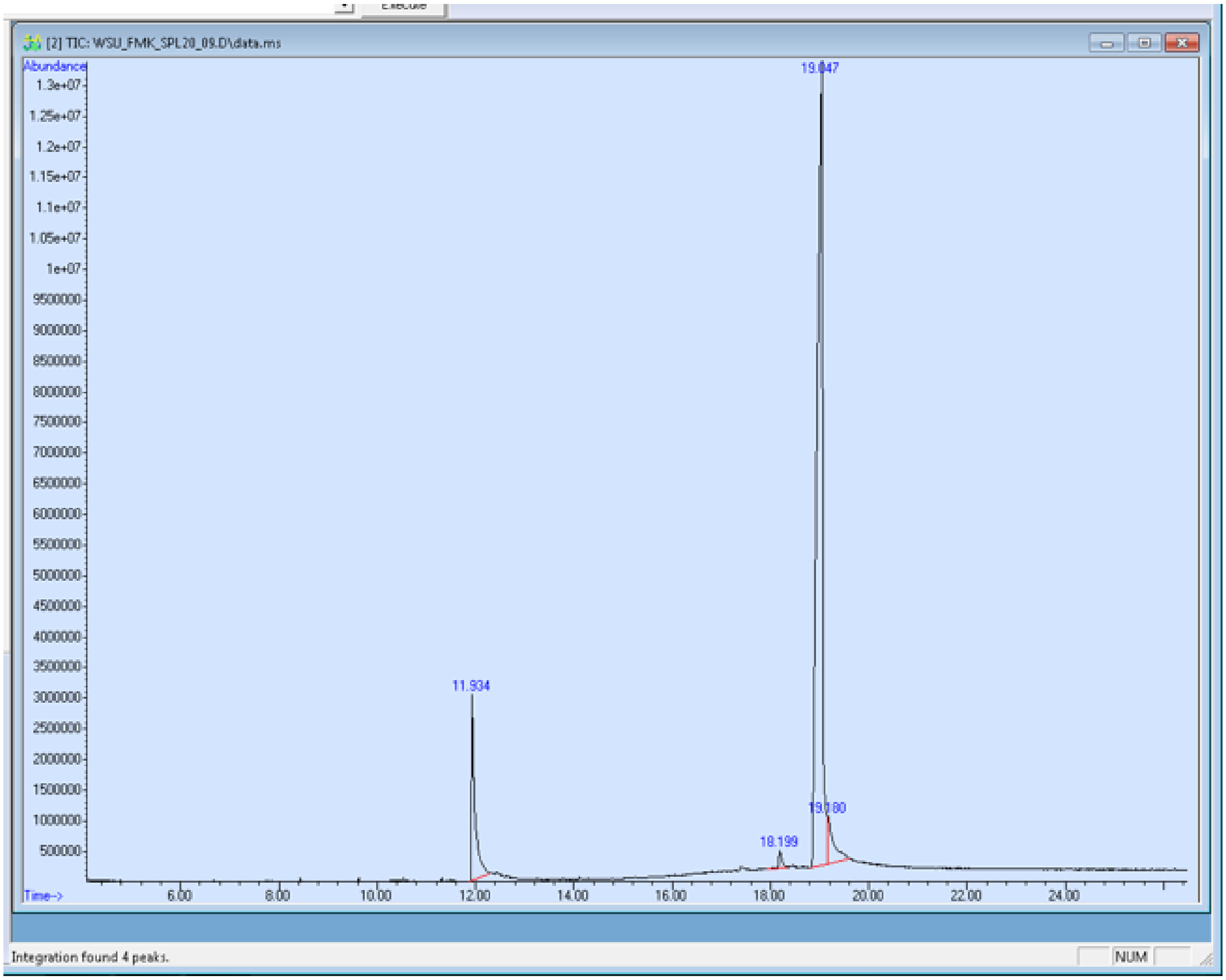

Chromatograph of **31**.

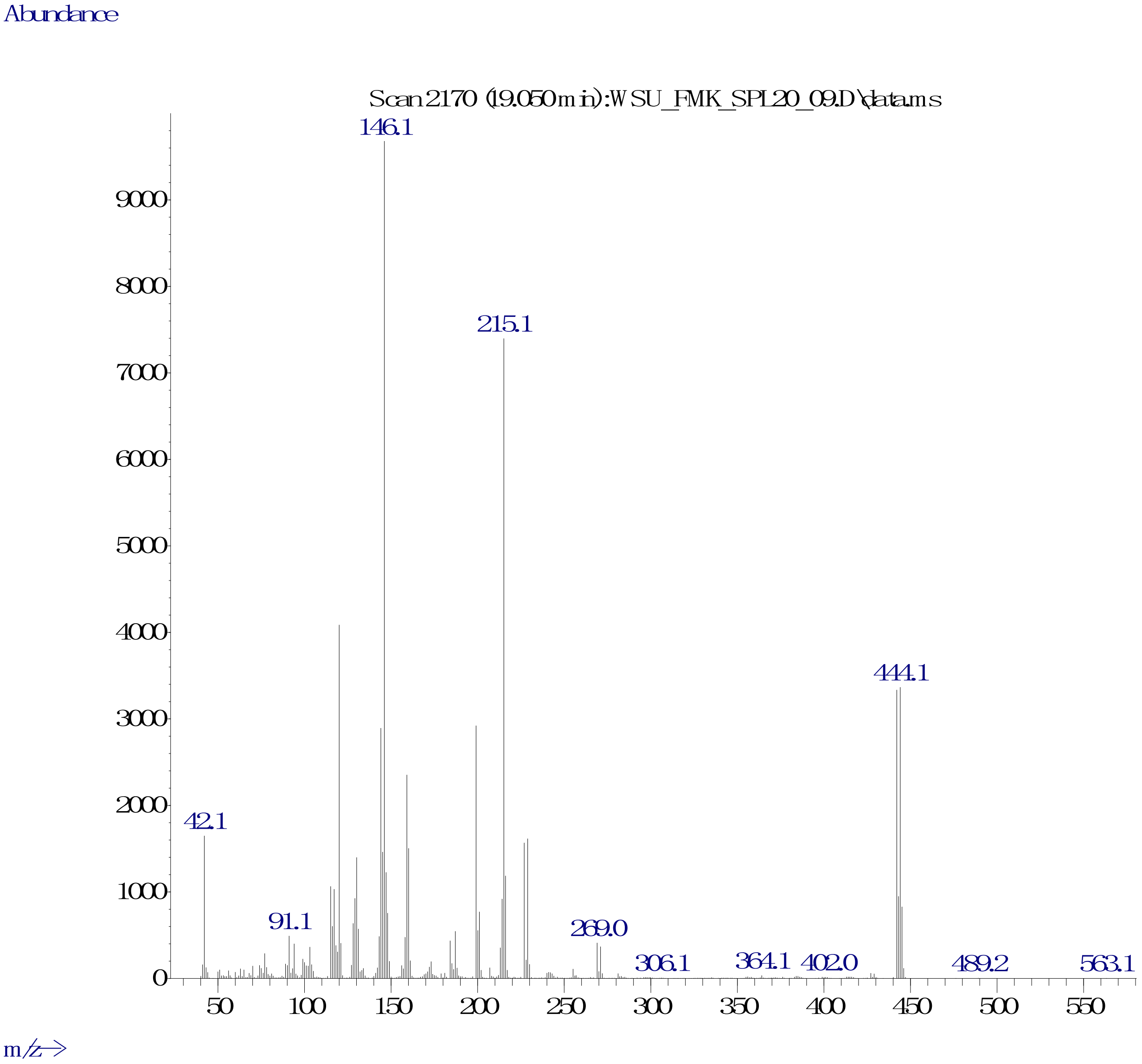

Mass spectrum (m/z) of **31**.

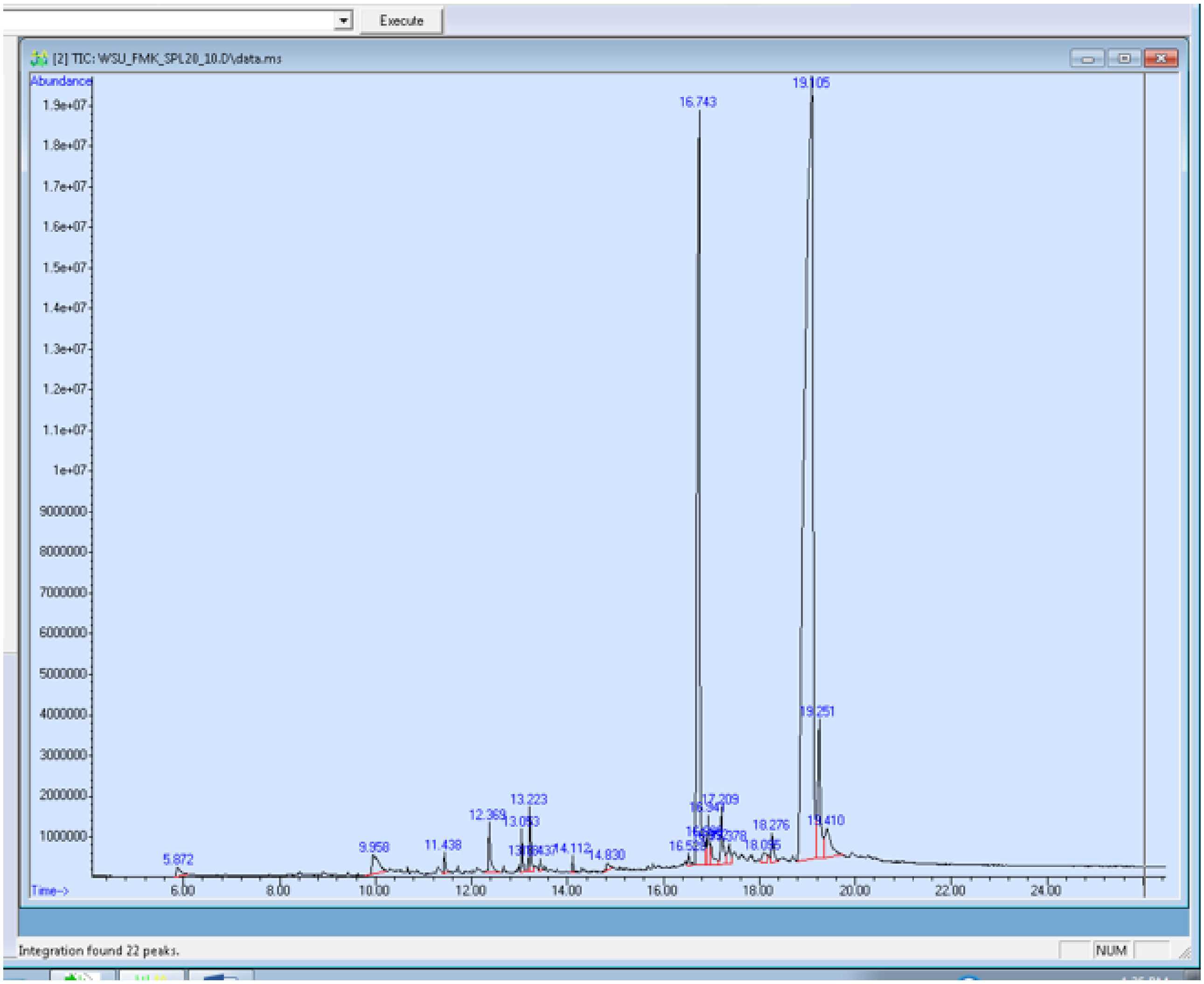

Chromatograph of **32**.

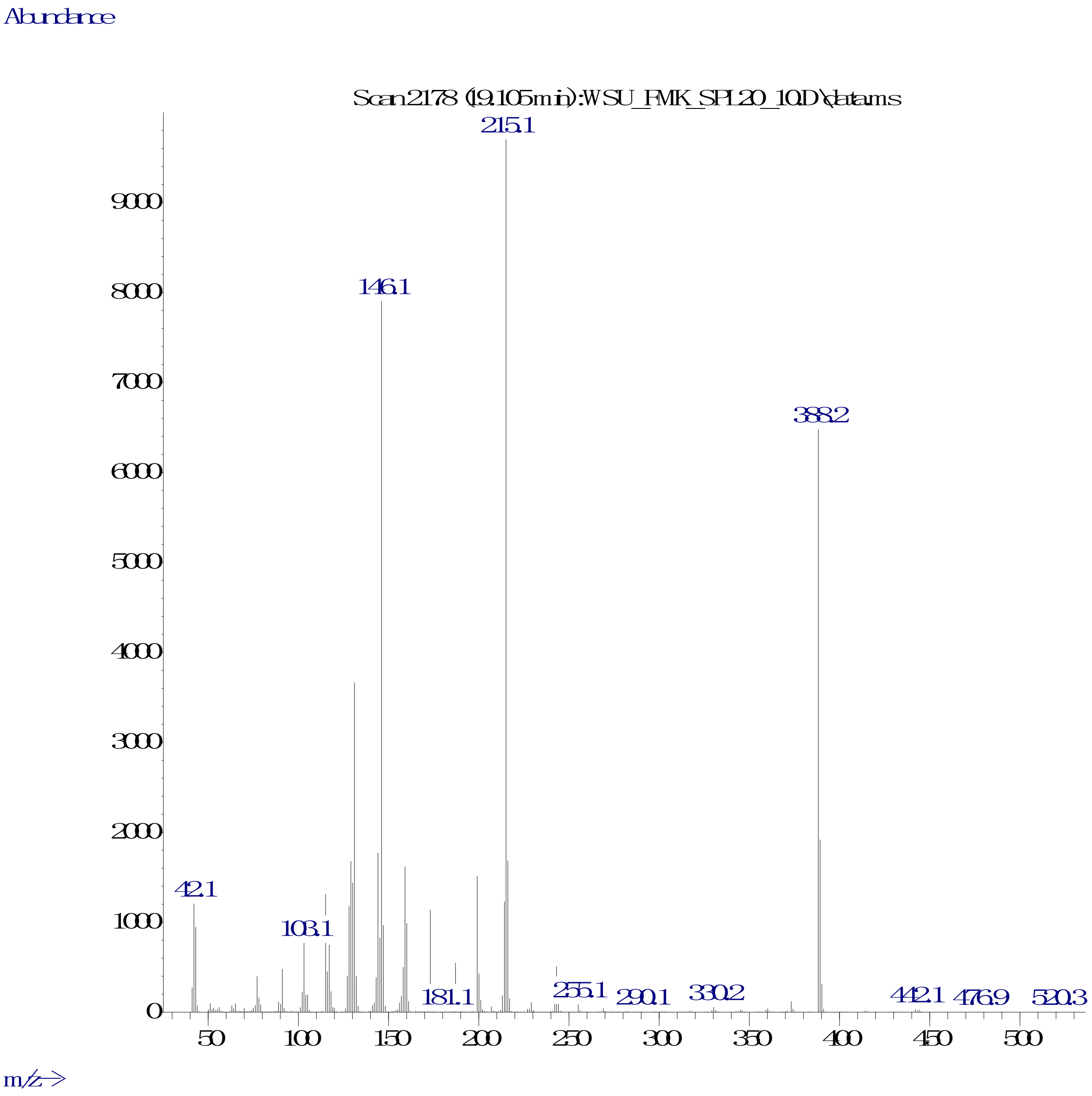

Mass spectrum (m/z) of **32**.

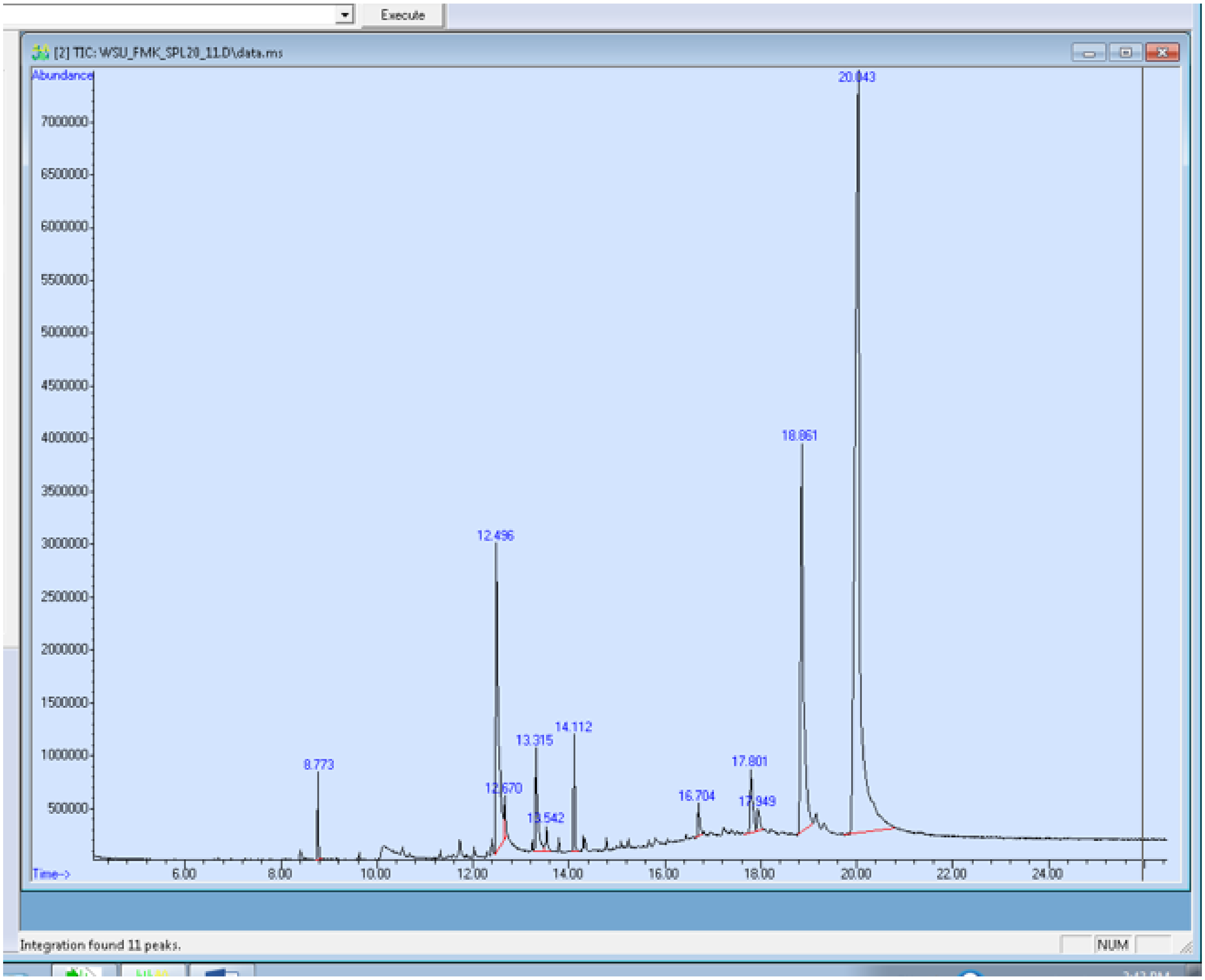

Chromatograph of **33**.

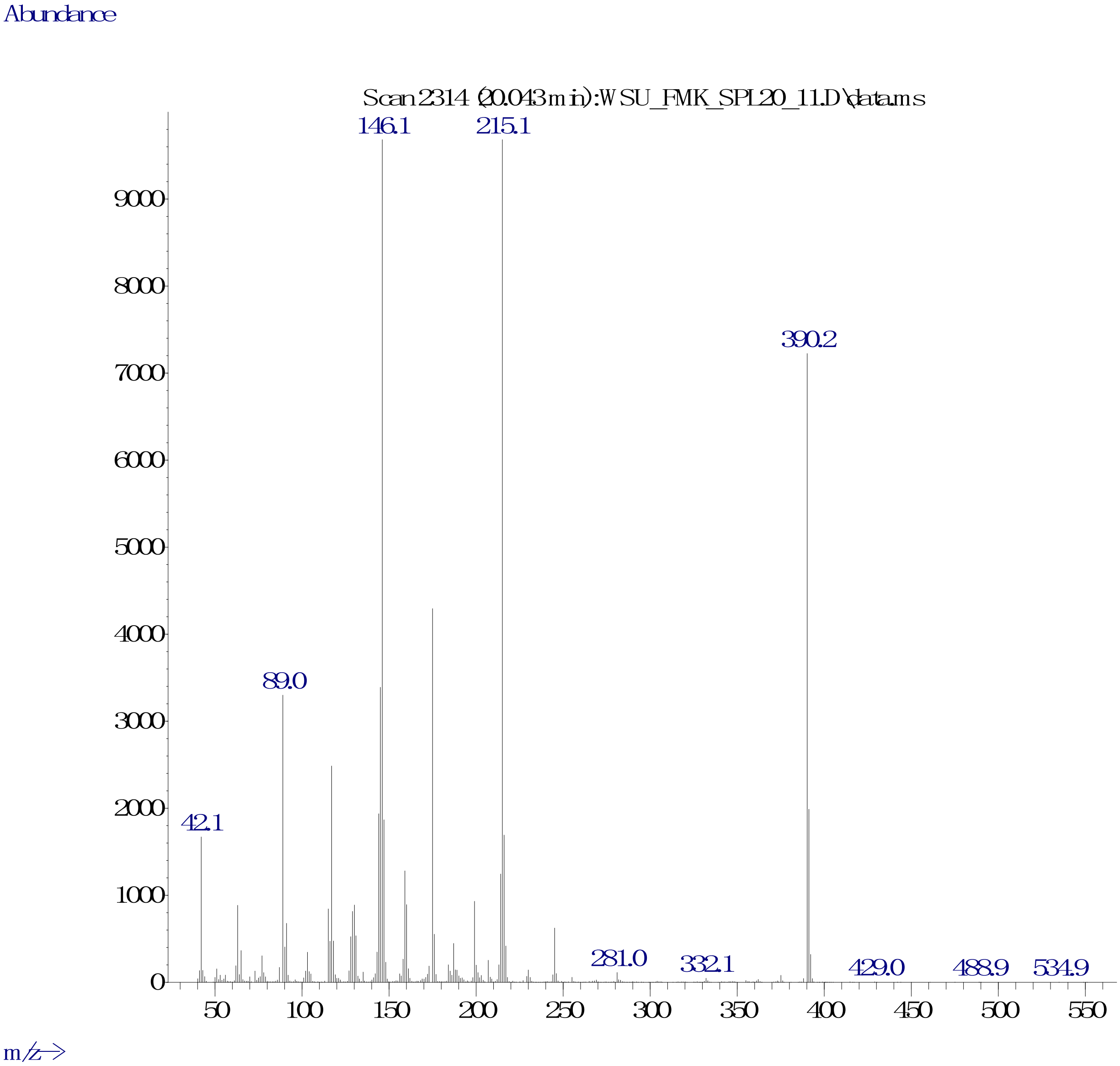

Mass spectrum (m/z) of **33**.

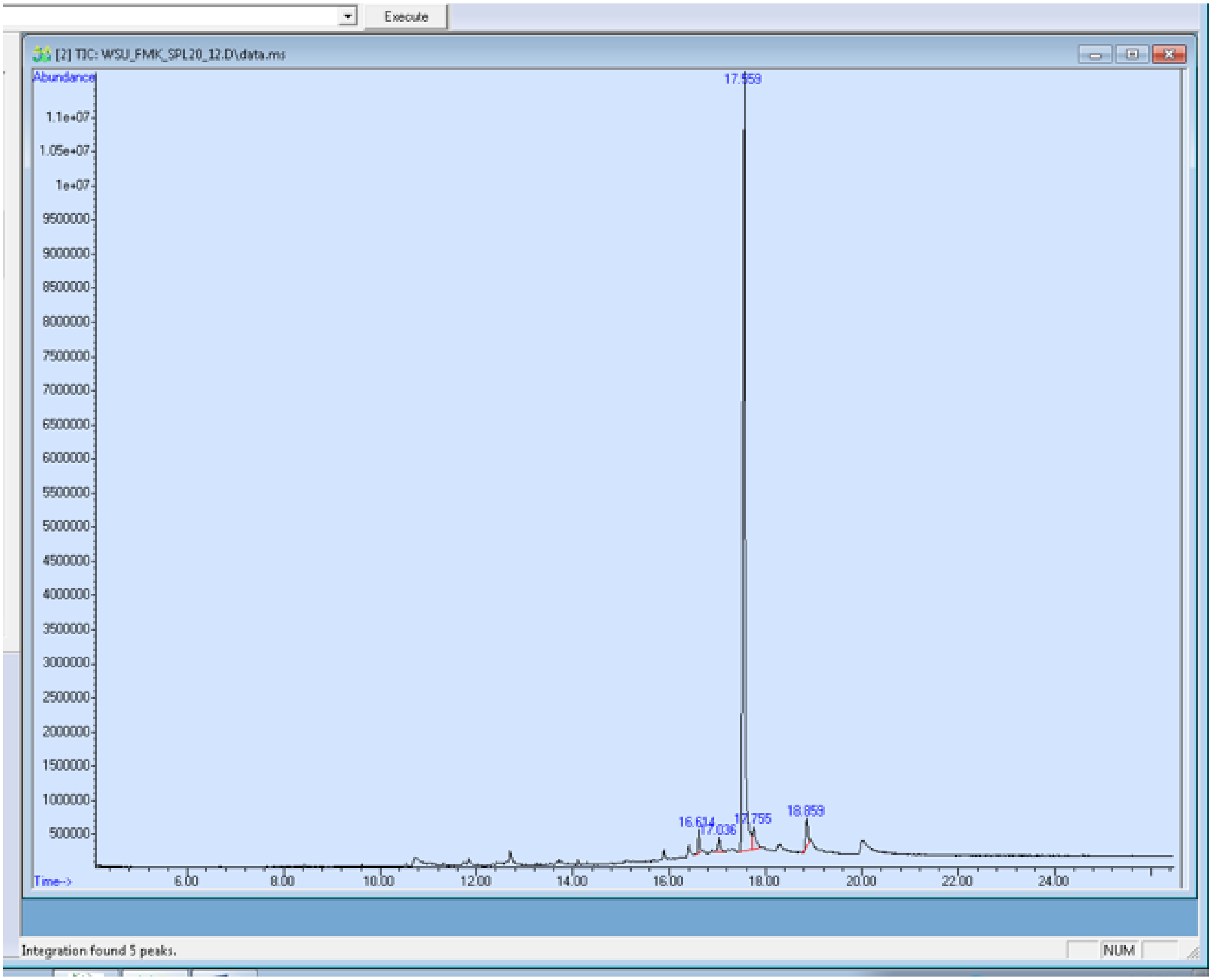

Chromatograph of **34**.

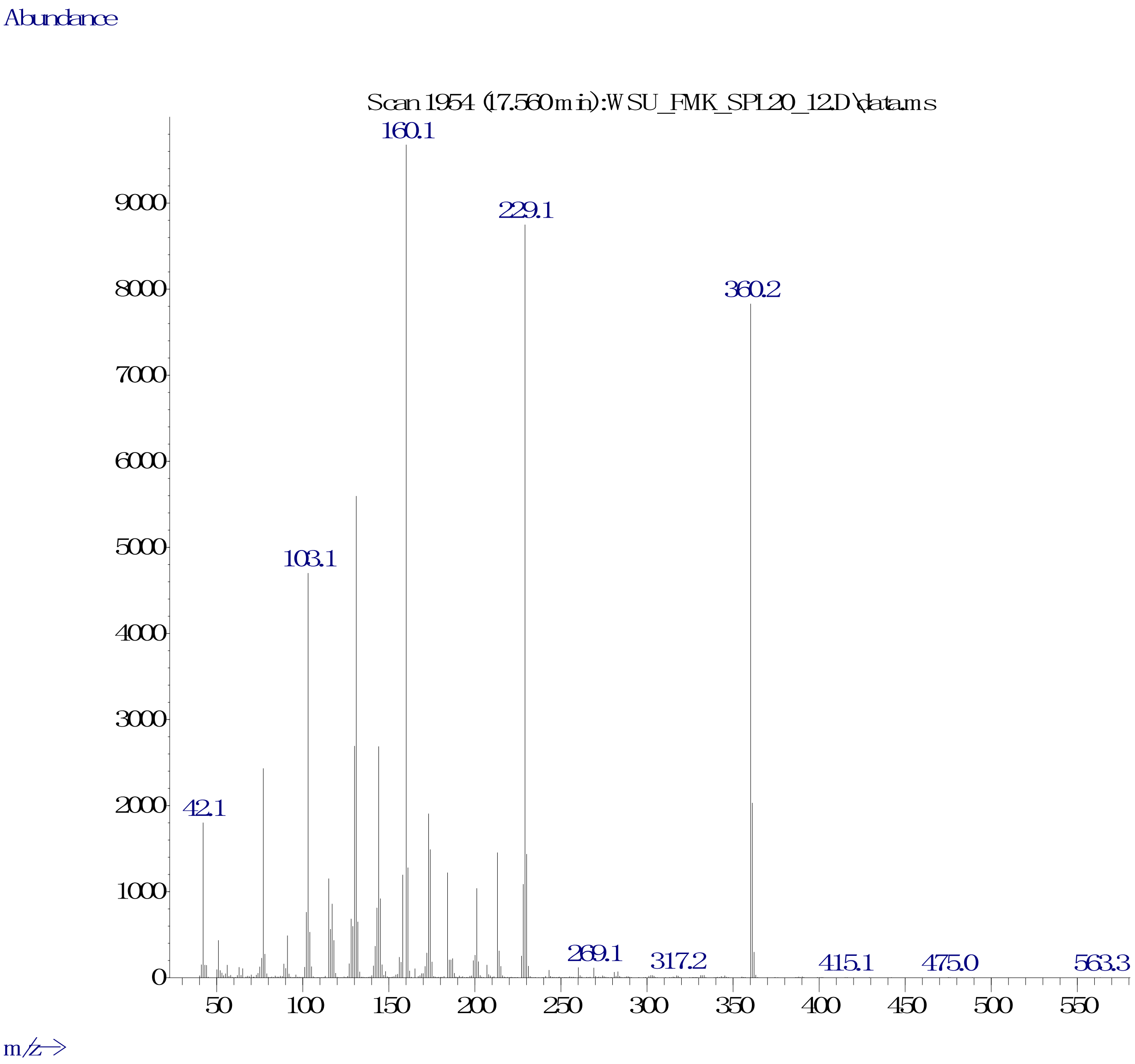

Mass spectrum (m/z) of **34**.

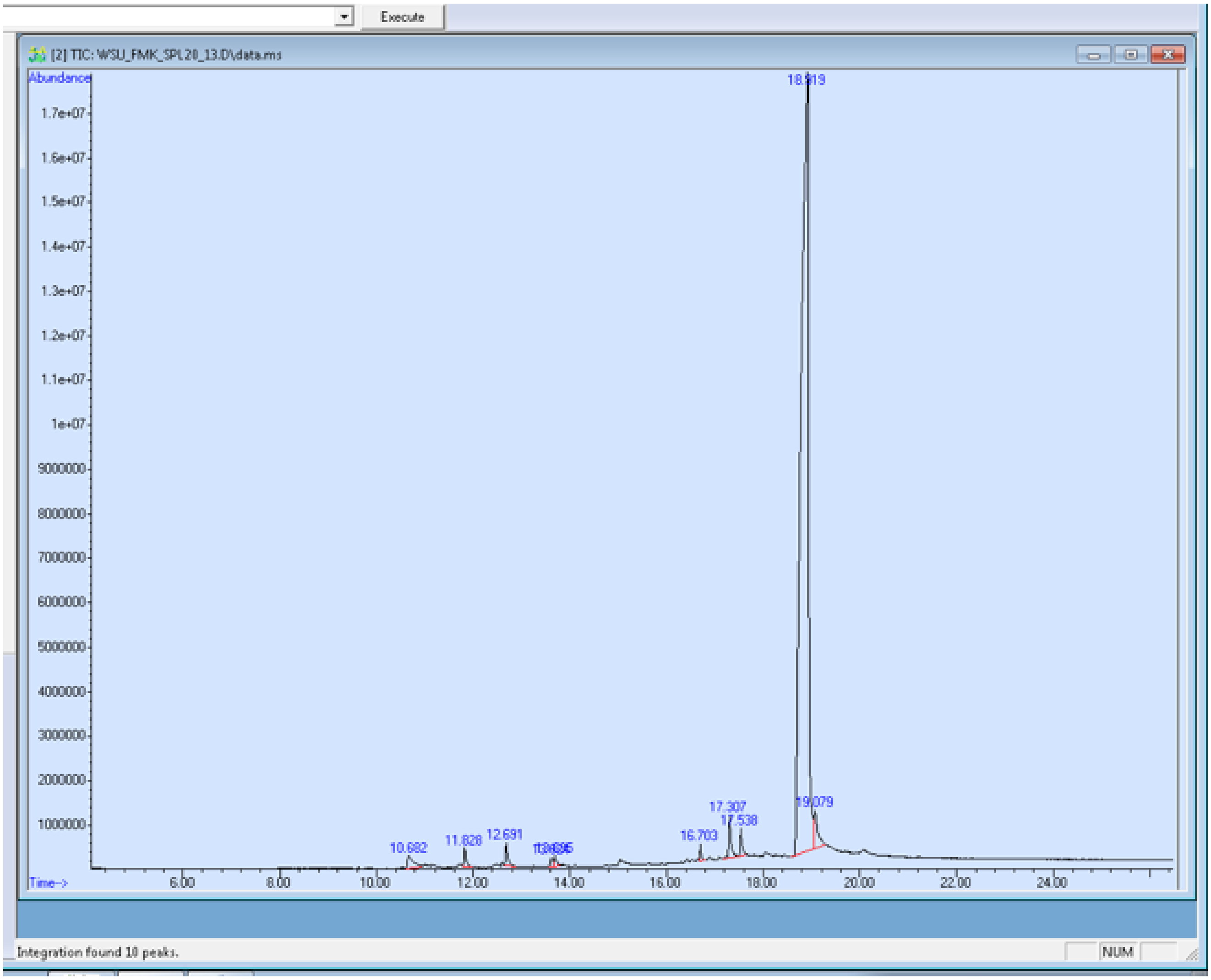

Chromatograph of **35**.

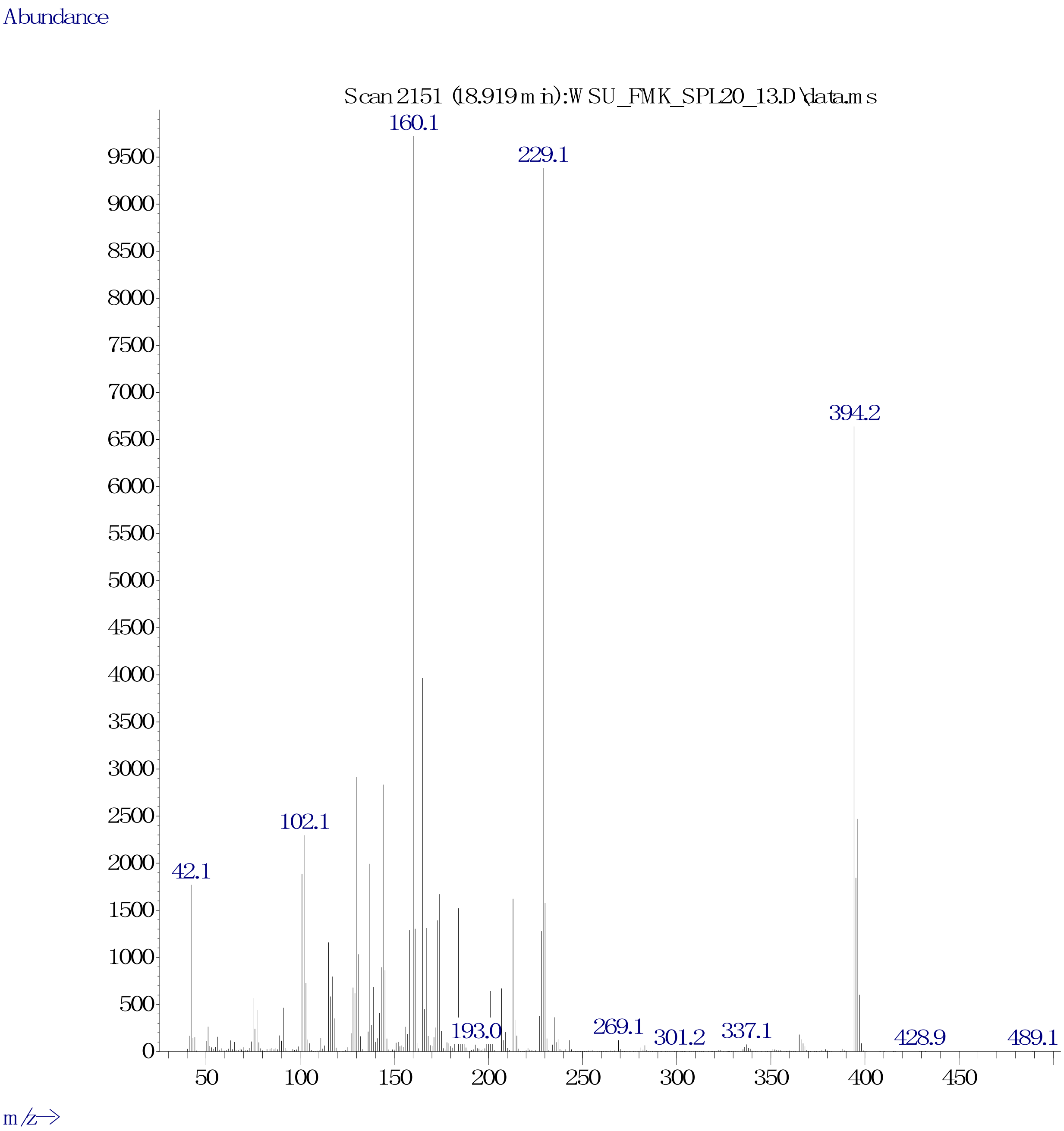

Mass spectrum (m/z) of **35**.

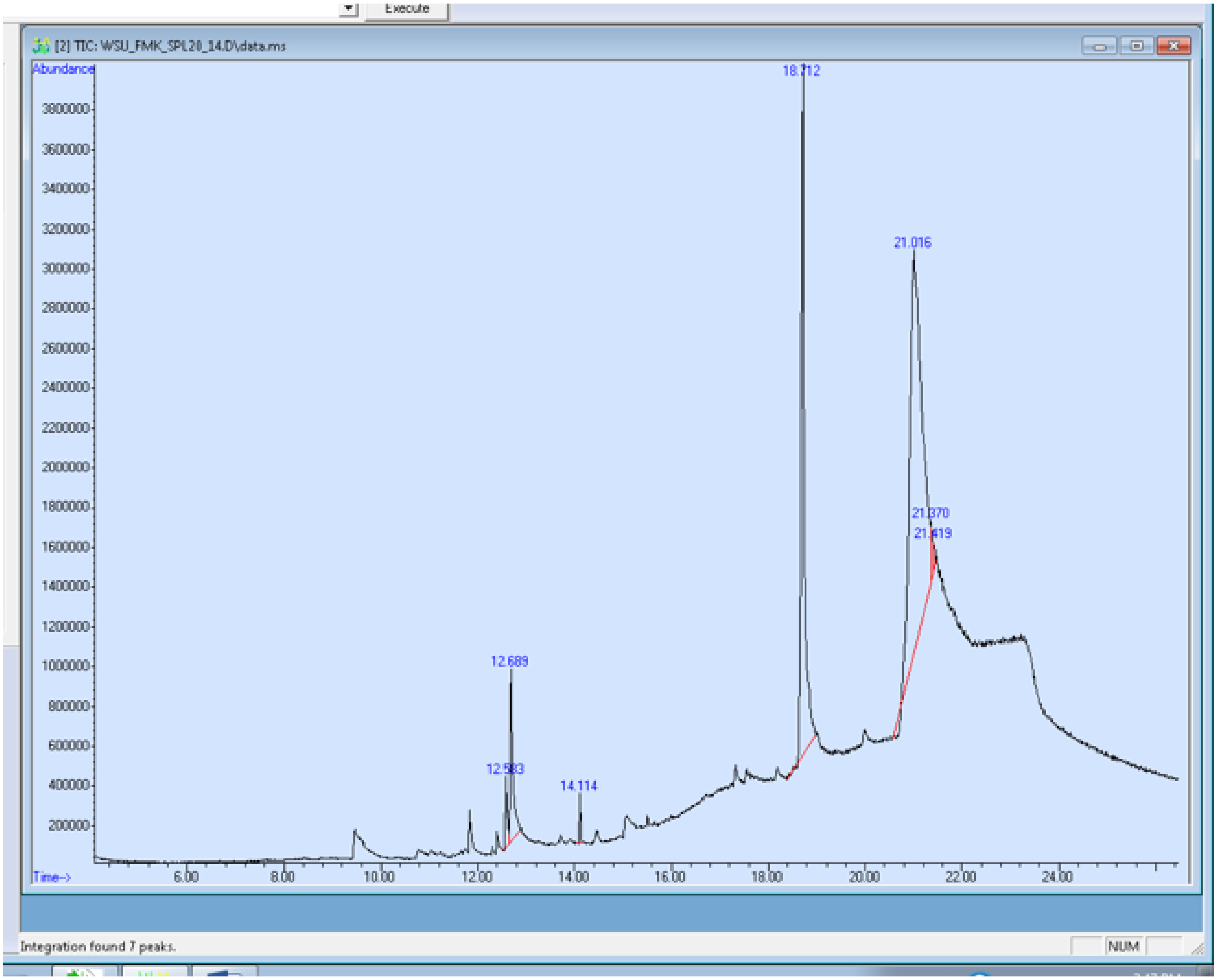

Chromatograph of **36**.

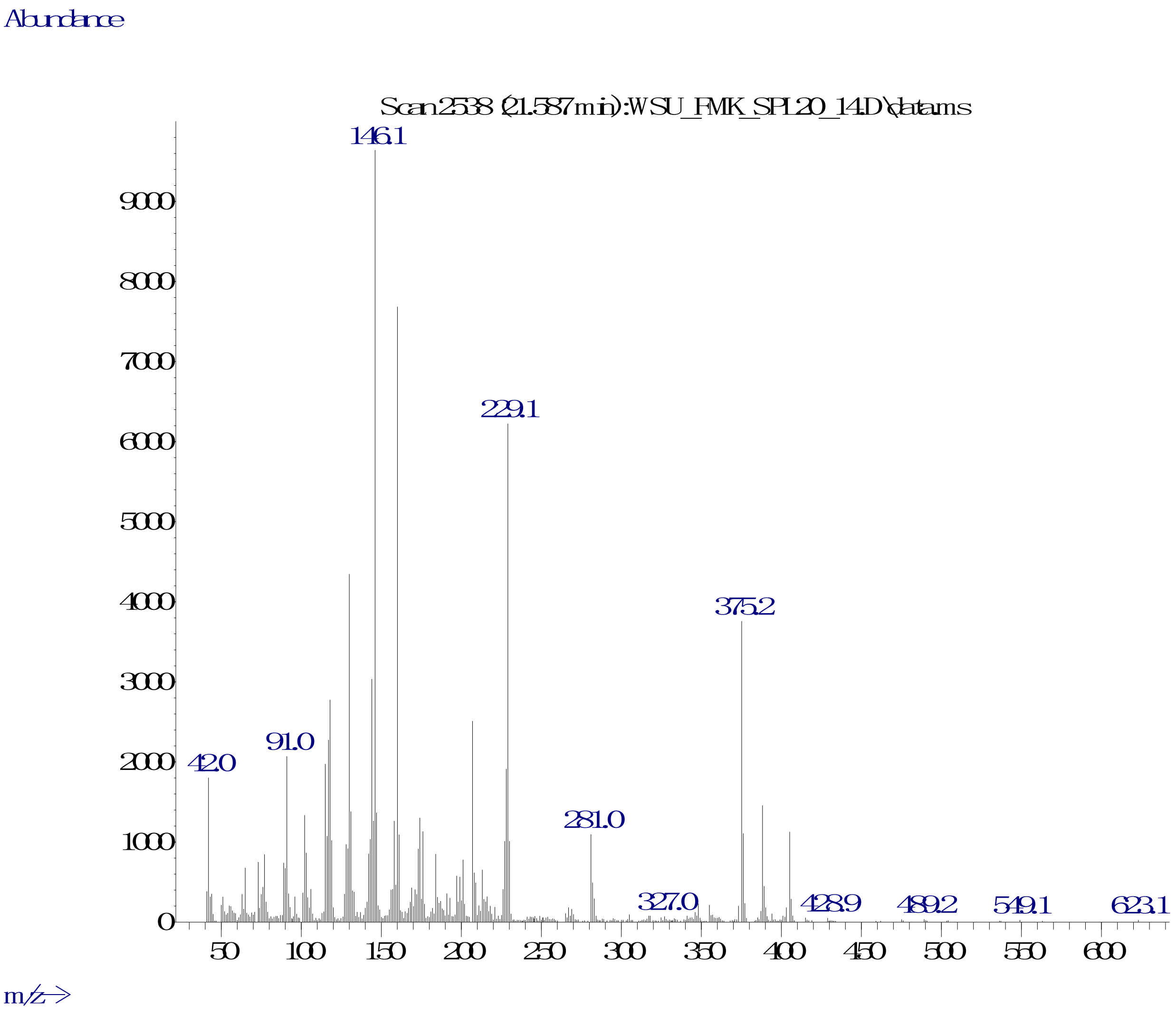

Mass spectrum (m/z) of **36**.

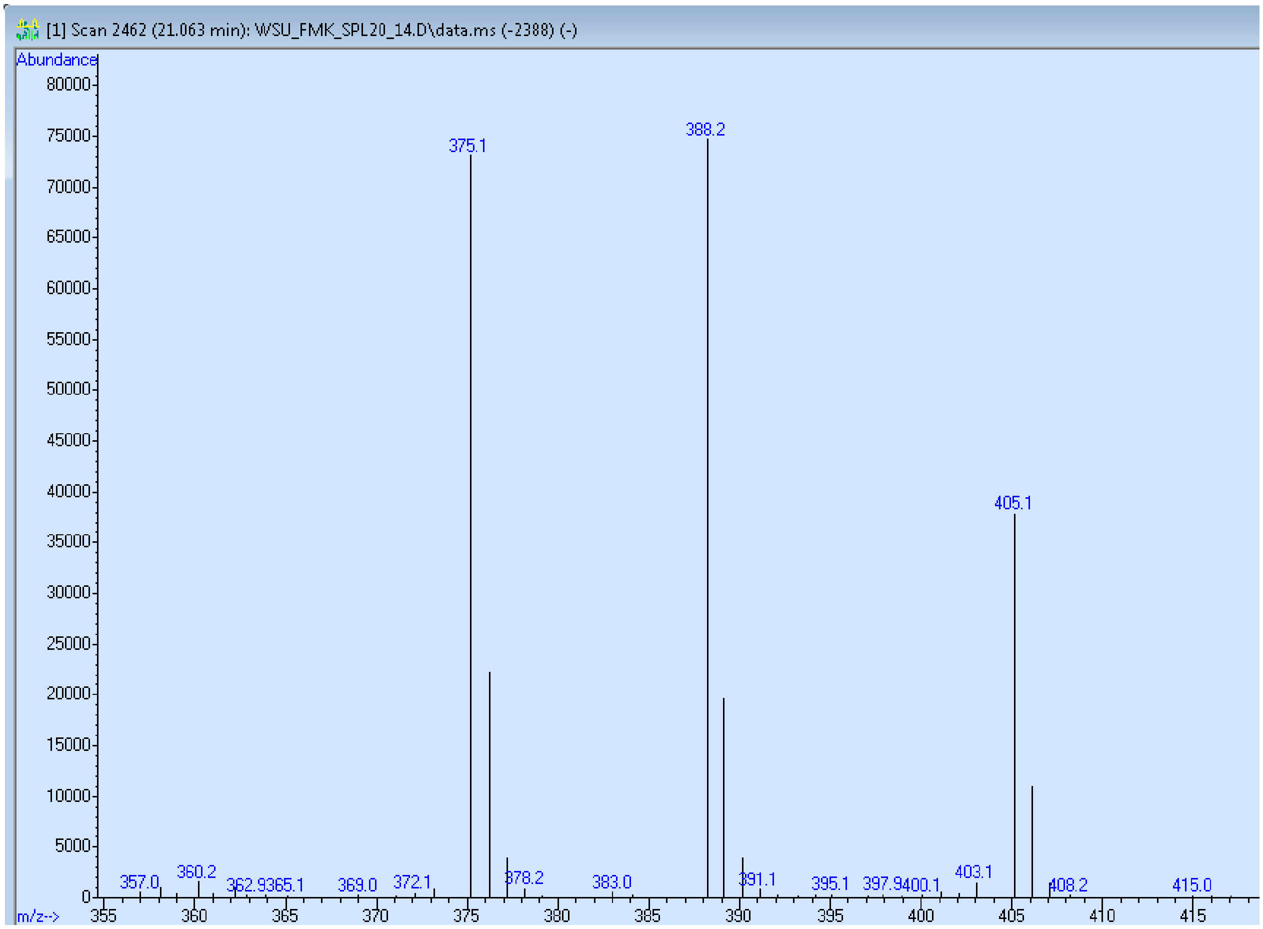

Mass spectrum (m/z) of **36**.

`

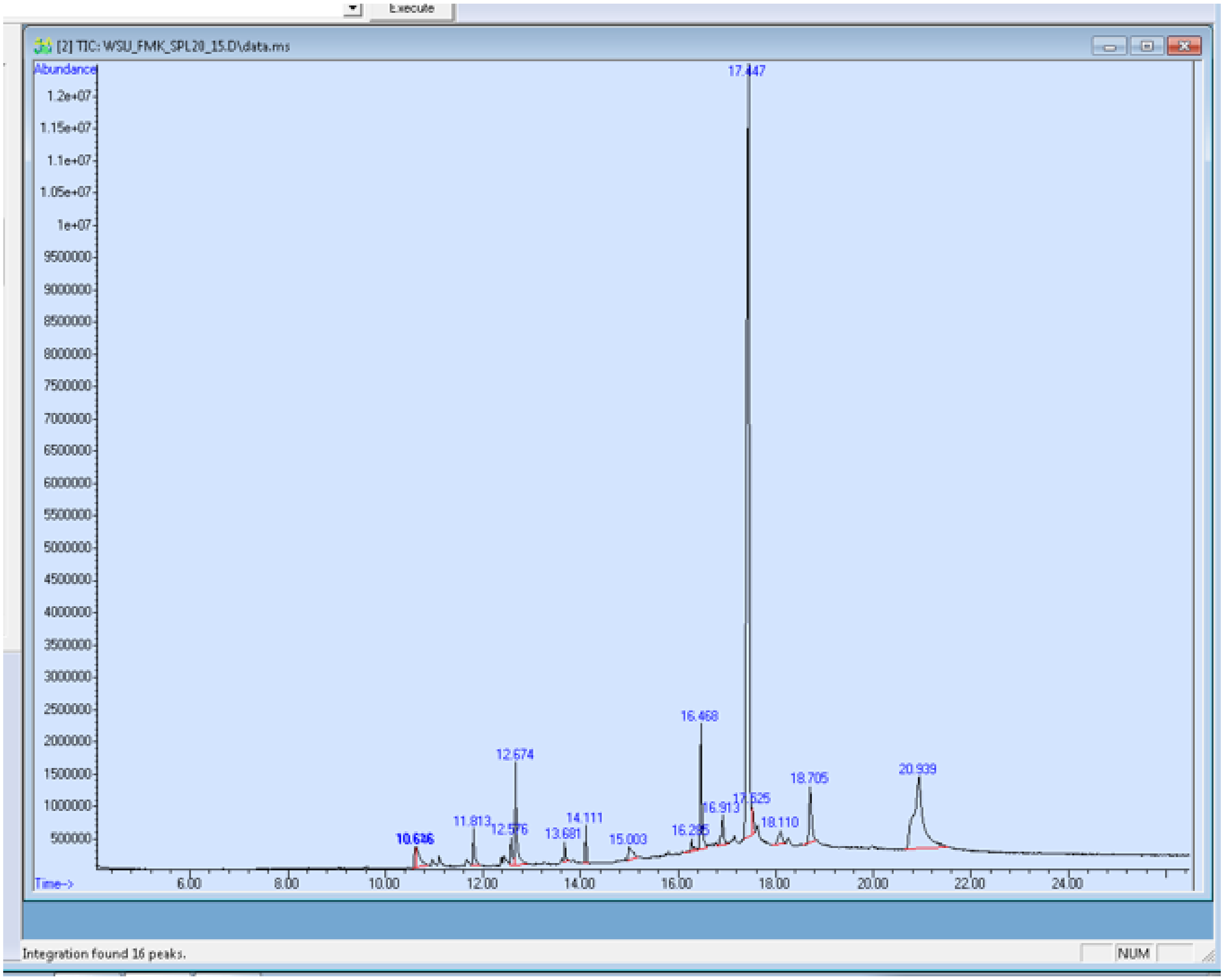

Chromatograph of **37**.

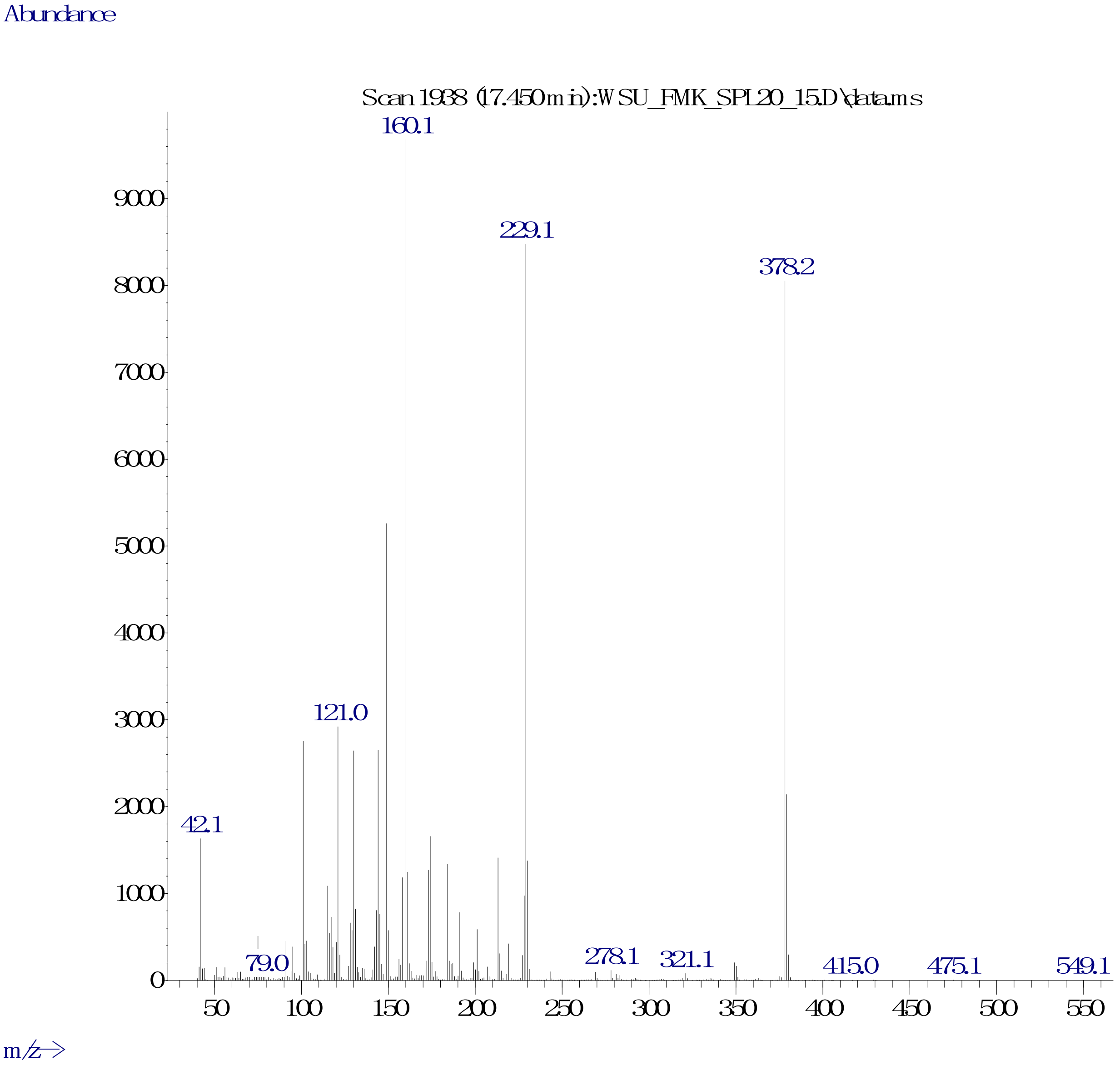

Mass spectrum (m/z) of **37**.

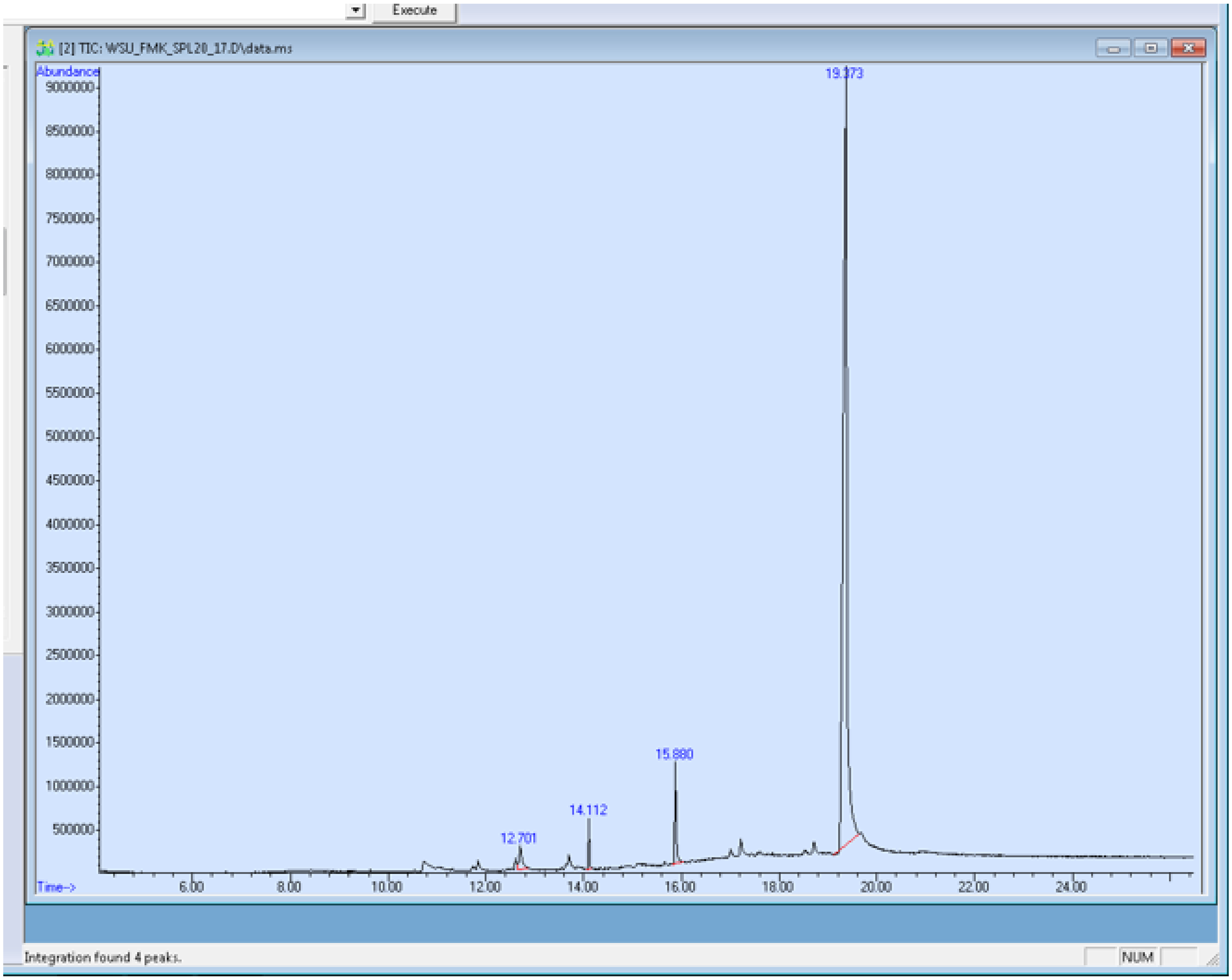

Chromatograph of **38**.

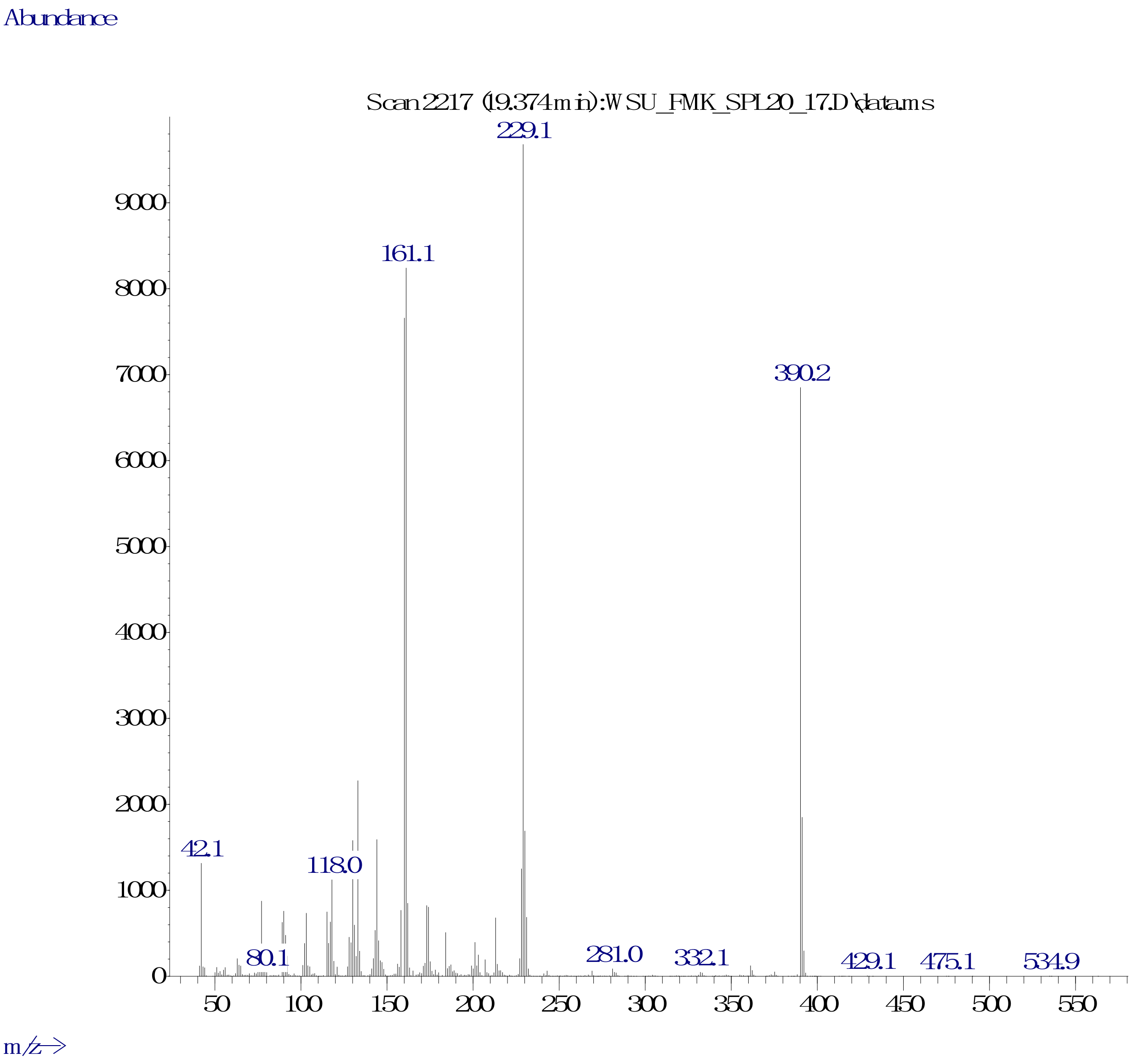

Mass spectrum (m/z) of **38**.

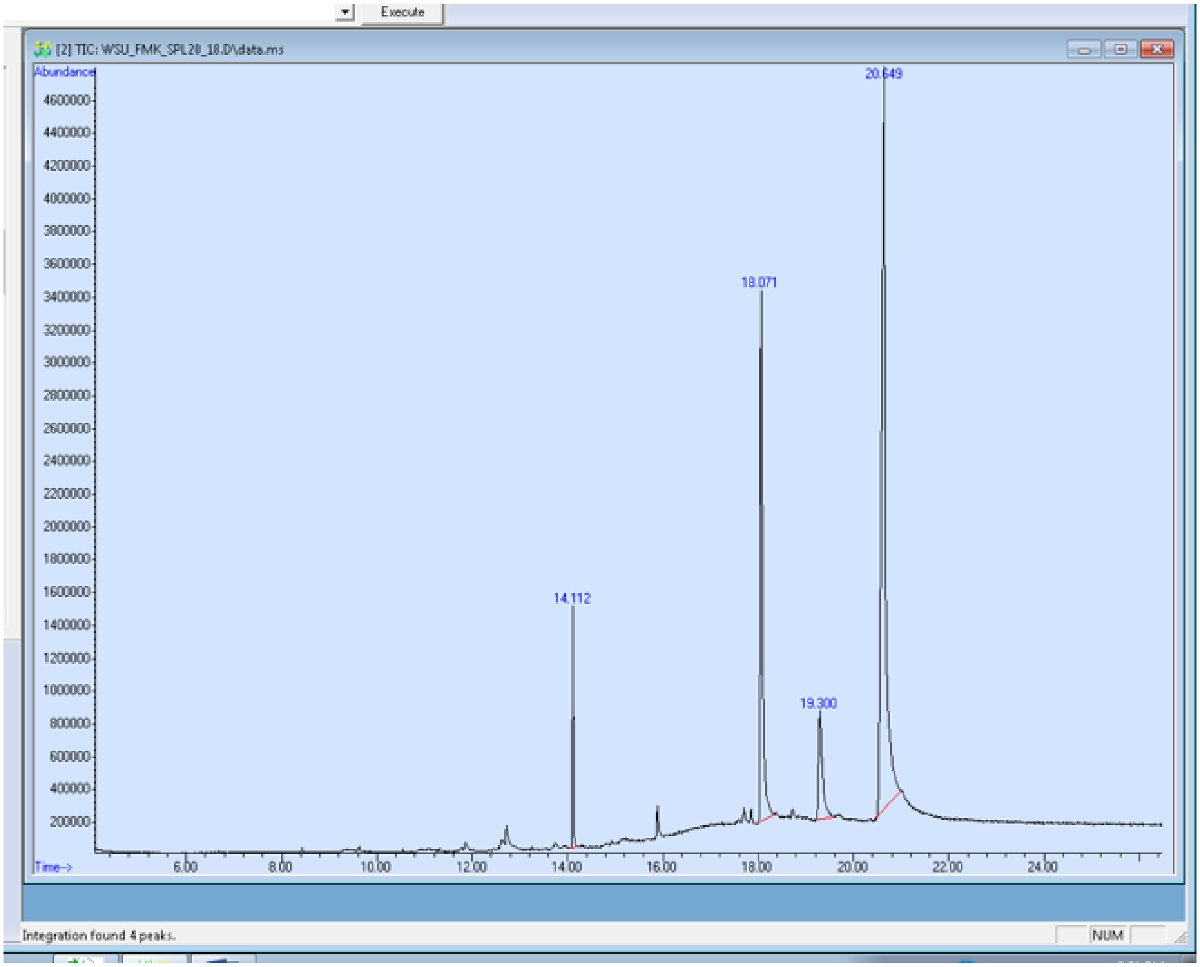

Chromatograph of **39**.

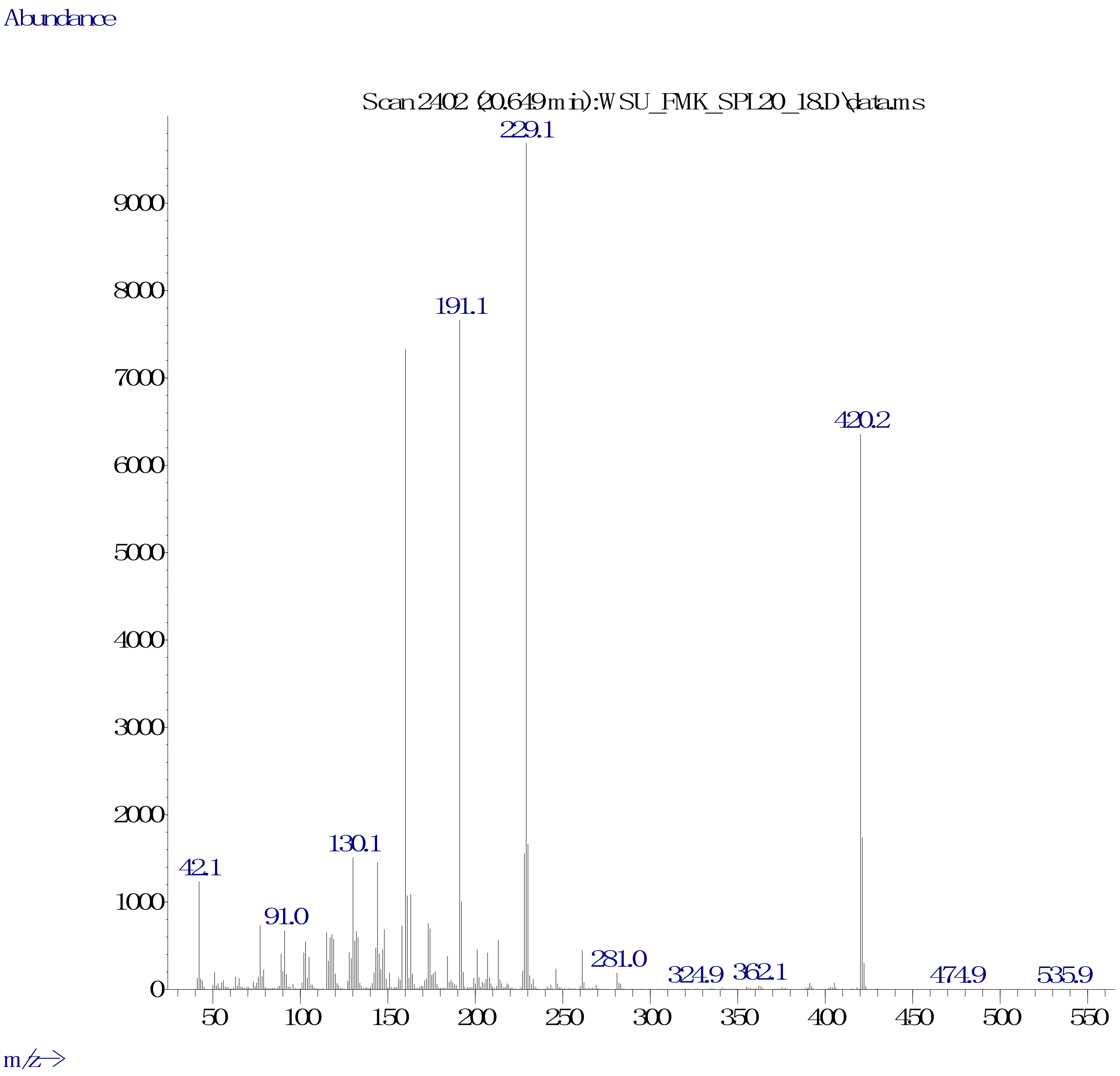

Mass spectrum (m/z) of **39**.

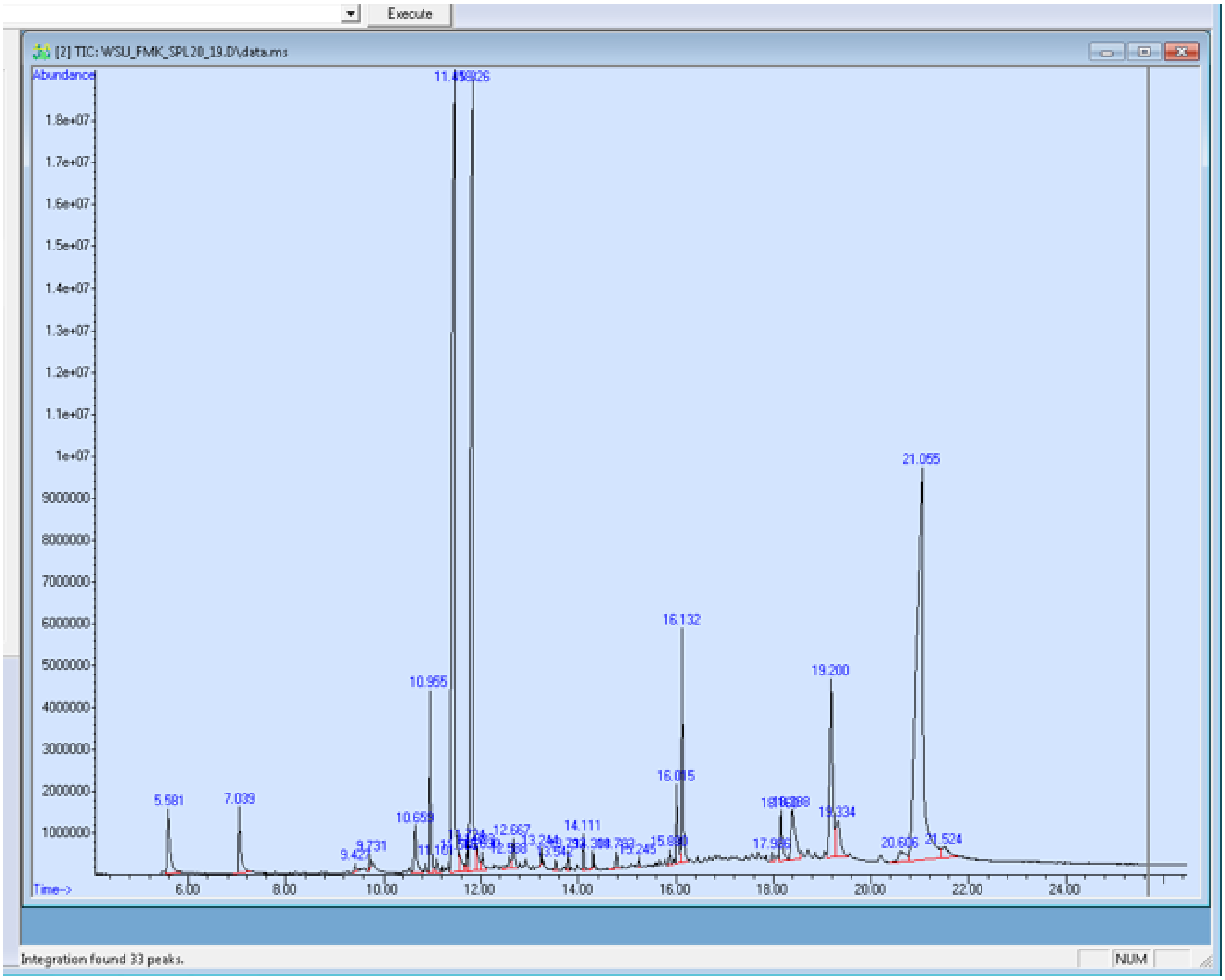

Chromatograph of **40**.

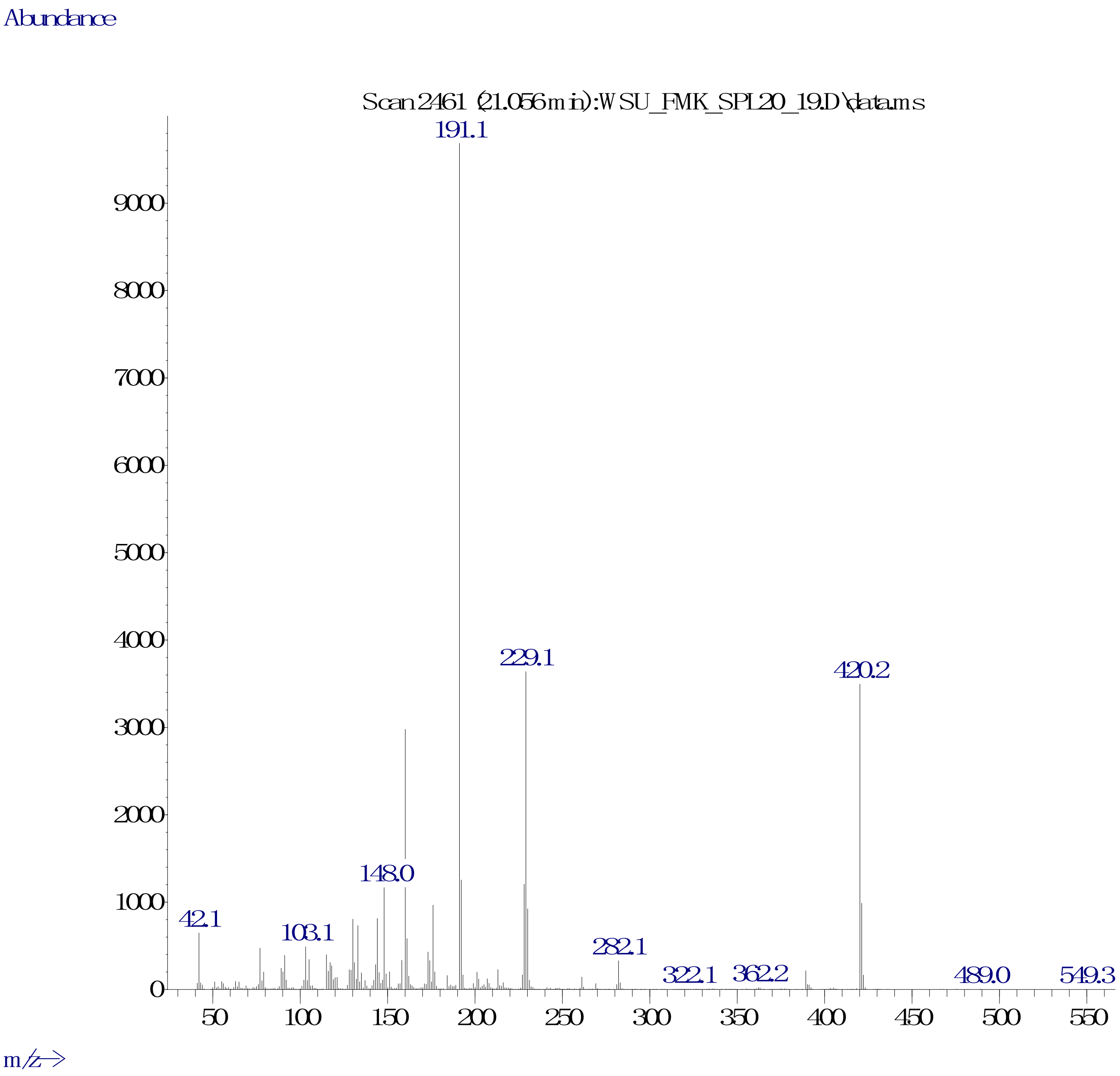

Mass spectrum (m/z) of **40**.
